# Synthetic Approach to Chromone and Flavonoid Piperidine Alkaloids

**DOI:** 10.1021/acs.joc.4c01926

**Published:** 2024-10-22

**Authors:** Karen A. Guarneros-Cruz, Silvano Cruz-Gregorio, Julio Romero-Ibañez, Rosa L. Meza-León, Fernando Sartillo-Piscil

**Affiliations:** Centro de Investigación en Síntesis Orgánica de la Facultad de Ciencias Químicas, Benemérita Universidad Autónoma de Puebla (BUAP), 14 Sur Esq. San Claudio, Col. San Manuel, 72570 Puebla, Mexico

## Abstract

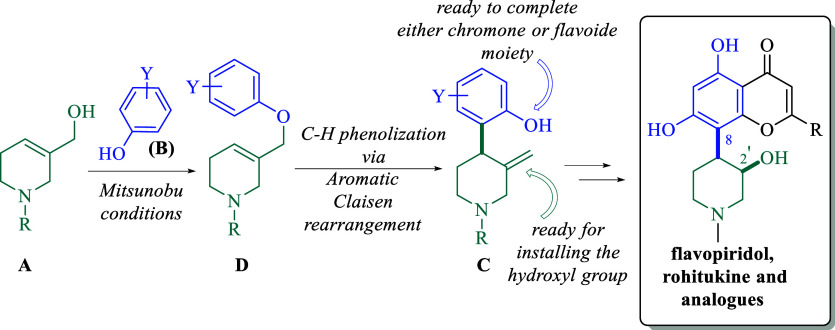

Despite the enormous importance of chromone and flavonoid piperidine alkaloids, a general method for their synthesis has not been described. Accordingly, from simple tetrahydro-3-pyridinemethanols (**A**) and phenol derivatives (**B**), a synthetic approach to chromone and flavonoid piperidine alkaloids is presented. The access to a novel chromone and flavonoid alkaloid precursors 4-(2-hydroxyphenyl)-3-methylenepiperidines (**C**) is achieved in only two steps: Mitsunobu reaction followed by an intramolecular C–H phenolization via an aromatic Claisen rearrangement of the respective Mitsunobu adducts (**D**). Consequently, the simultaneous installation of the functionalized phenol group and the exo-methylene group within the piperidine skeleton, permits, not only the easy construction of the chromone or flavonoid cores but also the simultaneous installation of the hydroxyl group with the required *cis*-orientation. Additionally, the synthetic utility of this novel approach is showcased in the formal synthesis of flavopiridol, rohitukine, and their *N*-Moc analogues.

## Introduction

**Figure 1:**
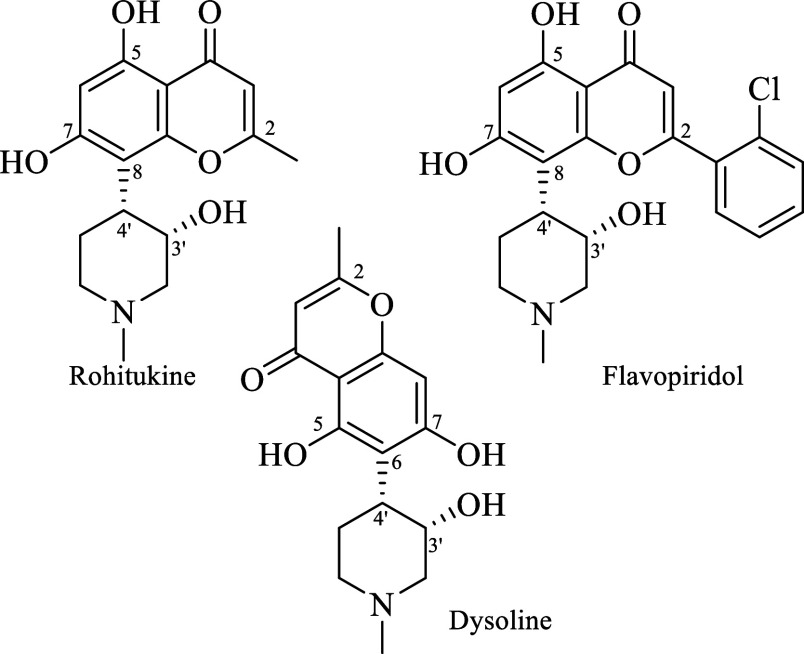
Rohitukine, flavopiridol and dysoline as representative examples of chromone and flavonoid alkaloids.

Chromone piperidine alkaloids^1^ belong to a selected class of natural products isolated from Maliaceae^2a,2b^ and Rubiaceae families,^2c^ and widely used in traditional medicine,^3^ especially in the Indian folk medicine.^4^ Since they have shown potent anticancer activity,^5^ scientists have designed flavonoid piperidine alkaloids, a type of synthetic alkaloids, that with the simple structural modification of changing a methyl group by an aryl group (e.g., rohitukine and flavopiridol; Figure 1), the anticancer activity is considerably increased.^6^ Presumably, the high anticancer activity of flavopiridol, is attributed to the chlorine atom,^6,7^ which enhance the interactions with the active site of CDK2, which induces cell cycle arrest in many cancer cells.^7^

Unlike general classification of alkaloids, which is based on the nitrogen skeleton, the chromone and flavonoid alkaloids are classified on the basis of their 5,7-dihydroxy-chromone (noreugenin) moiety linked to the piperidine ring.^1a^

Accordingly, rohitukine and dysoline, which possess a methyl group at C2 position, are isomeric chromones at C8–C4′ and C6–C4′, respectively; while the flavopiridol, with the aryl group at C2-position, is a C8–C4′ synthetic flavonoid alkaloid (Figure 1). Additionally, another relevant structural feature is the *cis*-oriented hydroxyl group at C3′ position.

Based on this brief structural analysis, it is clear that the major synthetic challenge to prepare these very important alkaloids (and analogues thereof), is the selective installation of the polyhydroxy phenol group at C4′ position and the C3′-hydroxyl group, both *cis*-oriented.

**Scheme 1:**
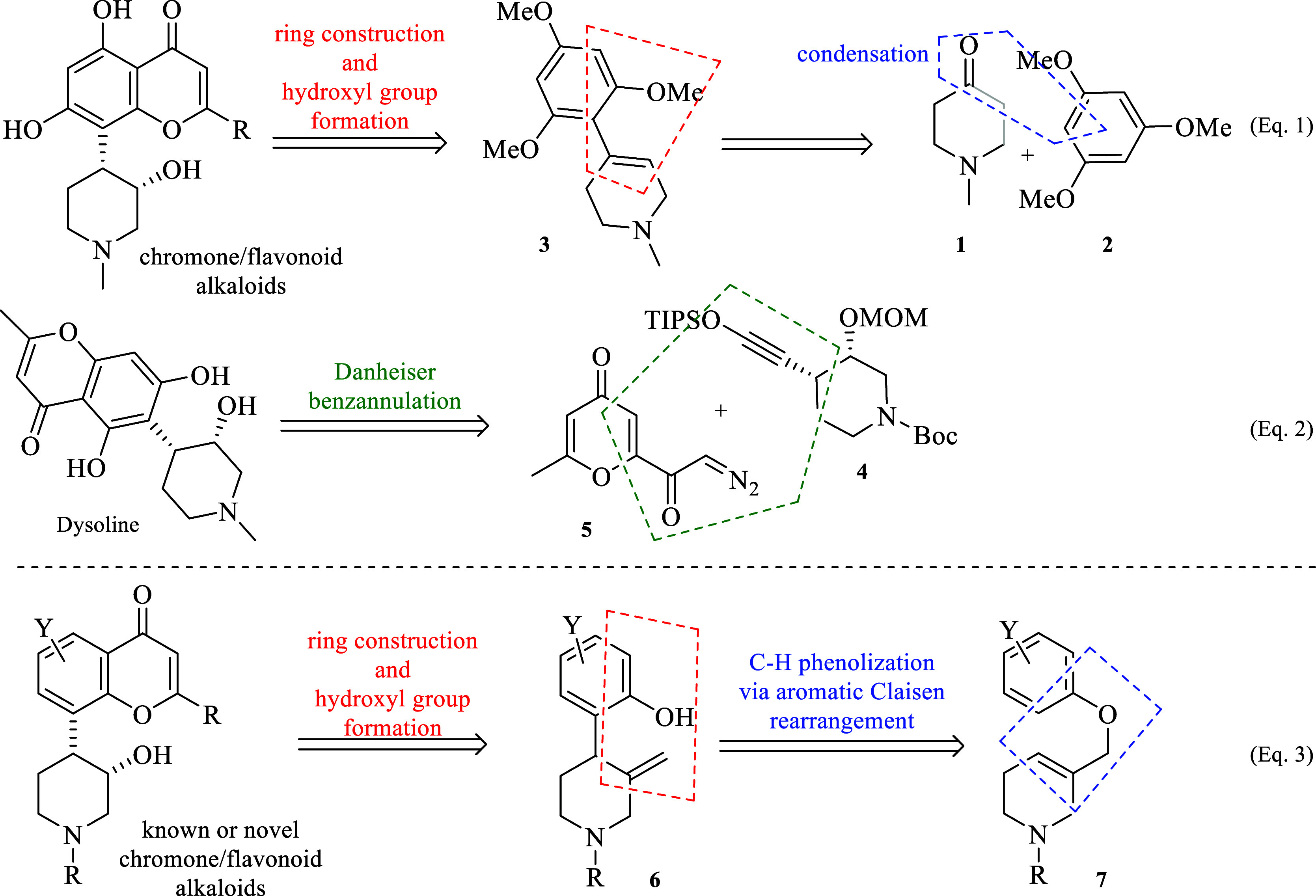
Existing Approaches for Chromone/Flavonoid Piperidine Alkaloids (eqs 1 and 2); Current Approach (eq 3)

In this regard, the condensation of 1-methyl-4-piperidinone **1** with 1,3,5-trimethoxybenzene **2** to unsaturated piperidine **3** followed by hydroboration, oxidation and reduction processes to set the C3′ hydroxyl group, is the standard synthesis procedure for C8–C4′ isomers (eq 1; Scheme 1).^8^ In contrast, an elegant asymmetric synthesis of chromone alkaloid dysoline (C6–C4′ isomer) was reported by Coffin and Ready,^9^ in which the construction of the chromone core was achieved by using the Danheiser benzannulation^10^ reaction between protected silyl ynol ether **4** and diazo ketone **5** (eq 2; Scheme 1). Although the two strategies are complementary, there are still shortcomings either on the need of preparing analogues for medicinal chemistry or with the long and complex routes for preparing precursors, such as ynol ether **4** and diazo keto **5**.

In this sense, a simple approach that enables rapid and modular access to either known or novel chromone/flavonoid piperidine alkaloids was envisioned based on an intramolecular C–H phenolization of tetrahydro-3-pyridinemethanol derivatives (**7**) to 4-(2-hydroxyphenyl)-3-methylenepiperidines (**6**) via an aromatic Claisen rearrangement reaction,^11^ followed by the construction of flavone/chromone core from the phenol group and the hydroxyl group with the required relative *cis* configuration from the concomitant exocyclic double-bond (eq 3; Scheme 1).

## Results and Discussion

**Scheme 2:**
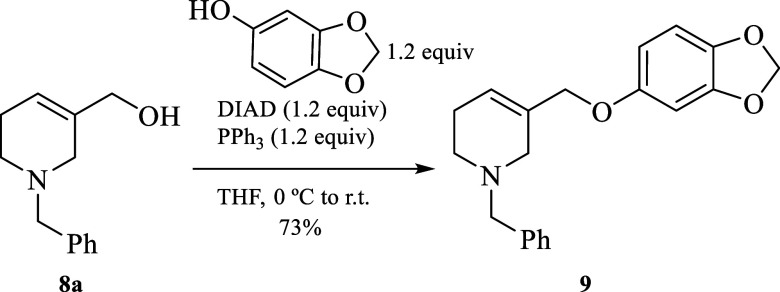
Preparation of the Aromatic Claisen Precursor **9**

The *N*-benzylated tetrahydro-3-pyridimethanol **8a**(12) and sesamol were selected as suitable partners for the preparation of the aromatic Claisen precursor **9** in good chemical yield through a standard Mitsunobu reaction conditions [(diisopropyl azodicarboxylate (DIAD) and triphenylphosphine (PPh_3_)] (Scheme 2).^13^

Once precursor **9** was prepared, thermal optimal reaction conditions for the aromatic Claisen rearrangement were investigated (Table 1; entries 1–10). The precursor **9** was dissolved and loaded inside a tube, which was sealed and submerged in an oil bath. Heating at 180 °C in CH_3_CN, the entire starting material **9** was transformed into adduct **10** in 180 min in a good chemical yield (entry 1). Increasing the temperature by 10 °C was enough to complete the rearrangement in 90 min with a better yield (entry 2). However, when increasing the temperature another 10 °C, the consumption of **9** occurred in only 40 min but at a lower yield (entry 3). Switching solvent to EtOAc and toluene gave similar results to those obtained with CH_3_CN (entries 4, 5, and 6); however, the lowest chemical yield was obtained with DMF (entry 7).

**Table 1:**
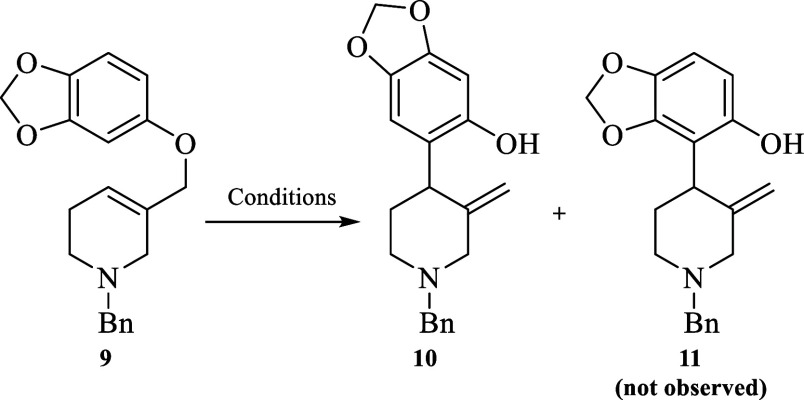
Aromatic Claisen Rearrangement Optimization of **9**

entry^a^	solvent	temperature (°C)	time (min)	yield of **10** (%)^b^
1^c^	CH_3_CN	180	180	65
2^c^	CH_3_CN	190	90	76
3^c^	CH_3_CN	200	40	66
4^c^	EtOAc	200	40	68
5^c^	EtOAc	200	60	80
6^c^	PhMe	200	40	70
7^c^	DMF	200	40	58
8^d^	CH_3_CN	200	40	60
9^d^	PhMe	200	40	92
10^d^	EtOAc	200	40	48

a Reaction scale at 0.14 mmol of **9**.

b Yields after purification.

c Conventional heating in an oil bath.

d Under microwave irradiation.

At this point we realized that the aromatic Claisen rearrangement is completed in 40 min at 200 °C with good chemical yields in all the solvents but DMF (entry 7); therefore, we decided to explore the rearrangement under microwave irradiation (entries 8–10). Accordingly, by using toluene as a solvent under microwave irradiation, the best chemical yield (92%) was obtained in 40 min (entry 9). Finally, it is important to remark that the regio-isomer **11** was not detected, which is consistent with the influence of the meta substituent in the regio-selectivity of this sigmatropic rearrangement.^14^

**Scheme 3:**
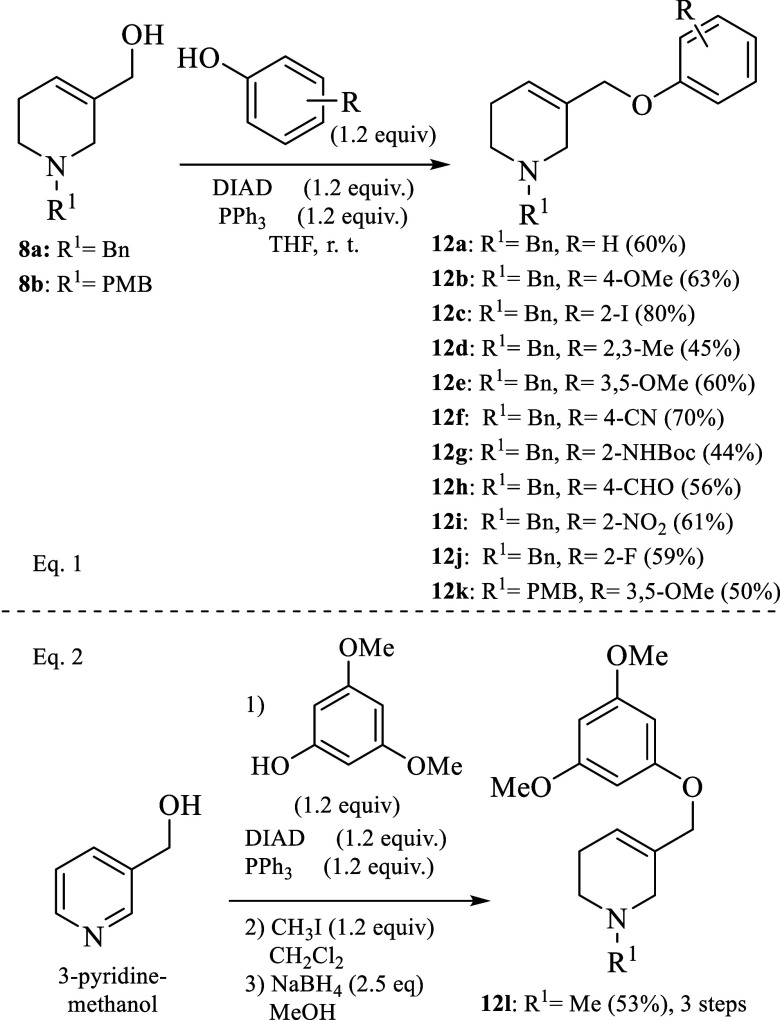
Synthesis of Claisen Precursors **12a**–**l**

Once the optimal reaction conditions for the aromatic Claisen rearrangement were determined, we proceeded to extend the scope of this aromatic Claisen rearrangement to a series of Claisen precursors **12a**–**l** containing both electron-withdrawing and electron-donating groups in all positions within the aromatic ring to, not only investigate the effect of the electronic nature of the substituents in the aromatic Claisen rearrangement but also to prepare potential valuable chromone and flavone precursors, which might be valuable for medicinal chemistry. Accordingly, **8a** and **8b** were transformed into **12a**–**k** in moderate to good yields by reacting with DIAD and PPh_3_ in THF at room temperature (eq 1; Scheme 3).^13^ The precursor **12l** was prepared from 3-pyridinemethanol following the procedure depicted Scheme 3; eq 2.

**Scheme 4:**
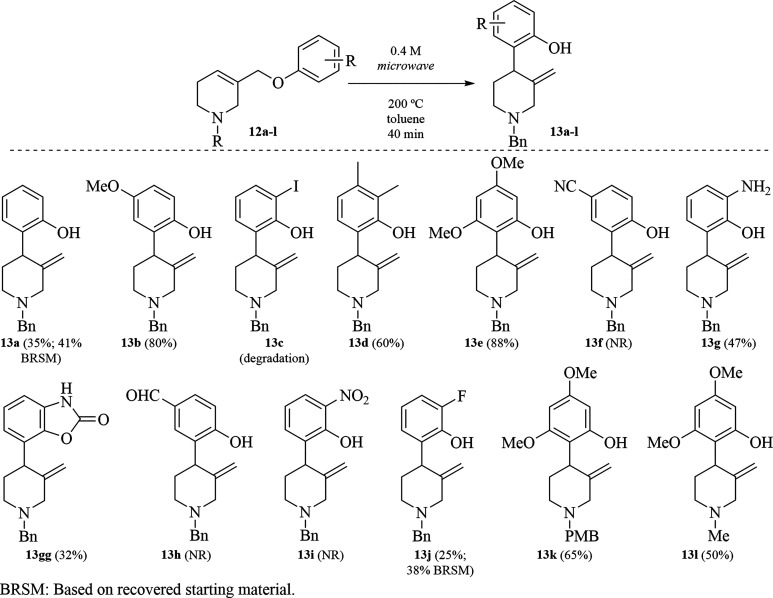
Intramolecular C**–**H Phenolization via Aromatic Claisen Rearrangement

Then, the aromatic Claisen rearrangement reaction conditions in toluene at 200 °C in a microwave reactor for 40 min were applied to **12a**–**l** (Scheme 4). As expected, the more activated the aromatic rings, the higher chemical yields are obtained, conversely with deactivated aromatic rings, negligible reactivity is observed. For the case R = H (**12a**), low chemical yield of **13a** was obtained; albeit a small amount of the starting material was recovered and reused (∼20% of **12a**) to increase the yield up to 41%. On the other hand, when the aromatic ring bears electron-donating substituents such as **9**, **12b**, **12d**, **12e**, **12g**, **12k** and **12l**, good to high chemical yields of their respective Claisen adducts (**10**, **13b**, **13d**, **13e**, **13g**/**13gg**, **13k** and **13l**) were obtained.

The combined chemical yield for the Claisen rearrangement of **12g** → **13g** plus **13gg** is 79% since both derive from the same Claisen adduct (not shown), from which, Boc protecting group is either removed (**13g**) or retained in the form of an oxazolidinone (**13gg**). In contrast, electron-withdrawing groups (**12f**, **12h** and **12i**), inhibit the formation of the Claisen adducts (**13f**, **13h** and **13i**). While halogens like iodide (**12c**) did not provide the respective Claisen adduct (**13c**), fluoride (**12j**) substituent behaves similar to simple phenyl group, and Claisen adduct **13j** is obtained in moderate yield. Other substituents at the nitrogen atom rather than Bn or *p*-methoxybenzyl (PMB), such as the methyl group, do not affect the Claisen rearrangement when the aromatic ring is activated (**13k** and **13l**).

**Scheme 5:**
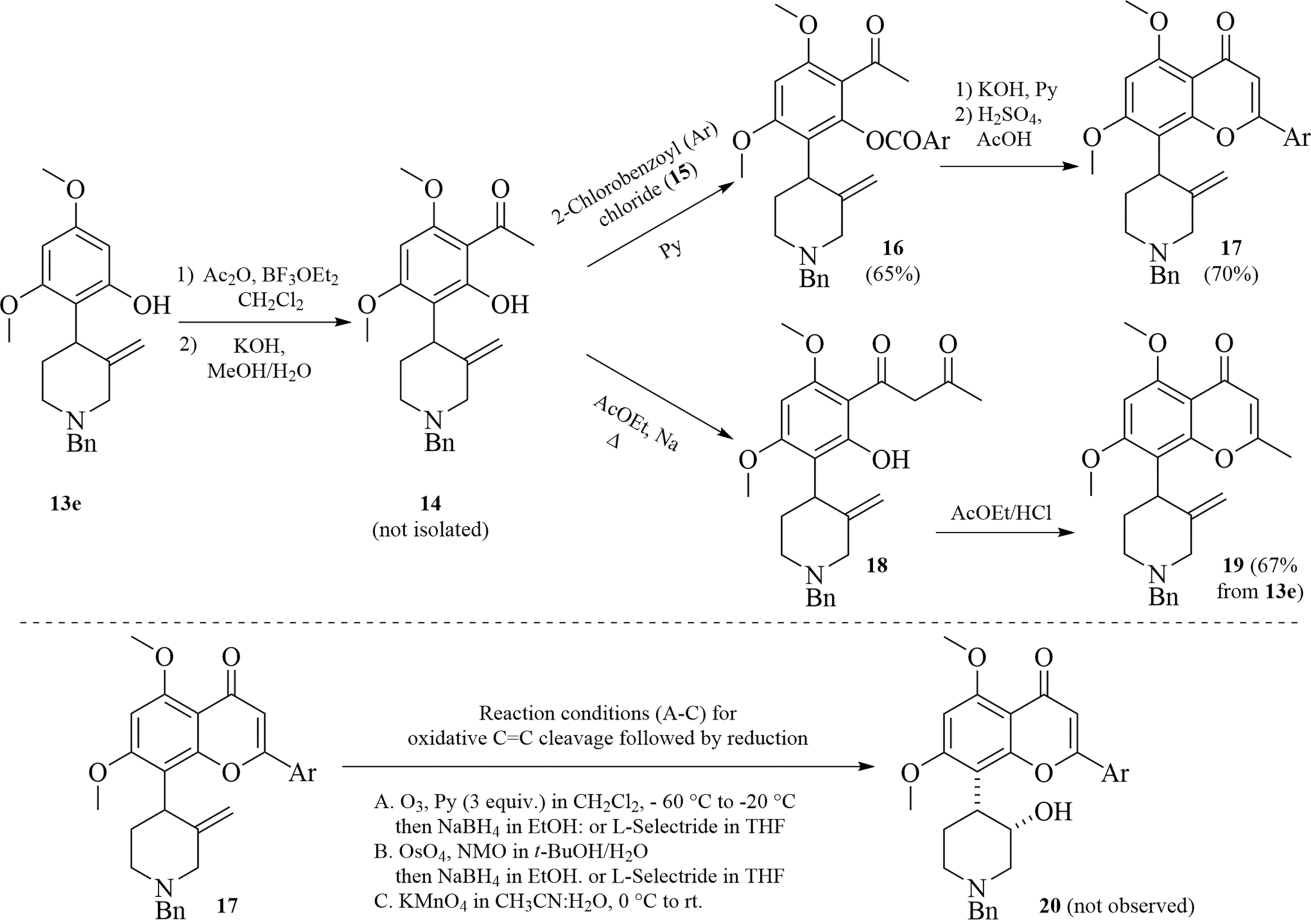
Construction of Flavone and Chromone Moiety and Attempts to Install the *cis*-Hydroxyl Group at C3′ of the Piperidine Ring

Thereafter, we proceeded to prepare both flavone and chromone cores from the aromatic ring of **13e** following reported protocols,^6,8^ to then install the C3′ hydroxyl group with the *cis* relative configuration at the expense of the exocyclic double-bond via an oxidative double-bond cleavage followed by carbonyl group reduction. Accordingly, Friedel–Crafts acylation of **13e** with acetic anhydride in the presence of BF_3_·OEt_2_ followed by base treatment with base (NaOH) afforded **14** quantitatively, which without further purification process, was subjected to either submitted, for one side to phenol acylation with 2-chlorobenzoyl chloride (**15**) to give **16**, and for another side or treated with ethyl acetate to produce **18**. Unlike arylated analogue **16**, intermediate **18** was directly transformed into chromone **19** under acidic conditions with high overall yield (67% from **13e**), while the aryl intermediate **16** had to be transformed into flavone derivative **17** in two sequential steps (see Scheme 5; above). Moreover, the installation of the hydroxyl group at C3′ position was not achieved as we planned. Attempts to cleavage the exocyclic double bond with ozone, OsO_4_ or KMnO_4_ were unsuccessful (Scheme 5; below). Although starting material **17** is consumed, the transient ketone intermediate (not shown) was neither isolated or trapped by subsequent reduction with NaBH_4_ or L-selectride. Probably, the premature N-oxidation and further decomposition is faster than the expected double-bond oxidation, therefore, product **20** was not observed.

**Scheme 6:**
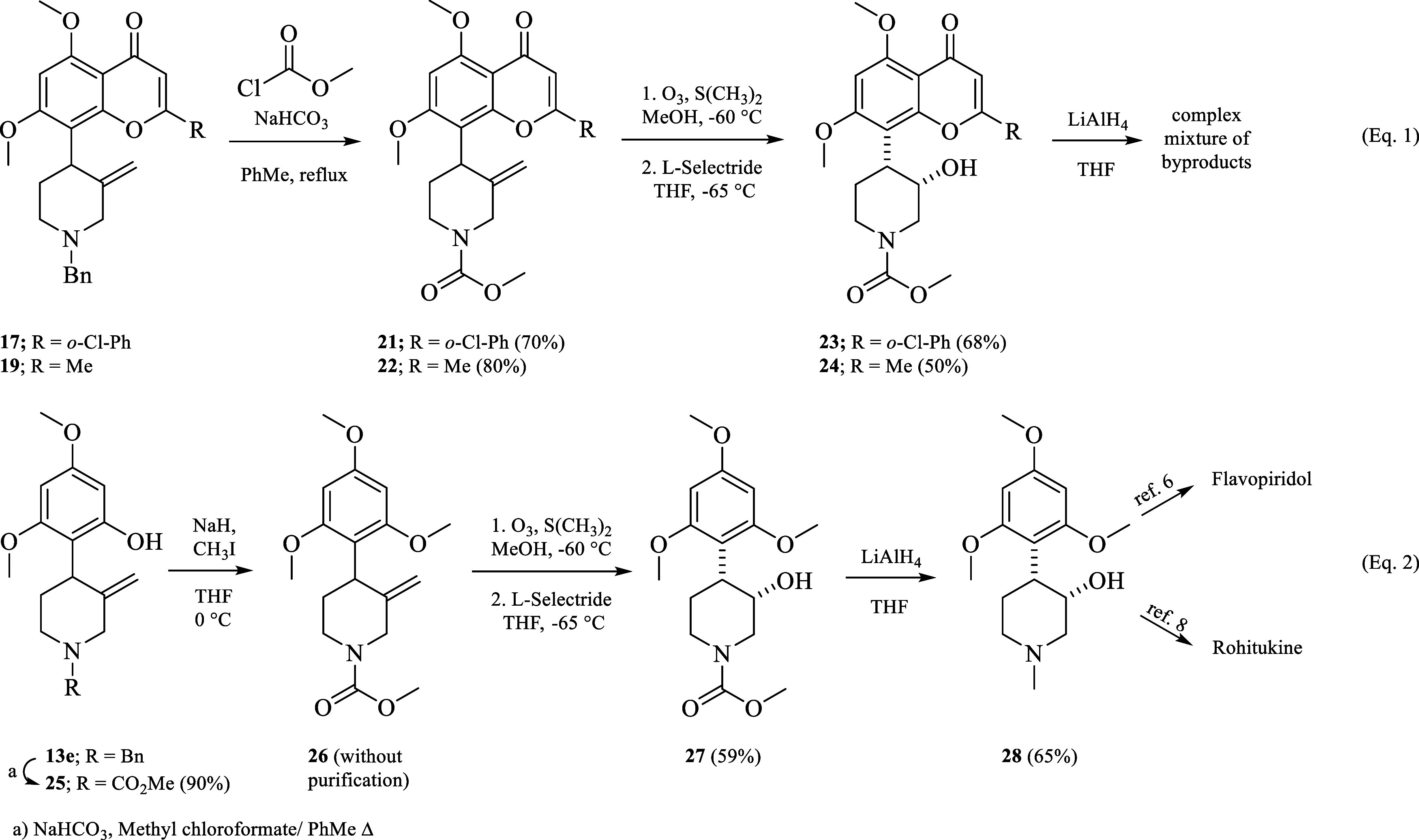
Synthesis of Flavopiridol-Moc and Rohitukine-Moc Analogues (**23** and **24**; eq 1); Formal Synthesis of Flavopiridol and Rohitukine (eq 2)

The problem was solved by designing novel chromone/flavone piperidine alkaloids containing a methyl carbamate (Moc) instead of a benzyl group (**23** and **24**, respectively), which led us to initiate a project for the search of novel flavopiridol and rohitukine analogs with anticancer activity.^15^ Thus, benzyl group of flavone **17** and chromone **19** was switched to Moc group (**21** and **22**, respectively) by using methyl chloroformate in the presence of NaHCO_3_.^12^ With the nitrogen atom deactivated, the installation of the *cis*-hydroxyl group at C3′ position was achieved in a sequential two-steps-one chromatographic purification: first ozone and dimethyl sulfide, then stereoselective carbonyl reduction with L-selectride to yield **23** and **24** in 68% and 50%, respectively. As expected, owing to both: the sterically bulky carbonyl group of the transitory ketone intermediates and the greater steric hindrance of the bulky hydride reagent, highly 3,4-*cis*-stereoselective reduction is observed; indeed, no traces of the *trans* diastereoisomer was detected (eq 1, Scheme 6). Additionally, we selected the *N*-Moc group because this group would also provide the *N*-Methyl group under reductive conditions.

With these two novel flavopiridol-Moc and rohitukine-Moc analogues in hand, we tried to convert them into flavopiridol and rohitukine by exhaustive reduction of the *N*-Moc group into *N*-methyl with LiAlH_4_; however, both the flavone and chromone core resulted to be more reactive than the Moc group, and a complex mixture of byproducts was obtained. In order to avoid struggling with the reactivity of the flavone and chromone core under strong reductive conditions, the synthesis of flavopiridol and rohitukine was achieved from *N*-methyl-4-(2,4,6-trimethoxyphenyl)piperidin-3-ol (**28**).

Accordingly, *N*-Moc piperidine **25** was first methylated to **26** with CH_3_I and NaH, and then, without further purification, was subjected to oxidative cleavage with ozone and S(Me)_2_ in methanol at −60 °C followed by carbonyl reduction with L-selectride to obtain *N*-Moc-(2,4,6-trimethoxyphenyl)piperidin-3-ol 27 in 59% overall yield. Finally, the reduction of **27** under the same reaction conditions as for **23** and **24**, afforded the synthetic intermediate **28**, which its NMR data is consistent with the literature values.^8a,16^ Finally, **28** could conduct to flavopiridol and rohitukine in only three or four steps following the reported procedures (eq 2, Scheme 6).^8a,8b^

## Conclusions

In summary, we have developed a modular approach that allows the installing of functionalized phenol groups at C4 position and a concomitant C3 *exo*-methylene group within the piperidine ring for the straightforward construction of the chromone/flavone skeleton and the C3-hydroxyl group with the required *cis*-relationship. The efficient aromatic Claisen rearrangement with electron-donating groups in the aromatic rings, such as OR, NHR and CH_3_ groups, and also with moderated deactivating atoms like F, provides a novel synthetic approach to a number of novel chromone and flavonoid piperidine alkaloids that might be anticancer candidate drugs. In this regard, besides the synthetic application of this novel approach to the formal synthesis of flavopiridol and rohitukine, we also introduced two novel flavopiridol and rohitukine analogues which, along with others not reported herein, are in process of patent application and will be disclosed in due course.

## Experimental Section

### General Considerations

Commercially available reagents were purchased from Sigma-Aldrich and used without further purification. Unless otherwise noted, reactions were carried out under an inert argon atmosphere with dry solvents under anhydrous conditions. Reactions were monitored by thin-layer chromatography (TLC). Purifications of products were performed by column chromatography using silica gel (230–400 mesh). Microwave synthesis was performed in the microwave reactor CEM Discover System (model 908005) in a sealed vessel. Claisen arrangement reactions were also carried out in a sealed tube and heated in an oil bath at 200 °C. The temperature was regulated using a digital contact thermometer. NMR spectra were obtained on Bruker-500 (500 MHz) spectrometer using TMS as an internal reference for ^1^H (0.00 ppm) and CDCl_3_ for ^13^C (77.16 ppm) unless otherwise noted. Chemical shifts (δ) are stated in parts per million (ppm) and Hz for the coupling constants (*J*). The following abbreviations were used to explain the multiplicities: s = singlet, d = doublet, t = triplet, q = quartet, m = multiplet, br = broadened, dd = doublet of doublets, td = triplet of doublets, qd = quartet of doublets. Structural assignments were made with additional information from gCOSY, gHSQC, and gHMBC experiments. Melting points were not corrected and carried out on a Fisher-Scientific 12–144 melting point apparatus. High-resolution mass spectra-electron impact mode (HRMS-EI). High-resolution mass spectra-fast atom bombardment mode (HRMS-FAB). High-resolution mass spectra-electrospray ionization mode (HRMS-ESI).

#### General Procedure for the Preparation of (1,2,5,6-Tetrahydropyridin-3-yl)methanol **8a**–**8b**

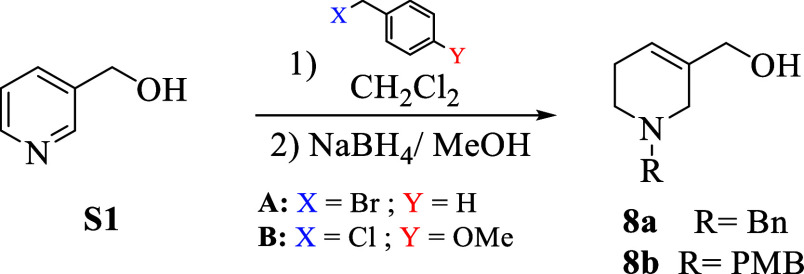



The tetrahydropyridines **8a** and **8b** were prepared using the procedure reported by Winkler et al.^12^. The pyridine-3-methanol **S1** (8.0 g, 73.31 mmol) was treated with the corresponding benzyl halide (76.97 mmol) in CH_2_Cl_2_ anhydrous (27.0 mL). The crude mixture was treated with NaBH_4_ (6.10 g, 161.27 mmol) in MeOH (80.0 mL) at 0 °C for 12 h to obtain tetrahydropyridines **8a** and **8b** after quenching and purification processes.

##### (1-Benzyl-1,2,5,6-tetrahydropyridin-3-yl)methanol (**8a**)

Following the general protocol, **S1** and benzyl bromide (9.16 mL) were used to obtain **8a**. The residue was purified with SiO_2_ column chromatography and eluted with hexanes/EtOAc (1:1) to obtain 13.70 g of **8a** (92%) as a yellow oil. *R*_*f*_ = 0.16 (hexanes/EtOAc = 1:1); ^1^H NMR (500 MHz, CDCl_3_): δ 7.30 (m, 4H), 7.24 (m, 1H), 5.53 (m, 1H), 3.88 (s, 2H), 3.57 (s, 2H), 2.93 (apparent s, 2H), 2.50 (t, *J* = 5.8 Hz, 2H), 2.11 (br, 2H). ^13^C{^1^H} NMR (125 MHz, CDCl_3_): δ 137.2 (C), 136.5 (C), 129.6 (2CH), 128.2 (2CH), 127.2 (CH), 120.4 (CH), 64.7 (CH_2_), 62.9 (CH_2_), 52.8 (CH_2_), 49.6 (CH_2_), 25.4 (CH_2_).

##### (1-(4-Methoxybenzyl)-1,2,5,6-tetrahydropyridin-3-yl)methanol (**8b**)

Following the procedure, **S1** and 4-methoxybenzyl chloride (10.4 mL) were used to obtain **8b**. The residue was purified with SiO_2_ column chromatography and eluted with hexanes/EtOAc (1:1) to obtain 15.40 g of **8b** (90%) as a yellow oil. *R*_*f*_ = 0.16 (hexanes/EtOAc = 1:1); ^1^H NMR (500 MHz, CDCl_3_): δ 7.24 (d, *J* = 8.5 Hz, 2H), 6.85 (d, *J* = 8.5 Hz, 2H), 5.55 (m, 1H), 3.90 (s, 2H), 3.78 (s, 3H), 3.52 (s, 2H), 2.92 (s, 2H), 2.49 (t, *J* = 5.8 Hz, 2H), 2.12 (br, 2H). ^13^C{^1^H} NMR (125 MHz, CDCl_3_): δ 158.8 (C), 136.6 (C), 130.9 (2CH), 129.3 (C), 120.5 (CH), 113.6 (2CH), 64.9 (CH_2_), 62.3 (CH_2_), 55.3 (CH_3_), 52.8 (CH_2_), 49.5 (CH_2_), 25.5 (CH_2_). HRMS-EI *m*/*z*: [M]^+^ calcd for C_14_H_19_NO_2_, 233.1416; found, 233.1434.

#### General Procedure for the Mitsunobu Reaction

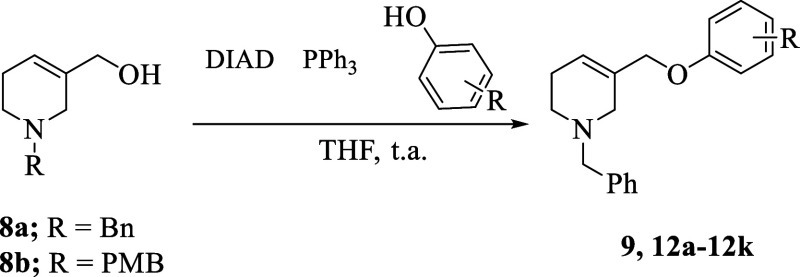



To a solution of the tetrahydropyridine (0.25 mmol), triphenylphosphine (0.30 mmol), and the corresponding phenol (0.30 mmol) in THF anhydrous (3.0 mL) at 0 °C, under argon atmosphere, was added dropwise DIAD (0.30 mmol). The reaction mixture was stirred for 15 min at 0 °C and warmed to room temperature. Once the raw material was consumed, the solvent was removed under reduced pressure and the residue was treated with 1 N NaOH solution. The aqueous layer was extracted with EtOAc (3 × 10 mL), and the organic phase was dried with Na_2_SO_4_ and concentrated for its purification by column chromatography.

##### 5-((Benzo[*d*][1,3]dioxol-5-yloxy) methyl)-1-benzyl-1,2,3,6-tetrahydropyridine (**9**)

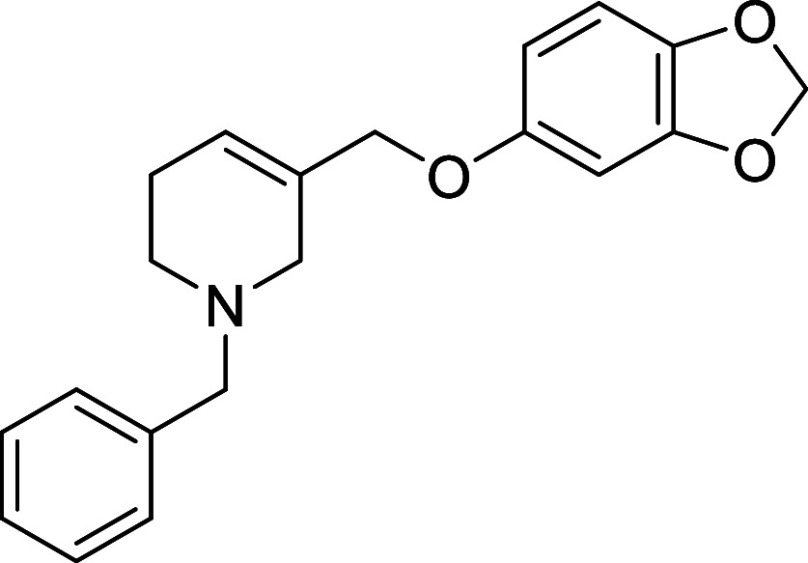



Following the general protocol for the Mitsunobu reaction, 59.0 mg of **9** (73%) was obtained as a brown oil from **8a** (50.0 mg, 0.25 mmol) and sesamol (41.0 mg, 0.30 mmol). Purified with SiO_2_ column chromatography and eluted with hexanes/EtOAc (6:1). *R*_*f*_= 0.10 (hexanes/EtOAc = 3:1); ^1^H NMR (500 MHz, CDCl_3_): δ 7.32 (m, 4H), 7.25 (m, 1H), 6.67 (d, *J* = 8.0 Hz, 1H), 6.48 (d, *J* = 2.5 Hz, 1H), 6.30 (dd, *J* = 8.5, 2.5 Hz, 1H), 5.89 (s, 2H), 5.84 (m, 1H), 4.30 (s, 2H), 3.62 (s, 2H), 3.04 (s, 2H), 2.55 (t, *J* = 5.8 Hz, 2H), 2.20 (br, 2H). ^13^C{^1^H} NMR (125 MHz, CDCl_3_): δ 154.4 (C), 148.3 (C), 141.8 (C), 138.3 (C), 132.7 (C), 129.3 (2CH), 128.4 (2CH), 127.2 (CH), 124.2 (CH), 108.0 (CH), 106.1 (CH), 101.2 (CH_2_), 98.4 (CH), 71.9 (CH_2_), 62.8 (CH_2_), 53.5 (CH_2_), 49.4 (CH_2_), 25.9 (CH_2_). HRMS-EI *m*/*z*: [M]^+^ calcd for C_20_H_21_NO_3_ 323.1521; found, 323.1513.

##### 1-Benzyl-5-(phenoxymethyl)-1,2,3,6-tetrahydropyridine (**12a**)

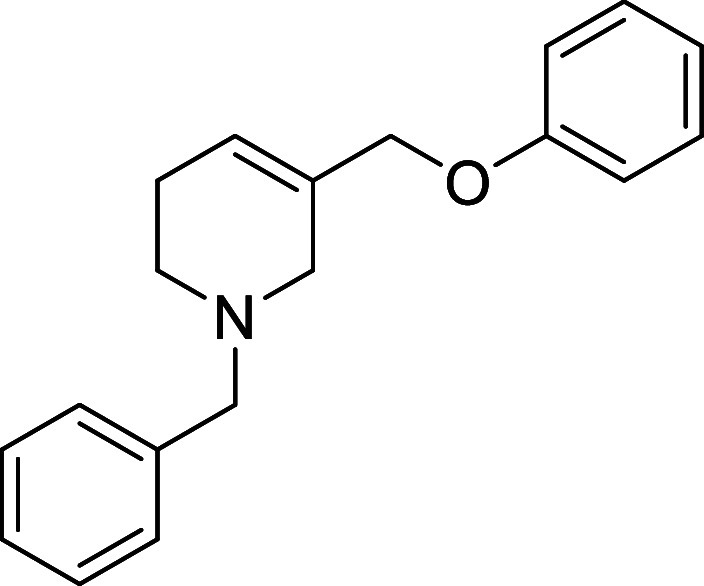



Following the general protocol, **8a** (50.0 mg, 0.25 mmol) and phenol (30.0 mg, 0.30 mmol) were used to obtain **12a**. The residue was purified with SiO_2_ column chromatography and eluted with hexanes/EtOAc (6:1) to obtain 41.9 mg of **12a** (60%) as a yellow oil. *R*_*f*_= 0.10 (hexanes/EtOAc = 3:1); ^1^H NMR (500 MHz, CDCl_3_): δ 7.32 (m, 4H), 7.25 (m, 3H), 6.90 (m, 3H), 5.87 (m, 1H), 4.37 (s, 2H), 3.62 (s, 2H), 3.07 (apparent q, *J* = 2.5 Hz, 2H), 2.56 (t, *J* = 5.8 Hz, 2H), 2.21 (br, 2H). ^13^C{^1^H} NMR (125 MHz, CDCl_3_): δ 158.9 (C), 138.3 (C), 132.7 (C), 129.5 (2CH), 129.3 (2CH), 128.4 (2CH), 127.2 (CH), 124.1 (CH), 120.9 (CH), 114.8 (2CH), 70.8 (CH_2_), 62.8 (CH_2_), 53.6 (CH_2_), 49.4 (CH_2_), 25.9 (CH_2_). HRMS-EI *m*/*z*: [M] ^+^ calcd for C_19_H_21_NO 279.1623; found, 279.1627.

##### 1-Benzyl-5-((4-methoxyphenoxy) methyl)-1,2,3,6-tetrahydropyridine (**12b**)

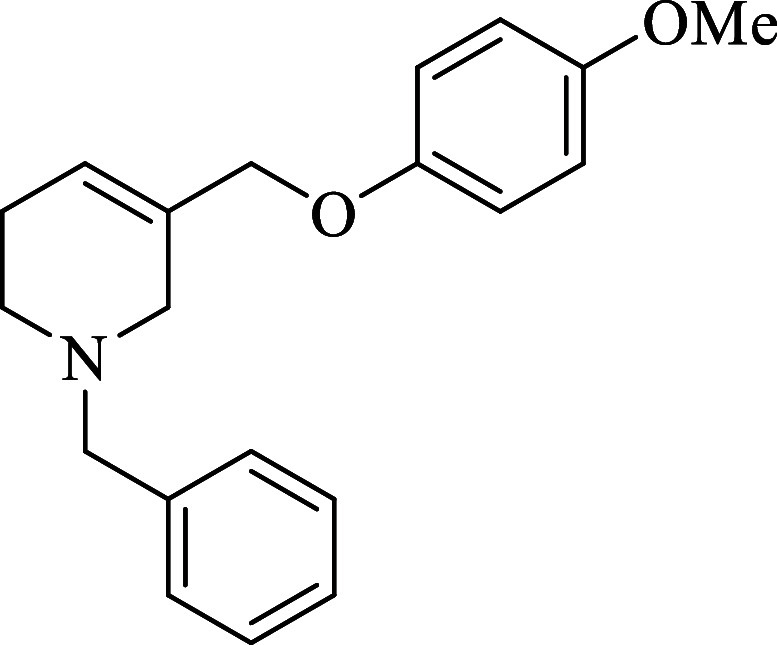



Following the protocol, 48.7 mg of **12b** (63%) was obtained from **8a** (50.0 mg, 0.25 mmol) and 4-methoxyphenol (40.0 mg, 0.30 mmol). Purified with SiO_2_ column chromatography and eluted with hexanes/EtOAc (6:1) to give **12b** as a yellow oil. *R*_*f*_= 0.10 (hexanes/EtOAc = 3:1); ^1^H NMR (500 MHz, CDCl_3_): δ 7.36–7.30 (m, 4H), 7.25 (m, 1H), 6.81 (m, 4H), 5.85 (m, 1H), 4.33 (s, 2H), 3.75 (s, 3H), 3.62 (s, 2H), 3.06 (m, 2H), 2.56 (t, *J* = 5.8 Hz, 2H), 2.20 (br, 2H). ^13^C{^1^H} NMR (125 MHz, CDCl_3_): δ 154.0 (C), 153.1 (C), 138.4 (C), 132.9 (C), 129.3 (2CH), 128.4 (2CH), 127.2 (CH), 124.0 (CH), 115.8 (2CH), 114.7 (2CH), 71.6 (CH_2_), 62.8 (CH_2_), 55.8 (CH_3_), 53.6 (CH_2_), 49.4 (CH_2_), 25.9 (CH_2_). HRMS-FAB *m*/*z*: [M + H]^+^ calcd for C_20_H_24_NO_2_ 310.1807; found, 310.1808.

##### 1-Benzyl-5-((2-iodophenoxy)methyl)-1,2,3,6-tetrahydropyridine (**12c**)

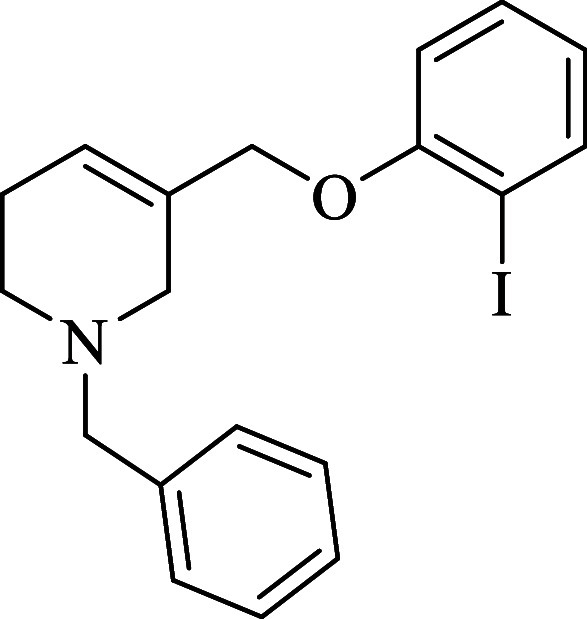



Following the protocol, 81.0 mg of **12c** (80%) was obtained as a yellow oil from **8a** (50.0 mg, 0.25 mmol) and 2-iodophenol (66.0 mg, 0.30 mmol). Purified with SiO_2_ column chromatography and eluted with hexanes/EtOAc (6:1). The NMR data agree with those reported by Mayrargue et al.^17^*R*_*f*_= 0.06 (hexanes/EtOAc = 3:1); ^1^H NMR (500 MHz, CDCl_3_): δ 7.74 (d, *J* = 8.0 Hz, 1H), 7.36 (m, 2H), 7.32 (m, 2H), 7.25 (m, 2H), 6.78 (d, *J* = 8.0 Hz, 1H), 6.69 (t, *J* = 7.8 Hz, 1H), 5.91 (m, 1H), 4.43 (s, 2H), 3.64 (s, 2H), 3.12 (m, 2H), 2.60 (t, *J* = 5.8 Hz, 2H), 2.23 (br, 2H). ^13^C{^1^H} NMR (125 MHz, CDCl_3_): δ 157.3 (C), 139.5 (CH), 138.3 (C), 132.1 (C), 129.5 (CH), 129.4 (2CH), 128.4 (2CH), 127.2 (CH), 123.9 (CH), 122.7 (CH), 112.5 (CH), 86.9 (C), 71.8 (CH_2_), 62.9 (CH_2_), 53.4 (CH_2_), 49.6 (CH_2_), 25.9 (CH_2_). HRMS-EI *m*/*z*: [M]^+^ calcd for C_19_H_20_INO 405.0590; found, 405.0593.

##### 1-Benzyl-5-((2,3-dimethylphenoxy) methyl)-1,2,3,6-tetrahydropyridine (**12d**)

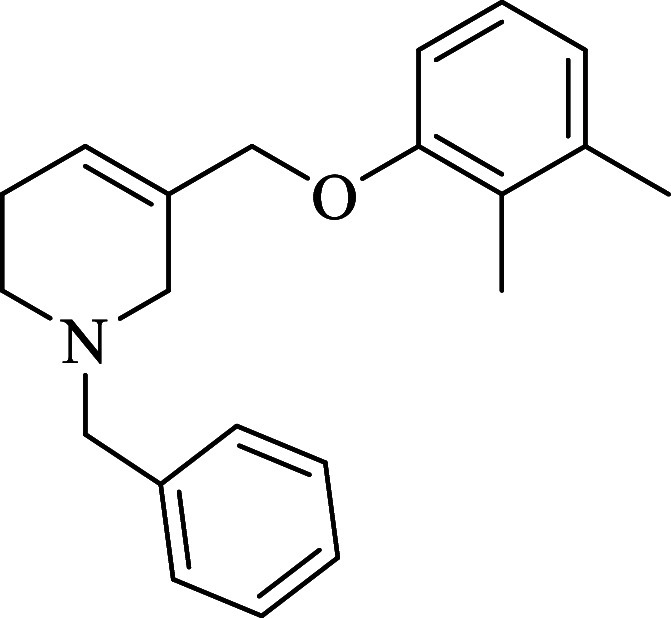



Following the protocol, 0.18 g of **12d** (45%) was obtained as a yellow oil from **8a** (0.26 g, 1.27 mmol) and 2,3-dimethylphenol (0.19 g, 1.52 mmol). Purified with SiO_2_ column chromatography and eluted with hexanes/EtOAc (6:1). *R*_*f*_= 0.12 (hexanes/EtOAc = 3:1); ^1^H NMR (500 MHz, CDCl_3_): δ 7.36–7.31 (m, 4H), 7.25 (m, 1H), 7.01 (apparent t, *J* = 7.8 Hz, 1H), 6.76 (d, *J* = 7.5 Hz, 1H), 6.67 (d, *J* = 8.5 Hz, 1H), 5.86 (m, 1H), 4.35 (s, 2H), 3.62 (s, 2H), 3.07 (s, 2H), 2.58 (t, *J* = 5.8 Hz, 2H), 2.25 (s, 3H), 2.22 (br, 2H), 2.10 (s, 3H). ^13^C{^1^H} NMR (125 MHz, CDCl_3_): δ 156.7 (C), 138.2 (C), 138.0 (C), 133.0 (C), 129.3 (2CH), 128.3 (2CH), 127.2 (CH), 125.8 (CH), 125.4 (C), 123.3 (CH), 122.4 (CH), 109.1 (CH), 71.0 (CH_2_), 62.9 (CH_2_), 53.4 (CH_2_), 49.6 (CH_2_), 25.8 (CH_2_), 20.2 (CH_3_), 11.8 (CH_3_). HRMS (ESI-TOF) *m*/*z*: [M + H]^+^ calcd for C_21_H_26_NO 308.2014; found, 308.2018.

##### 1-Benzyl-5-((3,5-dimethoxyphenoxy)methyl)-1,2,3,6-tetrahydropyridine (**12e**)

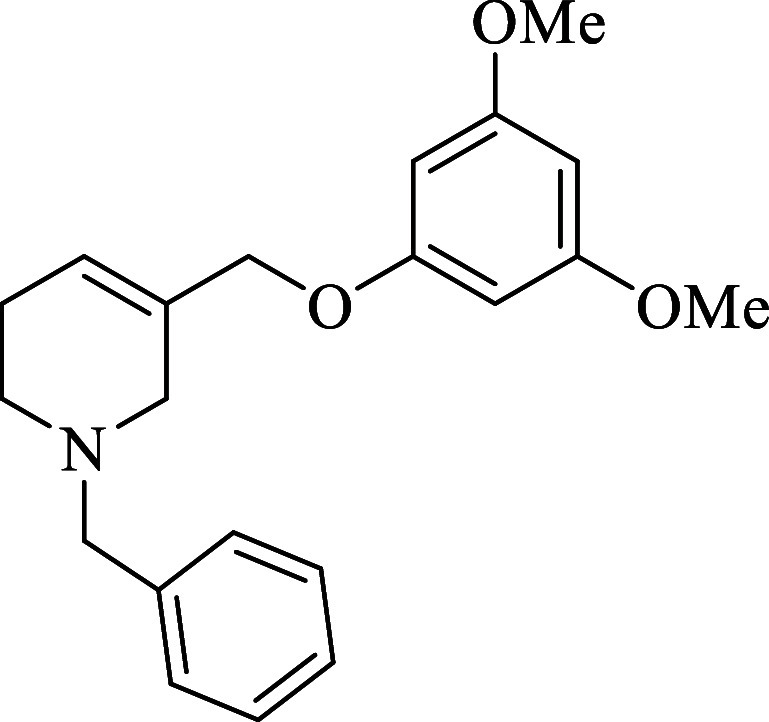



Following the protocol, 0.28 g of **12e** (60%) was obtained as a yellow oil from **8a** (0.28 g, 1.38 mmol) and 0.26 g of 3,5-dimethoxyphenol (1.66 mmol). Purified with SiO_2_ column chromatography and eluted with hexanes/EtOAc (6:1). *R*_*f*_= 0.10 (hexanes/EtOAc = 3:1); ^1^H NMR (500 MHz, CDCl_3_): δ 7.36 (m, 2H), 7.31 (m, 2H), 7.25 (m, 1H), 6.08 (m, 3H), 5.87 (m, 1H), 4.33 (s, 2H), 3.74 (s, 6H), 3.62 (s, 2H), 3.06 (m, 2H), 2.57 (t, *J* = 5.8 Hz, 2H), 2.20 (br, 2H). ^13^C{^1^H} NMR (125 MHz, CDCl_3_): δ 161.5 (2C), 160.8 (C), 138.3 (C), 132.5 (C), 129.3 (2CH), 128.3 (2CH), 127.2 (CH), 124.3 (CH), 93.6 (2CH), 93.1 (CH), 70.9 (CH_2_), 62.8 (CH_2_), 55.4 (2CH_3_), 53.6 (CH_2_), 49.3 (CH_2_), 25.9 (CH_2_). HRMS-FAB *m*/*z*: [M + H]^+^ calcd for C_21_H_26_NO_3_, 340.1913; found, 340.1889.

##### 4-((1-Benzyl-1,2,5,6-tetrahydropyridin-3-yl)methoxy)benzonitrile (**12f**)

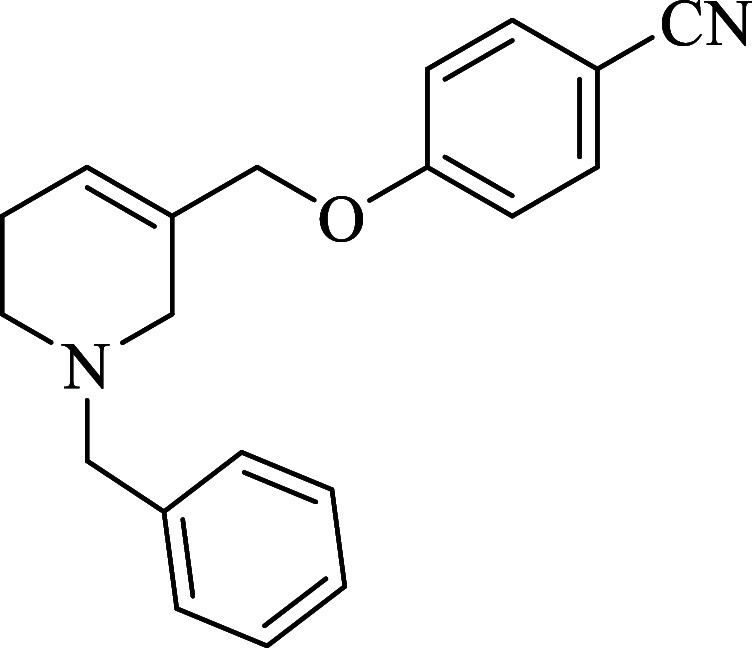



Following the protocol, 0.23 g of **12f** (70%) was obtained as a yellow oil from **8a** (0.22 g, 1.08 mmol) and 4-cyanophenol (0.15 g, 1.30 mmol). Purified with SiO_2_ column chromatography and eluted with hexanes/EtOAc (6:1). *R*_*f*_= 0.06 (hexanes/EtOAc = 3:1); ^1^H NMR (500 MHz, CDCl_3_): δ 7.55 (d, *J* = 8.5 Hz, 2H), 7.33 (m, 4H), 7.25 (m, 1H), 6.92 (d, *J* = 8.5 Hz, 2H), 5.88 (m, 1H), 4.42 (s, 2H), 3.62 (s, 2H), 3.03 (br, 2H), 2.57 (t, *J* = 5.8 Hz, 2H), 2.22 (br, 2H). ^13^C{^1^H} NMR (125 MHz, CDCl_3_): δ 162.1 (C), 138.1 (C), 134.0 (2CH), 131.6 (C), 129.2 (2CH), 128.4 (2CH), 127.2 (CH), 125.2 (CH), 119.3 (C), 115.5 (2CH), 104.1 (C), 71.1 (CH_2_), 62.7 (CH_2_), 53.3 (CH_2_), 49.3 (CH_2_), 25.9 (CH_2_). HRMS-EI *m*/*z*: [M]^+^ calcd for C_20_H_20_N_2_O 304.1576; found, 304.1580.

##### *tert*-Butyl-(2-((1-benzyl-1,2,5,6-tetrahydropyridin-3-yl)methoxy)phenyl)carbamate (**12g**)

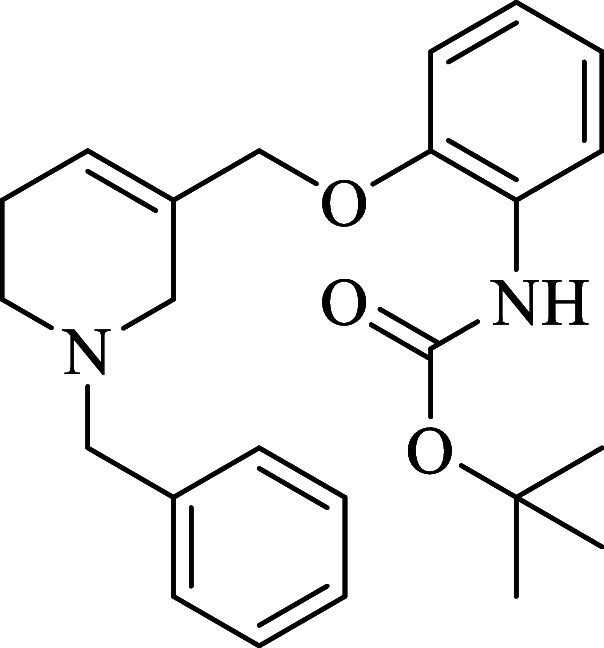



Following the protocol, 0.18 g of **12g** (44%) was obtained as a yellow oil from **8a** (0.21 g, 1.03 mmol) and 0.26 g of *N*-Boc-2-aminophenol (1.24 mmol). Purified with SiO_2_ column chromatography and eluted with hexanes/EtOAc (6:1). *R*_*f*_= 0.06 (hexanes/EtOAc = 3:1); ^1^H NMR (500 MHz, CDCl_3_): δ 8.08 (br, 1H), 7.32 (m, 4H), 7.26 (m, 1H), 7.05 (br, 1H), 6.92 (m, 2H), 6.83 (m, 1H), 5.87 (m, 1H), 4.42 (s, 2H), 3.63 (s, 2H), 3.06 (apparent q, *J* = 2.3 Hz, 2H), 2.57 (t, *J* = 5.8 Hz, 2H), 2.22 (br, 2H), 1.54 (s, 9H). ^13^C{^1^H} NMR (125 MHz, CDCl_3_): δ 152.9 (C), 146.7 (C), 138.0 (C), 132.2 (C), 129.3 (2CH), 128.4 (2CH), 128.3 (CH), 127.3 (CH), 124.7 (CH), 122.3 (CH), 121.3 (CH), 118.2 (CH), 111.4 (CH), 80.4 (C), 71.4 (CH_2_), 62.8 (CH_2_), 53.5 (CH_2_), 49.2 (CH_2_), 28.5 (3CH_3_), 25.8 (CH_2_). HRMS (ESI-TOF) *m*/*z*: [M + H]^+^ calcd for C_24_H_31_N_2_O_3_ 395.2335; found, 395.2331.

##### 4-((1-Benzyl-1,2,5,6-tetrahydropyridin-3-yl)methoxy)benzaldehyde (**12h**)

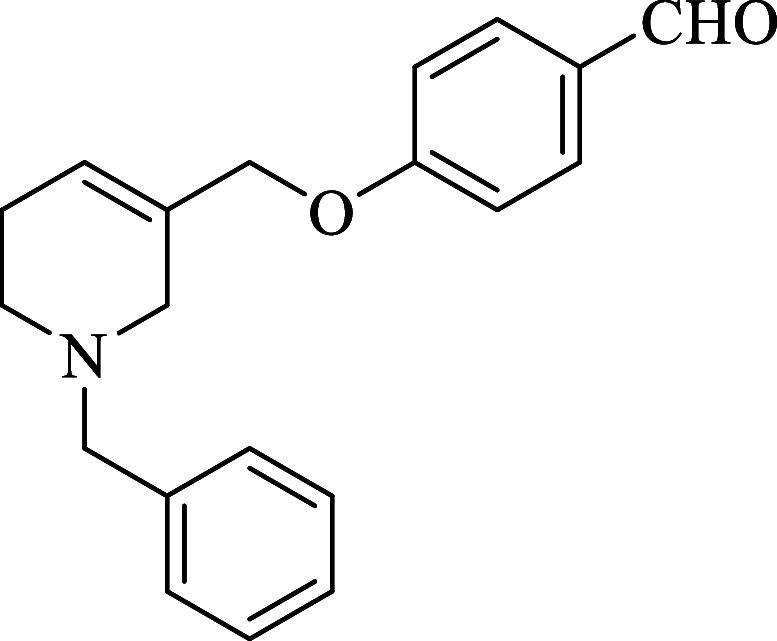



Following the protocol, 43.0 mg of **12h** (56%) was obtained as a yellow oil from **8a** (50.0 mg, 0.25 mmol) and 4-hydroxybenzaldehyde (36.6 mg, 0.30 mmol). Purified with SiO_2_ column chromatography and eluted with hexanes/EtOAc (6:1). *R*_*f*_= 0.04 (hexanes/EtOAc = 3:1); ^1^H NMR (500 MHz, CDCl_3_): δ 9.88 (s, 1H), 7.82 (d, *J* = 8.5 Hz, 2H), 7.32 (m, 4H), 7.25 (m, 1H), 6.99 (d, *J* = 8.5 Hz, 2H), 5.91 (m, 1H), 4.47 (s, 2H), 3.63 (s, 2H), 3.06 (m, 2H), 2.58 (t, *J* = 5.8 Hz, 2H), 2.23 (br, 2H). ^13^C{^1^H} NMR (125 MHz, CDCl_3_): δ 191.0 (C), 164.0 (C), 138.2 (C), 132.1 (2CH), 131.8 (C), 130.1 (C), 129.3 (2CH), 128.4 (2CH), 127.3 (CH), 125.1 (CH), 115.1 (2CH), 71.1 (CH_2_), 62.8 (CH_2_), 53.4 (CH_2_), 49.4 (CH_2_), 25.9 (CH_2_). HRMS (ESI-TOF) *m*/*z*: [M + H] ^+^ calcd for C_20_H_22_NO_2_ 308.1651; found, 308.1648.

##### 1-Benzyl-5-((2-nitrophenoxy) methyl)-1,2,3,6-tetrahydropyridine (**12i**)

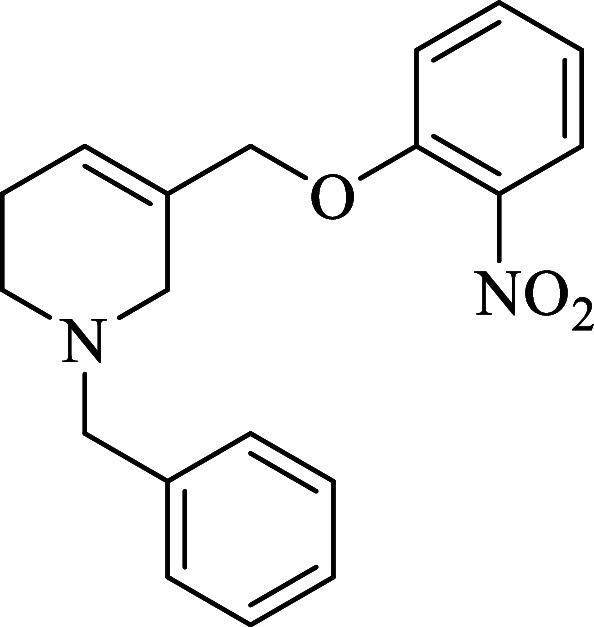



Following the protocol, 0.25 g of **12i** (61%) was obtained as a yellow oil from **8a** (0.26 g, 1.27 mmol) and 2-nitrophenol (0.21 g, 1.52 mmol). Purified with SiO_2_ column chromatography and eluted with hexanes/EtOAc (6:1). *R*_*f*_= 0.06 (hexanes/EtOAc = 3:1); ^1^H NMR (500 MHz, CDCl_3_): δ 7.82 (dd, *J* = 8.3, 1.7 Hz, 1H), 7.49 (td, *J* = 8.3 Hz, 1.5 Hz, 1H), 7.33 (m, 4H), 7.25 (m, 1H), 7.05 (d, *J* = 8.5 Hz, 1H), 7.01 (t, *J* = 7.8 Hz, 1H), 5.92 (m, 1H), 4.52 (s, 2H), 3.63 (s, 2H), 3.07 (m, 2H), 2.56 (t, *J* = 5.8 Hz, 2H), 2.22 (br, 2H). ^13^C{^1^H} NMR (125 MHz, CDCl_3_): δ 152.1 (C), 139.9 (C), 138.1 (C), 134.1 (CH), 131.3 (C), 129.3 (2CH), 128.4 (2CH), 127.2 (CH), 125.7 (CH), 124.9 (CH), 120.4 (CH), 114.8 (CH), 72.1 (CH_2_), 62.7 (CH_2_), 53.2 (CH_2_), 49.3 (CH_2_), 25.8 (CH_2_). HRMS (ESI-TOF) *m*/*z*: [M + H] ^+^ calcd for C_19_H_21_N_2_O_3_ 325.1552; found, 325.1545.

##### 1-Benzyl-5-((2-fluorophenoxy)methyl)-1,2,3,6-tetrahydropyridine (**12j**)

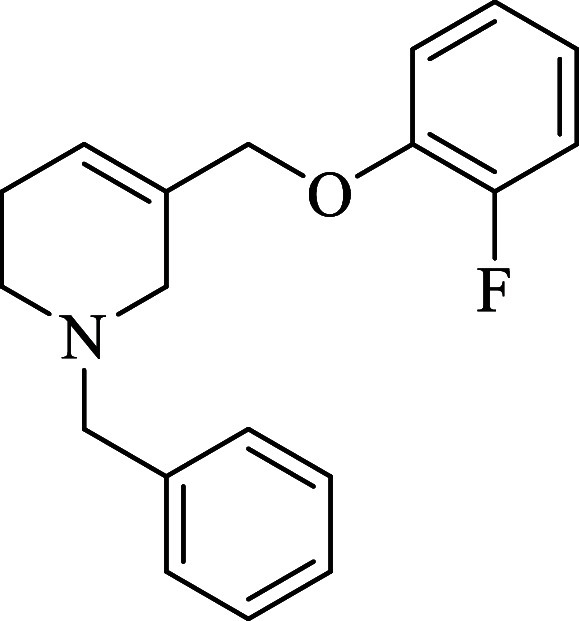



Following the protocol, 86.0 mg of **12j** (59%) was obtained as a yellow oil from **8a** (0.1 g, 0.49 mmol) and 2-fluorophenol (65.9 mg, 0.59 mmol). Purified with SiO_2_ column chromatography and eluted with hexanes/EtOAc (6:1). *R*_*f*_= 0.12 (hexanes/EtOAc = 3:1); ^1^H NMR (500 MHz, CDCl_3_): δ 7.36–7.30 (m, 4H), 7.25 (m, 1H), 7.07–7.00 (m, 2H), 6.94 (td, *J* = 8.3, 1.7 Hz, 1H), 6.88 (m, 1H), 5.88 (m, 1H), 4.45 (s, 2H), 3.62 (s, 2H), 3.08 (m, 2H), 2.56 (t, *J* = 5.8 Hz, 2H), 2.20 (br, 2H). ^13^C{^1^H} NMR (125 MHz, CDCl_3_): δ 153.0 (d, *J* = 244.1 Hz, CF), 146.8 (d, *J* = 10.5 Hz, C), 138.2 (C), 132.3 (C), 129.3 (2CH), 128.3 (2CH), 127.2 (CH), 124.6 (CH), 124.3 (d, *J* = 4.0 Hz, CH), 121.4 (d, *J* = 6.8 Hz, CH), 116.4 (d, *J* = 18.4 Hz, CH), 115.8 (d, *J* = 1.9 Hz, CH), 72.3 (CH_2_), 62.7 (CH_2_), 53.4 (CH_2_), 49.3 (CH_2_), 25.8 (CH_2_). HRMS (ESI-TOF) *m*/*z*: [M + H] ^+^ calcd for C_19_H_21_FNO 298.1607; found, 298.1598.

##### 5-((3,5-Dimethoxyphenoxy)methyl)-1-(4-methoxybenzyl)-1,2,3,6-tetrahydropyridine (**12k**)

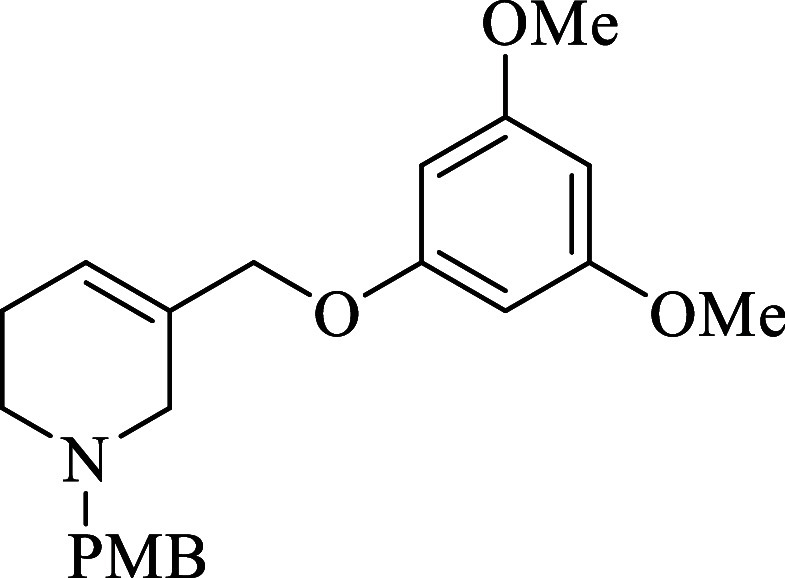



Following the general protocol for the Mitsunobu reaction, 0.71 g of **12k** (50%) was obtained as a yellow oil from **8b** (0.9 g, 3.86 mmol) and 3,5-dimethoxyphenol (0.71 g, 4.63 mmol). Purified with SiO_2_ column chromatography and eluted with hexanes/EtOAc (3:1). *R*_*f*_= 0.04 (hexanes/EtOAc = 3:2); ^1^H NMR (500 MHz, CDCl_3_): δ 7.25 (d, *J* = 8.5 Hz, 2H), 6.85 (d, *J* = 8.5 Hz, 2H), 6.08 (br, 3H), 5.86 (m, 1H), 4.33 (d, *J* = 2.0 Hz, 2H), 3.79 (s, 3H), 3.74 (s, 6H), 3.55 (s, 2H), 3.03 (m, 2H), 2.54 (t, *J* = 5.8 Hz, 2H), 2.20 (br, 2H). ^13^C{^1^H} NMR (125 MHz, CDCl_3_): δ 161.6 (2C), 160.9 (C), 158.9 (C), 132.6 (C), 130.5 (2CH), 130.3 (C), 124.4 (CH), 113.7 (2CH), 93.7 (2CH), 93.2 (CH), 71.0 (CH_2_), 62.1 (CH_2_), 55.4 (2CH_3_), 55.4 (CH_3_), 53.5 (CH_2_), 49.2 (CH_2_), 25.9 (CH_2_). HRMS-FAB *m*/*z*: [M + H]^+^ calcd for C_22_H_28_NO_4_, 370.2018; found, 370.2010.

#### Synthesis of Precursor 12l

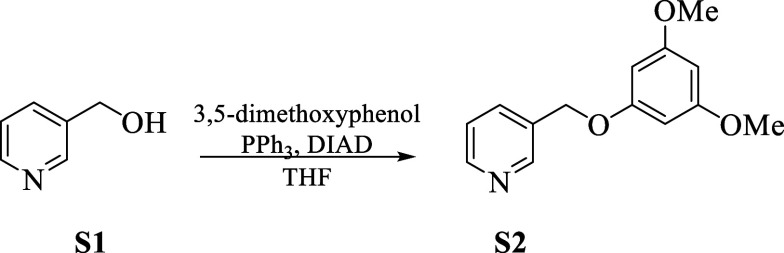



##### 3-((3,5-Dimethoxyphenoxy)methyl)pyridine (**S2**)

To a solution of the 3-pyridinemethanol **S1** (5.0 g, 45.82 mmol), triphenylphosphine (14.42 g, 54.98 mmol), and the 3,5-dimethoxyphenol (8.48 g, 54.98 mmol) in THF anhydrous (92.0 mL) at 0 °C, under argon atmosphere, was added dropwise DIAD (10.83 mL, 54.98 mmol). The reaction mixture was stirred for 15 min at 0 °C, consequently, the reaction was stirred at room temperature, and monitored by TLC. Once the raw material was consumed, the solvent was removed under reduced pressure and the residue was treated with 1 N NaOH aqueous solution. The resulting mixture was extracted with EtOAc (3 × 20 mL), and the organic phase was dried with Na_2_SO_4_ and concentrated under reduced pressure. The residue was purified by column chromatography with SiO_2_ and eluted with EtOAc/MeOH (4:1) to give 9.90 g (88%) of **S2** as a yellow oil. *R*_*f*_ = 0.5 (hexanes/EtOAc = 4:1); ^1^H NMR (500 MHz, CDCl_3_): δ 8.67 (d, *J* = 2.5 Hz, 1H), 8.58 (dd, *J* = 5.0, 1.5 Hz, 1H), 7.76 (d, *J* = 7.5 Hz, 1H), 7.32 (dd, *J* = 7.8, 4.8 Hz, 1H), 6.16 (d, *J* = 2.0 Hz, 1H), 6.12 (m, 1H), 5.03 (s, 2H), 3.77 (s, 6H). ^13^C{^1^H} NMR (125 MHz, CDCl_3_): δ 161.7 (2C), 160.4 (C), 149.6 (CH), 149.2 (CH), 135.4 (CH), 132.5 (C), 123.6 (CH), 93.8 (2CH), 93.6 (CH), 67.7 (CH_2_), 55.5 (2CH_3_). HRMS-FAB *m*/*z*: [M + H]^+^ calcd for C_14_H_16_NO_3_, 246.1130; found, 246.1127.
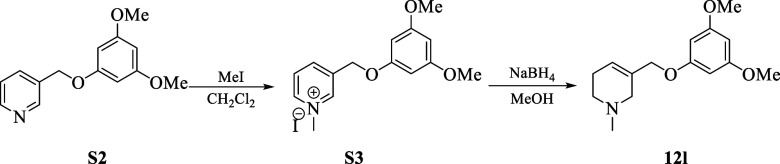


##### 5-((3,5-Dimethoxyphenoxy)methyl)-1-methyl-1,2,3,6-tetrahydropyridine(12l)

The compound **12l** was obtained using the procedure reported by Mayrargue et al.^17^ To a stirred solution of pyridine **S2** (1.95 g, 7.95 mmol) in dry dichloromethane (20 mL) was added dropwise iodomethane (0.59 mL, 9.54 mmol). The reaction mixture was stirred overnight at room temperature. Then, the white solid formed was filtered and washed with dichloromethane to afford the pyridinium salt **S3** which was used without further purification in the next step. To a suspension of salt **S3** (1.60 g, 4.29 mmol) in MeOH reagent grade (30 mL) cooled to 0 °C was added portion-wise of sodium borohydride (0.40 g, 10.73 mmol) over 20 min. Once the addition was complete, the mixture was allowed to stir overnight at room temperature. The methanol was removed under reduced pressure, the residue was treated with an aqueous solution of NaOH (15 mL, 1 N), and the aqueous phase was extracted with dichloromethane (3 × 15 mL). The combined organic phases were dried with Na_2_SO_4_, and the solvent was removed under reduced pressure. The residue was purified by column chromatography with SiO_2_ and eluted with EtOAc/Hexane (1:1) to give 1.25 g (60%) of **12l** as a yellow oil. *R*_*f*_ = 0.06 (hexanes/EtOAc = 1:1); ^1^H NMR (500 MHz, CDCl_3_): δ 6.09 (s, 2H), 6.07 (s, 1H), 5.85 (m, 1H), 4.36 (s, 2H), 3.75 (s, 6H), 2.99 (br, 2H), 2.51 (td, *J* = 5.5, 1.7 Hz, 2H), 2.38 (s, 3H), 2.25 (br, 2H). ^13^C{^1^H} NMR (125 MHz, CDCl_3_): δ 161.5 (2C), 160.8 (C), 132.5 (C), 123.8 (CH), 93.6 (2CH), 93.1 (CH), 70.9 (CH_2_), 55.4 (2CH_3_), 55.3 (CH_2_), 51.8 (CH_2_), 46.0 (CH_3_), 26.1 (CH_2_). HRMS (ESI-TOF) *m*/*z*: [M + H] ^+^ calcd for C_15_H_22_NO_3_ 264.1599; found, 264.1600.

### Synthesis of 4-(2-hydroxyphenyl)-3-methylenepiperidines via a Claisen Rearrangement

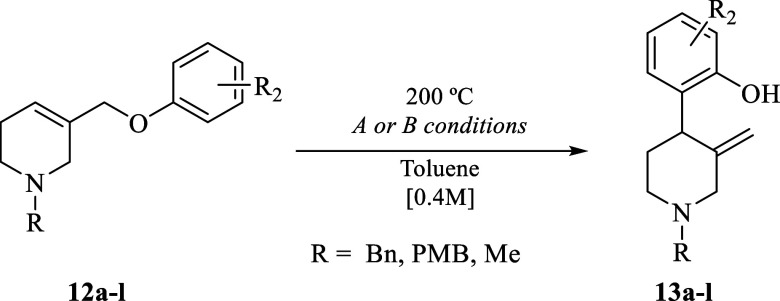



#### Bath Oil Conditions (A)

To scale up the reaction, a 0.4 M solution of the corresponding allyl aryl ether (0.10–2.94 mmol) in reagent grade toluene was stirred at 200 °C in an oil bath. Once the raw material was consumed, the reaction was cooled to room temperature and concentrated under reduced pressure for purification by column chromatography.

#### Microwave Conditions (B)

A 0.4 M solution of the corresponding allyl aryl ether (0.10–0.16 mmol) in reagent grade toluene was placed in a microwave tube. The reaction mixture was heated to 200 °C for 40 min and concentrated under reduced pressure for purification by column chromatography.

##### 6-(1-Benzyl-3-methylenepiperidin-4-yl)benzo[*d*][1,3]dioxol-5-ol (**10**)

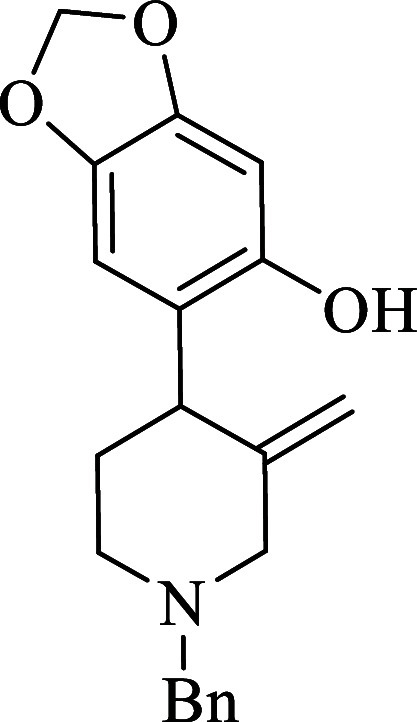



Following the microwave (mw) protocol for the Claisen rearrangement, 35.7 mg of **10** (92%) was obtained as a brown oil from **9** (40.0 mg, 0.12 mmol). Purified with SiO_2_ column chromatography and eluted with hexanes/EtOAc (6:1) as the solvent system. *R*_*f*_= 0.06 (hexanes/EtOAc = 3:1); ^1^H NMR (500 MHz, CDCl_3_): δ 7.34 (m, 4H), 7.28 (m, 1H), 6.61 (s, 1H), 6.41 (s, 1H), 5.88 (m, 2H), 4.86 (s, 1H), 4.46 (s, 1H), 3.65 (d, *J* = 13.0 Hz, 1H), 3.59 (d, *J* = 12.5 Hz, 1H), 3.48 (dd, *J* = 12.0, 2.0 Hz, 1H), 3.40 (m, 1H), 3.09 (d, *J* = 11.5 Hz, 1H), 2.82 (d, *J* = 12.5 Hz, 1H), 2.30 (td, *J* = 11.8, 3.0 Hz, 1H), 2.04 (qd, *J* = 12.3, 4.1 Hz, 1H), 1.81 (m, 1H). ^13^C{^1^H} NMR (125 MHz, CDCl_3_): δ 148.6 (C), 146.5 (C), 145.6 (C), 141.3 (C), 137.2 (C), 129.6 (2CH), 128.4 (2CH), 127.4 (CH), 120.2 (C), 110.5 (CH_2_), 108.4 (CH), 101.0 (CH_2_), 99.1 (CH), 62.8 (CH_2_), 60.6 (CH_2_), 53.6 (CH_2_), 42.3 (CH), 31.1 (CH_2_). HRMS-EI *m*/*z*: [M]^+^ calcd for C_20_H_21_NO_3_ 323.1521; found, 323.1516.

##### 2-(1-Benzyl-3-methylenepiperidin-4-yl)phenol (**13a**)

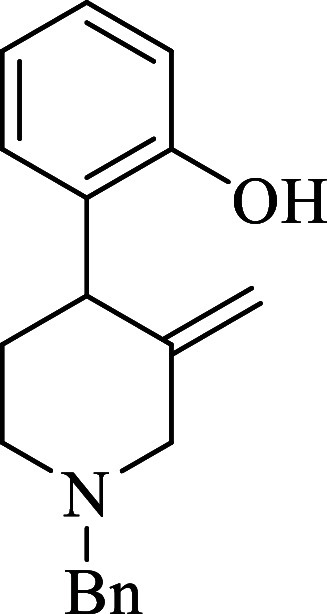



Following the *mw* protocol, **12a** (40.0 mg, 0.14 mmol) was used to obtain **13a**. The residue was purified with SiO_2_ column chromatography and eluted with hexanes/EtOAc (6:1) to obtain 13.6 mg of **13a** (35%) as a yellow oil. *R*_*f*_= 0.10 (hexanes/EtOAc = 3:1); ^1^H NMR (500 MHz, CDCl_3_): δ 7.38–7.32 (m, 4H), 7.29 (m, 1H), 7.15 (m, 2H), 6.91 (apparent td, *J* = 7.5, 1.5 Hz, 1H), 6.85 (dd, *J* = 8.0, 1.5 Hz, 1H), 4.89 (s, 1H), 4.46 (s, 1H), 3.68 (d, *J* = 13.0 Hz, 1H), 3.61 (d, *J* = 13.0 Hz, 1H), 3.49 (m, 2H), 3.10 (apparent d, *J* = 11.0 Hz, 1H), 2.86 (d, *J* = 12.0 Hz, 1H), 2.33 (m, 1H), 2.15 (qd, *J* = 12.0, 4.4 Hz, 1H), 1.88 (m, 1H). ^13^C{^1^H} NMR (125 MHz, CDCl_3_): δ 154.0 (C), 145.3 (C), 137.3 (C), 129.6 (2CH), 129.4 (CH), 128.5 (2CH), 128.1 (C), 128.1 (CH), 127.5 (CH), 120.9 (CH), 116.8 (CH), 110.8 (CH_2_), 62.7 (CH_2_), 60.4 (CH_2_), 53.3 (CH_2_), 43.1(CH), 30.7 (CH_2_). HRMS-EI *m*/*z*: [M]^+^ calcd for C_19_H_21_NO 279.1623; found, 279.1616.

##### 2-(1-Benzyl-3-methylenepiperidin-4-yl)-4-methoxyphenol (**13b**)

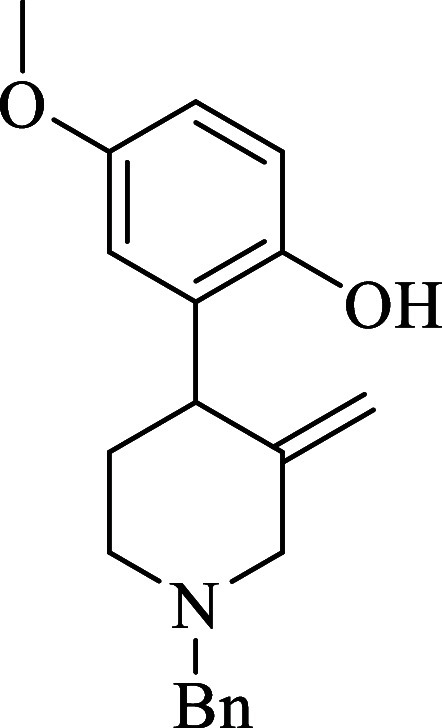



Following the *mw* protocol, 39.6 mg of **13b** (80%) was obtained from **12b** (50.0 mg, 0.16 mmol). Purified with SiO_2_ column chromatography and eluted with hexanes/EtOAc (6:1) to give **13b** as a yellow oil. *R*_*f*_= 0.10 (hexanes/EtOAc = 3:1); ^1^H NMR (500 MHz, CDCl_3_): δ 7.34 (m, 4H), 7.28 (m, 1H), 6.76 (d, *J* = 9.0 Hz, 1H), 6.71–6.67 (m, 2H), 4.86 (s, 1H), 4.43 (s, 1H), 3.74 (s, 3H), 3.66 (d, *J* = 13.0 Hz, 1H), 3.59 (d, *J* = 13.0 Hz, 1H), 3.48 (m, 2H), 3.09 (apparent d, *J* = 12.5 Hz, 1H), 2.84 (d, *J* = 13.0 Hz, 1H), 2.31 (td, *J* = 12.0, 3.0 Hz, 1H), 2.11 (qd, *J* = 12.3, 4.0 Hz, 1H), 1.85 (m, 1H). ^13^C{^1^H} NMR (125 MHz, CDCl_3_): δ 153.9 (C), 147.9 (C), 145.6 (C), 137.7 (C), 129.5 (C), 129.4 (2CH), 128.4 (2CH), 127.4 (CH), 117.4 (CH), 114.9 (CH), 112.8 (CH), 110.4 (CH_2_), 62.9 (CH_2_), 60.7 (CH_2_), 55.8 (CH_3_), 53.5 (CH_2_), 43.3 (CH), 31.0 (CH_2_). HRMS-EI *m*/*z*: [M]^+^ calcd for C_20_H_23_NO_2_ 309.1729; found, 309.1723.

##### 6-(1-Menzyl-3-methylenepiperidin-4-yl)-2,3-dimethylphenol (**13d**)

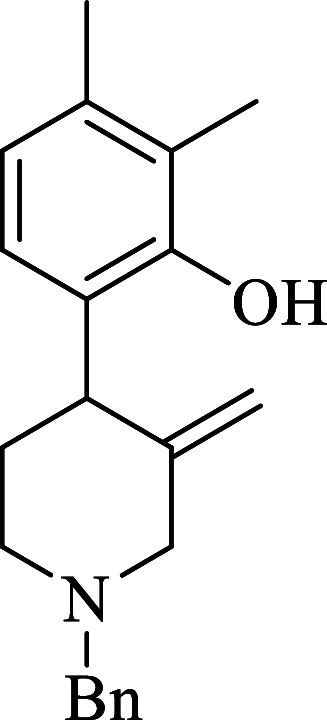



Following the *mw* protocol, 53.5 mg of **13d** (60%) was obtained as a yellow oil from **12d** (90.0 mg, 0.29 mmol). Purified with SiO_2_ column chromatography and eluted with hexanes/EtOAc (6:1). *R*_*f*_= 0.12 (hexanes/EtOAc = 3:1); ^1^H NMR (500 MHz, CDCl_3_): δ 7.37–7.32 (m, 4H), 7.28 (d, *J* = 6.5 Hz, 1H), 6.87 (d, *J* = 7.5 Hz, 1H), 6.75 (d, *J* = 7.5 Hz, 1H), 4.90 (d, *J* = 2.0 Hz, 1H), 4.50 (s, 1H), 3.64 (d, *J* = 13.0 Hz, 1H), 3.59 (d, *J* = 13.0 Hz, 1H), 3.48 (dd, *J* = 12.0, 2.0 Hz, 1H), 3.37 (dd, *J* = 12.0, 5.0 Hz, 1H), 3.08 (d, *J* = 11.5 Hz, 1H), 2.82 (d, *J* = 12.0 Hz, 1H), 2.31 (m, 1H), 2.26 (s, 3H), 2.18 (s, 3H), 2.13 (m, 1H), 1.86 (m, 1H). ^13^C{^1^H} NMR (125 MHz, CDCl_3_): δ 151.9 (C), 145.5 (C), 137.8 (C), 136.6 (C), 129.4 (2CH), 128.4 (2CH), 127.3 (CH), 125.8 (CH), 125.1 (C), 123.9 (C), 122.2 (CH), 110.7 (CH_2_), 62.8 (CH_2_), 60.5 (CH_2_), 53.6 (CH_2_), 43.9 (CH), 30.9 (CH_2_), 20.2 (CH_3_), 12.0 (CH_3_). HRMS-EI *m*/*z*: [M]^+^ calcd for C_21_H_25_NO 307.1936; found, 307.1935.

##### 2-(1-Benzyl-3-methylenepiperidin-4-yl)-3,5-dimethoxyphenol (**13e**)

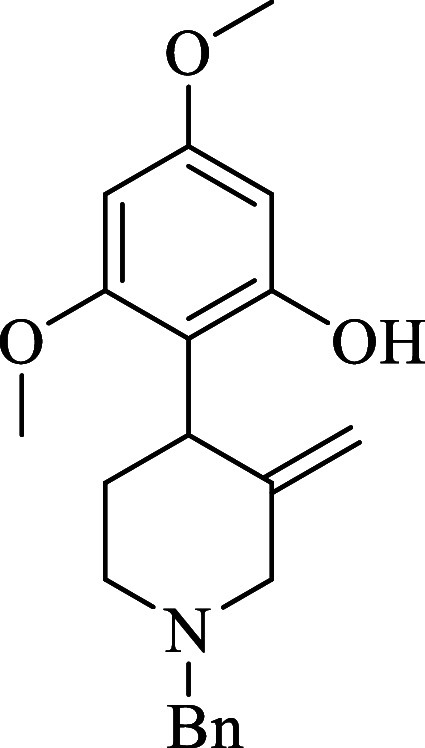



Following the *mw* protocol, 45.0 mg of **13e** (88%) was obtained as an orange solid (mp = 171–173 °C) from **12e** (0.05 g, 0.15 mmol). Purified with SiO_2_ column chromatography and eluted with hexanes/EtOAc (6:1). *R*_*f*_= 0.06 (hexanes/EtOAc = 3:1); ^1^H NMR (500 MHz, CDCl_3_): δ 7.37–7.32 (m, 4H), 7.28 (d, *J* = 6.5 Hz, 1H), 6.13 (d, *J* = 2.0 Hz, 1H), 6.08 (d, *J* = 2.5 Hz, 1H), 4.90 (s, 1H), 4.73 (s, 1H), 4.09 (m, 1H), 3.77 (s, 3H), 3.75 (s, 3H), 3.66 (d, *J* = 13.0 Hz, 1H), 3.58 (d, *J* = 13.0 Hz, 1H), 3.53 (d, *J* = 14.0 Hz, 1H), 3.00 (m, 1H), 2.86 (d, *J* = 13.0 Hz, 1H), 2.27 (td, *J* = 11.5, 3.5 Hz, 1H), 2.07 (m, 1H), 1.77 (m, 1H). ^13^C{^1^H} NMR (125 MHz, CDCl_3_): δ 159.9 (C), 158.7 (C), 156.4 (C), 144.8 (C), 137.3 (C), 129.5 (2CH), 128.5 (2CH), 127.5 (CH), 110.5 (CH_2_), 109.5 (C), 95.2 (CH), 91.6 (CH), 62.6 (CH_2_), 59.7 (CH_2_), 55.9 (CH_3_), 55.4 (CH_3_), 52.6 (CH_2_), 35.1 (CH), 29.5 (CH_2_). HRMS-EI *m*/*z*: [M]^+^ calcd for C_21_H_25_NO_3_ 339.1834; found, 339.1829.

##### 2-Amino-6-(1-benzyl-3-Methylenepiperidin-4-yl)phenol (**13g**)

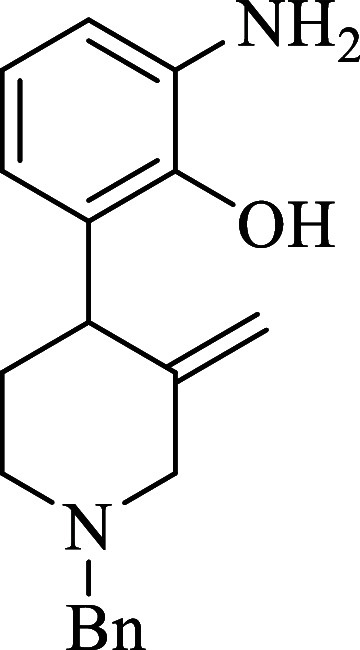



Following the *mw* protocol, **13g** and **13gg** were obtained from **12g** (34.0 mg, 0.09 mmol). Purified with SiO_2_ column chromatography and eluted with hexanes/EtOAc (6:1). **13g** as a yellow oil (12.5 mg, 47%). *R*_*f*_= 0.06 (hexanes/EtOAc = 3:1); ^1^H NMR (500 MHz, CDCl_3_): δ 7.37–7.32 (m, 4H), 7.29 (m, 1H), 6.74 (t, *J* = 7.5 Hz, 1H), 6.68 (dd, *J* = 7.8, 1.5 Hz, 1H), 6.55 (dd, *J* = 7.3, 1.8 Hz, 1H), 4.91 (d, *J* = 2.0 Hz, 1H), 4.60 (d, *J* = 2.0 Hz, 1H), 3.67 (d, *J* = 13.0 Hz, 1H), 3.61 (d, *J* = 13.0 Hz, 1H), 3.52 (d, *J* = 13.0 Hz, 1H), 3.39 (dd, *J* = 11.3, 5.8 Hz, 1H), 3.09 (m, 1H), 2.86 (d, *J* = 12.5 Hz, 1H), 2.32 (td, *J* = 11.6, 3.3 Hz, 1H), 2.16 (qd, *J* = 11.8, 4.5 Hz, 1H), 1.90 (m, 1H). ^13^C{^1^H} NMR (125 MHz, CDCl_3_): δ 145.0 (C), 142.3 (C), 137.2 (C), 135.9 (C), 129.5 (2CH), 128.5 (2CH), 128.3 (C), 127.5 (CH), 121.0 (CH), 119.7 (CH), 115.3 (CH), 111.0 (CH_2_), 62.6 (CH_2_), 60.0 (CH_2_), 52.9 (CH_2_), 44.3 (CH), 30.3 (CH_2_). HRMS-EI *m*/*z*: [M]^+^ calcd for C_19_H_22_N_2_O, 294.1732; found, 294.1732.

##### 7-(1-Benzyl-3-methylenepiperidin-4-yl)benzo[*d*]oxazol-2(3*H*)-one (**13gg**)

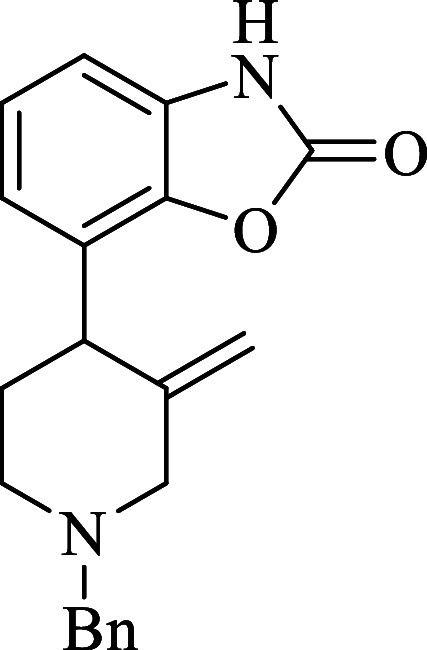



As a yellow oil (9.2 mg, 32%). *R*_*f*_= 0.10 (hexanes/EtOAc = 3:1); ^1^H NMR (500 MHz, CDCl_3_): δ 7.39–7.32 (m, 4H), 7.28 (m, 1H), 7.11 (t, *J* = 7.8 Hz, 1H), 7.00 (d, *J* = 8.0 Hz, 1H), 6.93 (dd, *J* = 7.5, 1.0 Hz, 1H), 4.83 (s, 1H), 4.21 (s, 1H), 3.69–3.61 (m, 3H), 3.47 (dd, *J* = 12.3, 1.8 Hz, 1H), 3.10 (d, *J* = 11.0 Hz, 1H), 2.88 (d, *J* = 11.5 Hz, 1H), 2.38 (td, *J* = 11.5, 2.7 Hz, 1H), 2.20 (qd, *J* = 12.3, 4.0 Hz, 1H), 1.87 (m, 1H). ^13^C{^1^H} NMR (125 MHz, CDCl_3_): δ 155.7 (C), 145.1 (C), 142.4 (C), 137.6 (C), 129.6 (2CH), 129.5 (C), 128.4 (2CH), 127.4 (CH), 125.3 (C), 124.1 (CH), 122.5 (CH), 110.7 (CH_2_), 108.2 (CH), 62.7 (CH_2_), 60.8 (CH_2_), 53.5 (CH_2_), 41.9 (CH), 31.3 (CH_2_). HRMS-EI *m*/*z*: [M]^+^ calcd for C_20_H_20_N_2_O_2_, 320.1525; found, 320.1523.

##### 2-(1-Benzyl-3-methylenepiperidin-4-yl)-6-fluorophenol (**13j**)

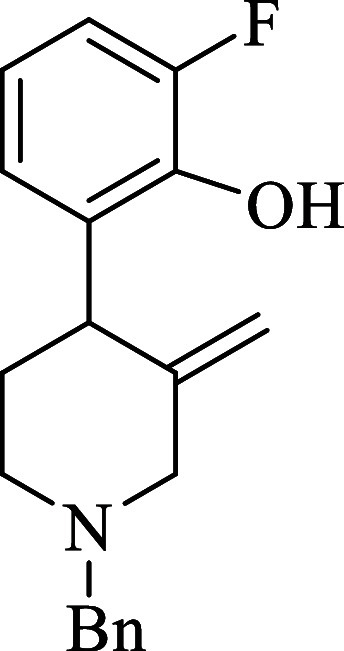



Following the *mw* protocol, 13.4 mg of **13j** (25%) was obtained as a yellow oil from **12j** (53.0 mg, 0.18 mmol). Purified with SiO_2_ column chromatography and eluted with hexanes/EtOAc (10:1). *R*_*f*_= 0.11 (hexanes/EtOAc = 6:1); ^1^H NMR (500 MHz, CDCl_3_): δ 7.37–7.32 (m, 4H), 7.28 (m, 1H), 6.97 (m, 2H), 6.82 (m, 1H), 4.82 (d, *J* = 2.0 Hz, 1H), 4.29 (d, *J* = 2.0 Hz, 1H), 3.65–3,60 (m, 3H), 3.47 (dd, *J* = 12.0, 1.5 Hz, 1H), 3.08 (m, 1H), 2.87 (dd, *J* = 12.8, 1.3 Hz, 1H), 2.33 (td, *J* = 12.0, 3.0 Hz, 1H), 2.11 (qd, *J* = 12.1, 4.1 Hz, 1H), 1.83 (m, 1H). ^13^C{^1^H} NMR (125 MHz, CDCl_3_): δ 151.6 (d, *J* = 235.8 Hz, C), 146.0 (C), 142.0 (d, *J* = 13.6 Hz, C), 137.8 (C), 131.0 (C), 129.5 (2CH), 128.4 (2CH), 127.3 (CH), 124.5 (d, *J* = 3.1 Hz, CH), 120.0 (d, *J* = 7.3 Hz, CH), 113.6 (d, *J* = 18.3 Hz, CH), 110.1 (CH_2_), 62.8 (CH_2_), 60.8 (CH_2_), 53.4 (CH_2_), 41.6 (CH), 31.2 (CH_2_). HRMS-FAB *m*/*z*: [M + H]^+^ calcd for C_19_H_21_FNO, 298.1607; found, 298.1613.

##### 3,5-Dimethoxy-2-(1-(4-methoxybenzyl)-3-methylenepiperidin-4-yl)phenol (**13k**)

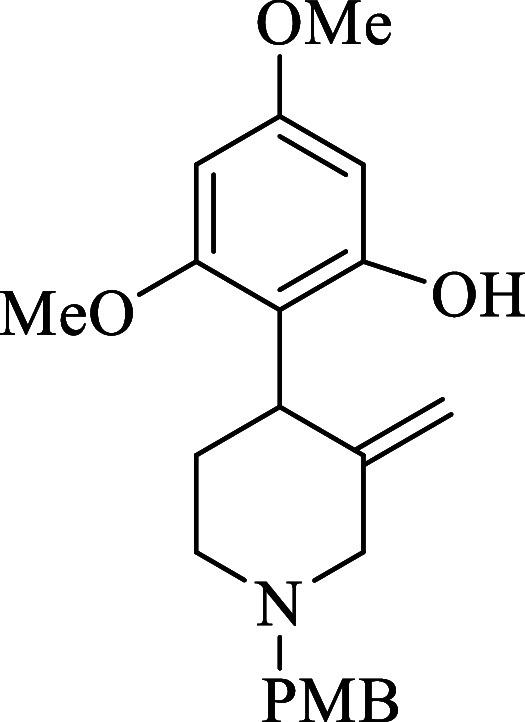



Using the bath oil conditions, 0.37 g of **13k** (65%) was obtained as a yellow oil from **12k** (0.52 g, 1.53 mmol). Purified with SiO_2_ column chromatography and eluted with hexanes/EtOAc (6:1). *R*_*f*_= 0.06 (hexanes/EtOAc = 3:1); ^1^H NMR (500 MHz, CDCl_3_): δ 7.27 (d, *J* = 9.0 Hz, 2H), 6.87 (d, *J* = 8.0 Hz, 2H), 6.12 (d, *J* = 2.5 Hz, 1H), 6.07 (d, *J* = 2.0 Hz, 1H), 4.88 (d, *J* = 2.0 Hz, 1H), 4.73 (d, *J* = 2.0 Hz, 1H), 4.10 (m, 1H), 3.81 (s, 3H), 3.76 (s, 3H), 3.75 (s, 3H), 3.60 (d, *J* = 12.5 Hz, 1H), 3.52 (m, 2H), 2.99 (m, 1H), 2.85 (d, *J* = 14.0 Hz, 1H), 2.25 (td, *J* = 11.0, 4.0 Hz, 1H), 2.06 (m, 1H), 1.78 (m, 1H). ^13^C{^1^H} NMR (125 MHz, CDCl_3_): δ 159.9 (C), 159.1 (C), 158.7 (C), 156.5 (C), 144.9 (C), 130.7 (2CH), 129.2 (C), 113.9 (2CH), 110.5 (CH_2_), 109.7 (C), 95.2 (CH), 91.5 (CH), 61.8 (CH_2_), 59.3 (CH_2_), 55.9 (CH_3_), 55.4 (CH_3_), 55.3 (CH_3_), 52.1 (CH_2_), 35.0 (CH), 29.3 (CH_2_). HRMS-FAB *m*/*z*: [M + H]^+^ calcd for C_22_H_28_NO_4_, 370.2018; found, 370.2022.

##### 3,5-Dimethoxy-2-(1-methyl-3-methylenepiperidin-4-yl)phenol (**13l**)

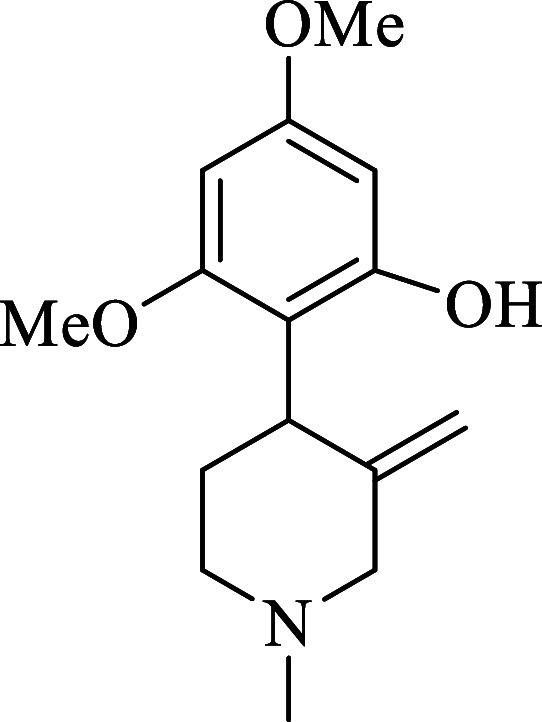



Following the bath oil conditions, 19.7 mg of **13l** (50%) was obtained from **12l** (40.0 mg, 0.15 mmol). The residue was purified by column chromatography with SiO_2_ and eluted with EtOAc/MeOH (10:0.5) to give **13l** as a white solid, mp = 109–202 °C.*R*_*f*_= 0.08 (EtOAc/MeOH = 20:1); ^1^H NMR (500 MHz, CDCl_3_): δ6.10 (s, 1H), 6.06 (s, 1H), 4.91 (s, 1H), 4.64 (s, 1H), 4.0 (m, 1H), 3.75 (s, 3H), 3.73 (s, 3H), 3.46 (d, *J* = 13.0 Hz, 1H), 3.01 (d, *J* = 10.0 Hz, 1H), 2.83 (d, *J* = 13.0 Hz, 1H), 2.39 (s, 3H), 2.27 (m, 2H), 1.72 (m, 1H). ^13^C{^1^H} NMR (125 MHz, CDCl_3_): δ 159.9 (C), 158.8 (C), 156.6 (C), 143.1 (C), 110.8 (CH_2_), 108.6 (C), 95.0 (CH), 91.2 (CH), 62.1 (CH_2_), 55.9 (CH_3_), 55.5 (CH_2_), 55.3 (CH_3_), 45.4 (CH_3_), 35.0 (CH), 28.8 (CH_2_). HRMS-EI *m*/*z*: [M]^+^ calcd for C_15_H_21_NO_3_ 263.1521; found, 263.1516.

##### Synthetic Application; Flavonoid Alkaloids


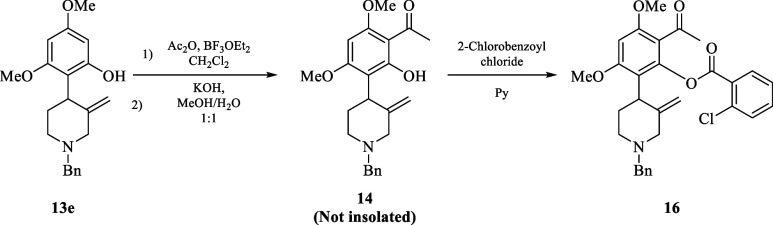


##### 2-Acetyl-6-(1–benzyl–3–methylenepiperidin–4-yl)-3,5-dimethoxyphenyl-2-chlorobenzoate (**16**)

To a solution of BF_3_ etherate (5.32 mmol) in dry CH_2_Cl_2_ (5 mL) at 0 °C, under an argon atmosphere, distilled acetic anhydride (5.32 mmol) was added. The reaction mixture was stirred for 5 min, followed by the addition of a solution of **13e** (0.38 mmol) in CH_2_Cl_2_ (6 mL). The resulting mixture was stirred at room temperature for 16 h, then quenched with a saturated aqueous solution of Na_2_CO_3_ until a yellow color appeared. The aqueous phase was extracted with CH_2_Cl_2_ (3 × 10 mL). The organic layer was separated, dried with Na_2_SO_4,_ and concentrated under reduced pressure. The crude reaction product was dissolved in 4 mL of a mixture of MeOH:H_2_O (1:1), cooled in an ice bath and treated with KOH (powder, 0.95 mmol). After addition, the reaction mixture was allowed to warm to room temperature and stirred for 2 h. The MeOH was removed under reduced pressure and H_2_O (3 mL) was added. The aqueous phase was extracted with CH_2_Cl_2_ (3 × 15 mL), and the organic layer was separated, treated with Na_2_SO_4,_ and evaporated under reduced pressure to afford **14** as a yellow oil. Intermediate **14** (0.34 mmol), without purification, was dissolved in pyridine (3.5 mL) at 0 °C, and under argon atmosphere, 2-chlorobenzoyl chloride (1.02 mmol) was added dropwise. Once the addition was completed, the mixture was stirred at room temperature overnight. The reaction mixture was then treated with a saturated aqueous solution of Na_2_CO_3._ The aqueous phase was extracted with CH_2_Cl_2_ (3 × 5 mL) and washed with H_2_O and brine. The organic layer was separated, dried with Na_2_SO_4,_ and concentrated. The crude product was purified with SiO_2_ column chromatography and eluted with hexanes/EtOAc (3:1), yielding 0.13 g (65%) of **16** as a yellow oil. *R*_*f*_ = 0.31 (hexanes/EtOAc = 1:1); ^1^H NMR (500 MHz, CDCl_3_): δ 8.14 (d, *J* = 8.0 Hz, 1H), 7.46 (m, 2H), 7.38 (m, 1H), 7.29–7.20 (m, 5H), 6.45 (s, 1H), 4.70 (d, *J* = 3.0 Hz, 1H), 4.37 (d, *J* = 2.0 Hz, 1H), 3.90 (s, 3H), 3.87 (s, 3H), 3.77 (m, 1H), 3.56 (d, *J* = 13.5 Hz, 1H), 3.47 (d, *J* = 13.0 Hz, 1H), 3.40 (d, *J* = 11.5 Hz, 1H), 2.97 (dd, *J* = 11.5, 3.5 Hz, 1H), 2.78 (d, *J* = 13.5 Hz, 1H), 2.54 (s, 3H), 2.45 (m, 1H), 2.21 (m, 1H), 1.62 (m, 1H). ^13^C{^1^H} NMR (125 MHz, CDCl_3_): δ 200.6 (C), 163.8 (C), 160.8 (C), 157.6 (C), 147.4 (C), 143.4 (C), 138.6 (C), 134.0 (C), 132.9 (CH), 132.1 (CH), 130.9 (CH), 129.7 (C), 129.0 (2CH), 128.2 (2CH), 126.9 (CH), 126.9 (CH), 117.6 (C), 116.2 (C), 108.7 (CH_2_), 93.5 (CH), 62.0 (CH_2_), 60.9 (CH_2_), 56.0 (CH_3_), 56.0 (CH_3_), 54.0 (CH_2_), 38.9 (CH), 31.9 (CH_3_), 29.3 (CH_2_). HRMS-EI *m*/*z*: [M]^+^ calcd for C_30_H_30_ClNO_5_, 519.1813; found, 519.1820.
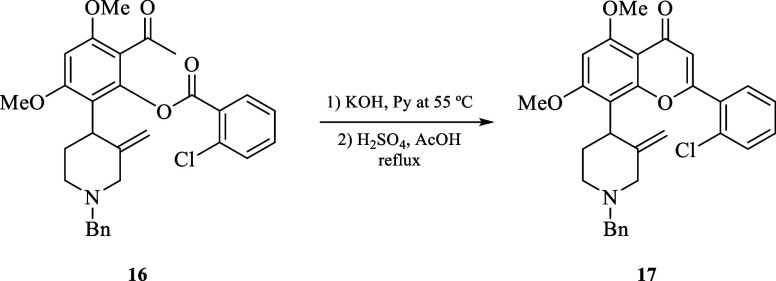


##### 8-(1-Benzyl-3-methylenepiperidin-4-yl)-2-(2-chlorophenyl)-5,7-dimethoxy-4H-chromen-4-one (**17**)

A solution of benzoate **16** (0.17 g, 0.38 mol) in pyridine (0.7 mL) with pulverized KOH (0.05 g, 0.95 mmol) was stirred at 55 °C for 1.5 h. The resulting black mixture was cooled with an ice bath, treated with a mixture of H_2_O (3 mL) and HCl (concentrated, 0.50 mL), and allowed to react for 30 min. A saturated aqueous solution of Na_2_CO_3_ was added until the pH reached 9. The aqueous layer was extracted by CH_2_Cl_2_ (3 × 7 mL), and the combined organic layers were washed with H_2_O and brine. The organic layer was dried with Na_2_SO_4_, and concentrated under reduced pressure, a yellow oil was obtained and used without further purification for the next step. The crude reaction was dissolved with a mixture of H_2_SO_4_ (0.05 mL) and glacial acetic acid (5.20 mL), and the reaction mixture was stirred at 100 °C for 1.5 h. Then, the reaction mixture was cooled to room temperature and a saturated solution of Na_2_CO_3_ was added until the pH reached 9. The aqueous layer was extracted with CH_2_Cl_2_ (3 × 7 mL), and the organic phase was dried with Na_2_SO_4_ and concentrated under reduced pressure. The residue was purified by column chromatography with SiO_2_ and eluted with EtOAc/Hex (2:1) to give 0.13 g (70%) of **17** as a yellow oil. *R*_*f*_ = 0.13 (EtOAc); ^1^H NMR (500 MHz, CDCl_3_): δ 7.76 (d, *J* = 8.0 Hz, 1H), 7.52 (dd, *J* = 7.8, 2.0 Hz, 1H), 7.44–7.38 (m, 2H), 7.34–7.23 (m, 5H), 6.63 (s, 1H), 6.48 (s, 1H), 4.74 (s, 1H), 4.26 (s, 1H), 4.11 (m, 1H), 4.03 (s, 3H), 3.95 (s, 3H), 3.59 (d, *J* = 13.5 Hz, 1H), 3.51 (d, *J* = 13.5 Hz, 1H), 3.47 (dd, *J* = 12.5, 2.0 Hz, 1H), 2.98 (m, 1H), 2.86 (dd, *J* = 12.0, 2.0 Hz, 1H), 2.55 (qd, *J* = 12.6, 4.0 Hz, 1H), 2.28 (td, *J* = 12.0, 2.5 Hz, 1H), 1.58 (m, 1H). ^13^C{^1^H} NMR (125 MHz, CDCl_3_): δ 178.1 (C), 162.1 (C), 160.0 (C), 159.5 (C), 157.3 (C), 144.3 (C), 138.7 (C), 132.8 (C), 131.6 (C), 131.5 (CH), 131.2 (CH), 131.0 (CH), 129.1 (2CH), 128.2 (2CH), 127.2 (CH), 127.0 (CH), 113.9 (CH), 110.2 (C), 109.1 (C), 108.0 (CH_2_), 92.2 (CH), 62.0 (CH_2_), 61.0 (CH_2_), 56.5 (CH_3_), 56.0 (CH_3_), 54.2 (CH_2_), 38.3 (CH), 29.1 (CH_2_). HRMS-FAB *m*/*z*: [M + H]^+^ calcd for C_30_H_29_ClNO_4_, 502.1785; found, 502.1776.

#### Synthetic Application; Chromone Alkaloids

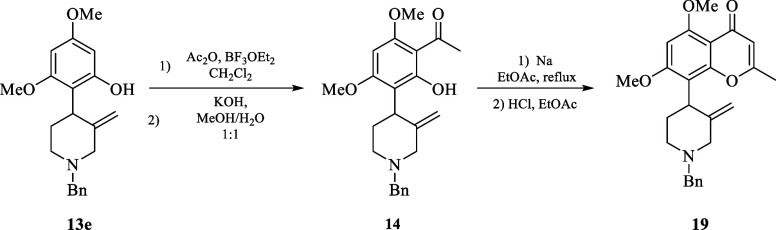



##### 8-(1-Benzyl-3-methylenepiperidin-4-yl)-5,7-dimethoxy-2-methyl-4H-chromen-4-one (**19**)

The intermediate **14** was prepared from **13e** (0.11 g, 0.30 mmol) following the procedure described for the synthesis of **16**. Without further purification, intermediate **14** was dissolved in dry EtOAc (5 mL) and sodium (80.0 mg, 3.48 mmol) was added. The reaction mixture was heated to reflux for 2 h, then, a saturated solution of Na_2_CO_3_ was added (2 mL) and the aqueous layer was extracted with EtOAc (3 × 10 mL). The organic phase was separated, dried with Na_2_SO_4_, and concentrated under reduced pressure. The residue was dissolved in EtOAc (2 mL) and concentrated HCl (0.01 mL) was added at room temperature. The reaction was stirred for 1 h and quenched with a solution of Na_2_CO_3_ until get pH = 9. The aqueous layer was extracted with EtOAc (3 × 10 mL), next, the organic phase was collected, dried, and concentrated. The residue was purified by column chromatography with SiO_2_ and eluted with hexanes: EtOAc (1:1) to give 80.0 mg (67%) of **19** as a yellow oil. *R*_*f*_ = 0.10 (EtOAc); ^1^H NMR (500 MHz, CDCl_3_): δ 7.40–7.32 (m, 4H), 7.28 (m, 1H), 6.42 (s, 1H), 6.00 (s, 1H), 4.70 (s,1H), 4.17 (m, 1H), 4.03 (m, 1H), 3.98 (s, 3H), 3.91 (s, 3H), 3.70 (d, *J* = 13.0 Hz, 1H), 3.62 (d, *J* = 13.0 Hz, 1H), 3.47 (dd, *J* = 12.5, 2.0 Hz, 1H), 3.06 (m, 1H), 2.91 (dd, *J* = 12.0, 1.5 Hz, 1H), 2.57 (qd, *J* = 12.5, 3.9 Hz, 1H), 2.37 (td, *J* = 12.0, 2.5 Hz, 1H), 2.30 (s, 3H), 1.60 (m, 1H). ^13^C{^1^H} NMR (125 MHz, CDCl_3_): δ 178.3 (C), 163.1 (C), 161.5 (C), 159.8 (C), 157.3 (C), 143.9 (C), 138.4 (C), 129.2 (2CH), 128.3 (2CH), 127.1 (CH), 111.3 (CH), 109.7 (C), 108.8 (C), 107.8 (CH_2_), 91.8 (CH), 61.9 (CH_2_), 60.8 (CH_2_), 56.3 (CH_3_), 55.9 (CH_3_), 54.1 (CH_2_), 38.2 (CH), 29.2 (CH_2_), 20.0 (CH_3_). HRMS-EI *m*/*z*: [M]^+^ calcd for C_25_H_27_NO_4_, 405.1940; found, 405.1940.

#### Synthesis of Chromone/Flavone Piperidine Alkaloids Containing Methyl Carbamate (Moc) Group

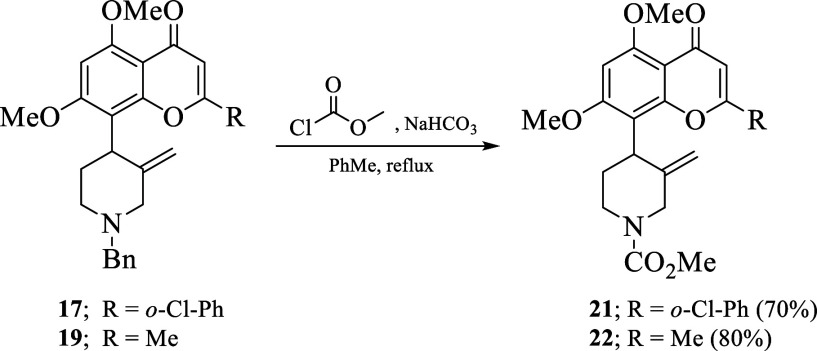



To a suspension of **17** or **19** (1.0 mmol) and NaHCO_3_ (85.0 mg, 1.0 mmol) in dry toluene (20 mL) under N_2_ atmosphere was added dropwise methyl chloroformate (0.1 mL, 1.2 mmol). Next, the reaction mixture was heated to reflux for 2 h. Then, the mixture of reaction was cooled, and the solids were filtered and washed with EtOAc (3 × 5 mL). The crude was concentrated for purification.

##### Methyl 4-(2-(2-Chlorophenyl)-5,7-dimethoxy-4-oxo-4*H*-chromen-8-yl)-3-methylenepiperidine-1-carboxylate (**21**)

Following the debenzylation protocol, 0.17 g of **21** (70%) was obtained as a colorless oil from **17** (0.26 g, 0.52 mmol). Purified with SiO_2_ column chromatography and eluted with hexanes/EtOAc (1:1). *R*_*f*_ = 0.22 (EtOAc); ^1^H NMR (500 MHz, CD_3_CN): δ 7.67 (d, *J* = 7.5 Hz, 1H), 7.55 (dd, *J* = 7.8, 1.3 Hz, 1H), 7.49 (td, *J* = 7.6, 1.8 Hz, 1H), 7.45–7.40 (m, 1H), 6.62 (s, 1H), 6.39 (br, 1H), 4.82 (s, 1H), 4.52 (m, 1H), 4.28 (s, 1H), 4.23 (m, 1H), 4.01 (m, 1H), 3.94 (s, 3H), 3.91 (s, 3H), 3.57 (m, 4H), 3.01 (br, 1H), 2.31 (qd, *J* = 13.0, 4.5 Hz, 1H), 1.60 (d, *J* = 11.0 Hz, 1H). ^13^C{^1^H} NMR (125 MHz, CD_3_CN): δ 177.4 (C), 162.9 (C), 160.9 (C), 160.0 (C), 157.9 (C), 156.2 (C), 144.2 (C), 133.0 (C), 132.8 (CH), 132.2 (C), 132.0 (CH), 131.6 (CH), 128.5 (CH), 114.4 (CH), 110.3 (C), 109.5 (C), 109.4 (CH_2_), 93.8 (CH), 56.9 (CH_3_), 56.8 (CH_3_), 52.9 (CH_3_), 51.2 (CH_2_), 45.5 (CH_2_), 38.3 (CH), 30.2 (CH_2_). HRMS-FAB *m*/*z*: [M + H]^+^ calcd for C_25_H_25_ClNO_6_, 470.1370; found, 470.1345.

##### Methyl 4-(5,7-Dimethoxy-2-methyl-4-oxo-4H-chromen-8-yl)-3-methylenepiperidine-1-carboxylate (**22**)

Following the debenzylation protocol, 0.30 g of **22** (80%) was obtained as a white solid (mp = 155–157 °C) from **19** (0.41 g, 1.01 mmol). Purified with SiO_2_ column chromatography and eluted with hexanes/EtOAc (1:2). *R*_*f*_ = 0.13 (EtOAc); ^1^H NMR (500 MHz, CDCl_3_): δ 6.41 (s, 1H), 5.99 (d, *J* = 0.5 Hz, 1H), 4.83 (m, 1H), 4.60 (m, 1H), 4.25 (br, 2H), 4.15 (m, 1H), 3.99 (s, 3H), 3.91 (s, 3H), 3.74 (s, 3H), 3.70 (m, 1H), 3.08 (br, 1H), 2.40 (br, 1H), 2.22 (d, *J* = 1.0 Hz, 3H), 1.70 (d, *J* = 13.0 Hz, 1H). ^13^C{^1^H} NMR (125 MHz, CDCl_3_): δ 178.2 (C), 163.0 (C), 161.3 (C), 160.3 (C), 160.0 (C), 157.2 (C), 156.1 (C), 142.5 (C), 111.5 (CH), 109.6 (C), 108.9 (CH_2_), 91.8 (CH), 56.5 (CH_3_), 56.0 (CH_3_), 52.7 (CH_3_), 50.9 (CH_2_), 45.1 (CH_2_), 37.3 (CH), 29.5 (CH_2_), 19.9 (CH_3_). HRMS-EI *m*/*z*: [M]^+^ calcd for C_20_H_23_NO_6_, 373.1525; found, 373.1520.

##### Installation of the *cis*-hydroxyl Group at C3′ Position (Oxidative Cleavage/Reduction)

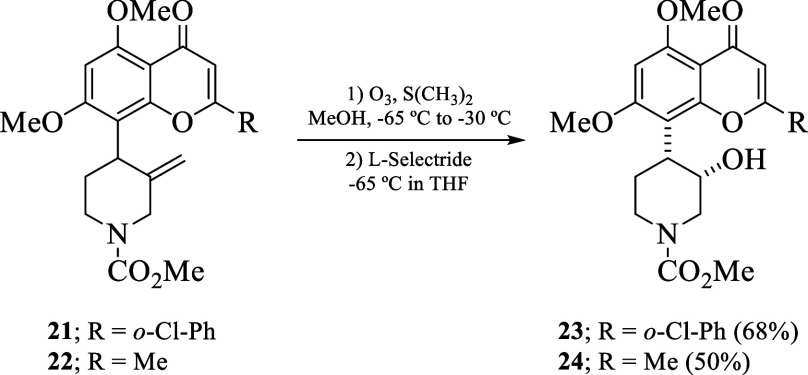



A stream of ozone was bubbled through a solution of the corresponding alkene **21** or **22** (0.42 mmol) in MeOH (reagent grade, 10.0 mL) at −65 °C. Once raw material was consumed, N_2_ was bubbled to the reaction, and S(CH_3_)_2_ (0.06 mL, 0.84 mmol) was added. The reaction mixture was stirred for 30 min at −35 °C and then, the solvent was removed at a reduced pressure. The residue (without purification) was solved in 10 mL of anhydrous THF. Next, L-selectride was dropwise added (0.5 mL of 1.0 M solution, 0.50 mmol) at −65 °C. The reaction mixture was stirred at the same temperature for 2 h. The reaction was quenched with a saturated solution of Rochelle salt (5.0 mL) and stirred at room temperature for 20 min. The aqueous layer was extracted with CH_2_Cl_2_ (4 × 10 mL) then the organic layer was separated, dried with Na_2_SO_4,_ and concentrated for purification.

##### Methyl (3*S*,4*R*)-4-(2-(2-Chlorophenyl)-5,7-dimethoxy-4-oxo-4*H*-chromen-8-yl)-3-hydroxypiperidine-1-carboxylate (**23**)

Following the oxidative cleavage/reduction protocol, 0.14 g of **23** (68%) was obtained as a yellow oil from **21** (0.20 g, 0.42 mmol). Purified with SiO_2_ column chromatography and eluted with EtOAc. *R*_*f*_ = 0.40 (EtOAc/MeOH = 10:1); ^1^H NMR (500 MHz, CD_3_CN): δ 7.81 (dd, *J* = 7.5, 2.0 Hz, 1H), 7.56 (d, *J* = 7.5 Hz, 1H), 7.50 (td, *J* = 7.5, 1.5 Hz, 1H), 7.46 (t, *J* = 7.5 Hz, 1H), 6.56 (s, 1H), 6.32 (s, 1H), 4.12 (m, 2H), 3.93 (s, 3H), 3.89 (s, 3H), 3.86 (m, 1H), 3.62 (s, 3H), 3.57 (dt, *J* = 12.5, 2.7 Hz, 1H), 2.96 (br, 1H), 2.82 (m, 1H), 2.75 (br, 1H), 1.46 (d, *J* = 12.0 Hz, 1H). ^13^C{^1^H} NMR (125 MHz, CD_3_CN): δ 177.5 (C), 163.6 (C), 160.6 (C), 160.4 (C), 158.4 (C), 157.3 (C), 133.0 (C), 132.9 (CH), 132.5 (C), 132.2 (CH), 131.6 (CH), 128.5 (CH), 114.4 (CH), 111.4 (C), 109.6 (C), 94.1 (CH), 69.7 (CH), 57.1 (CH_3_), 56.8 (CH_3_), 52.9 (CH_3_), 51.7 (CH_2_), 46.1 (CH_2_), 40.0 (CH), 24.9 (CH_2_). HRMS-FAB *m*/*z*: [M + H]^+^ calcd for C_24_H_25_ClNO_7_ 474.1320; found, 474.1315.

##### Methyl (3*S*,4*R*)-4-(5,7-Dimethoxy-2-methyl-4-oxo-4H-chromen-8-yl)-3-hydroxypiperidine-1-carboxylate (**24**)

Following the oxidation protocol, 26.0 mg of **24** (50%) was obtained as a yellow oil from **22** (0.05 g, 0.14 mmol). Purified with SiO_2_ column chromatography and eluted with EtOAc/MeOH (1.0:0.01). *R*_*f*_ = 0.23 (EtOAc/MeOH = 10:1); ^1^H NMR (500 MHz, CDCl_3_): δ 6.42 (s, 1H), 6.02 (s, 1H), 4.43 (br, 1H), 4.31 (br, 1H), 3.97 (s, 3H), 3.96 (s, 3H), 3.94 (br, 1H), 3.75 (s, 3H), 3.60 (m, 1H), 3.02 (m, 1H), 2.87 (m, 2H), 2.31 (s, 3H), 1.53 (m, 1H). ^13^C{^1^H} NMR (125 MHz, CDCl_3_): δ 177.9 (C), 162.8 (C), 161.7 (C), 160.1 (C), 157.3 (C), 157.0 (C), 111.8 (CH), 109.8 (C), 109.2 (C), 92.2 (CH), 69.4 (CH), 56.5 (CH_3_), 56.2 (CH_3_), 52.9 (CH_3_), 51.0 (CH_2_), 45.6 (CH_2_), 38.9 (CH), 24.0 (CH_2_), 20.0 (CH_3_). HRMS-FAB *m*/*z*: [M + H]^+^ calcd for C_19_H_24_NO_7_ 378.1553; found, 378.1553.

#### Formal Synthesis of Rohitukine and Flavopiridol

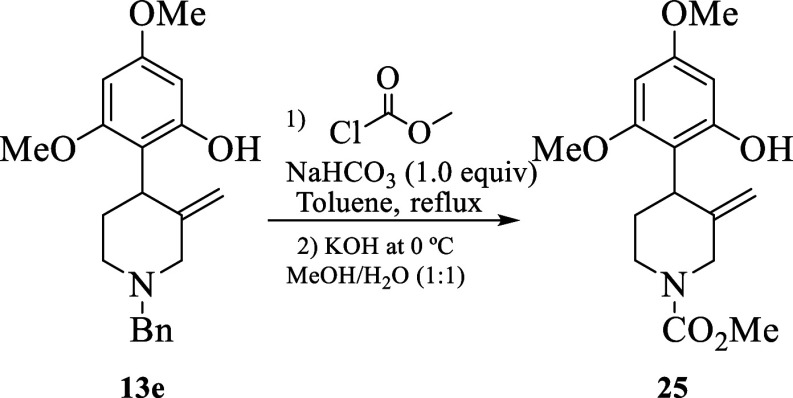



##### Methyl 4-(2-Hydroxy-4,6-dimethoxyphenyl)-3-methylenepiperidine-1-carboxylate (**25**)

To a stirred solution of **13e** (0.30 g, 0.88 mmol) and sodium bicarbonate (0.074 g, 0.88 mmol) in 18.0 mL of toluene anhydrous was added dropwise methyl chloroformate (0.17 mL, 2.20 mmol). The reaction mixture was heated to reflux and monitored by TLC until the total consumption of **13e**. The resulting mixture was allowed to room temperature, filtered, and concentrated under reduced pressure. The product without purification was dissolved in a mixture of MeOH/H_2_O (1:1, 9 mL), cooled to 0 °C, and treated with KOH (powder, 2.20 mmol), the reaction mixture was stirred for 1 h. The MeOH was removed, and the aqueous phase was extracted with CH_2_Cl_2_ (3 × 10 mL), the organic phase was dried with Na_2_SO_4_ and concentrated. The residue was purified by column chromatography with SiO_2_ and eluted with hexanes/EtOAc (3:1) to give 0.25 g (90%) of **25** as a colorless oil. *R*_*f*_ = 0.70 (EtOAc); ^1^H NMR (500 MHz, CD_3_CN): δ 6.89 (br, 1H), 6.12 (d, *J* = 2.0 Hz, 1H), 6.06 (d, *J* = 2.5 Hz, 1H), 4.77 (s, 1H), 4.51 (d, *J* = 14.0 Hz, 1H), 4.28 (s, 1H), 4.08 (br, 1H), 3.95 (m, 1H), 3.73 (s, 3H), 3.70 (s, 3H), 3.65 (s, 3H), 3.58 (m, 1H), 3.00 (br, 1H), 2.35 (qd, *J* = 12.9, 4.4 Hz, 1H), 1.53 (m, 1H). ^13^C{^1^H} NMR (125 MHz, CD_3_CN): δ 160.7 (C), 160.6 (C), 157.2 (C), 156.6 (C), 144.9 (C), 109.7 (C), 109.1 (CH_2_), 95.0 (CH), 92.0 (CH), 56.2 (CH_3_), 55.8 (CH_3_), 53.0 (CH_3_), 51.3 (CH_2_), 45.7 (CH_2_), 38.2 (CH), 30.3 (CH_2_). HRMS-FAB *m*/*z*: [M + H]^+^ calcd for C_16_H_22_NO_5_ 308.1498; found, 308.1508.
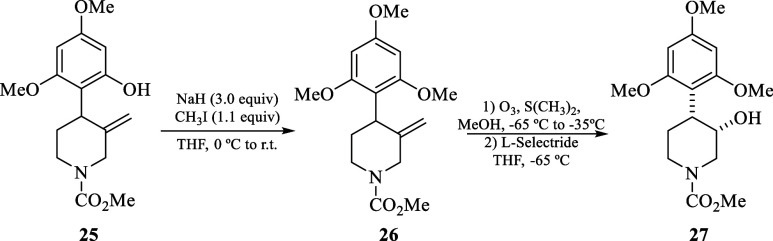


##### Methyl (3*S*,4*R*)-3-Hydroxy-4-(2,4,6-trimethoxyphenyl)piperidine-1-carboxylate (**27**)

A solution of **25** (0.25 g, 0.81 mmol) in 8 mL of THF anhydrous was added to a flask with NaH (0.58 g, 2.43 mmol) under N_2_ atmosphere at 0 °C temperature. After 10 min 0.06 mL of CH_3_I (0.89 mmol) was added, and the reaction was carried to room temperature. The reaction mixture was stirred for 1h, quenched with H_2_O (2 mL), and extracted with CH_2_Cl_2_ (4 × 10 mL). The organic phase was dried with Na_2_SO_4_ and concentrated for the next step. Then, a stream of ozone was bubbled through a solution of alkene (without purification) **26** in MeOH (8.0 mL) at −65 °C for 1.5 h followed by bubbled N_2_ to the reaction and addition of S(CH_3_)_2_ (0.12 mL, 1.62 mmol). The reaction mixture was stirred for 30 min at −35 °C. The solvent was removed at reduced pressure, and the residue was solved in 10 mL of THF anhydrous. Then, a 1.0 M solution of L-selectride was dropwise added (0.98 mL, 0.97 mmol) at −65 °C. After 2 h, the reaction was quenched with a saturated solution of Rochelle salt (5.0 mL) and stirred to room temperature for 20 min. The aqueous layer was extracted with CH_2_Cl_2_ (4 × 10 mL) and the organic layer was separated, dried with Na_2_SO_4,_ and concentrated. The crude product was purified by column chromatography with SiO_2_ and eluted with hexanes/EtOAc (2:1) to give 0.15 g (59%) of **27** as a white solid, mp = 86–89 °C. *R*_*f*_ = 0.53 (EtOAc);^1^H NMR (500 MHz, CDCl_3_): δ 6.17 (s, 2H), 4.38 (br, 1H), 4.20 (br, 1H), 3.84 (br, 1H), 3.81 (s, 9H), 3.73 (s, 3H), 3.54 (m, 1H), 2.97 (m, 1H), 2.83 (br, 1H), 2.74 (qd, *J* = 13.0, 4.0 Hz, 1H), 1.36 (d, *J* = 13.5 Hz, 1H). ^13^C{^1^H} NMR (125 MHz, CDCl_3_): δ 160.1 (C), 159.1 (2C), 157.2 (C), 111.3 (C), 91.6 (2CH), 69.9 (CH), 56.0 (2CH_3_), 55.5 (CH_3_), 52.7 (CH_3_), 50.9 (CH_2_), 45.5 (CH_2_), 37.8 (CH), 24.0 (CH_2_). HRMS-EI *m*/*z*: [M]^+^ calcd for C_16_H_23_NO_6_, 325.1525; found, 325.1523.
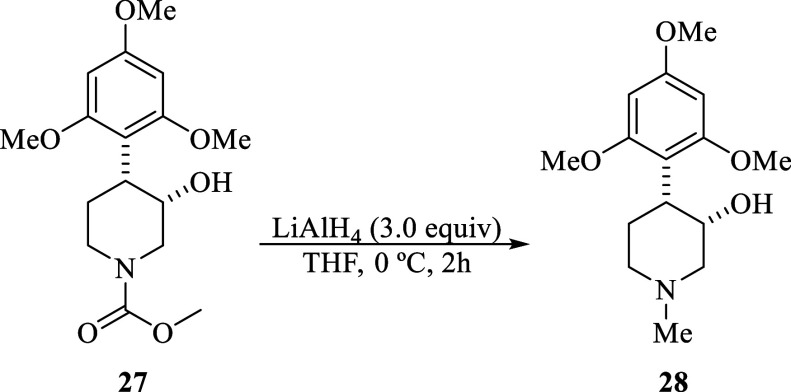


##### (3*S*,4*R*)-1-Methyl-4-(2,4,6-trimethoxyphenyl)piperidin-3-ol (**28**)

A solution of **27** (65.0 mg, 0.2 mmol) in THF anhydrous (4.0 mL) was added to a flask with LiAlH_4_ (22.0 mg, 0.6 mmol) at 0 °C. The reaction mixture was stirred at the same temperature for 2 h. Then, 2.0 mL of H_2_O was added, and the aqueous phase was extracted with CH_2_Cl_2_ (4 × 10 mL). The organic layer was separated, dried with Na_2_SO_4_, and concentrated. The crude product was purified by column chromatography with SiO_2_ and eluted with a gradient system MeOH/EtOAc (0.1:1) to MeOH to give 31.0 mg (65%) of **28** as a colorless oil. The NMR data agree with those reported by Naik.^8a^*R*_*f*_ = 0.10 (EtOAc/MeOH = 10:1); ^1^H NMR (500 MHz, CDCl_3_): δ 6.16 (s, 2H), 3.84 (br, 1H), 3.80 (s, 9H), 3.37 (dt, *J* = 13.5, 3.3 Hz, 1H), 2.99 (m, 2H), 2.89 (qd, *J* = 13.0, 3.9 Hz, 1H), 2.31 (s, 3H), 2.14 (d, *J* = 12.5 Hz, 1H), 2.04 (td, *J* = 11.9, 2.8 Hz, 1H), 1.40 (dd, *J* = 13.5, 3.5 Hz, 1H). ^13^C{^1^H} NMR (125 MHz, CDCl_3_): δ 159.8 (C), 159.3 (2C), 111.7 (C), 91.6 (2CH), 70.6 (CH), 62.7 (CH_2_), 57.2 (CH_2_), 55.9 (2CH_3_), 55.4 (CH_3_), 46.7 (CH_3_), 37.2 (CH), 24.7 (CH_2_). HRMS-FAB *m*/*z*: [M + H]^+^ calcd for C_15_H_24_NO_4_, 282.1705; found, 282.1711.

## Data Availability

The data underlying this study are available in the published article and its Supporting Information.

## Supporting Information Available

^1^H and ^13^C{^1^H} spectra for new compounds (PDF)
