# Supplementary material for: Synthetic Approach to Chromone and Flavonoid Piperidine Alkaloids
Source: J Org Chem. 2024 Oct 22;89(21):15808–21. doi: 10.1021/acs.joc.4c01926 (PMC11536364; doi:10.1021/acs.joc.4c01926)
Supplement: Supplementary file 1 — jo4c01926_si_001.pdf [file jo4c01926_si_001.pdf]

Synthetic Approach to Chromone and Flavonoid Piperidine Alkaloids

Karen A. Guarneros-Cruz, Silvano Cruz-Gregorio, Julio Romero-Ibáñez, Rosa L. Meza-León, and Fernando Sartillo-Piscil *

Centro de Investigación en Síntesis Orgánica de la Facultad de Ciencias Químicas, Benemérita Universidad Autónoma de Puebla (BUAP), 14 Sur Esq. San Claudio, Col. San Manuel, 72570, Puebla, Mexico.

fernando.sartillo@correo.buap.mx . Fax: +522222454972; Tel: +52 222 2955500 ext. 7391

KEYWORDS: C-H phenolization, alkaloids, chromones, flavones, Claisen rearrangement.

Table of contents:

General considerations.....S4
^{1}H NMR spectrum of compound **8a** (500 MHz, $CDCl_3$): S5
$^{13}C\{^{1}H\}$ NMR spectrum of compound **8a** (125 MHz, $CDCl_3$): S6
^{1}H NMR spectrum of compound **8b** (500 MHz, $CDCl_3$): S7
$^{13}C\{^{1}H\}$ NMR spectrum of compound **8b** (125 MHz, $CDCl_3$): S8
H NMR spectrum of compound **9** (500 MHz, $CDCl_3$):.....S9
$^{13}C\{^{1}H\}$ NMR spectrum of compound **9** (125 MHz, $CDCl_3$): S10
^{1}H NMR spectrum of compound **12a** (500 MHz, $CDCl_3$):.....S11
$^{13}C\{^{1}H\}$ NMR spectrum of compound **12a** (125 MHz, $CDCl_3$): S12
^{1}H NMR spectrum of compound **12b** (500 MHz, $CDCl_3$): S13
$^{13}C\{^{1}H\}$ NMR spectrum of compound **12b** (125 MHz, $CDCl_3$): S14
^{1}H NMR spectrum of compound **12c** (500 MHz, $CDCl_3$):.....S15
$^{13}C\{^{1}H\}$ NMR spectrum of compound **12c** (125 MHz, $CDCl_3$):.....S16
^{1}H NMR spectrum of compound **12d** (500 MHz, $CDCl_3$): S17
$^{13}C\{^{1}H\}$ NMR spectrum of compound **12d** (125 MHz, $CDCl_3$): S18
^{1}H NMR spectrum of compound **12e** (500 MHz, $CDCl_3$):.....S19
$^{13}C\{^{1}H\}$ NMR spectrum of compound **12e** (125 MHz, $CDCl_3$):.....S20
^{1}H NMR spectrum of compound **12f** (500 MHz, $CDCl_3$): S21
$^{13}C\{^{1}H\}$ NMR spectrum of compound **12f** (125 MHz, $CDCl_3$): S22
^{1}H NMR spectrum of compound **12g** (500 MHz, $CDCl_3$):.....S23
$^{13}C\{^{1}H\}$ NMR spectrum of compound **12g** (125 MHz, $CDCl_3$): S24
^{1}H NMR spectrum of compound **12h** (500 MHz, $CDCl_3$): S25
$^{13}C\{^{1}H\}$ NMR spectrum of compound **12h** (125 MHz, $CDCl_3$): S26
^{1}H NMR spectrum of compound **12i** (500 MHz, $CDCl_3$): S27
$^{13}C\{^{1}H\}$ NMR spectrum of compound **12i** (125 MHz, $CDCl_3$): S28
^{1}H NMR spectrum of compound **12j** (500 MHz, $CDCl_3$): S29
$^{13}C\{^{1}H\}$ NMR spectrum of compound **12j** (125 MHz, $CDCl_3$): S30
^{1}H NMR spectrum of compound **12k** (500 MHz, $CDCl_3$): S31
$^{13}C\{^{1}H\}$ NMR spectrum of compound **12k** (125 MHz, $CDCl_3$): S32
^{1}H NMR spectrum of compound **S2** (500 MHz, $CDCl_3$): S33
$^{13}C\{^{1}H\}$ NMR spectrum of compound **S2** (125 MHz, $CDCl_3$): S34
^{1}H NMR spectrum of compound **12l** (500 MHz, $CDCl_3$): S35
$^{13}C\{^{1}H\}$ NMR spectrum of compound **12l** (125 MHz, $CDCl_3$): S36
^{1}H NMR spectrum of compound **10** (500 MHz, $CDCl_3$): S37
$^{13}C\{^{1}H\}$ NMR spectrum of compound **10** (125 MHz, $CDCl_3$): S38
Fig. 1. COSY of ***10*** S39
^{1}H NMR spectrum of compound **13a** (500 MHz, $CDCl_3$):.....S40
$^{13}C\{^{1}H\}$ NMR spectrum of compound **13a** (125 MHz, $CDCl_3$): S41
^{1}H NMR spectrum of compound **13b** (500 MHz, $CDCl_3$): S42
$^{13}C\{^{1}H\}$ NMR spectrum of compound **13b** (125 MHz, $CDCl_3$): S43
^{1}H NMR spectrum of compound **13d** (500 MHz, $CDCl_3$): S44
$^{13}C\{^{1}H\}$ NMR spectrum of compound **13d** (125 MHz, $CDCl_3$): S45
^{1}H NMR spectrum of compound **13e** (500 MHz, $CDCl_3$):.....S46
$^{13}C\{^{1}H\}$ NMR spectrum of compound **13e** (125 MHz, $CDCl_3$):.....S47
^{1}H NMR spectrum of compound **13g** (500 MHz, $CDCl_3$):.....S48
$^{13}C\{^{1}H\}$ NMR spectrum of compound **13g** (125 MHz, $CDCl_3$): S49
^{1}H NMR spectrum of compound **13gg** (500 MHz, $CDCl_3$):.....S50
$^{13}C\{^{1}H\}$ NMR spectrum of compound **13gg** (125 MHz, $CDCl_3$):.....S51

^{1}H NMR spectrum of compound **13j** (500 MHz, $CDCl_3$): S52
$^{13}C\{^{1}H\}$ NMR spectrum of compound **13j** (125 MHz, $CDCl_3$): S53
^{1}H NMR spectrum of compound **13k** (500 MHz, $CDCl_3$): S54
$^{13}C\{^{1}H\}$ NMR spectrum of compound **13k** (125 MHz, $CDCl_3$): S55
^{1}H NMR spectrum of compound **13l** (500 MHz, $CDCl_3$): S56
$^{13}C\{^{1}H\}$ NMR spectrum of compound **13l** (125 MHz, $CDCl_3$): S57
^{1}H NMR spectrum of compound **16** (500 MHz, $CDCl_3$): S58
$^{13}C\{^{1}H\}$ NMR spectrum of compound **16** (125 MHz, $CDCl_3$): S59
^{1}H NMR spectrum of compound **17** (500 MHz, $CDCl_3$): S60
$^{13}C\{^{1}H\}$ NMR spectrum of compound **17** (125 MHz, $CDCl_3$): S61
^{1}H NMR spectrum of compound **19** (500 MHz, $CDCl_3$): S62
$^{13}C\{^{1}H\}$ NMR spectrum of compound **19** (125 MHz, $CDCl_3$): S63
^{1}H NMR spectrum of compound **21** (500 MHz, CD_3CN): S64
$^{13}C\{^{1}H\}$ NMR spectrum of compound **21** (125 MHz, CD_3CN): S65
^{1}H NMR spectrum of compound **22** (500 MHz, $CDCl_3$): S66
$^{13}C\{^{1}H\}$ NMR spectrum of compound **22** (125 MHz, $CDCl_3$): S67
^{1}H NMR spectrum of compound **23** (500 MHz, $CDCl_3$): S68
$^{13}C\{^{1}H\}$ NMR spectrum of compound **23** (125 MHz, $CDCl_3$): S69
^{1}H NMR spectrum of compound **23** (500 MHz, CD_3CN): S70
$^{13}C\{^{1}H\}$ NMR spectrum of compound **23** (125 MHz, CD_3CN): S71
^{1}H NMR spectrum of compound **24** (500 MHz, $CDCl_3$): S72
$^{13}C\{^{1}H\}$ NMR spectrum of compound **24** (125 MHz, $CDCl_3$): S73
^{1}H NMR spectrum of compound **25** (500 MHz, CD_3CN): S74
$^{13}C\{^{1}H\}$ NMR spectrum of compound **25** (125 MHz, CD_3CN): S75
^{1}H NMR spectrum of compound **27** (500 MHz, $CDCl_3$): S76
$^{13}C\{^{1}H\}$ NMR spectrum of compound **27** (125 MHz, $CDCl_3$): S77
^{1}H NMR spectrum of compound **28** (500 MHz, $CDCl_3$): S78
$^{13}C\{^{1}H\}$ NMR spectrum of compound **28** (125 MHz, $CDCl_3$): S79
COSY NMR spectrum of compound **28** (500 MHz, CDCl3): S80
HSQC NMR spectrum of compound **28** (500 MHz, CDCl3): S81
Table 1. NMR data reported by Naik.[8a] and our work S82

General considerations

NMR spectra were obtained on Bruker-500 (500 MHz) spectrometer using TMS as an internal reference for ^{1}H (0.00 ppm) and $CDCl_3$ for ^{13}C (77.16 ppm) unless otherwise noted. Chemical shifts (δ) are stated in parts per million (ppm) and Hz for the coupling constants (J). The following abbreviations were used to explain the multiplicities: s = singlet, d = doublet, t = triplet, q = quartet, m = multiplet, br = broadened, dd = doublet of doublets, td = triplet of doublets, qd = quartet of doublets.

Melting points were not corrected and carried out on a Fisher-Scientific 12-144 melting point apparatus. High-resolution mass spectra-electron impact mode (HRMS-EI). High-resolution mass spectra-fast atom bombardment mode (HRMS-FAB). High-resolution mass spectra-electrospray ionization mode (HRMS-ESI).

^{1}H NMR spectrum of compound 8a (500 MHz, CDCl$_3$):

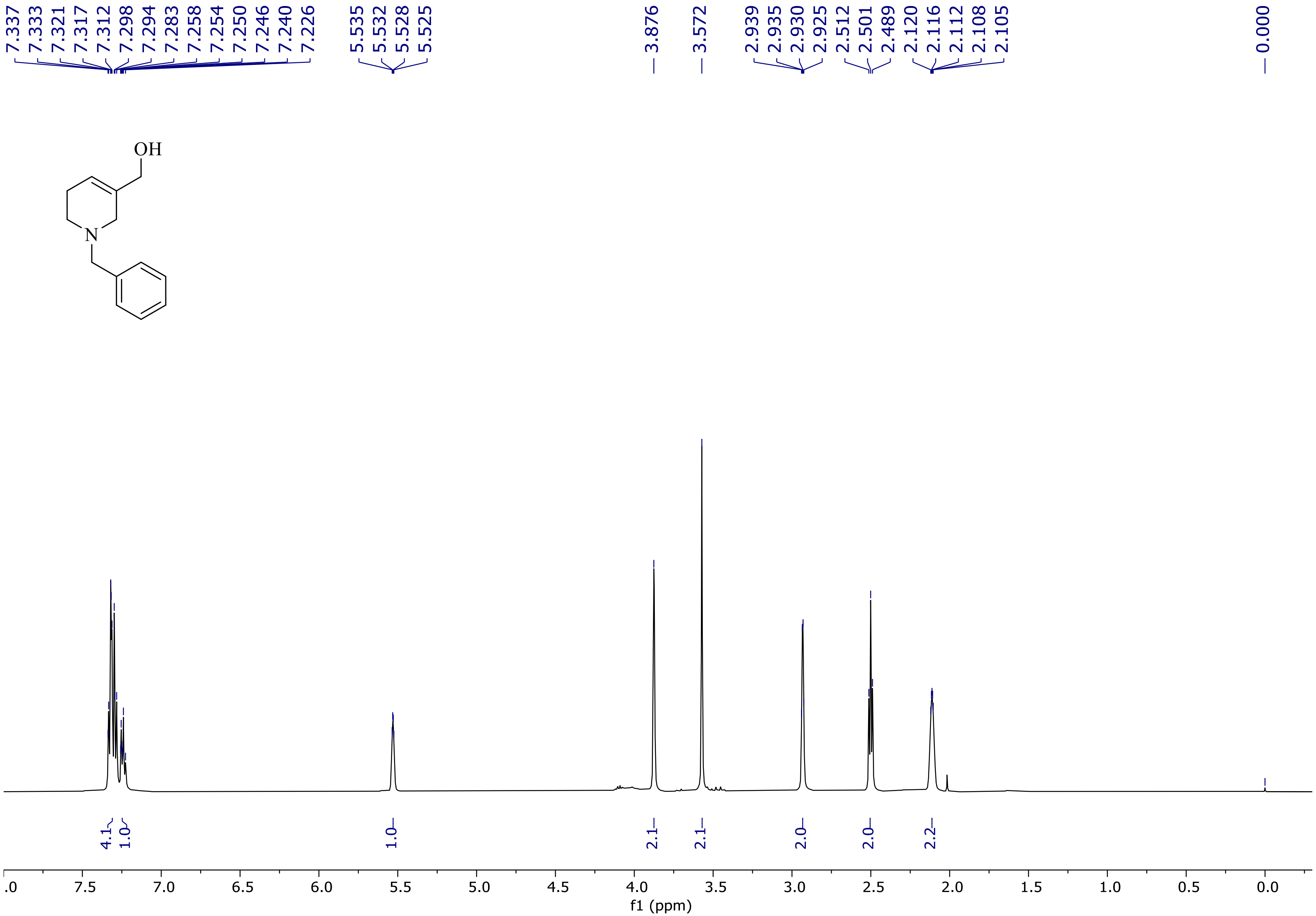

$^{13}C\{^1H\}$ NMR spectrum of compound 8a (125 MHz, $CDCl_3$):

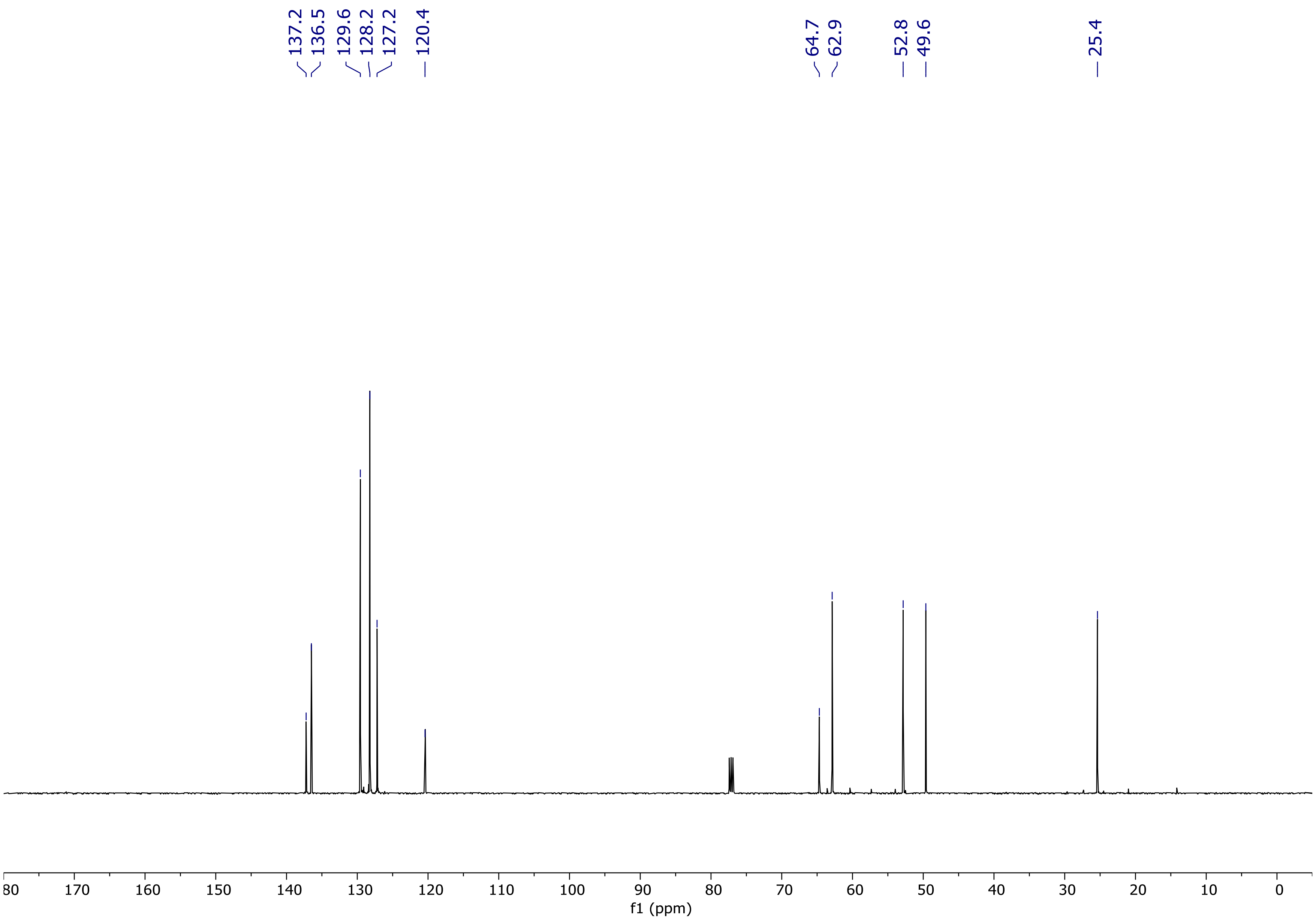

^{1}H NMR spectrum of compound 8b (500 MHz, $CDCl_3$):

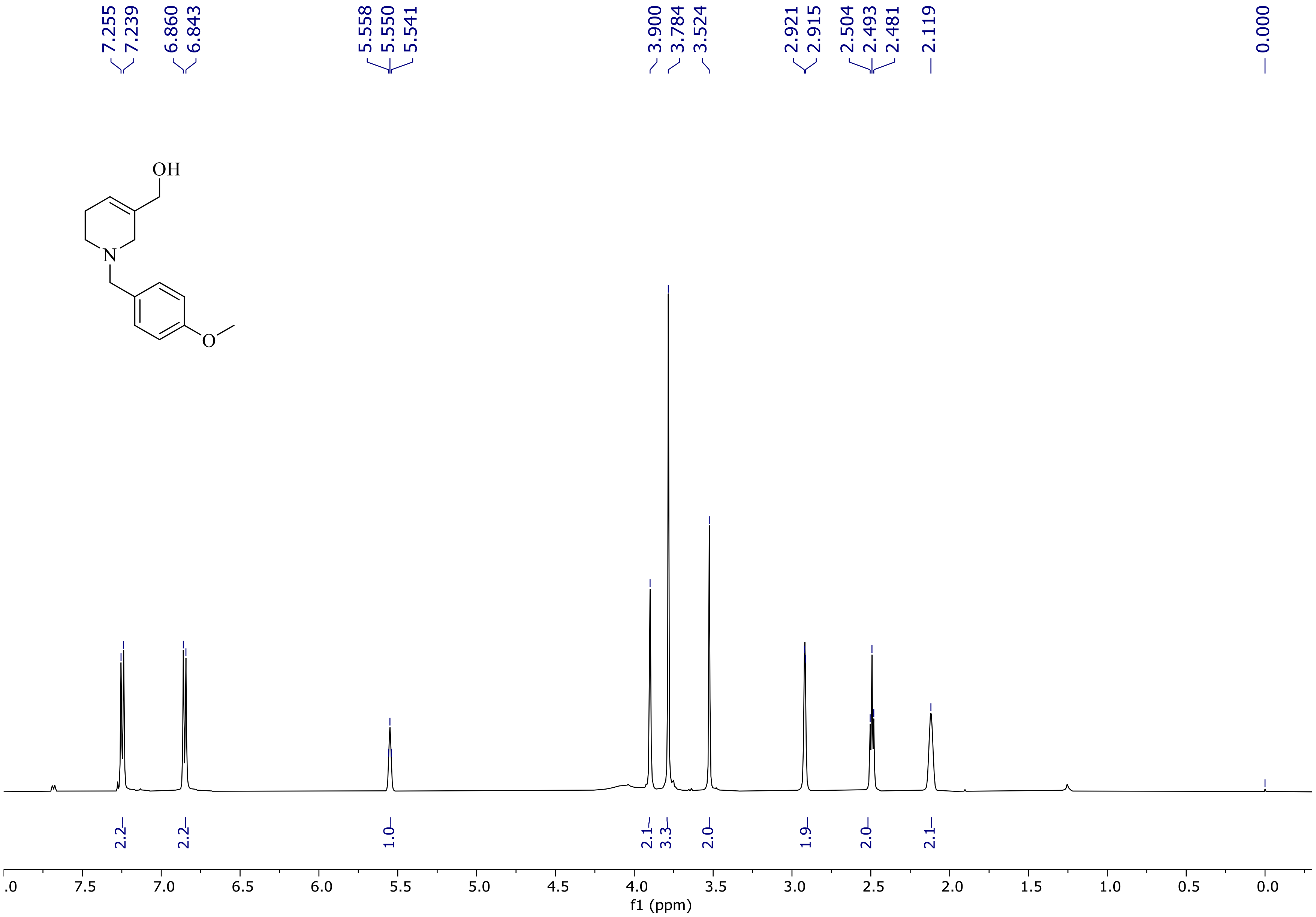

$^{13}C\{^1H\}$ NMR spectrum of compound 8b (125 MHz, $CDCl_3$):

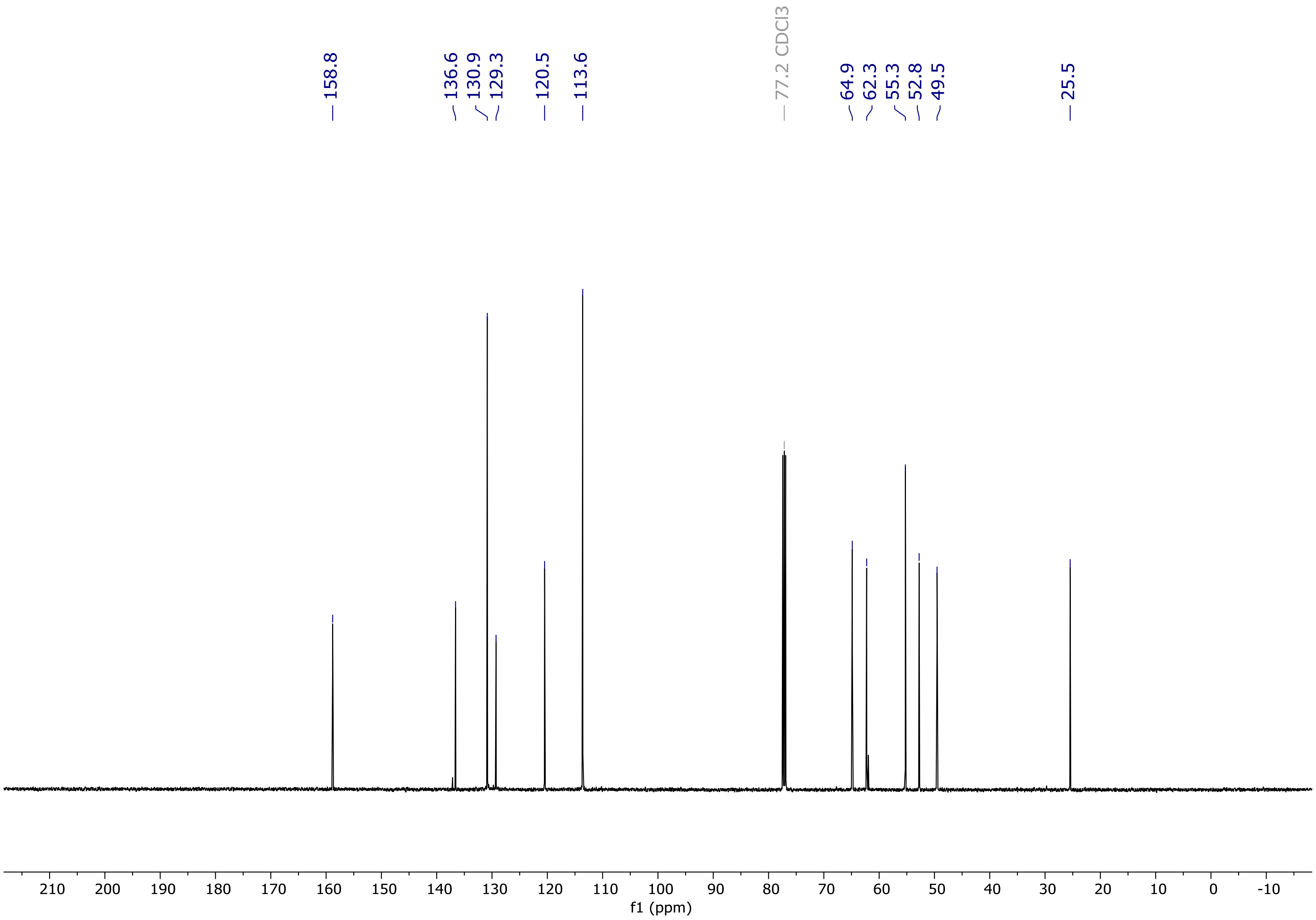

^{1}H NMR spectrum of compound 9 (500 MHz, CDCl3):

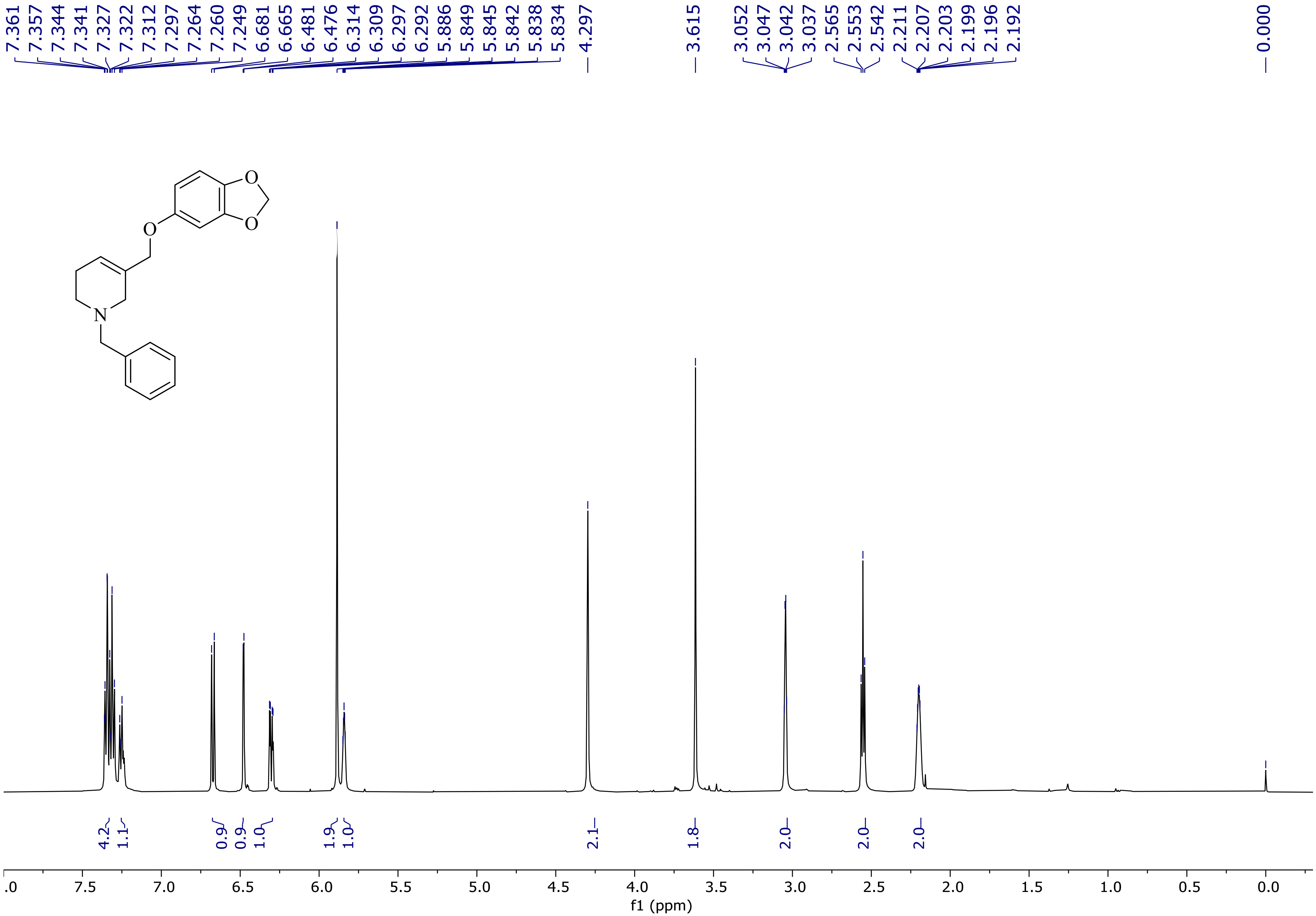

$^{13}C\{^{1}H\}$ NMR spectrum of compound 9 (125 MHz, $CDCl_3$):

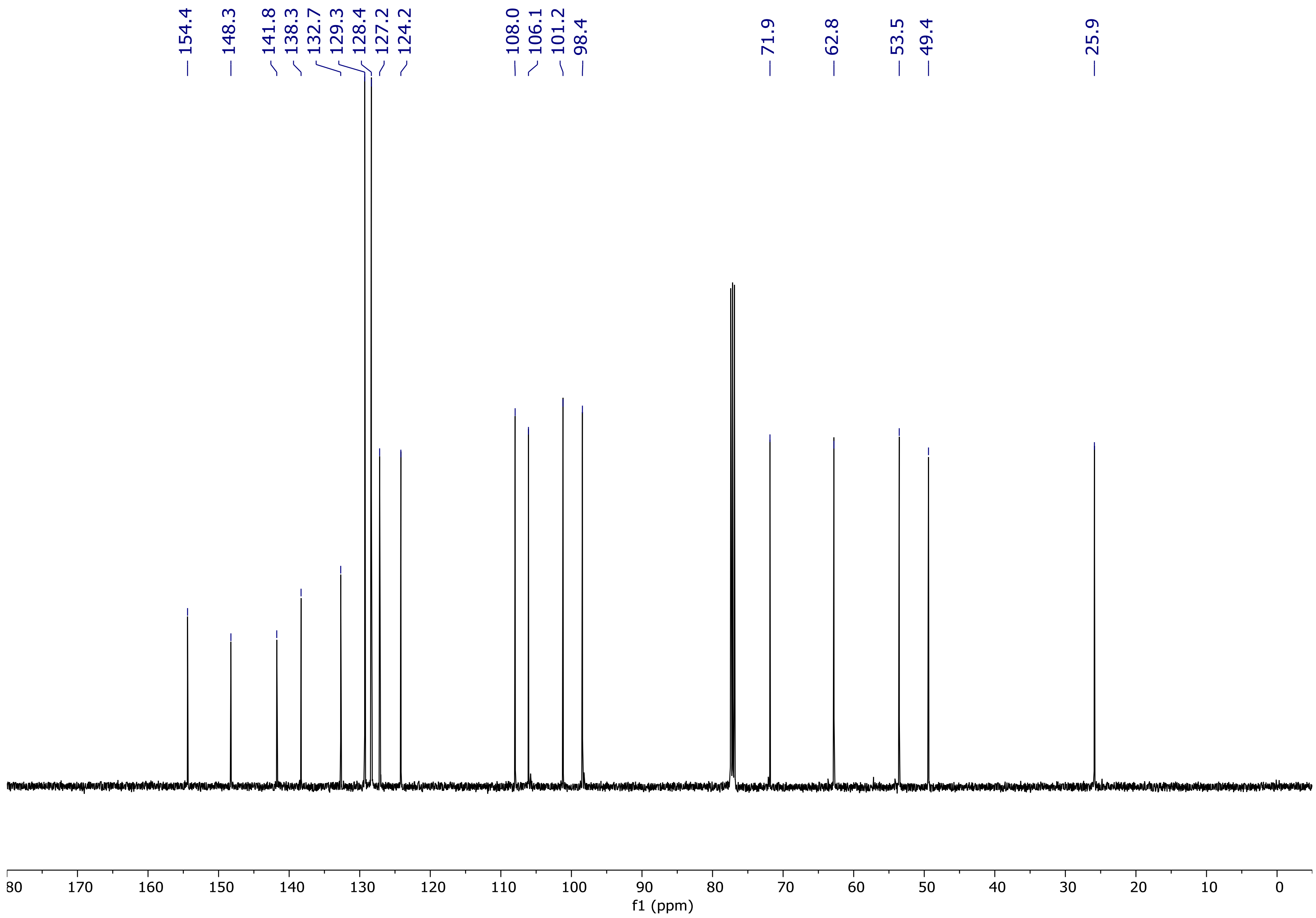

^{1}H NMR spectrum of compound 12a (500 MHz, $CDCl_3$):

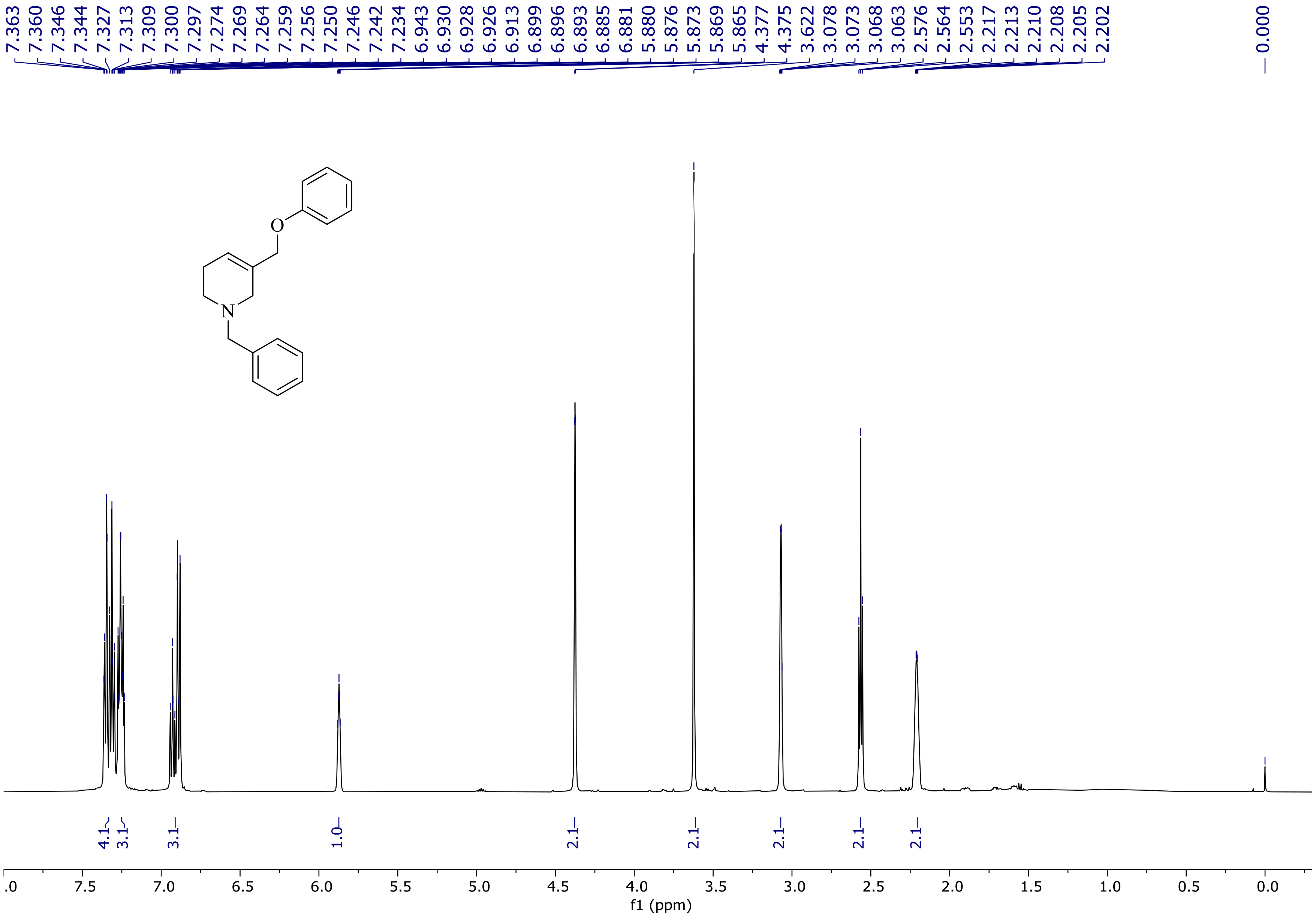

$^{13}C\{^1H\}$ NMR spectrum of compound 12a (125 MHz, $CDCl_3$):

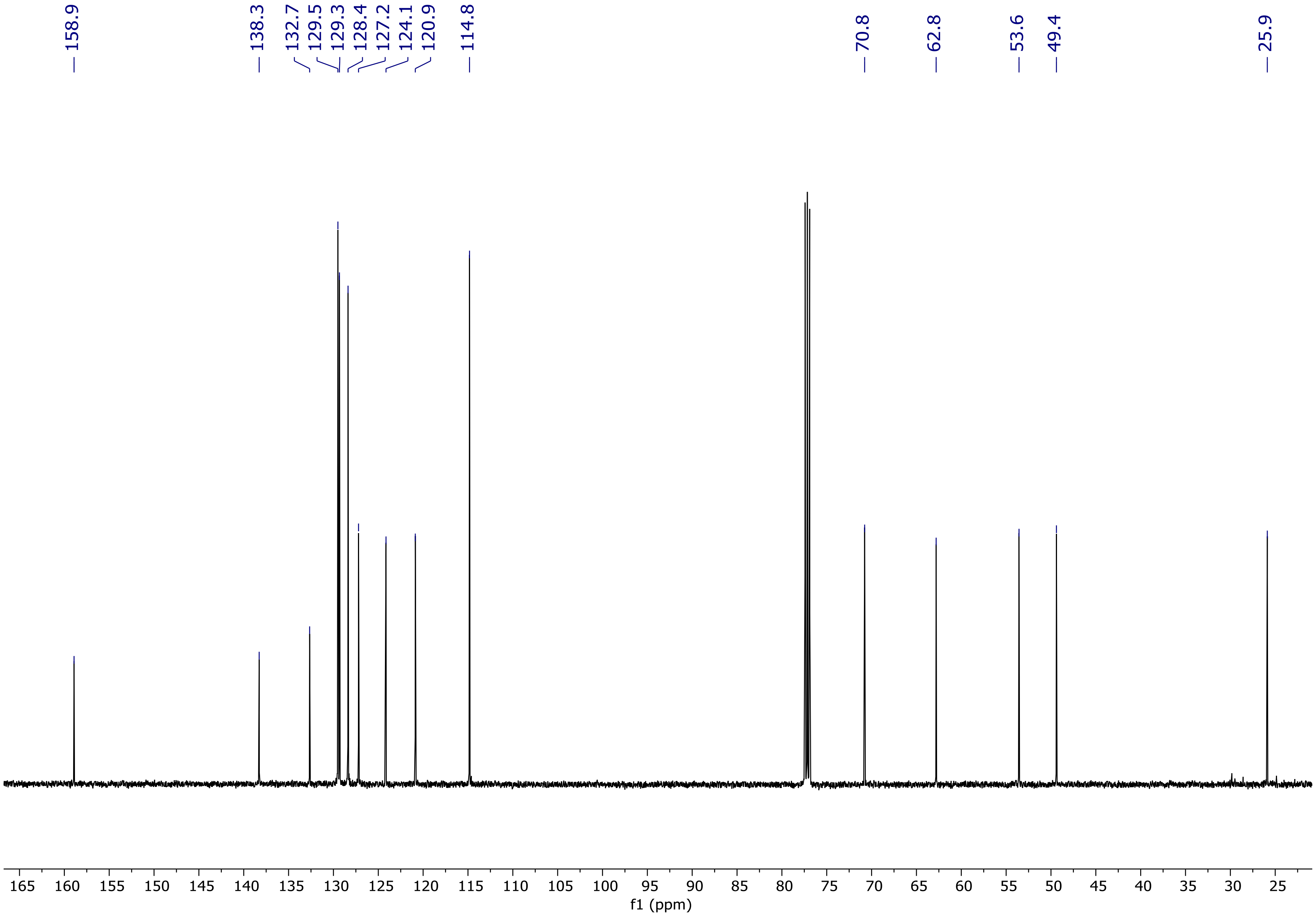

^{1}H NMR spectrum of compound 12b (500 MHz, $CDCl_3$):

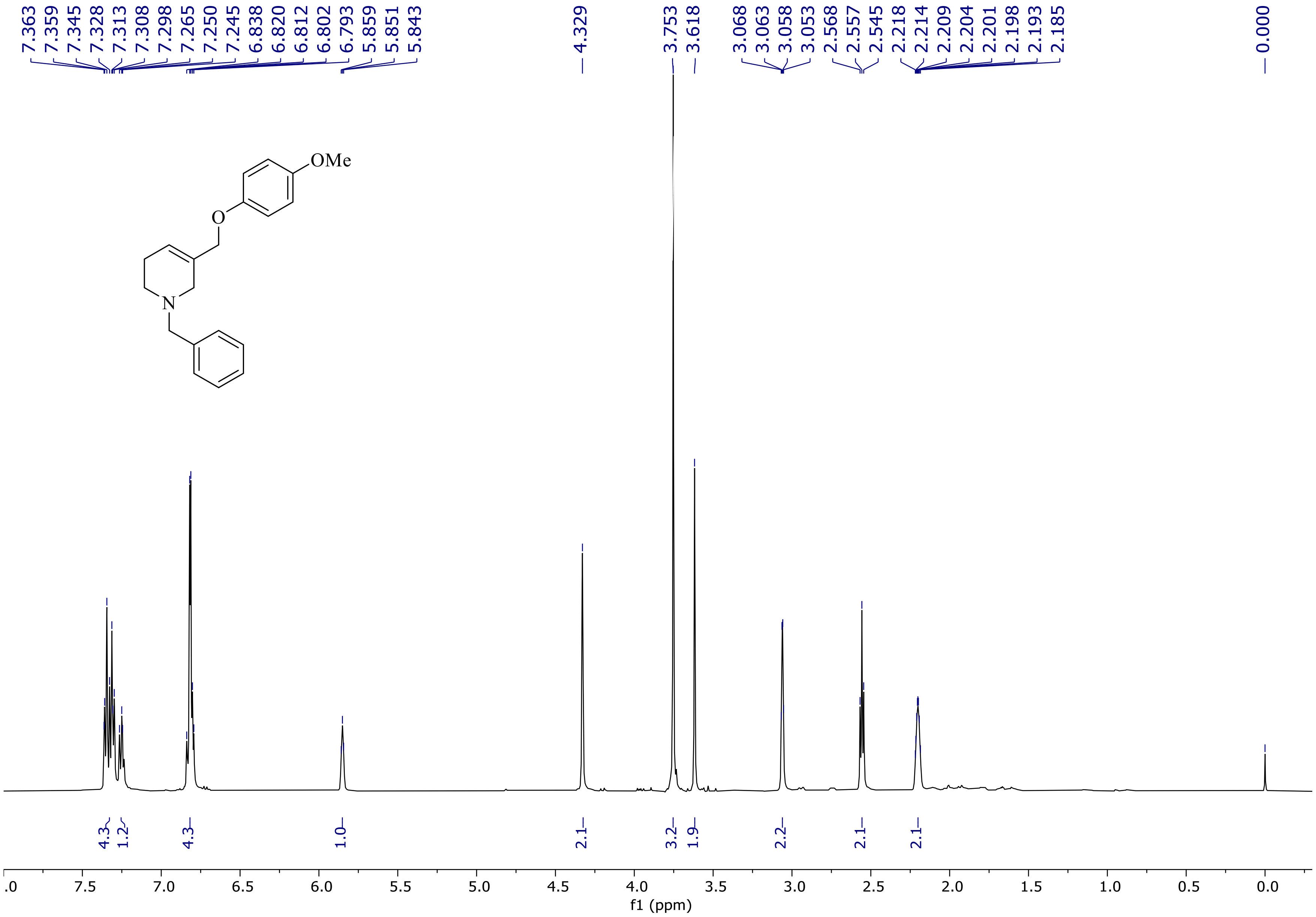

$^{13}C\{^1H\}$ NMR spectrum of compound 12b (125 MHz, $CDCl_3$):

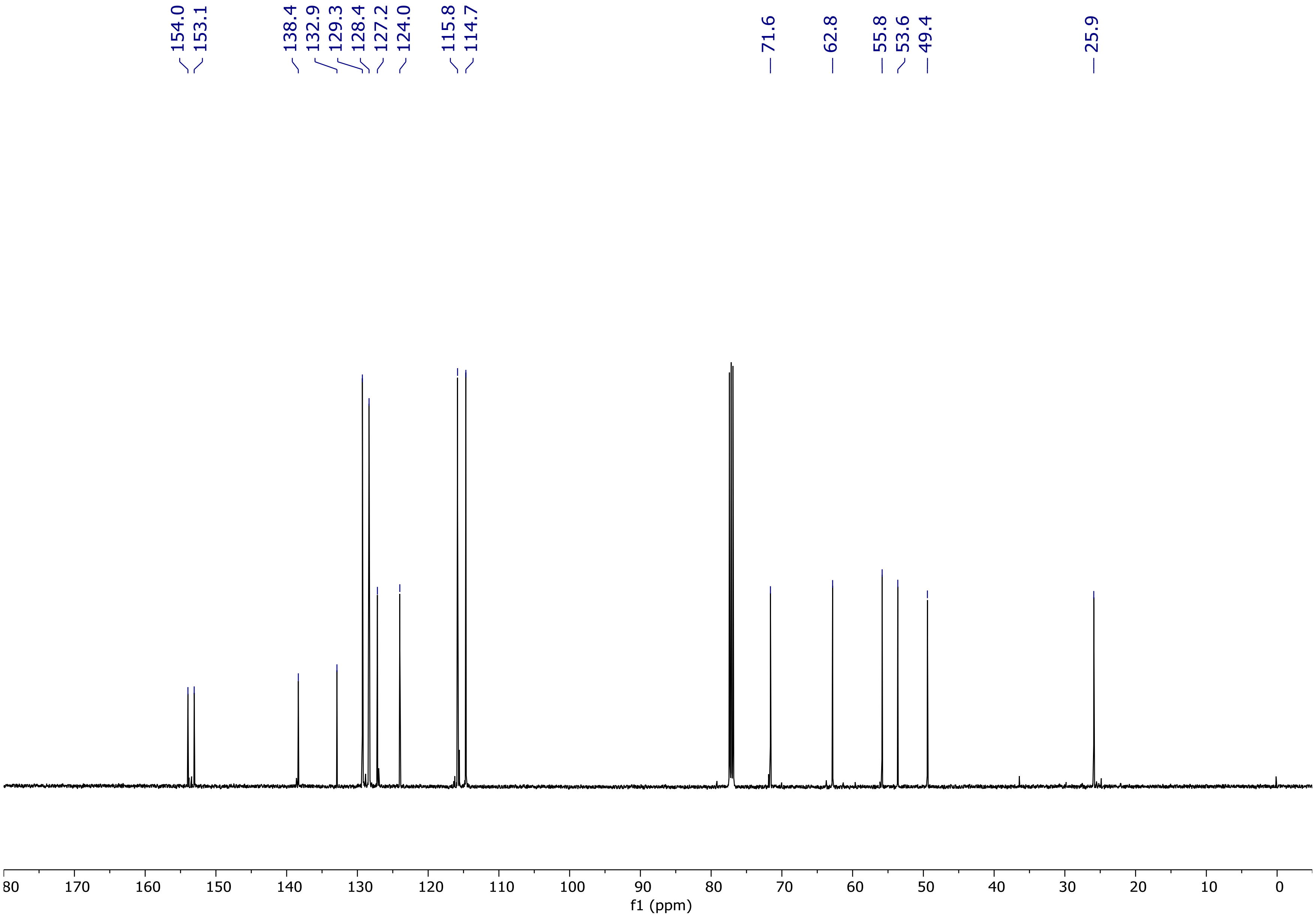

^{1}H NMR spectrum of compound 12c (500 MHz, $CDCl_3$):

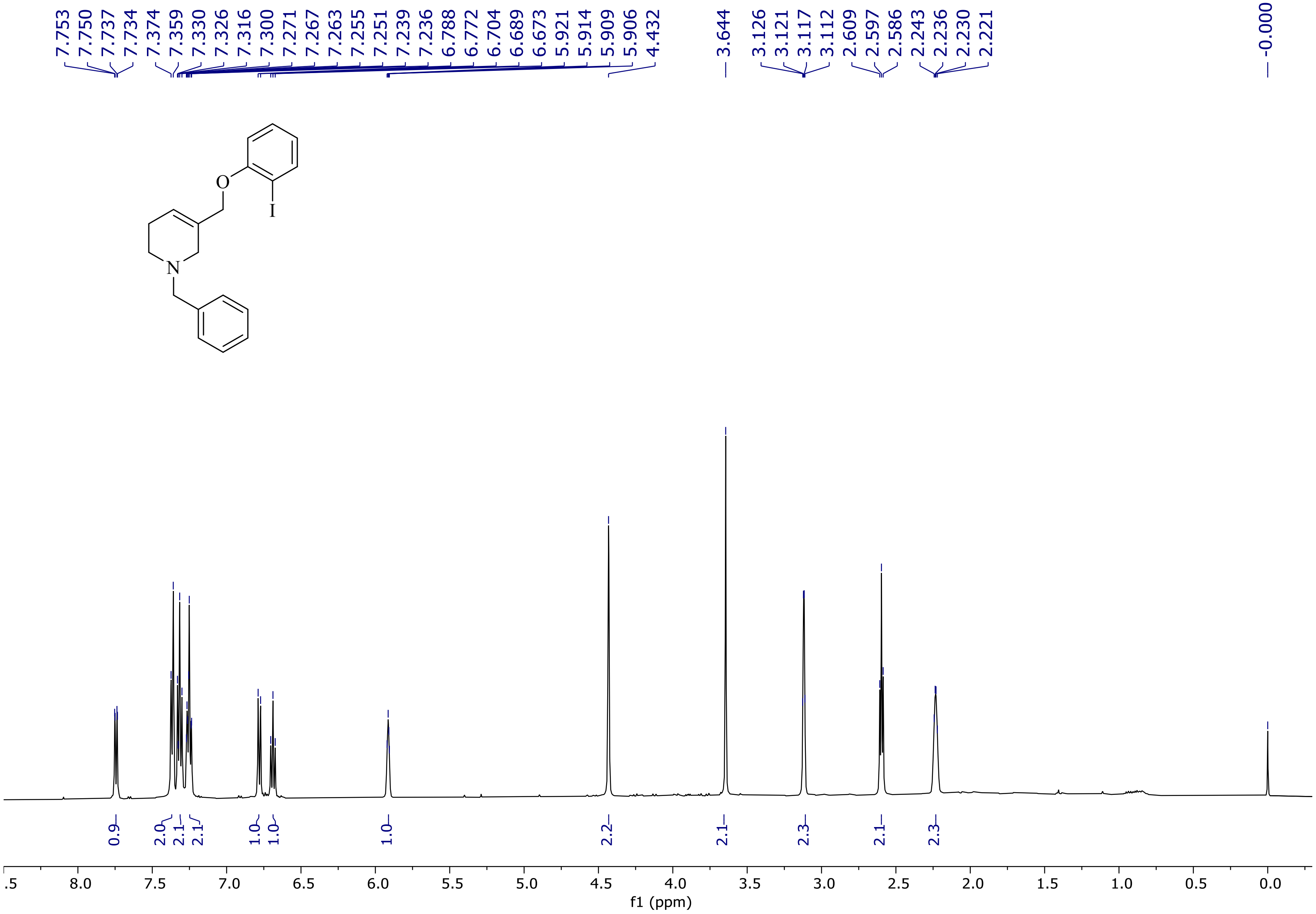

$^{13}C\{^{1}H\}$ **NMR spectrum of compound 12c (125 MHz, $CDCl_3$):**

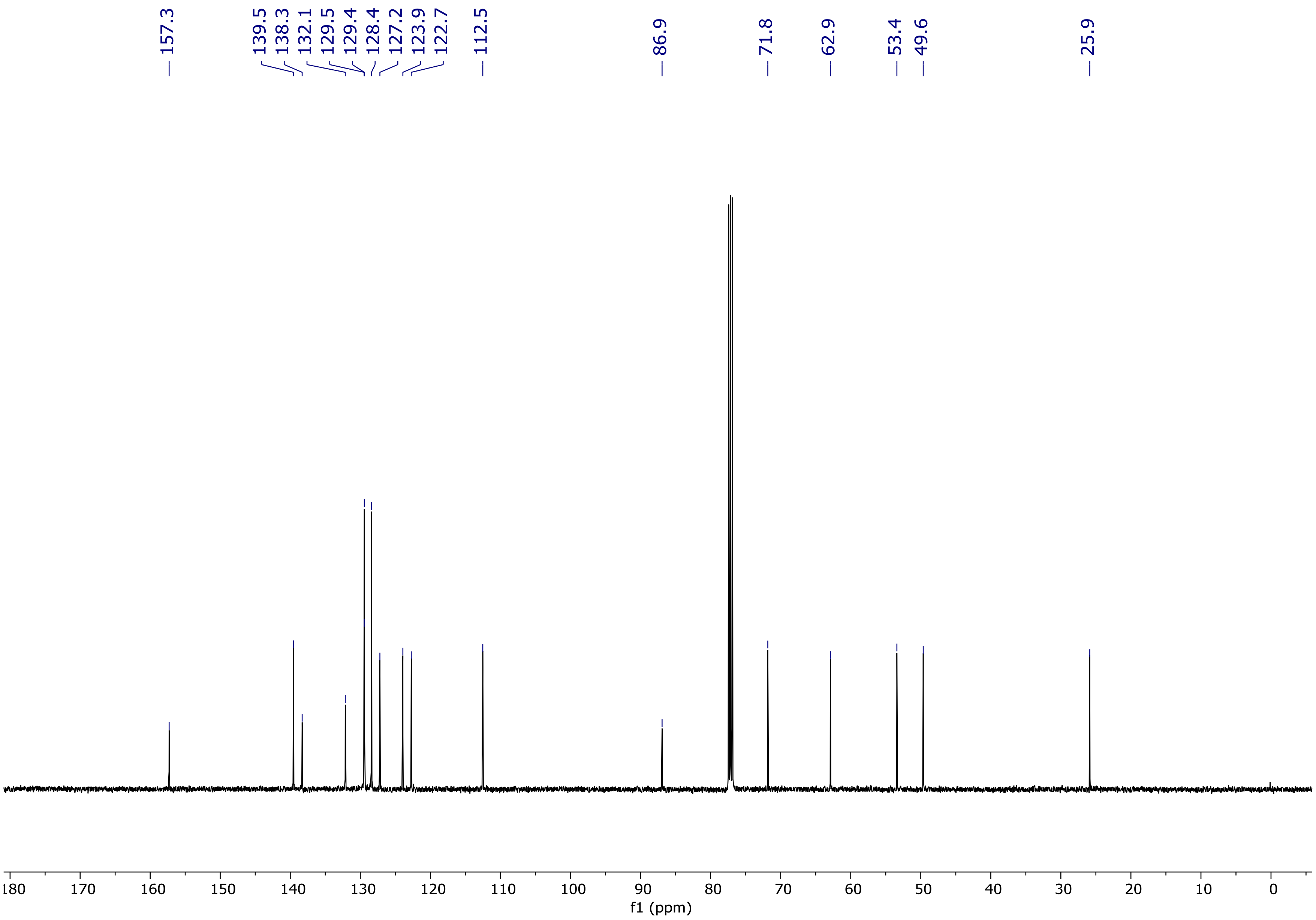

^{1}H NMR spectrum of compound 12d (500 MHz, $CDCl_3$):

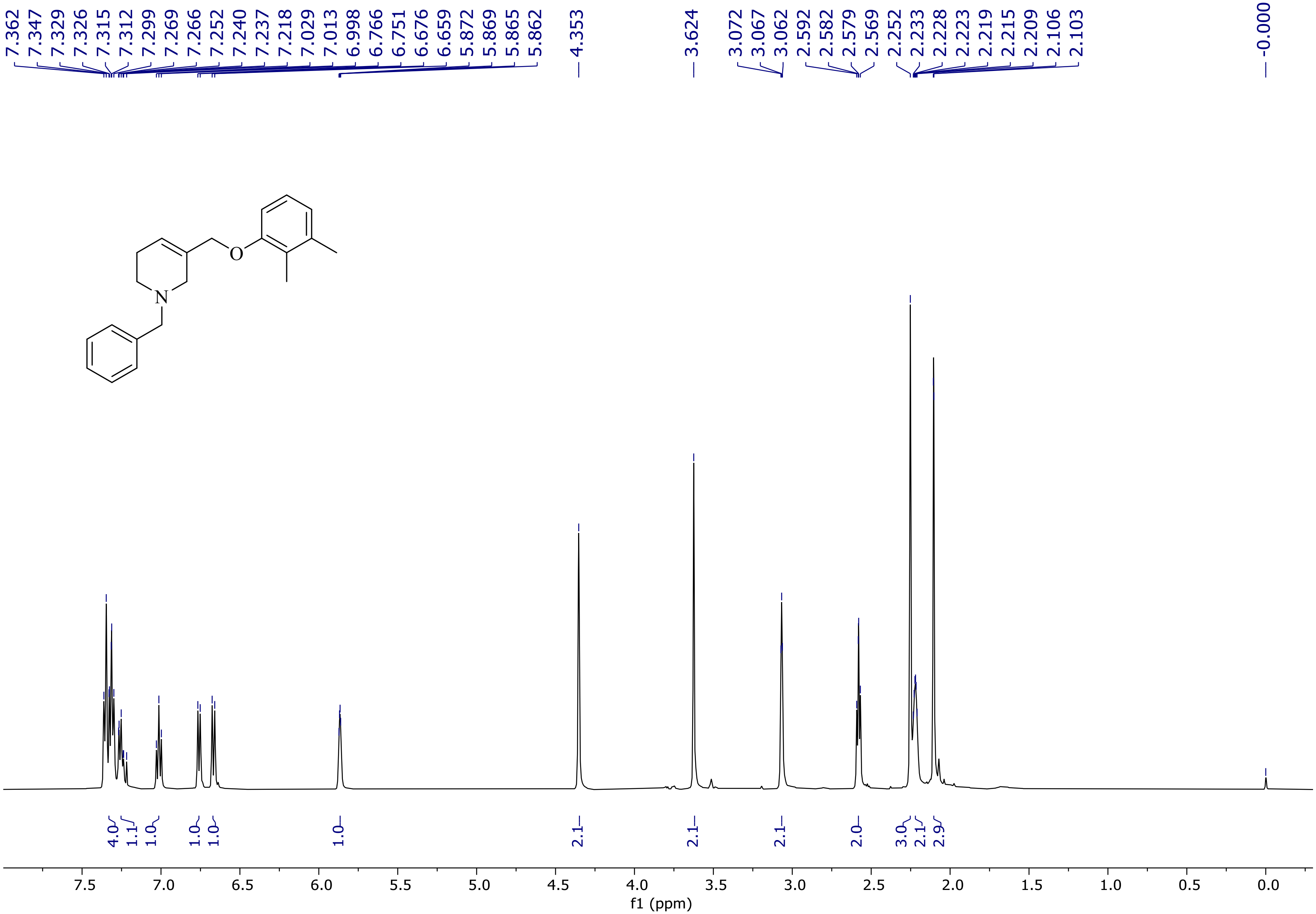

$^{13}C\{^{1}H\}$ NMR spectrum of compound 12d (125 MHz, $CDCl_3$):

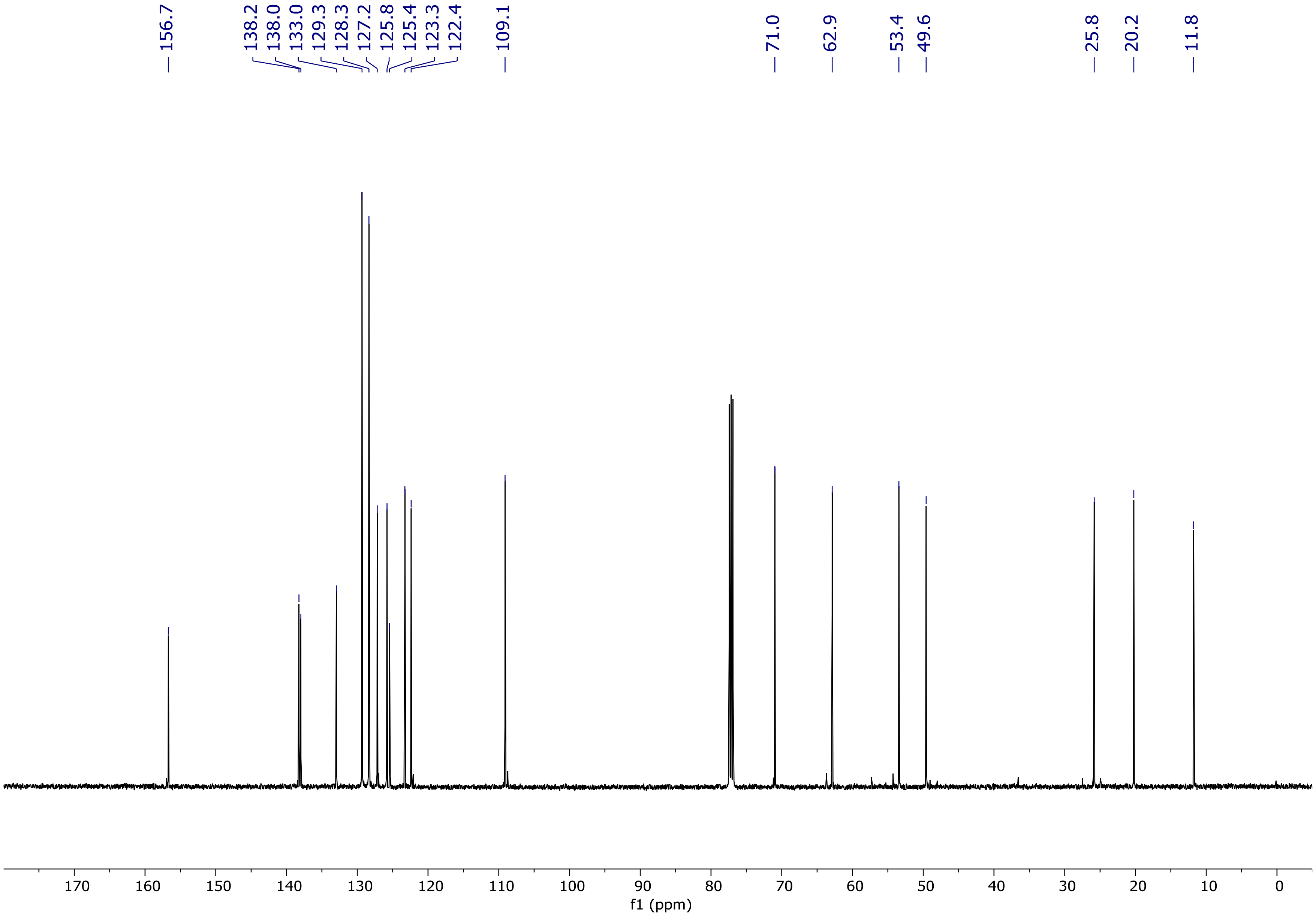

^{1}H NMR spectrum of compound 12e (500 MHz, CDCl₃):

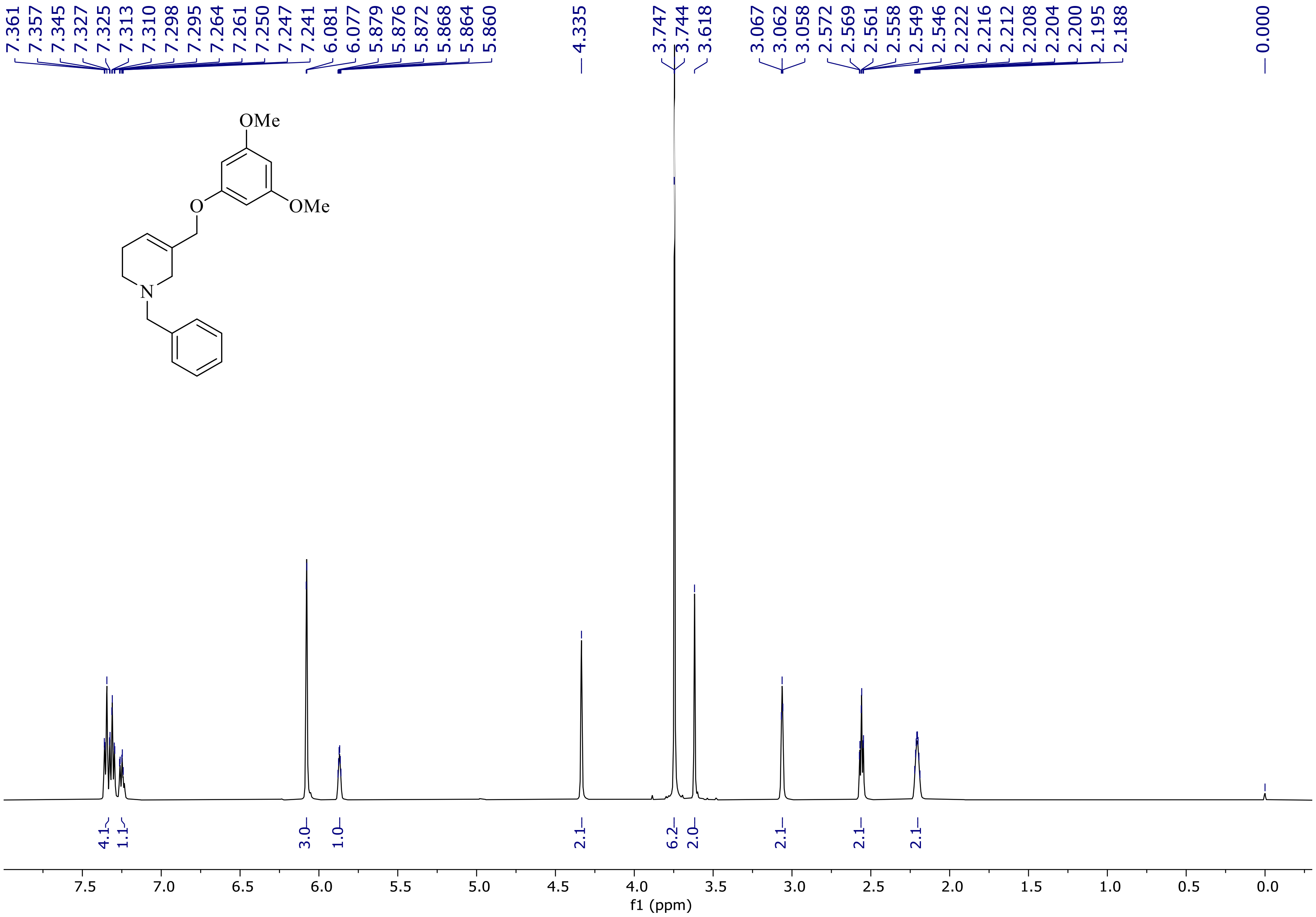

$^{13}C\{^1H\}$ NMR spectrum of compound 12e (125 MHz, $CDCl_3$):

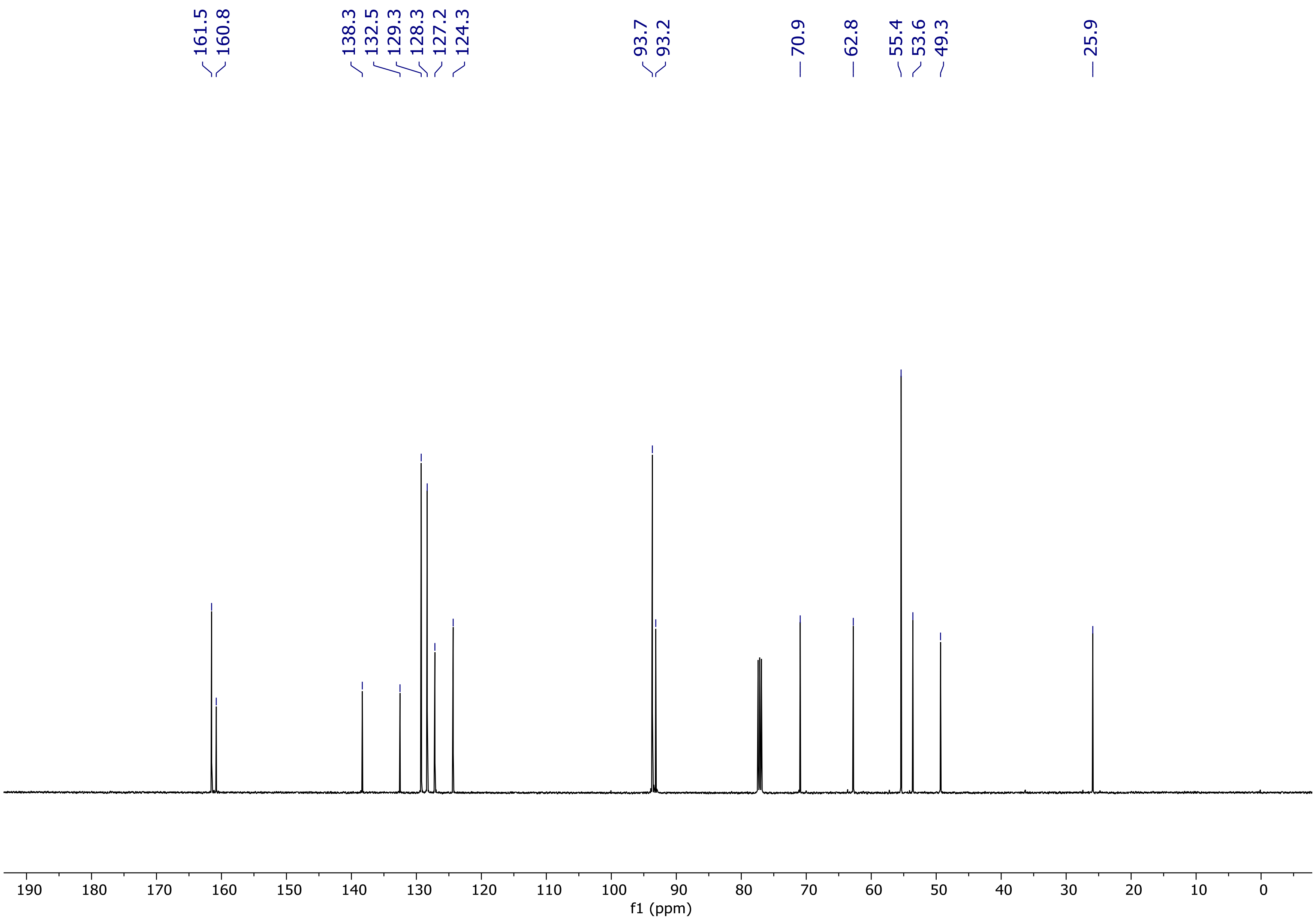

^{1}H NMR spectrum of compound 12f (500 MHz, CDCl3):

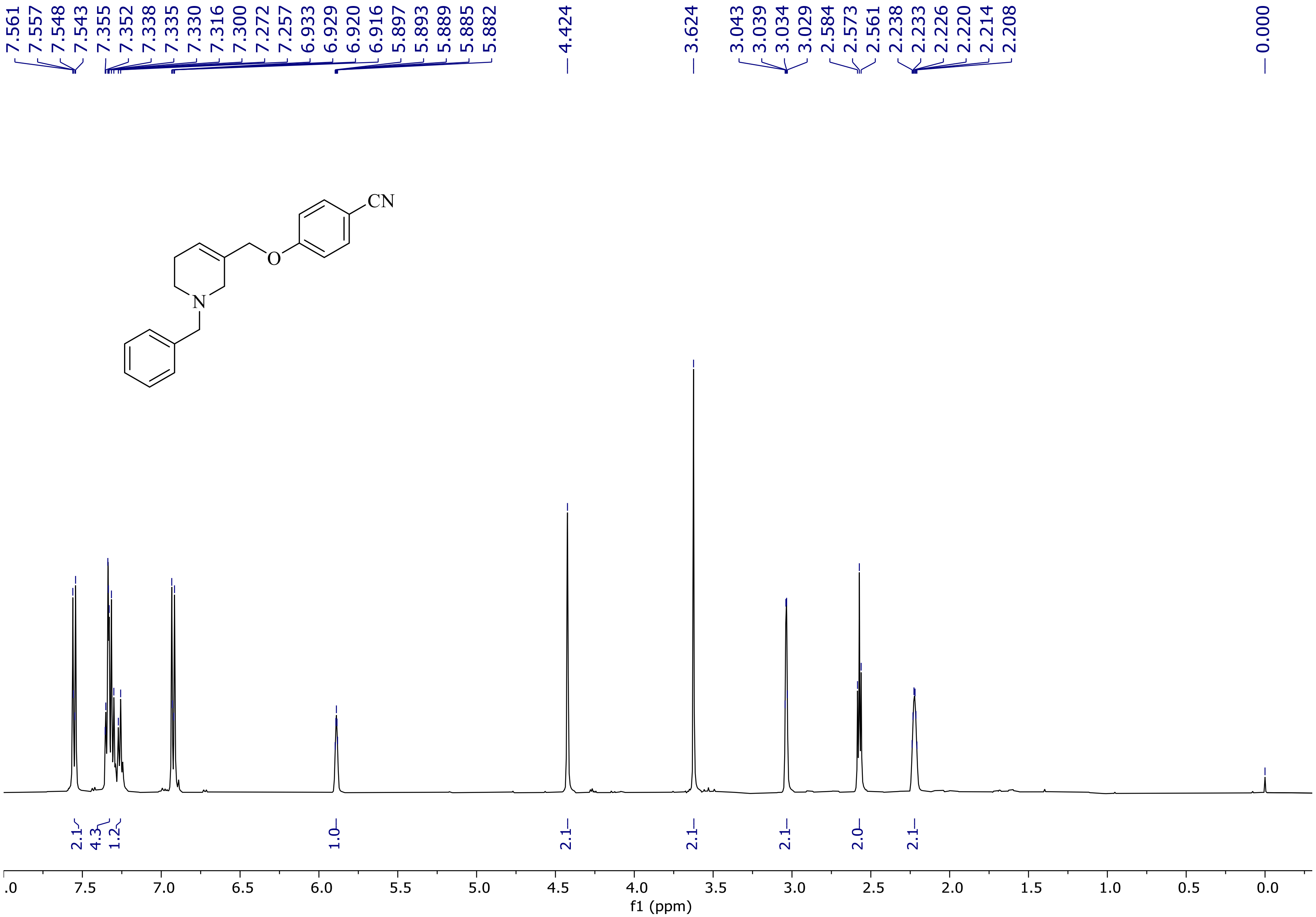

$^{13}C\{^1H\}$ NMR spectrum of compound 12f (125 MHz, $CDCl_3$):

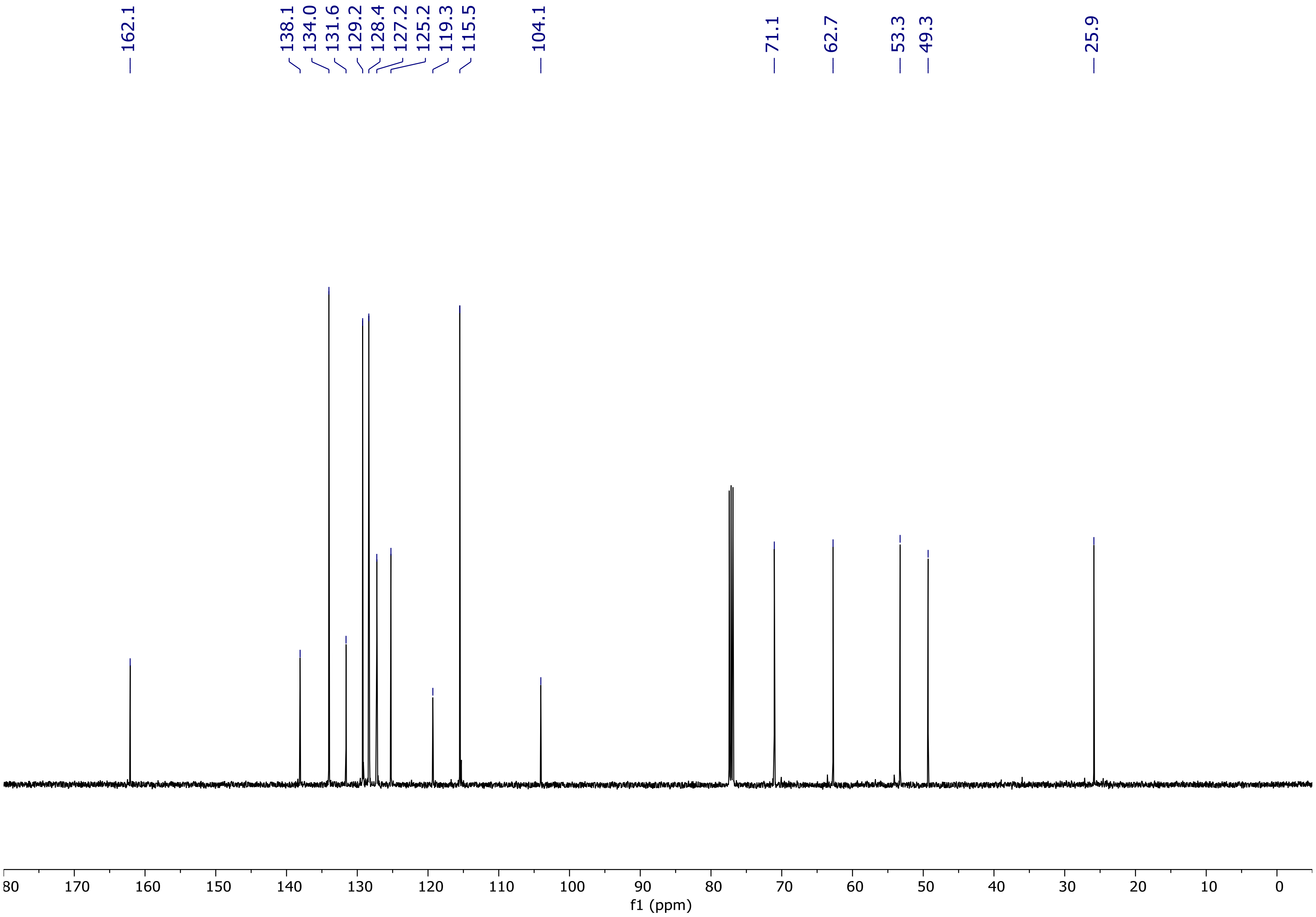

^{1}H NMR spectrum of compound 12g (500 MHz, $CDCl_3$):

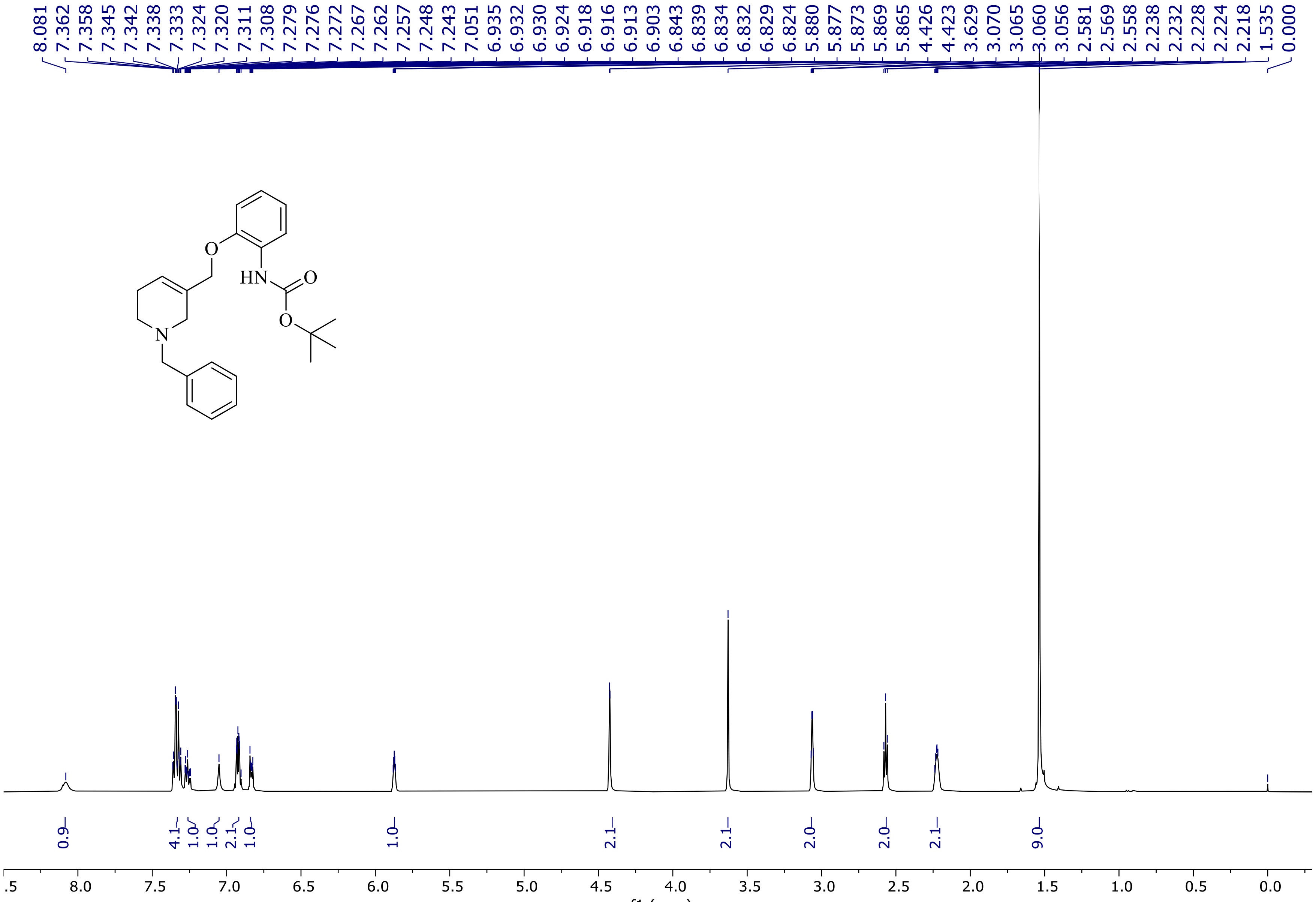

$^{13}C\{^1H\}$ NMR spectrum of compound 12g (125 MHz, $CDCl_3$):

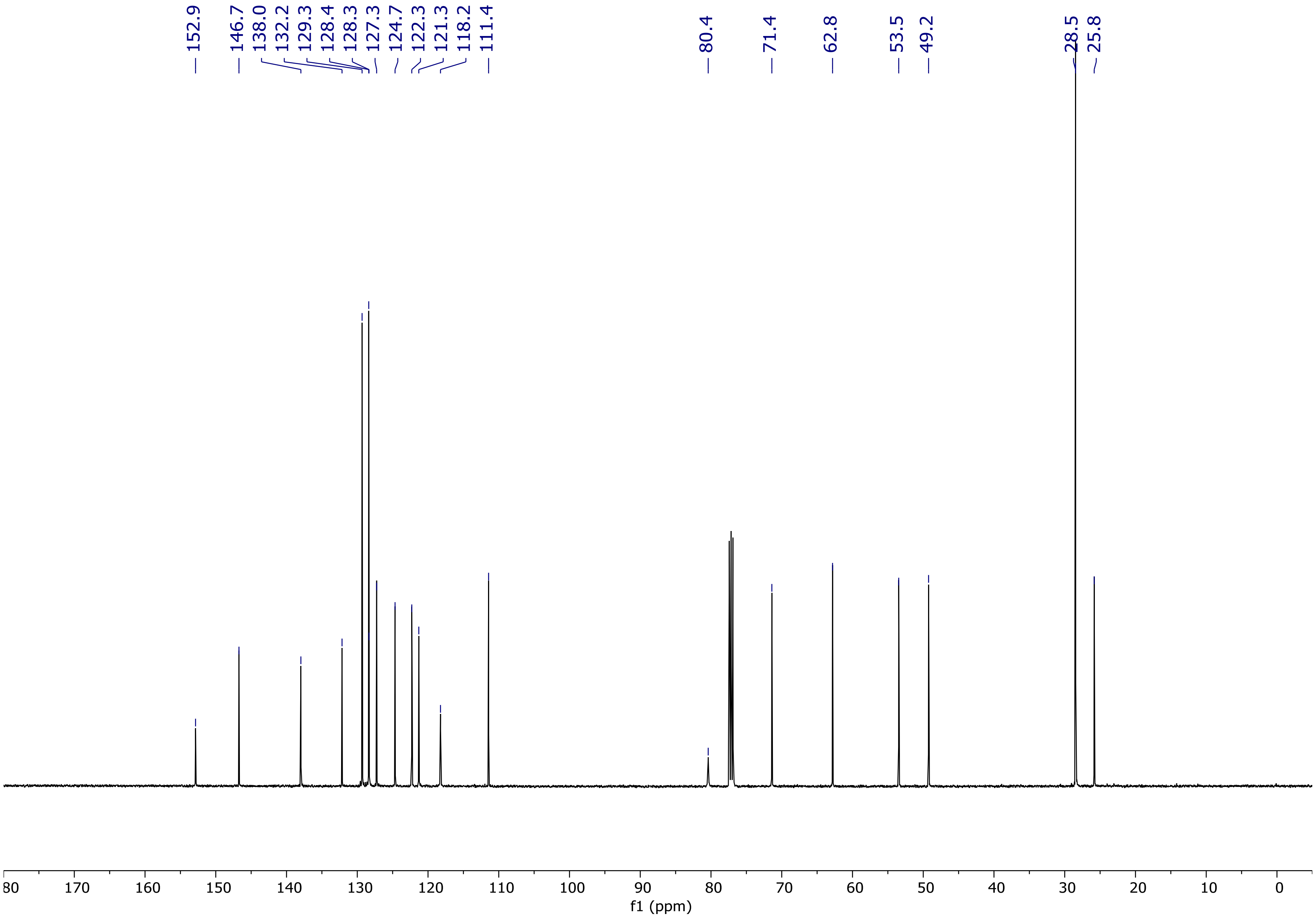

^{1}H NMR spectrum of compound 12h (500 MHz, $CDCl_3$):

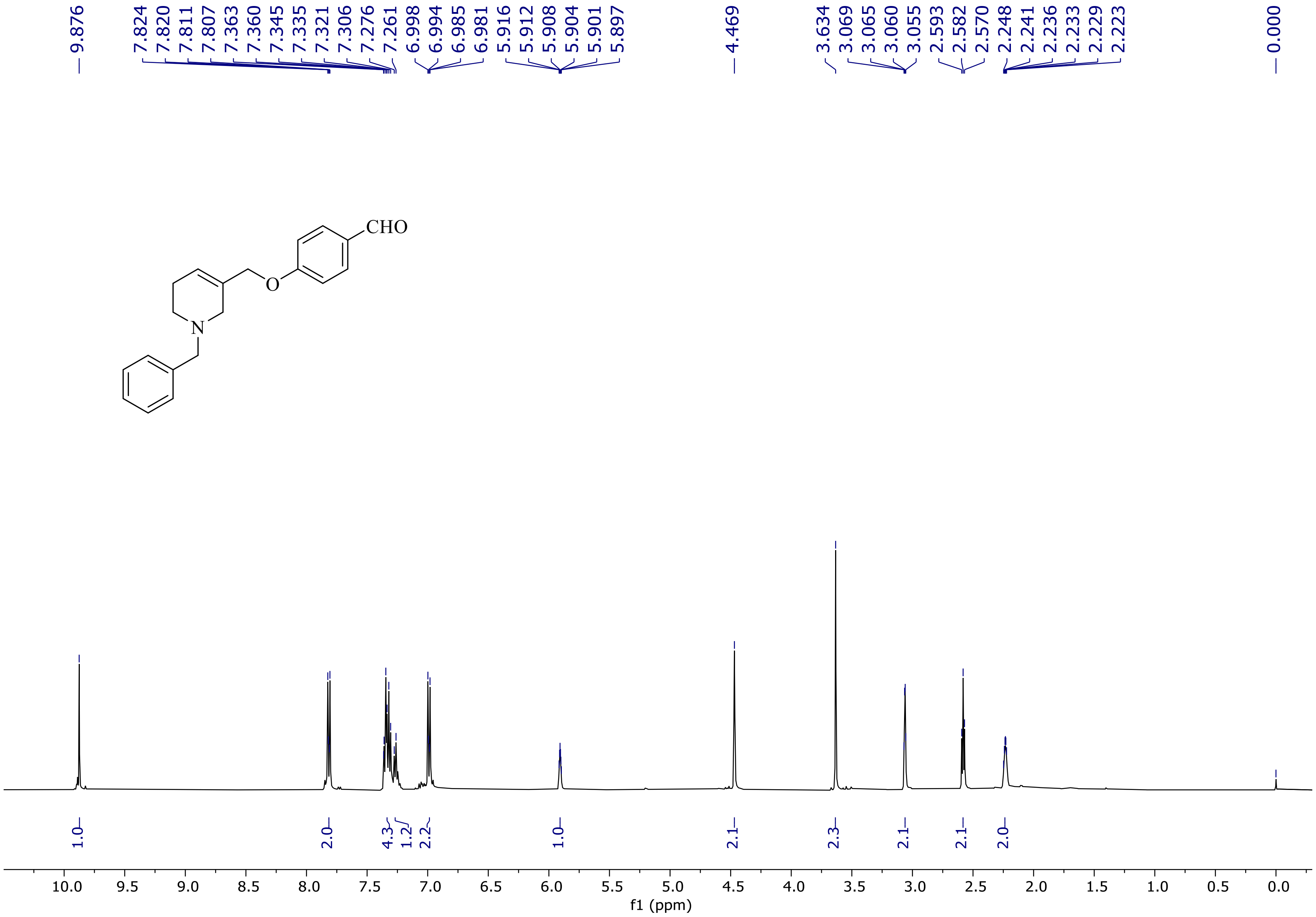

$^{13}C\{^{1}H\}$ NMR spectrum of compound 12h (125 MHz, $CDCl_3$):

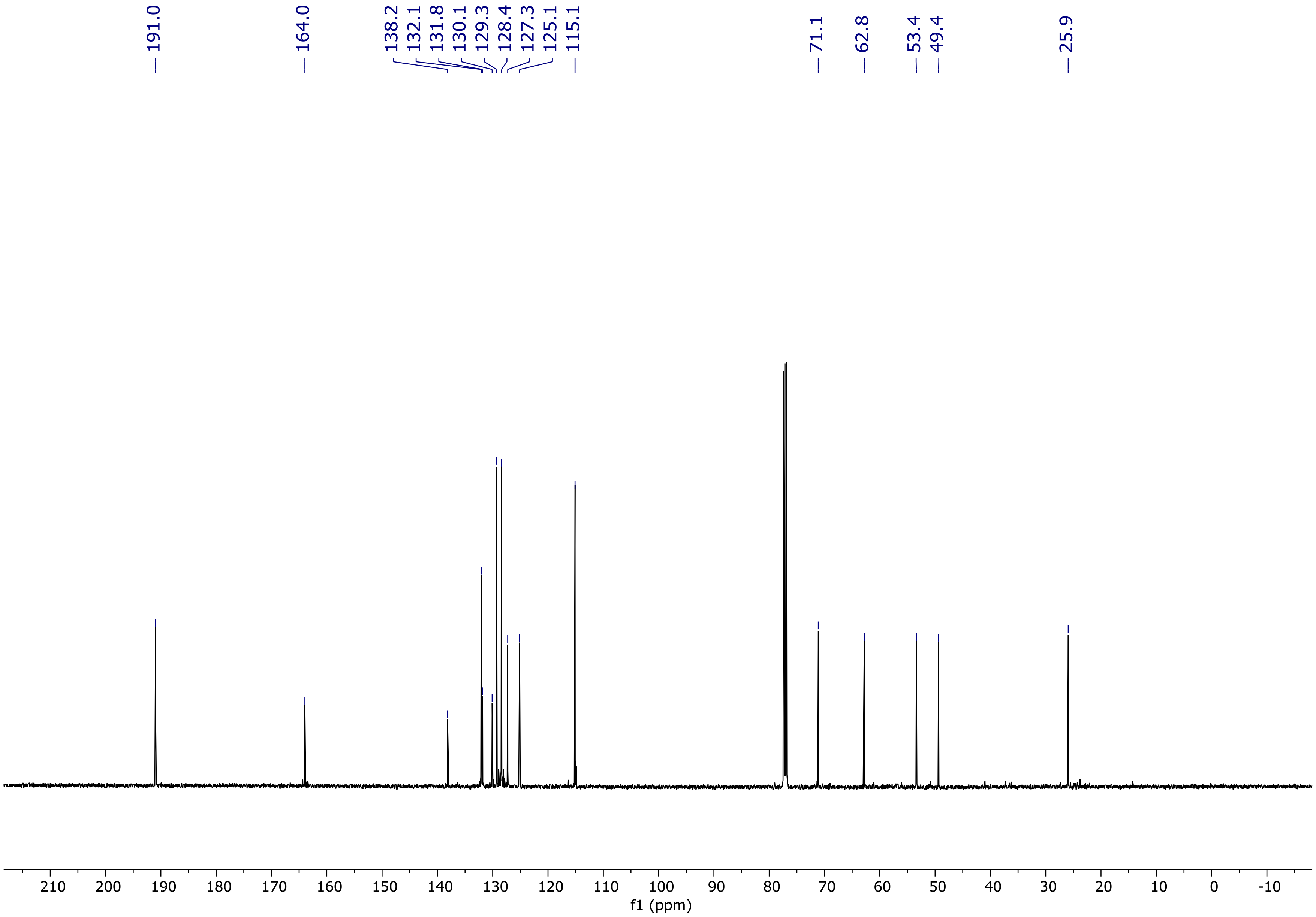

^{1}H NMR spectrum of compound 12i (500 MHz, $CDCl_3$):

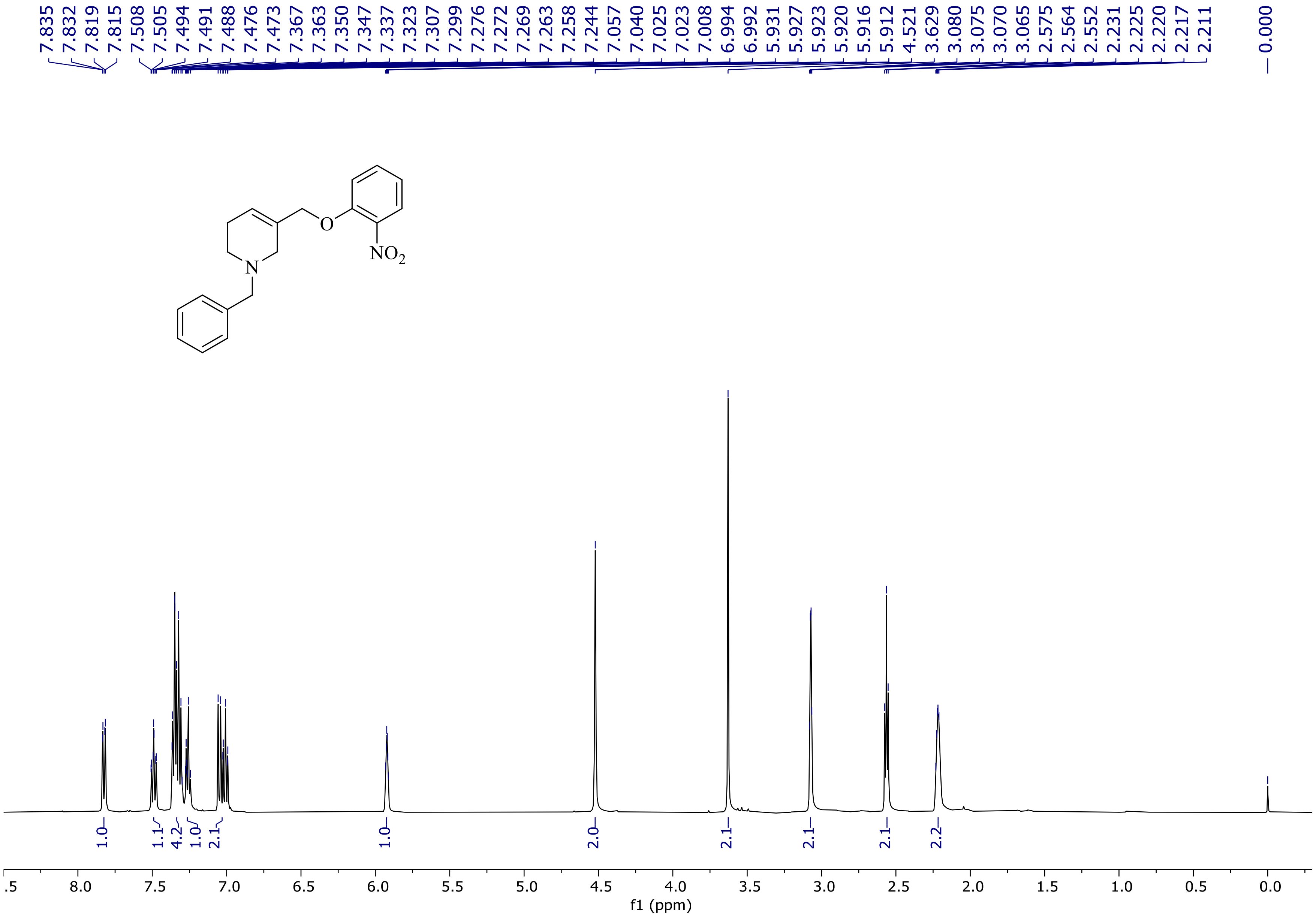

$^{13}C\{^1H\}$ NMR spectrum of compound 12i (125 MHz, $CDCl_3$):

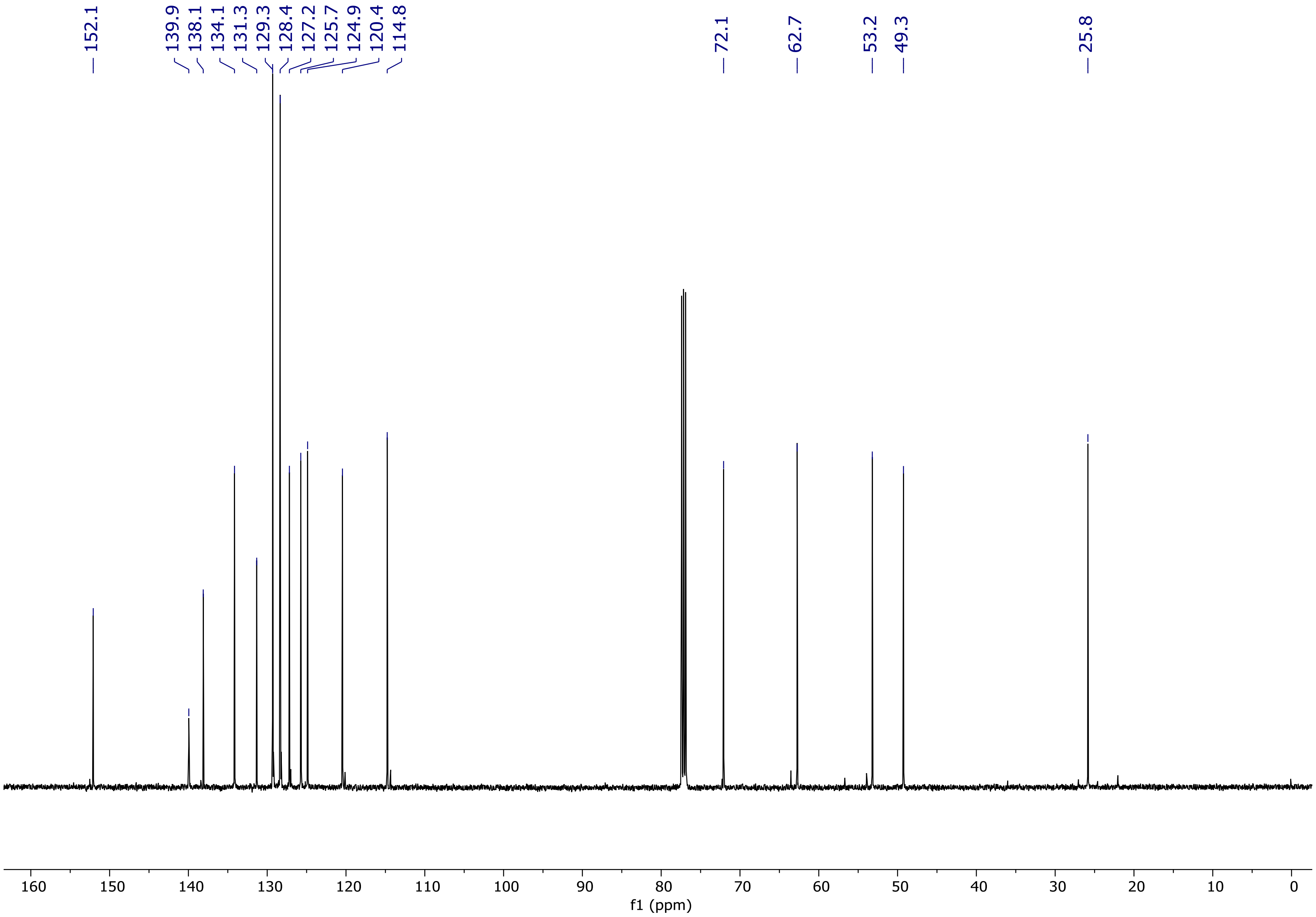

^{1}H NMR spectrum of compound 12j (500 MHz, $CDCl_3$):

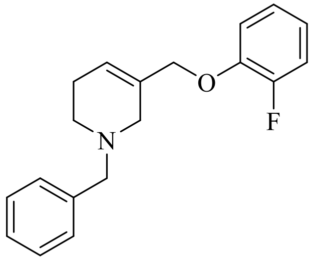

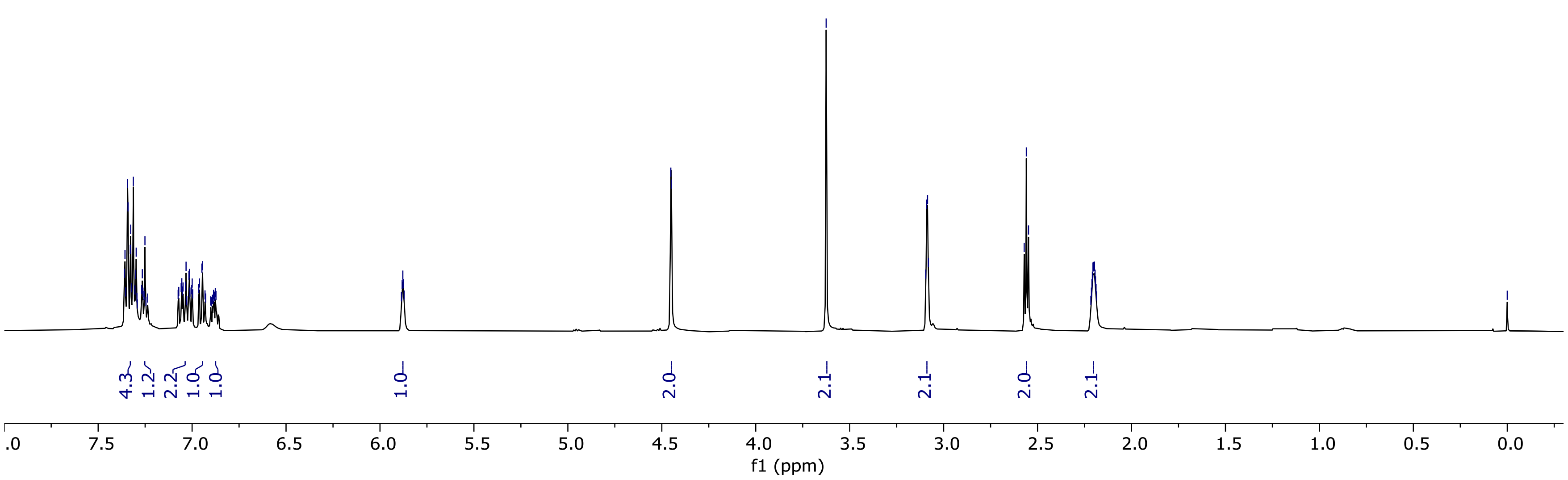

$^{13}C\{^{1}H\}$ NMR spectrum of compound 12j (125 MHz, $CDCl_3$):

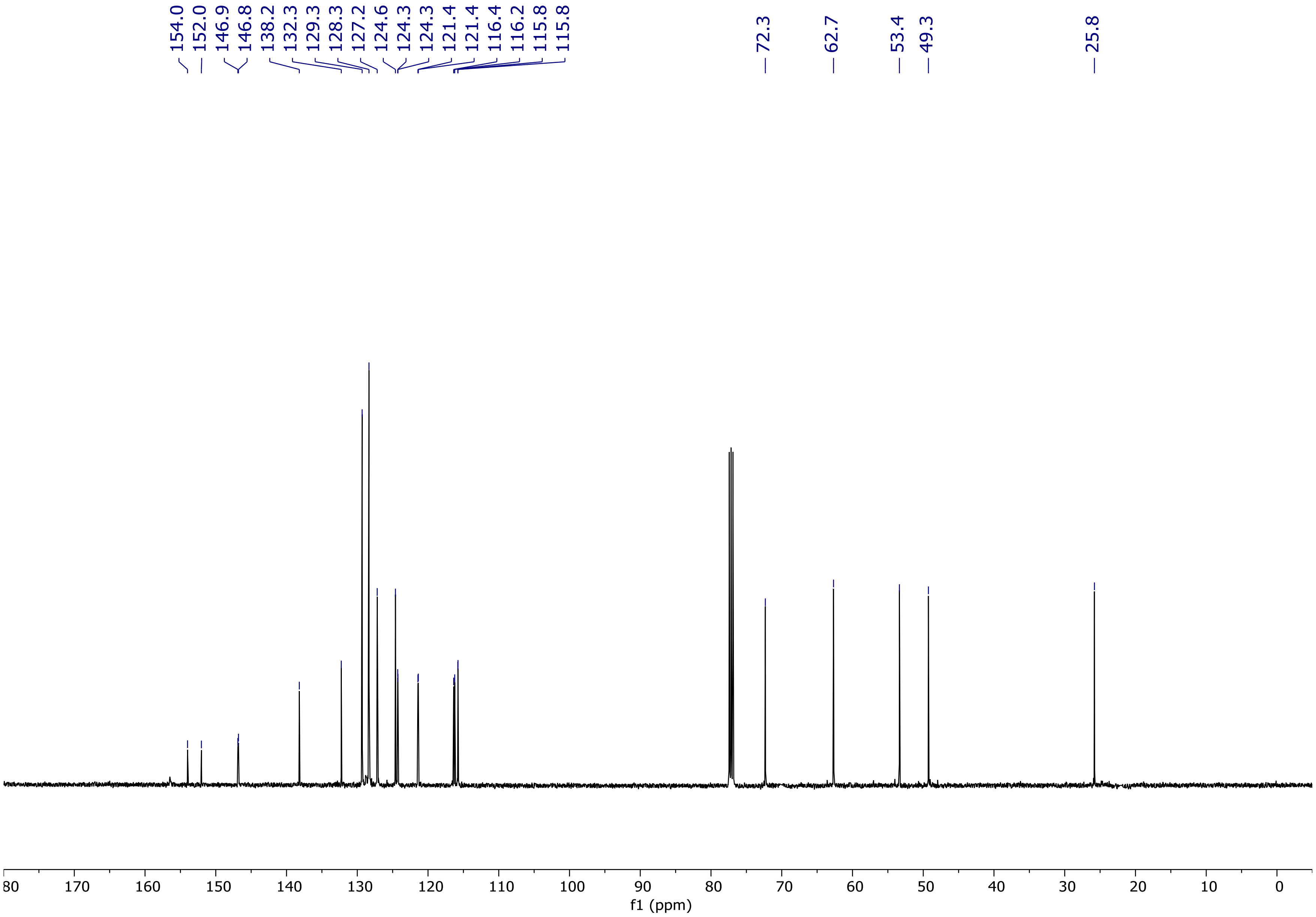

^{1}H NMR spectrum of compound 12k (500 MHz, $CDCl_3$):

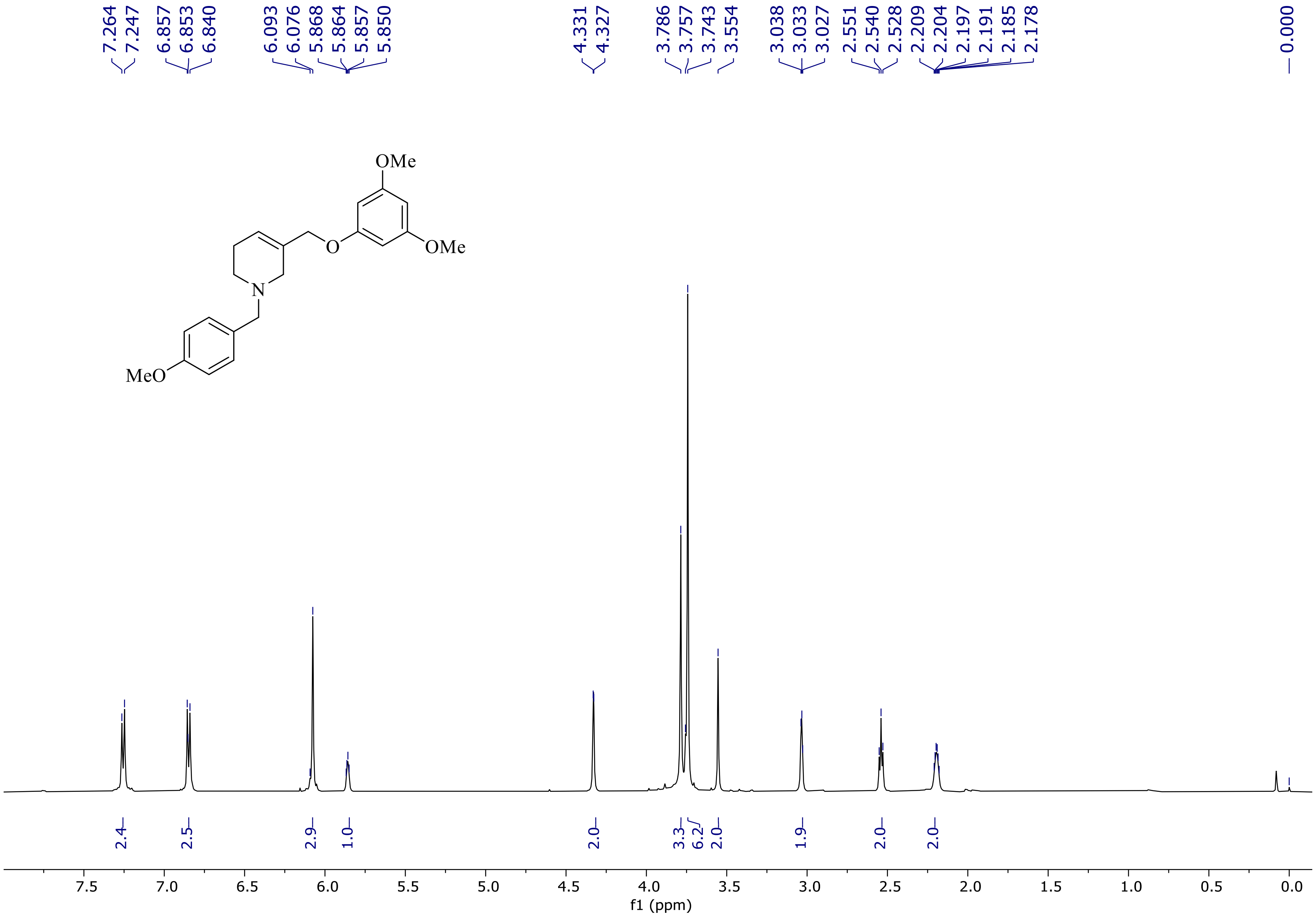

$^{13}C\{^{1}H\}$ NMR spectrum of compound 12k (125 MHz, $CDCl_3$):

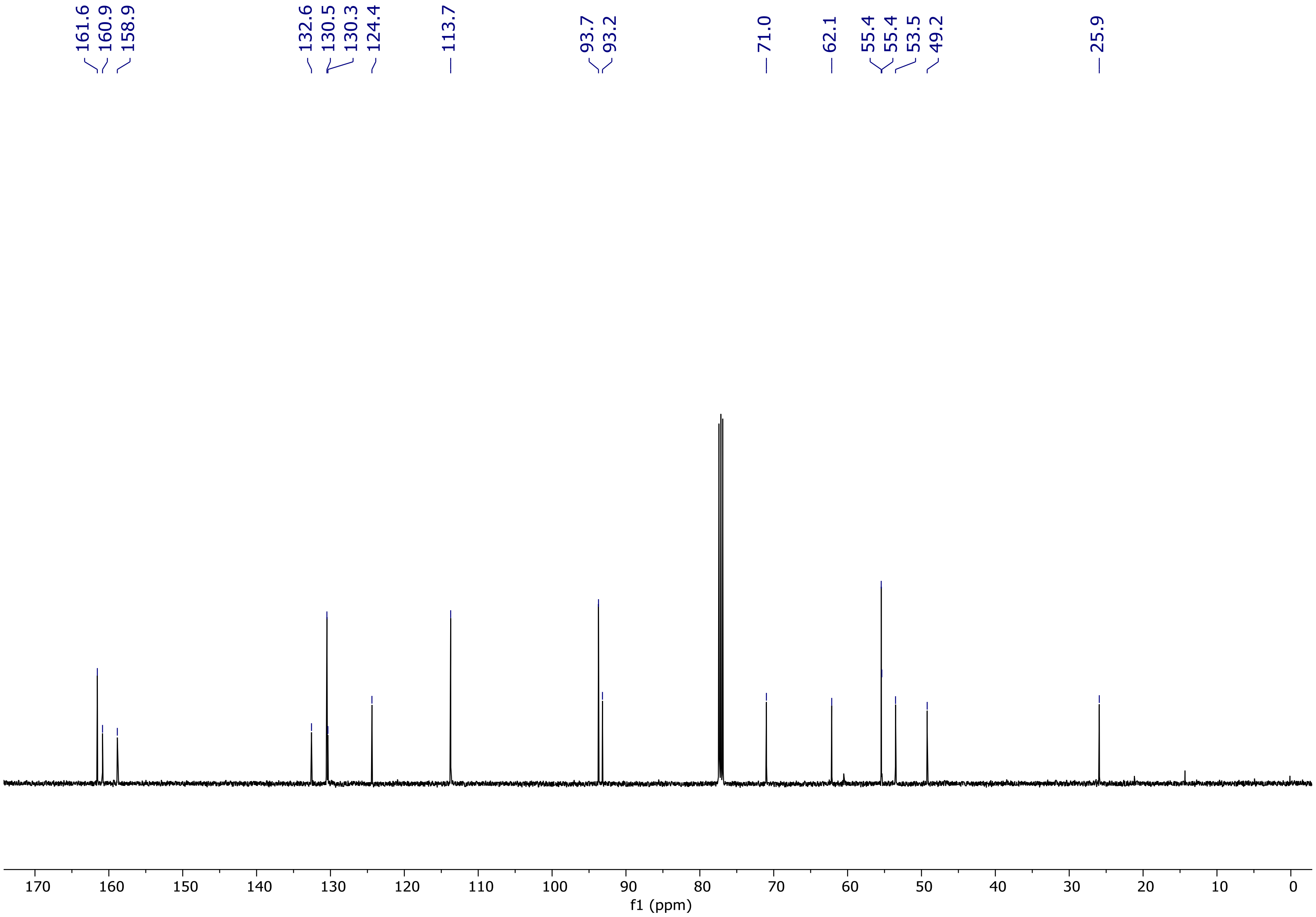

^{1}H NMR spectrum of compound S2 (500 MHz, $CDCl_3$):

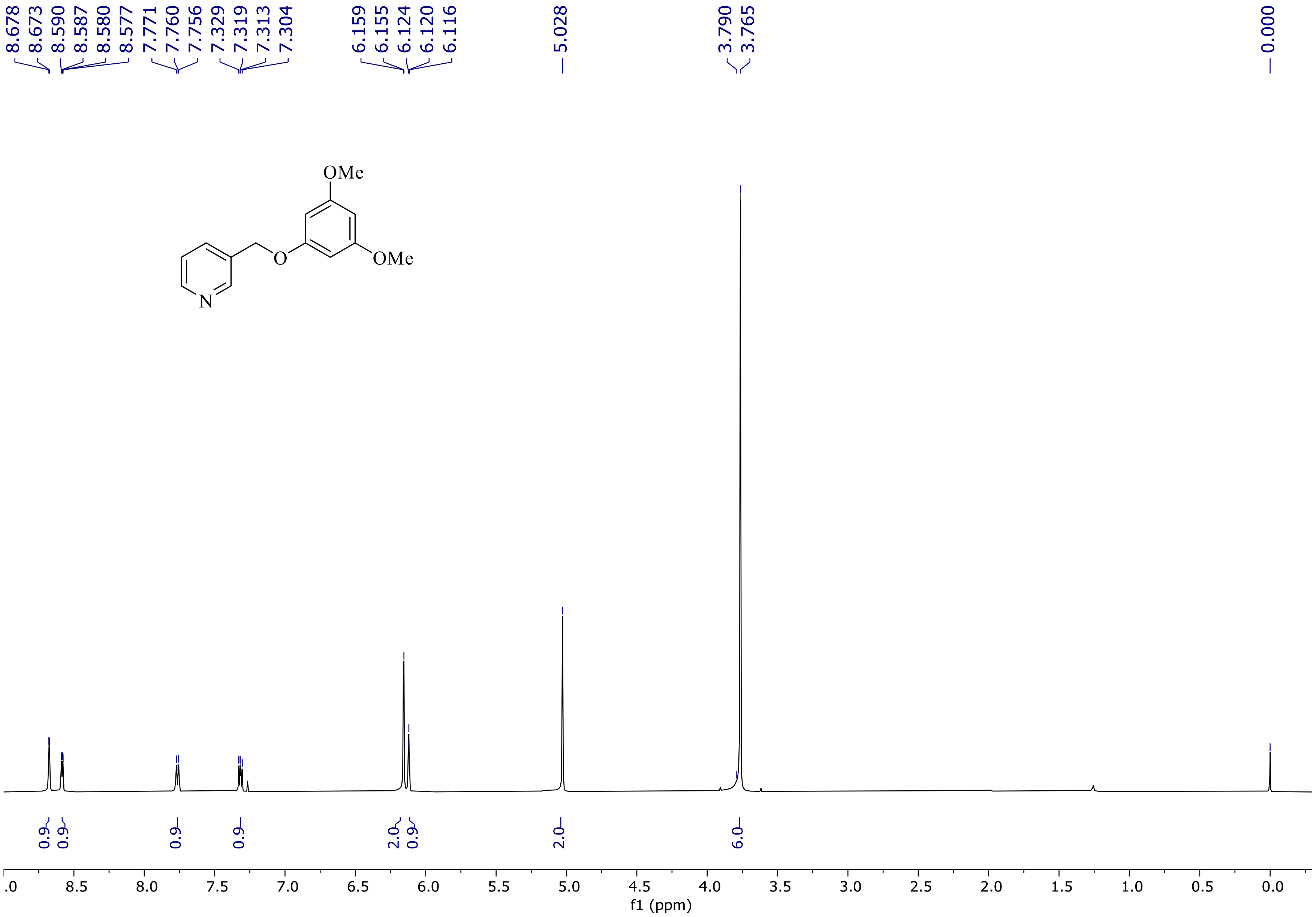

$^{13}C\{^1H\}$ NMR spectrum of compound S2 (125 MHz, $CDCl_3$):

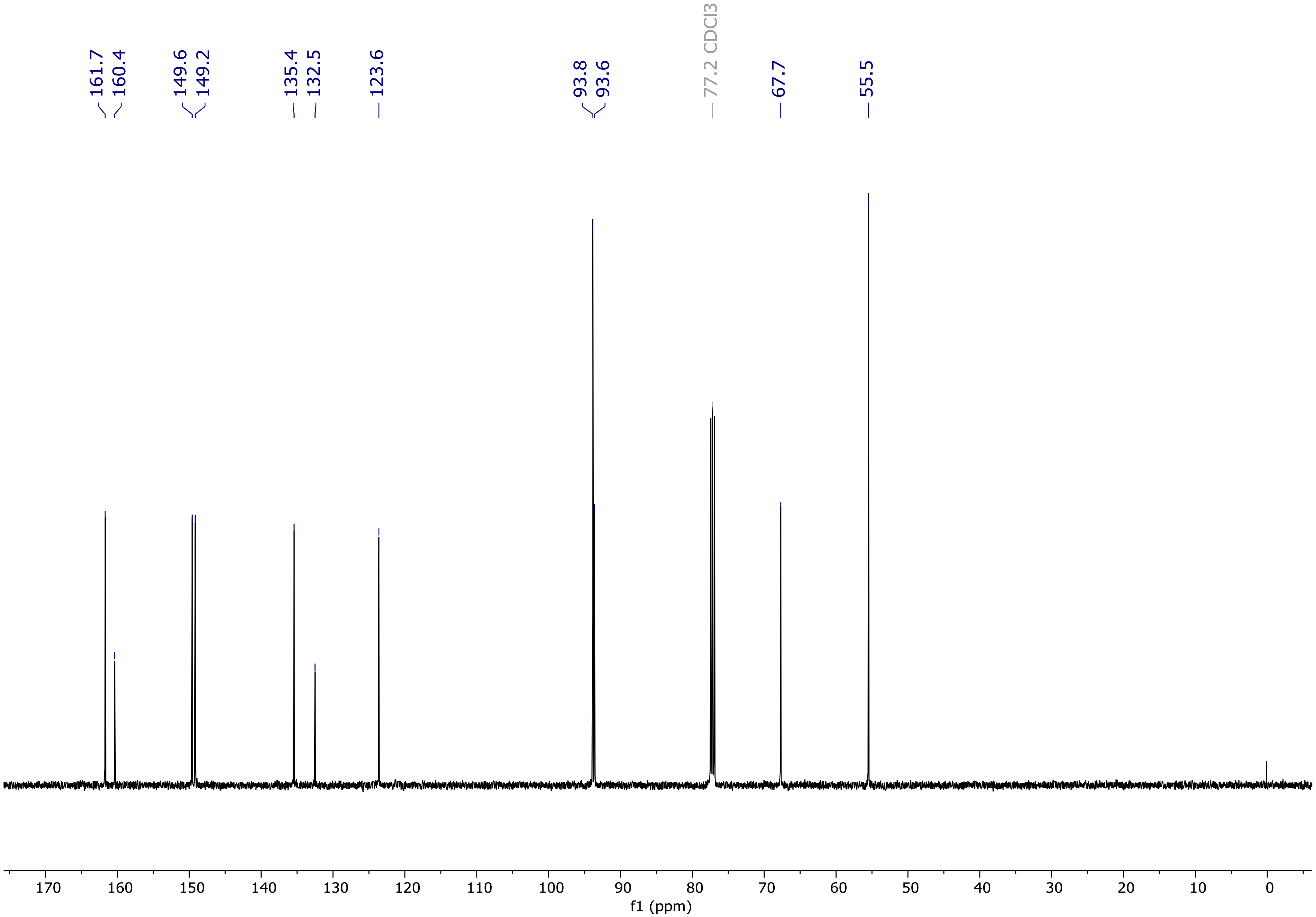

^{1}H NMR spectrum of compound 12l (500 MHz, $CDCl_3$):

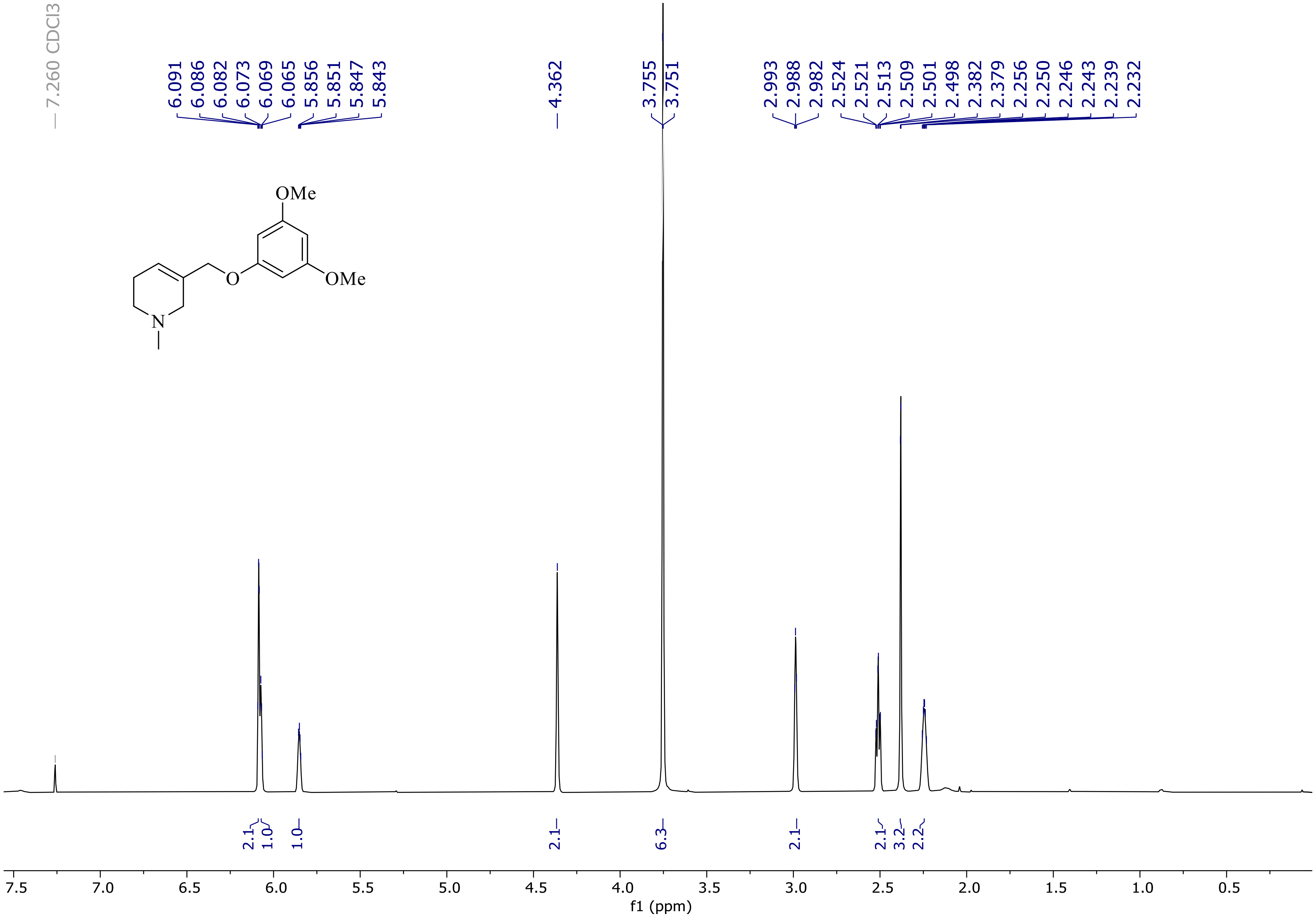

$^{13}C\{^{1}H\}$ NMR spectrum of compound 12l (125 MHz, $CDCl_3$):

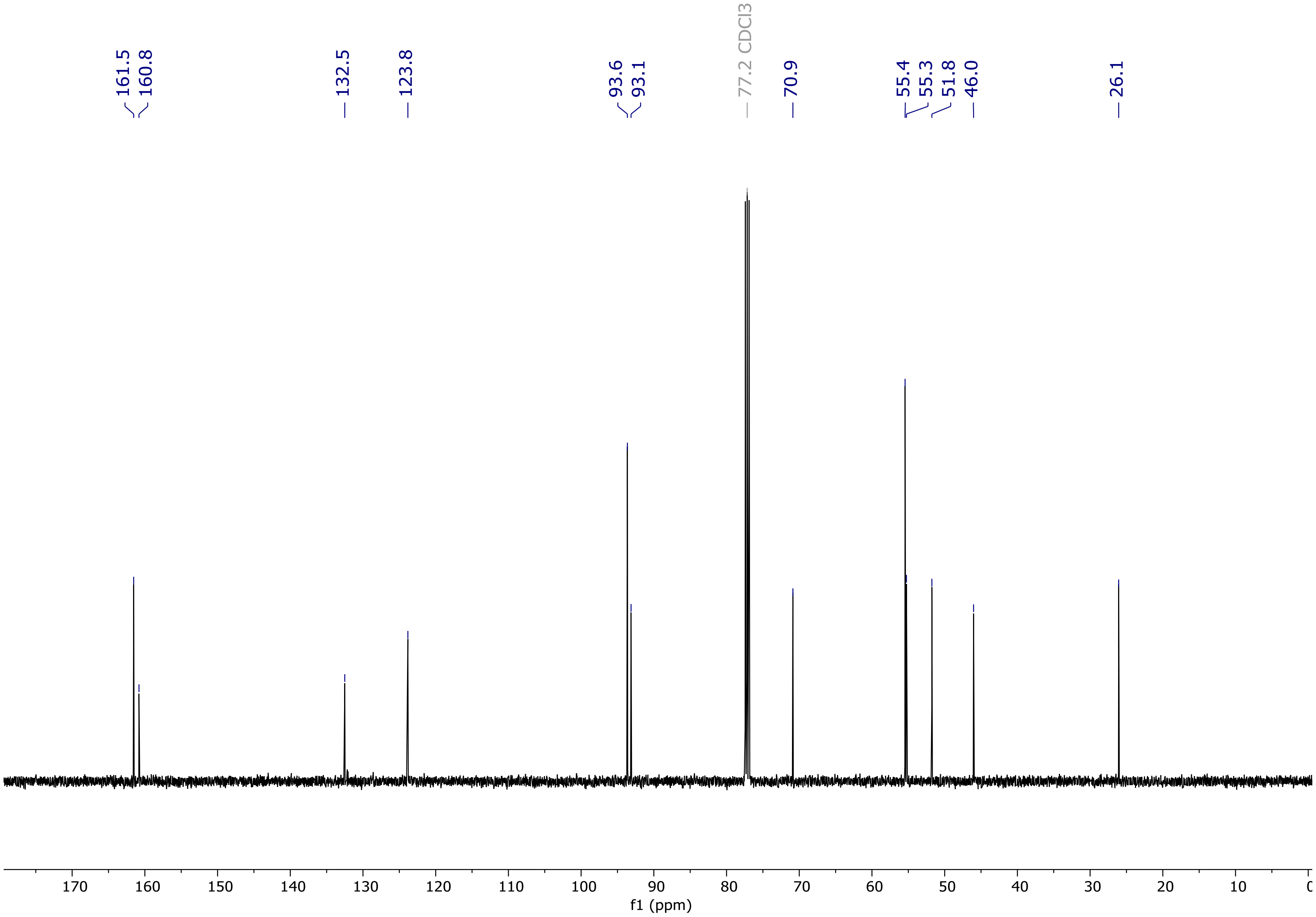

^{1}H NMR spectrum of compound 10 (500 MHz, $CDCl_3$):

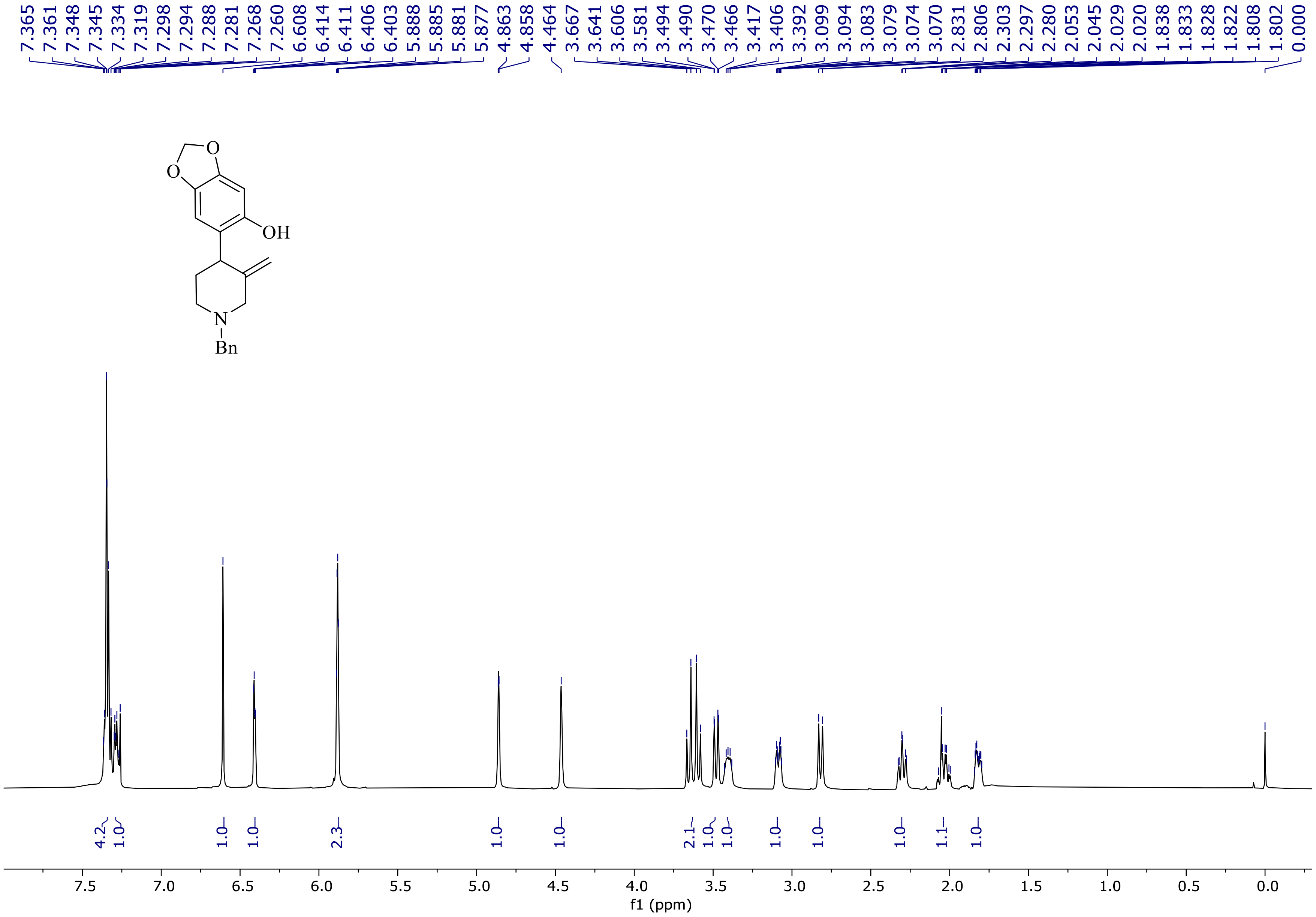

$^{13}C\{^{1}H\}$ NMR spectrum of compound 10 (125 MHz, $CDCl_3$):

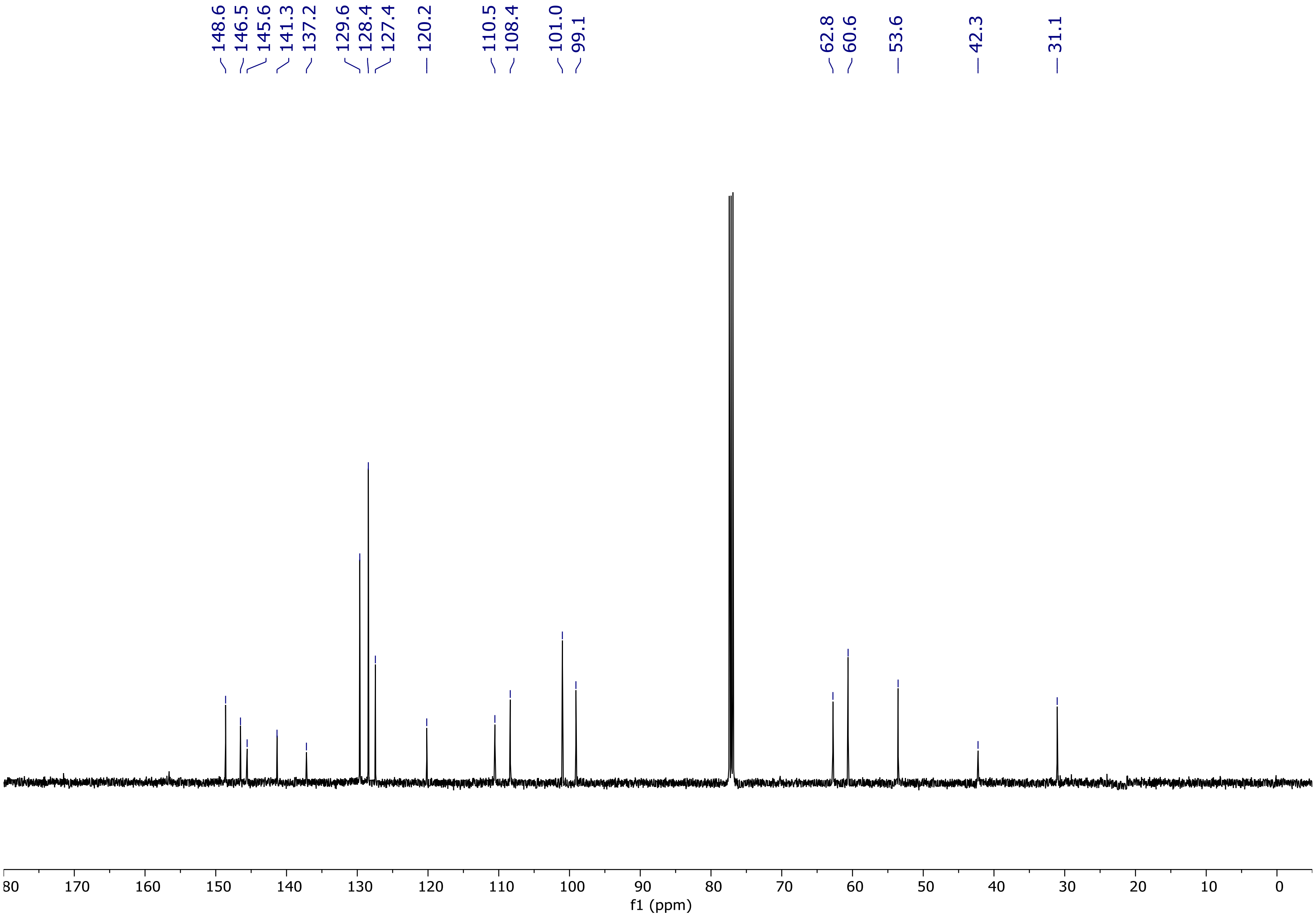

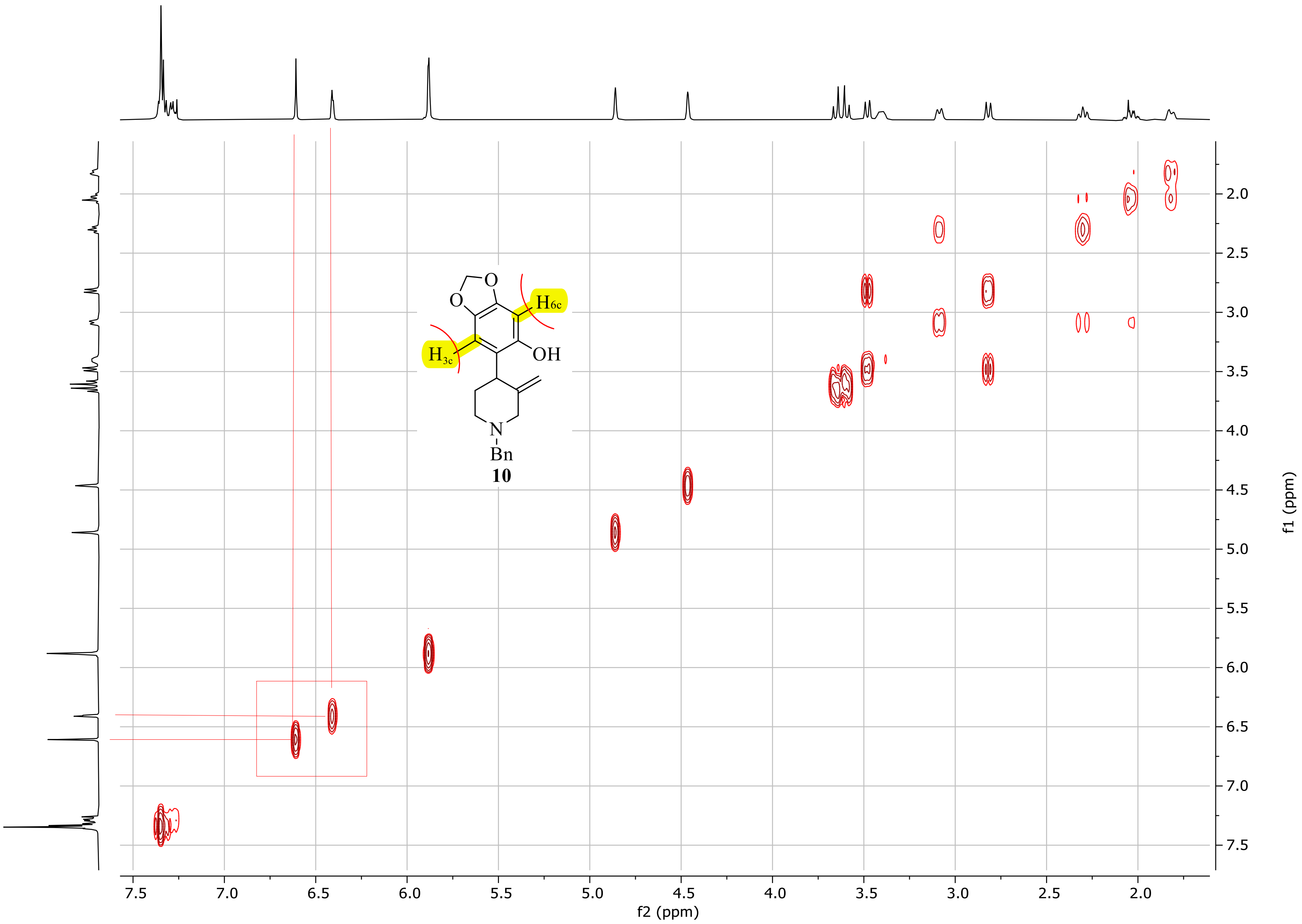

Fig. 1. COSY of ***10****; There is not coupling in the aryl hydrogens* H_{3c} *y* H_{6c}*.*

^{1}H NMR spectrum of compound 13a (500 MHz, $CDCl_3$):

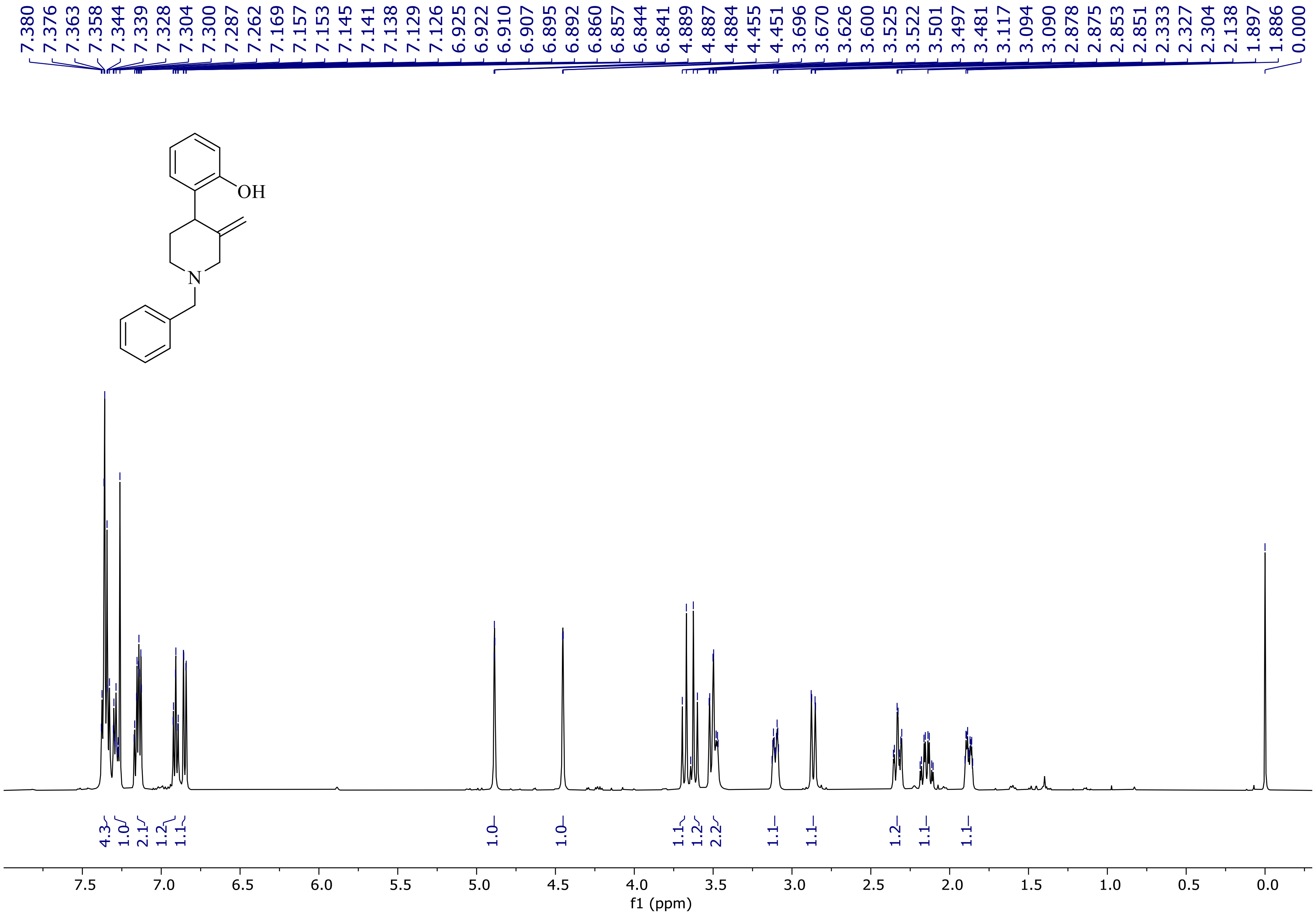

$^{13}C\{^1H\}$ NMR spectrum of compound 13a (125 MHz, $CDCl_3$):

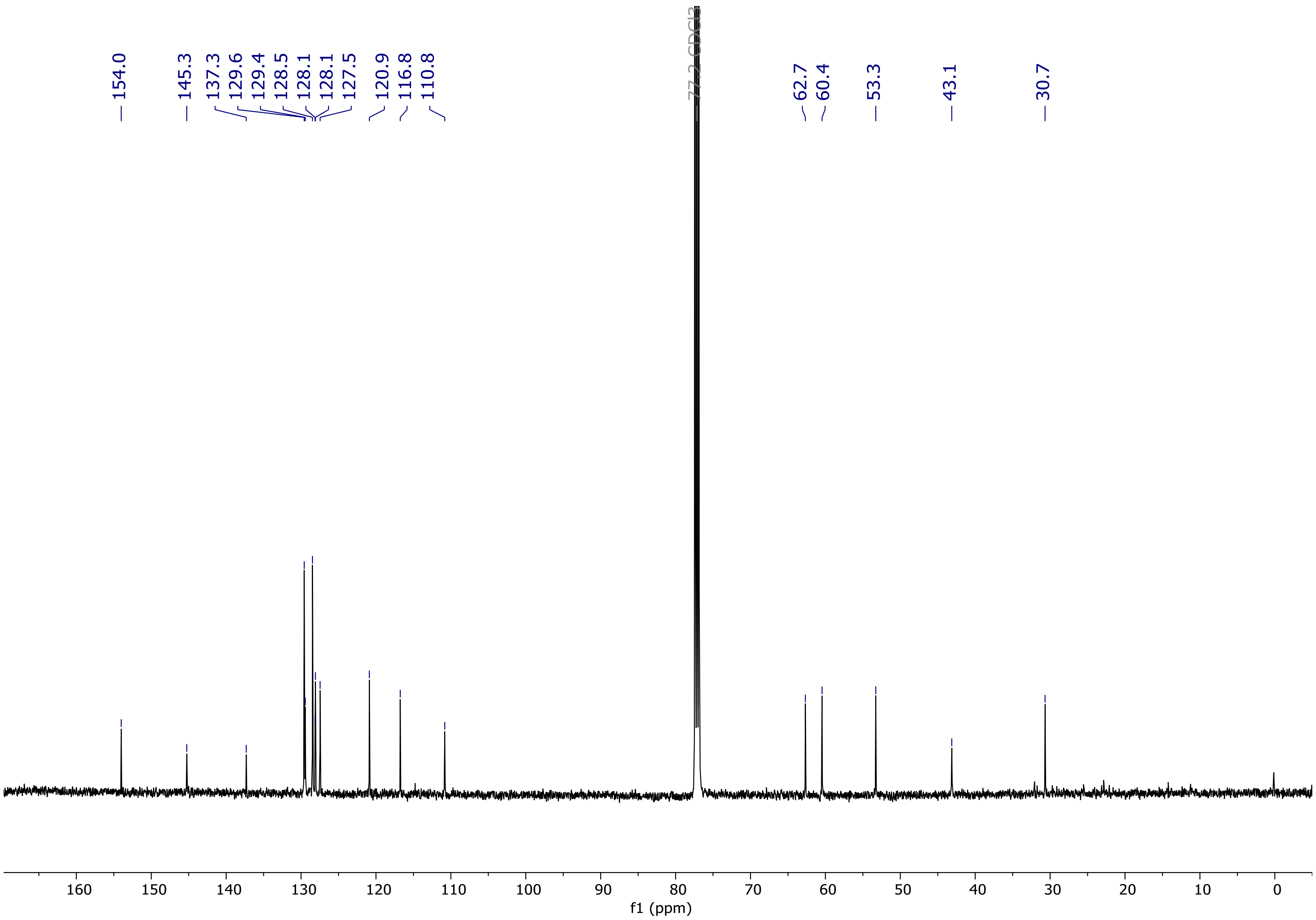

^{1}H NMR spectrum of compound 13b (500 MHz, $CDCl_3$):

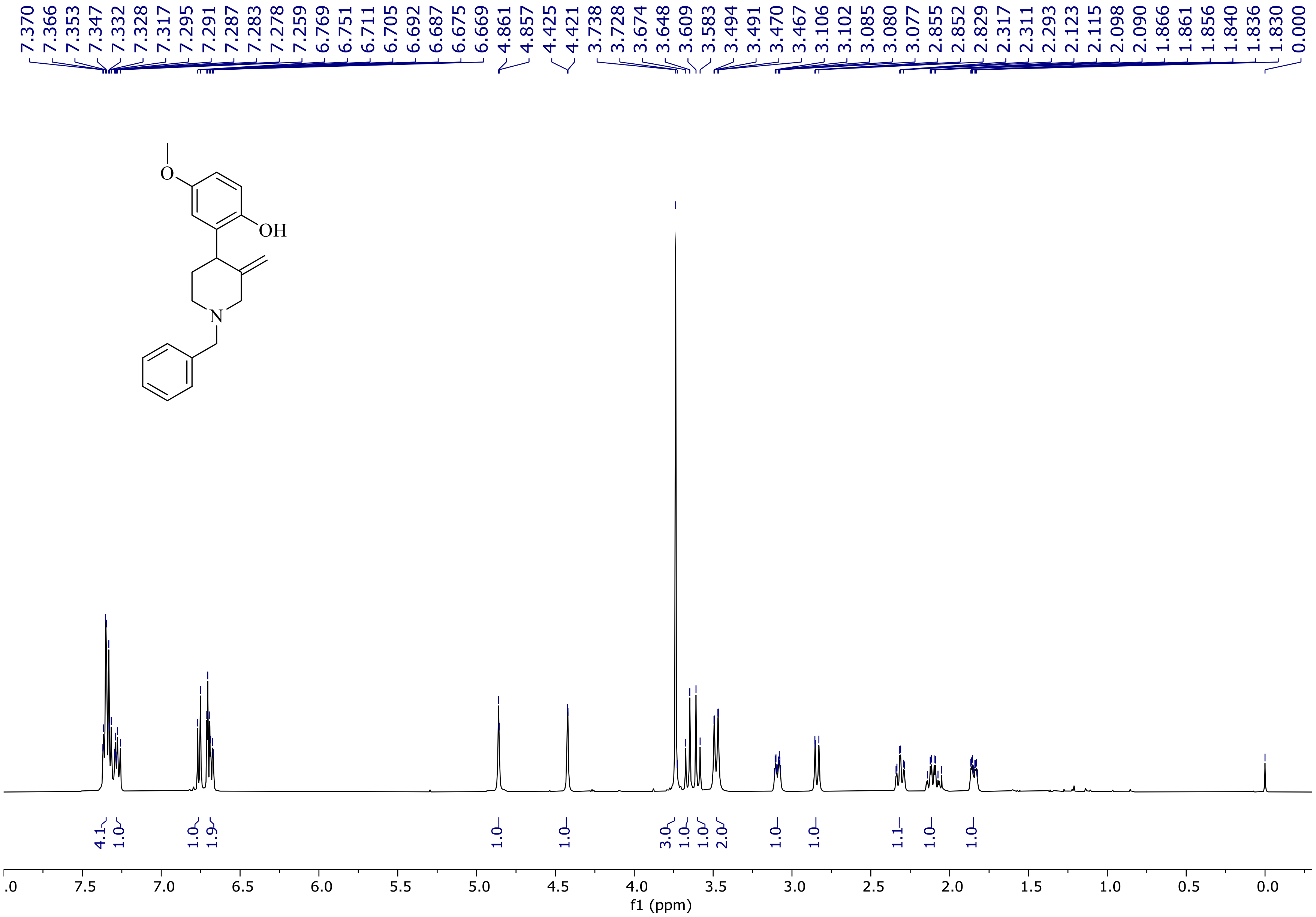

$^{13}C\{^{1}H\}$ **NMR spectrum of compound 13b (125 MHz, $CDCl_3$):**

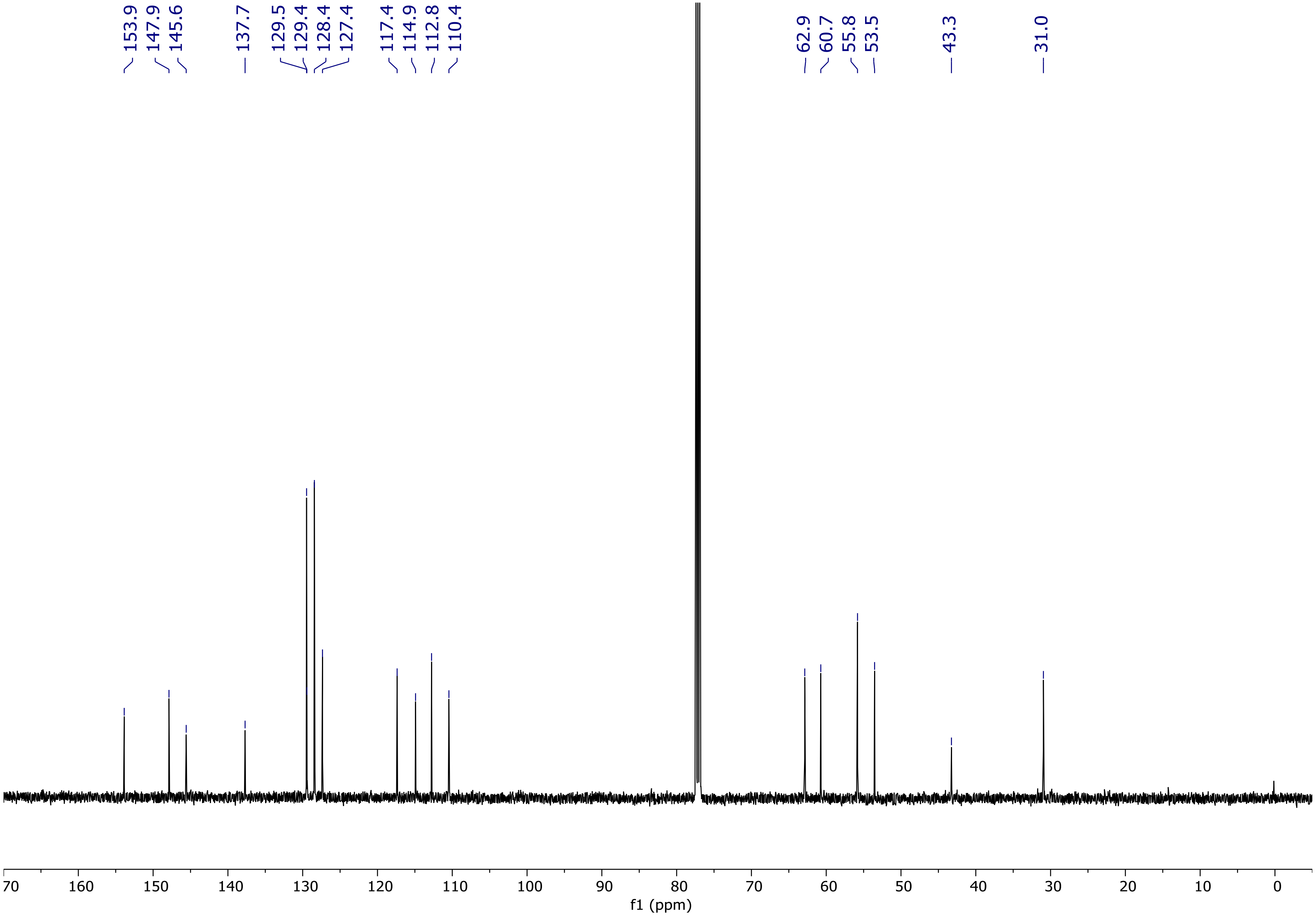

^{1}H NMR spectrum of compound 13d (500 MHz, $CDCl_3$):

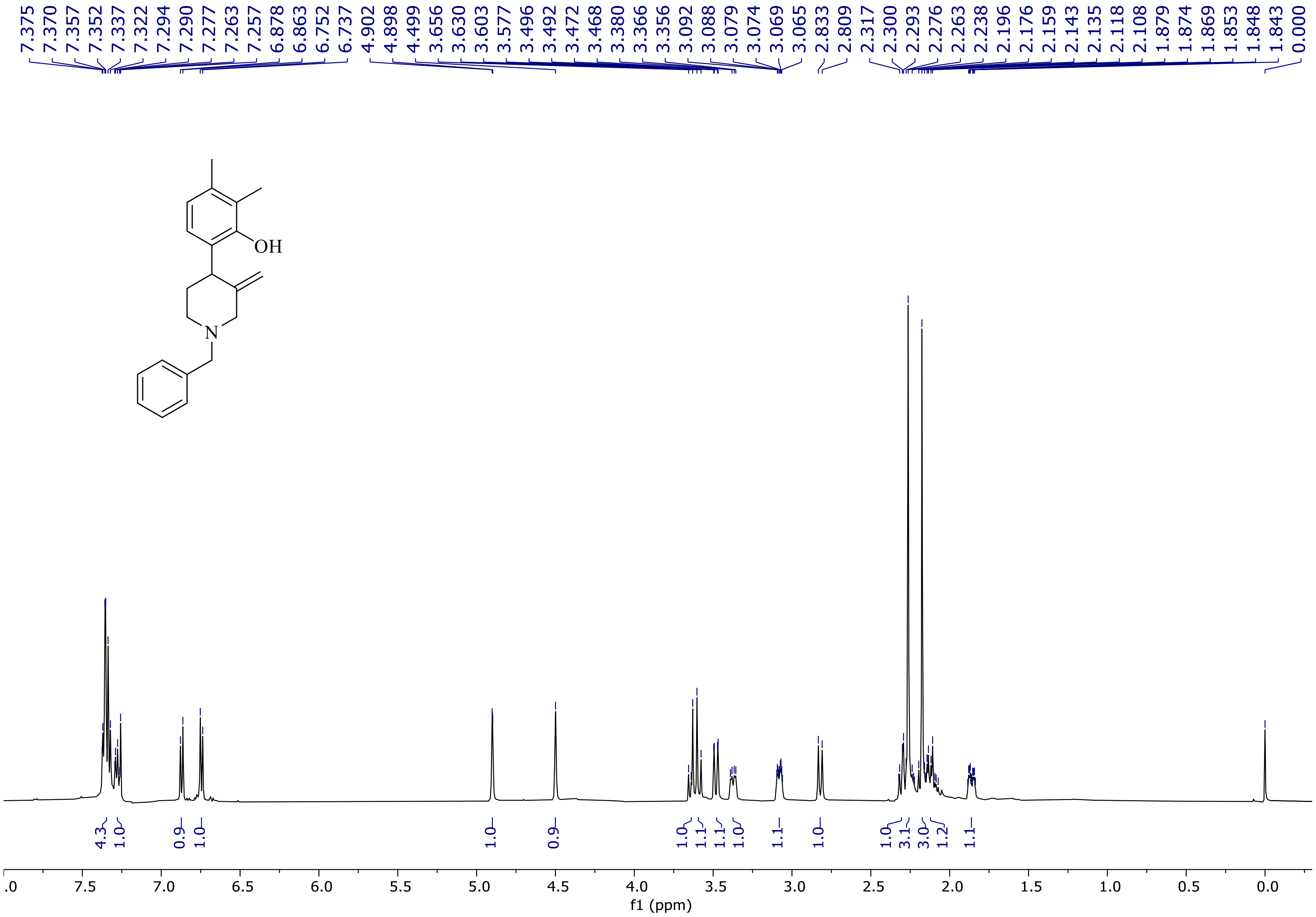

$^{13}C\{^{1}H\}$ NMR spectrum of compound 13d (125 MHz, $CDCl_3$):

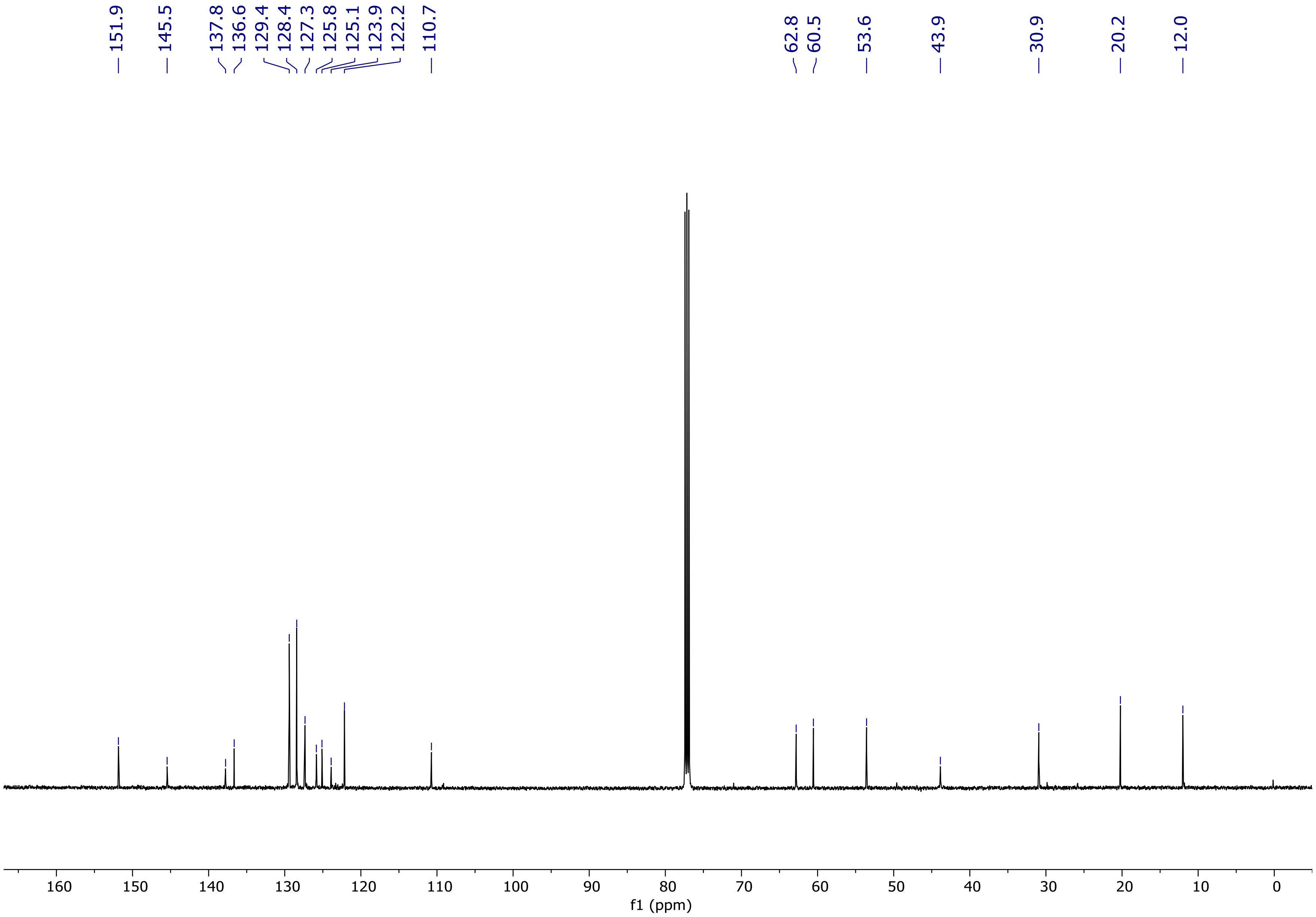

^{1}H NMR spectrum of compound 13e (500 MHz, CDCl3):

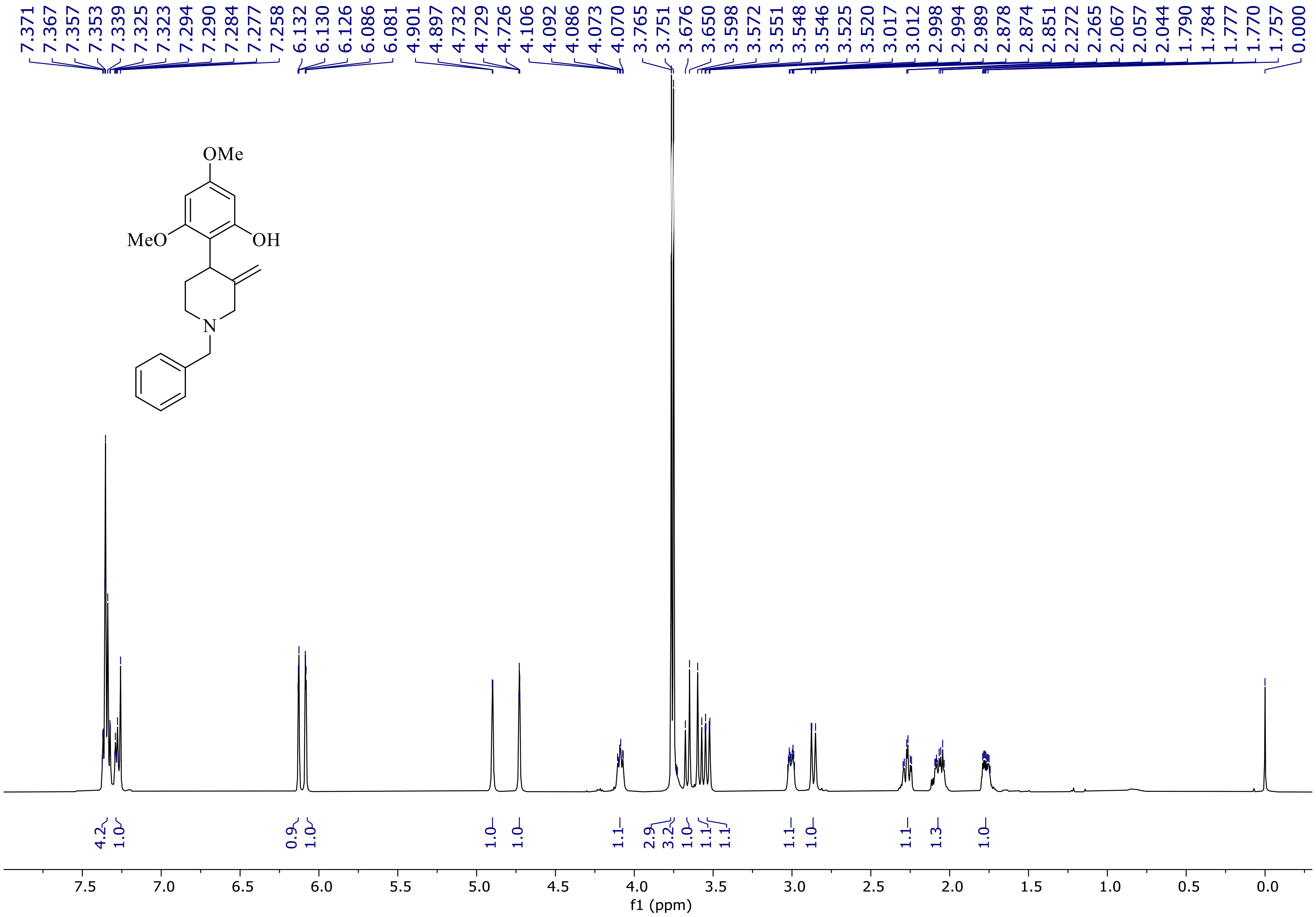

$^{13}C\{^1H\}$ NMR spectrum of compound 13e (125 MHz, $CDCl_3$):

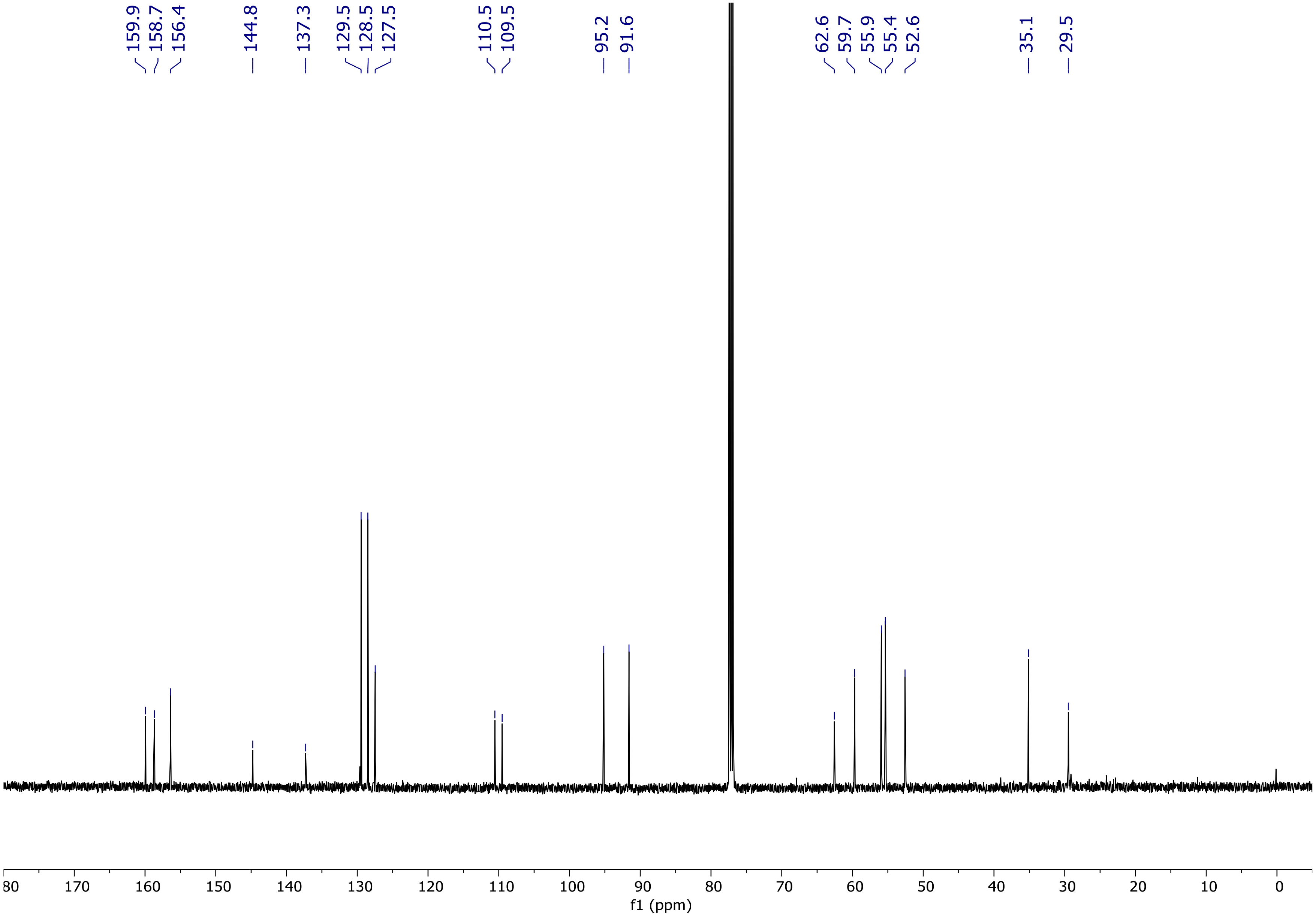

^{1}H NMR spectrum of compound 13g (500 MHz, $CDCl_3$):

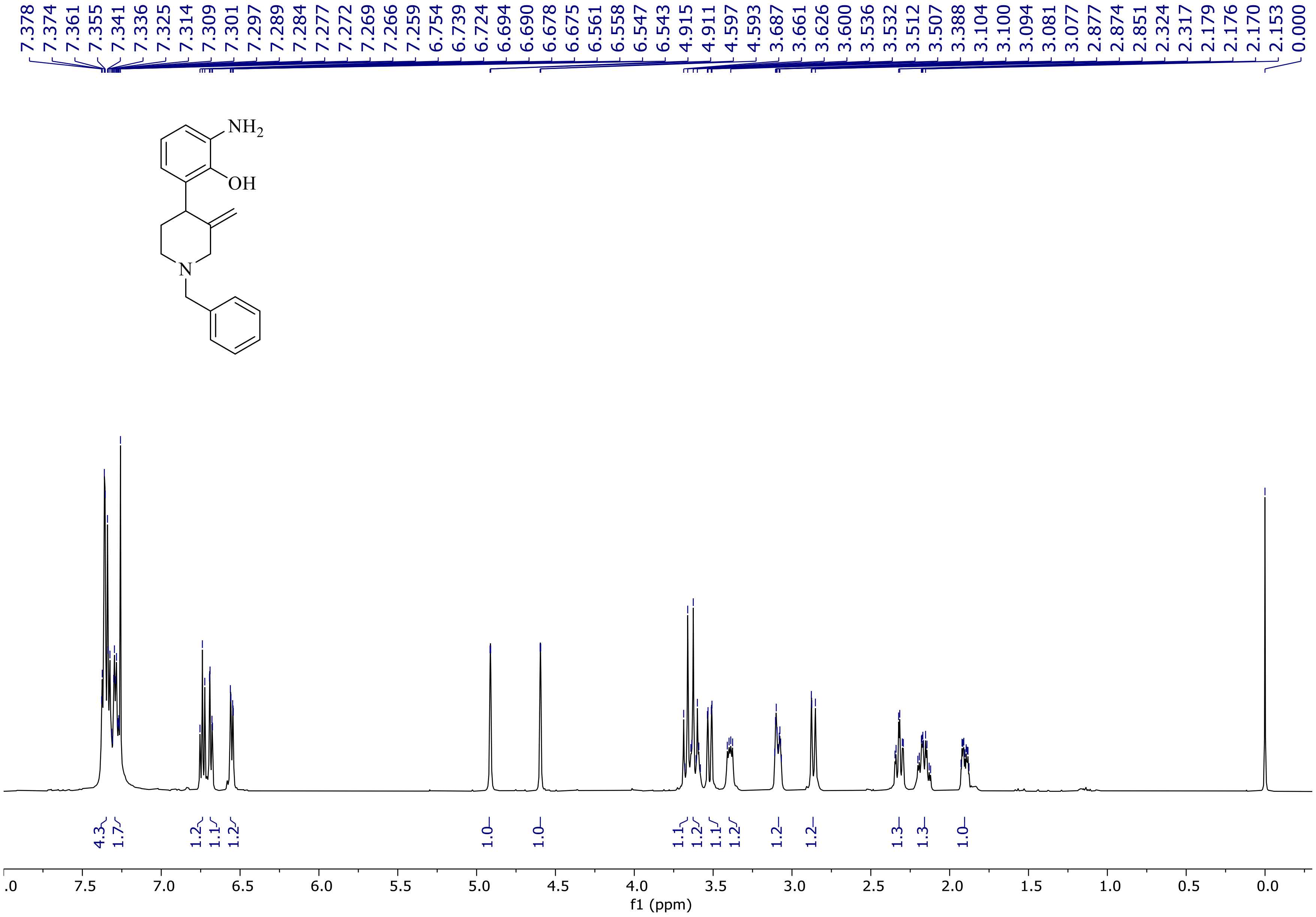

$^{13}C\{^1H\}$ NMR spectrum of compound 13g (125 MHz, $CDCl_3$):

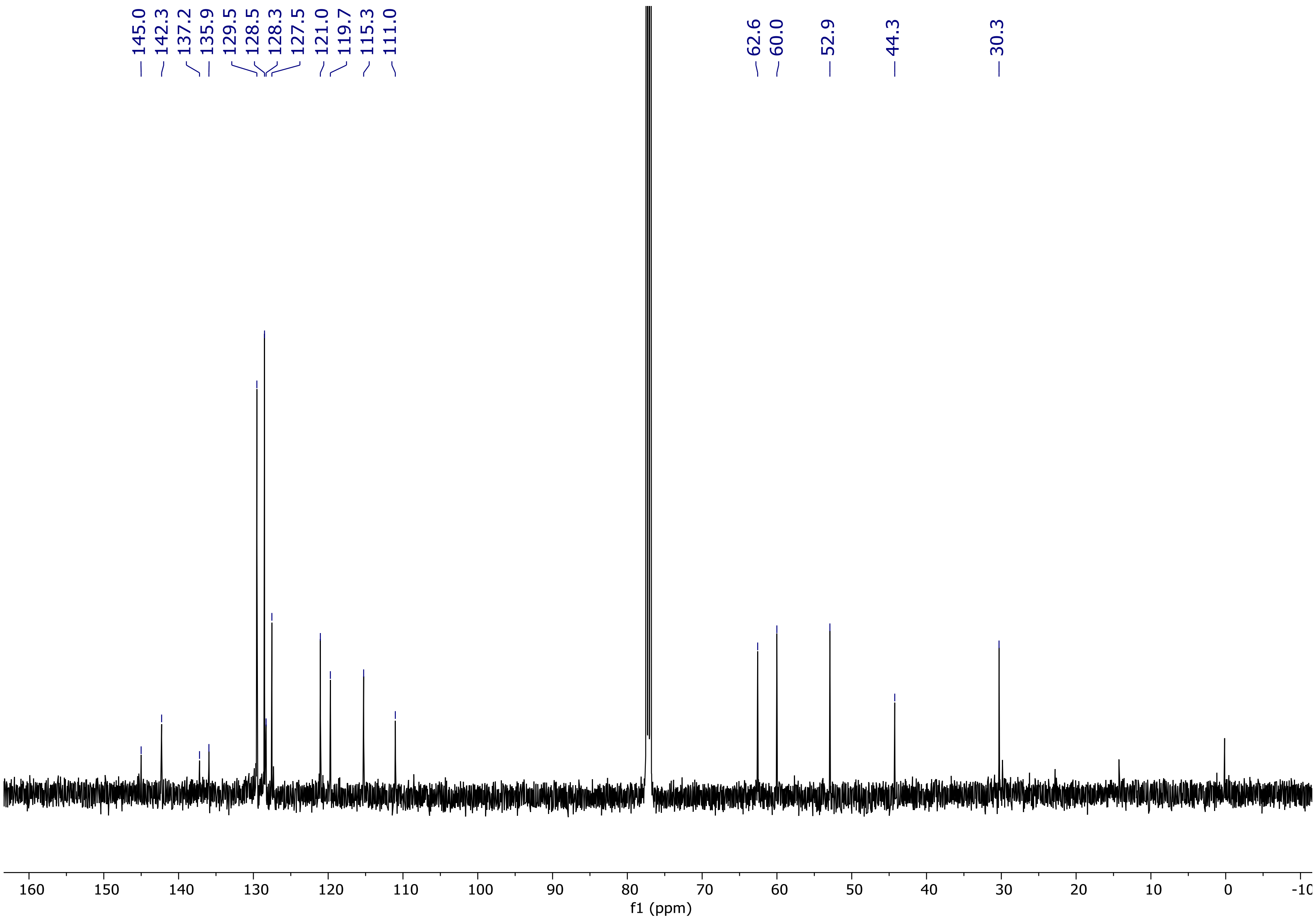

^{1}H NMR spectrum of compound 13gg (500 MHz, $CDCl_3$):

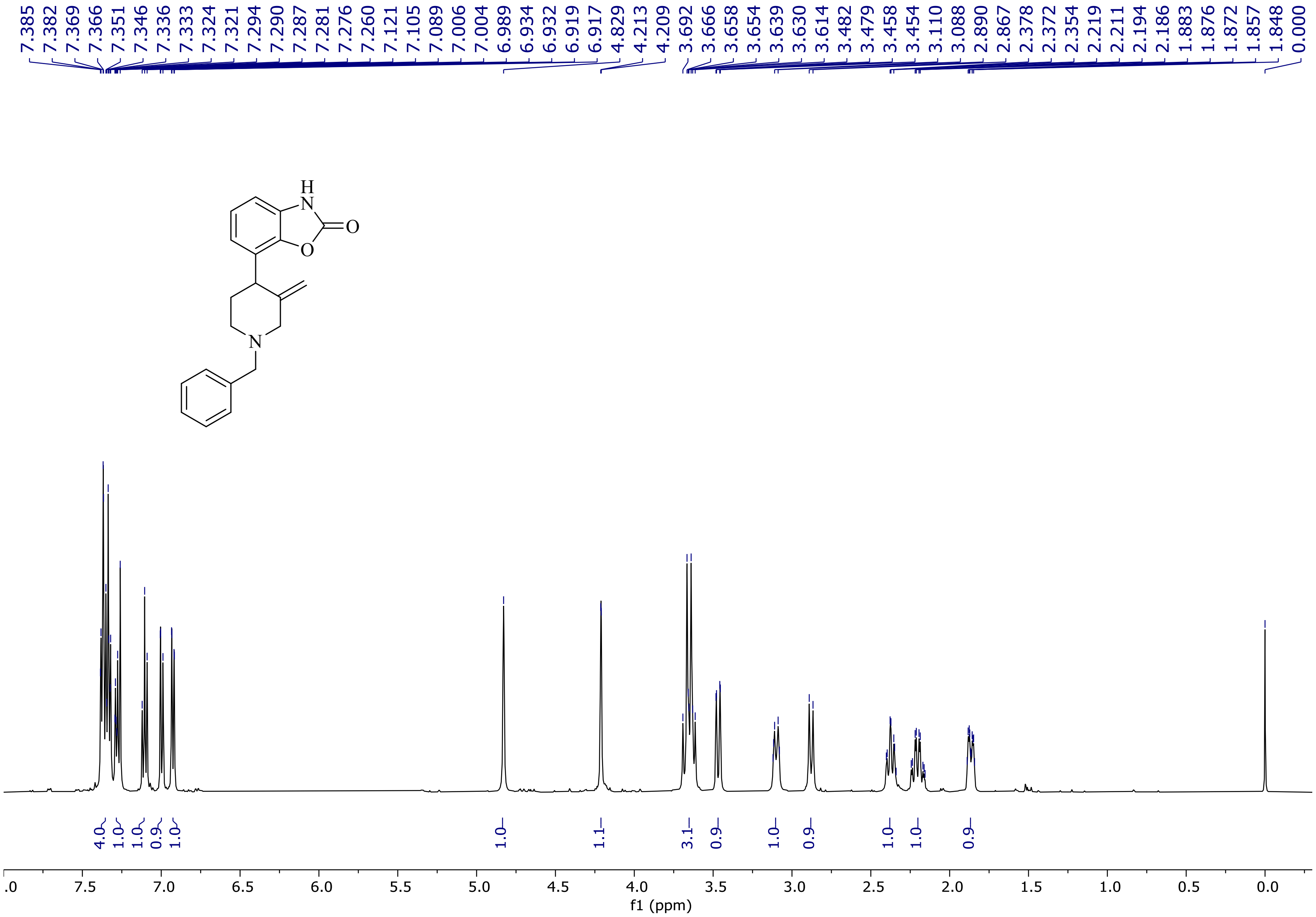

$^{13}C\{^{1}H\}$ NMR spectrum of compound 13gg (125 MHz, $CDCl_3$):

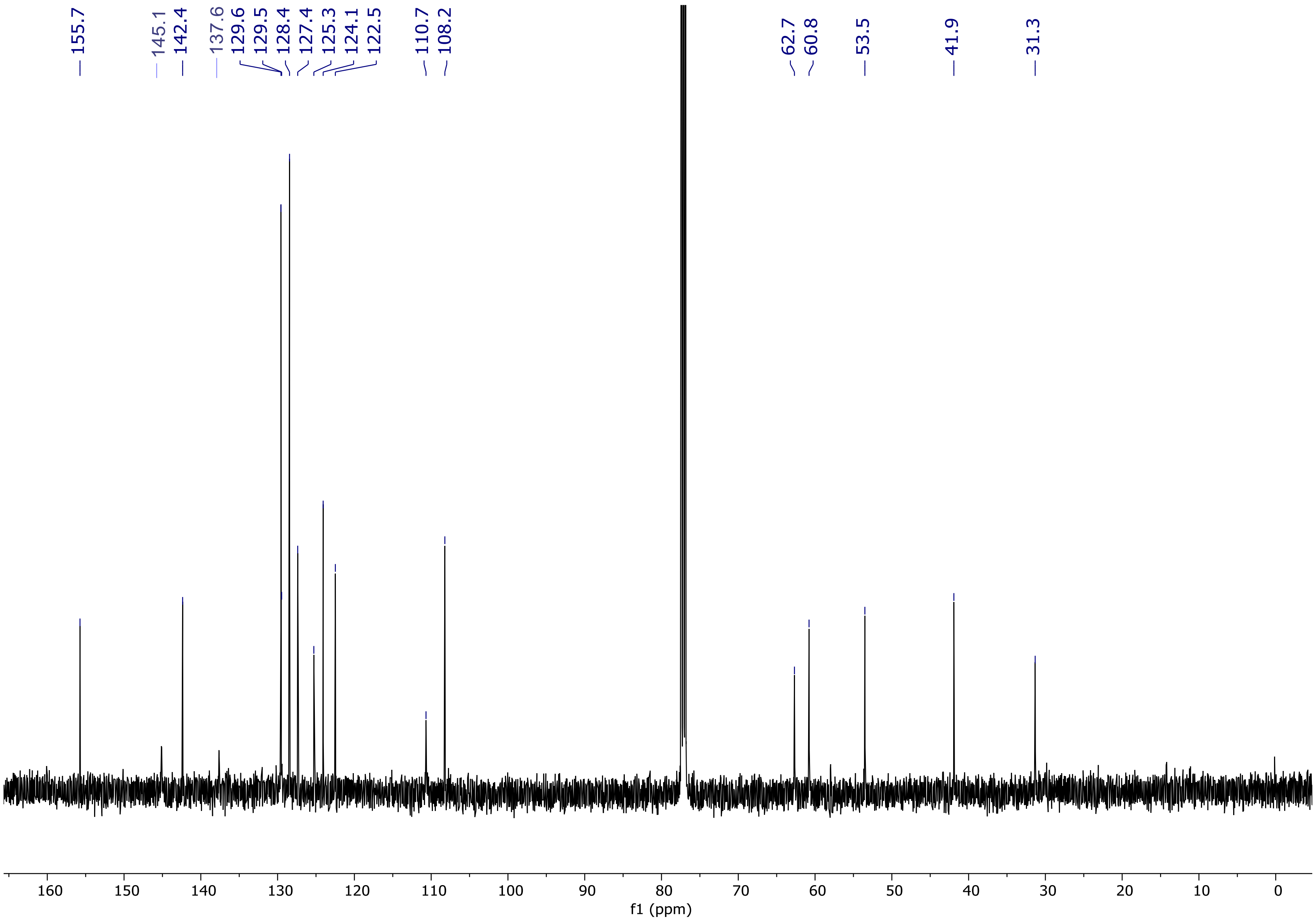

^{1}H NMR spectrum of compound 13j (500 MHz, $CDCl_3$):

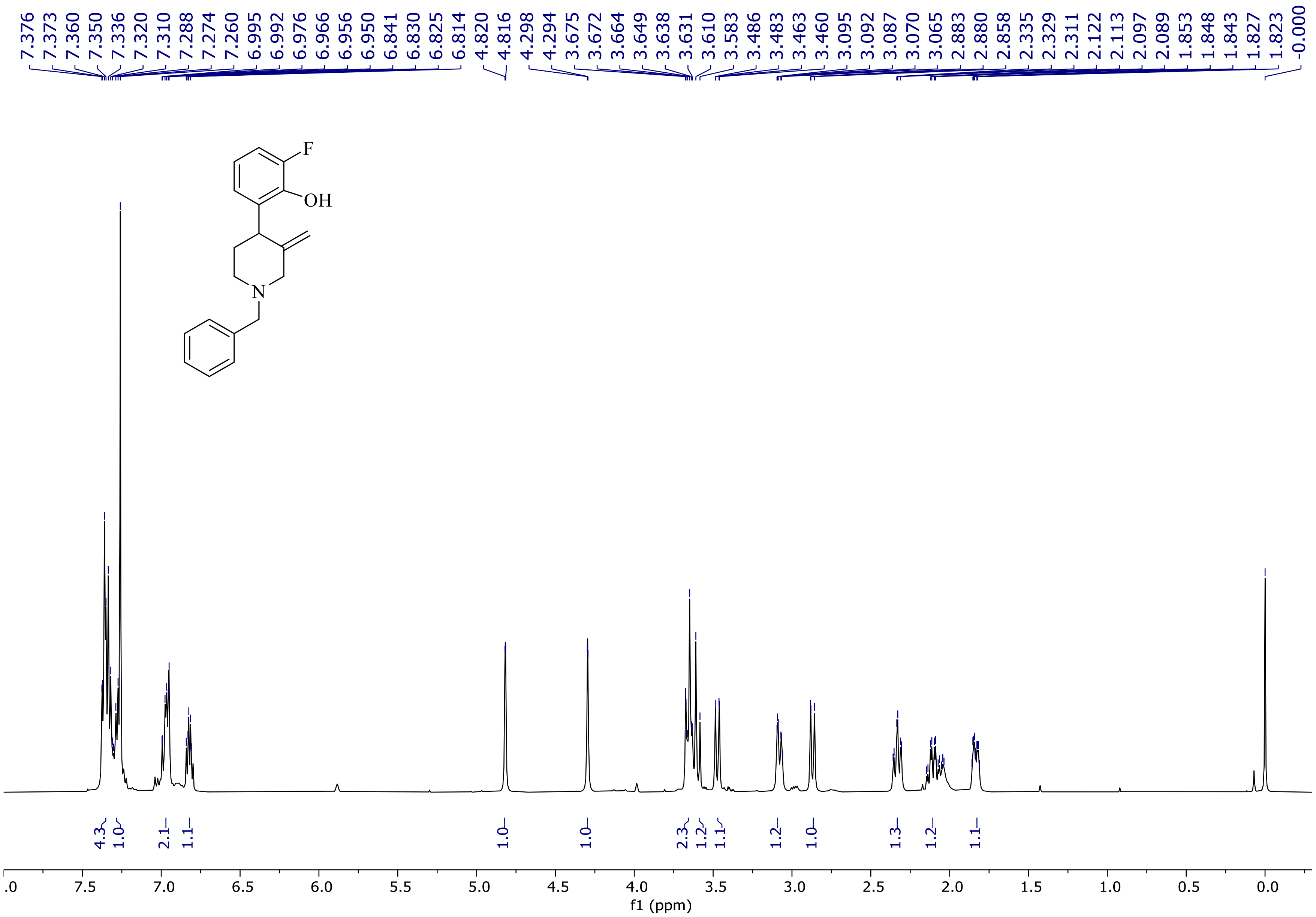

$^{13}C\{^1H\}$ NMR spectrum of compound 13j (125 MHz, $CDCl_3$):

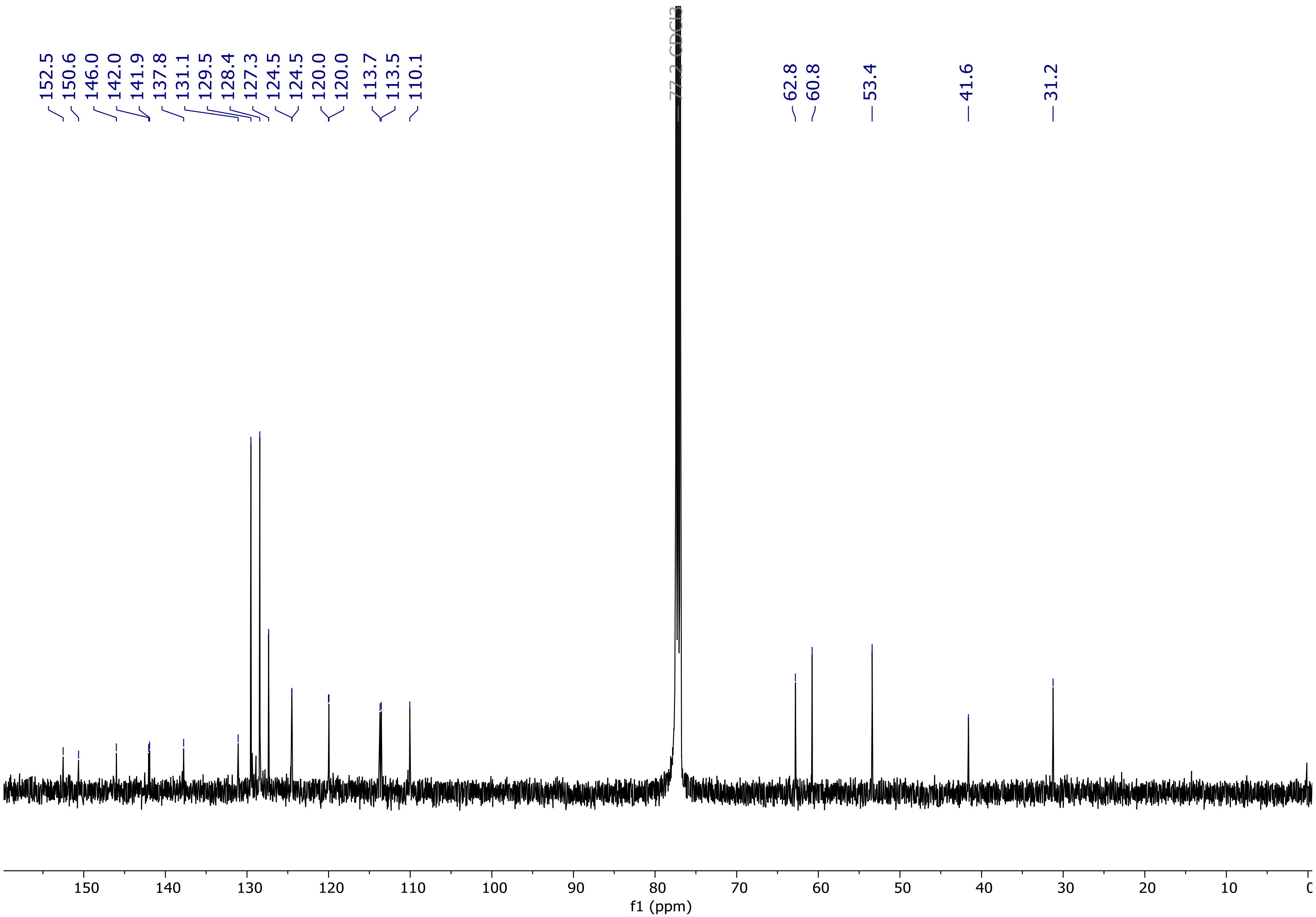

^{1}H NMR spectrum of compound 13k (500 MHz, $CDCl_3$):

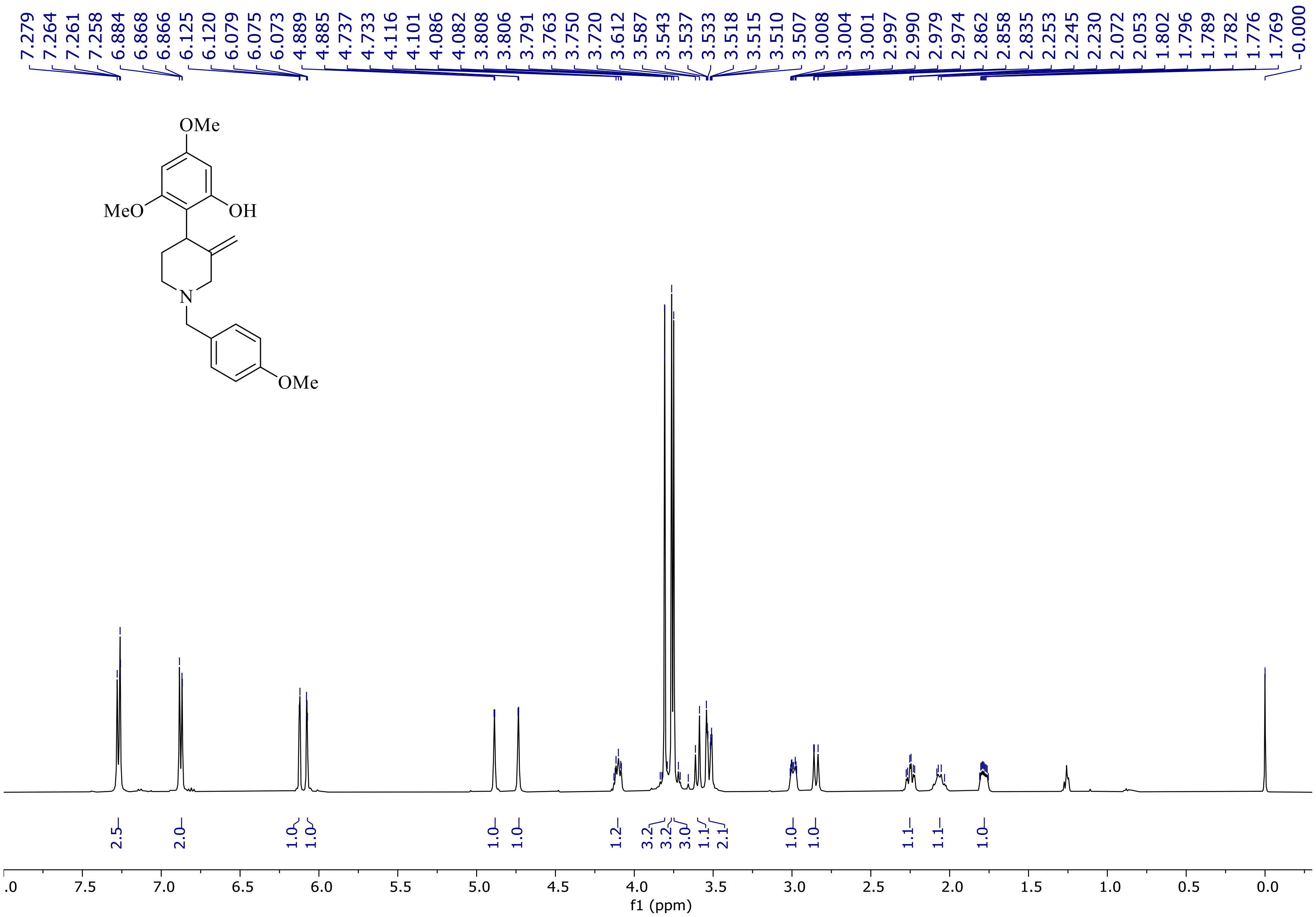

$^{13}C\{^{1}H\}$ NMR spectrum of compound 13k (125 MHz, CDCl$_3$):

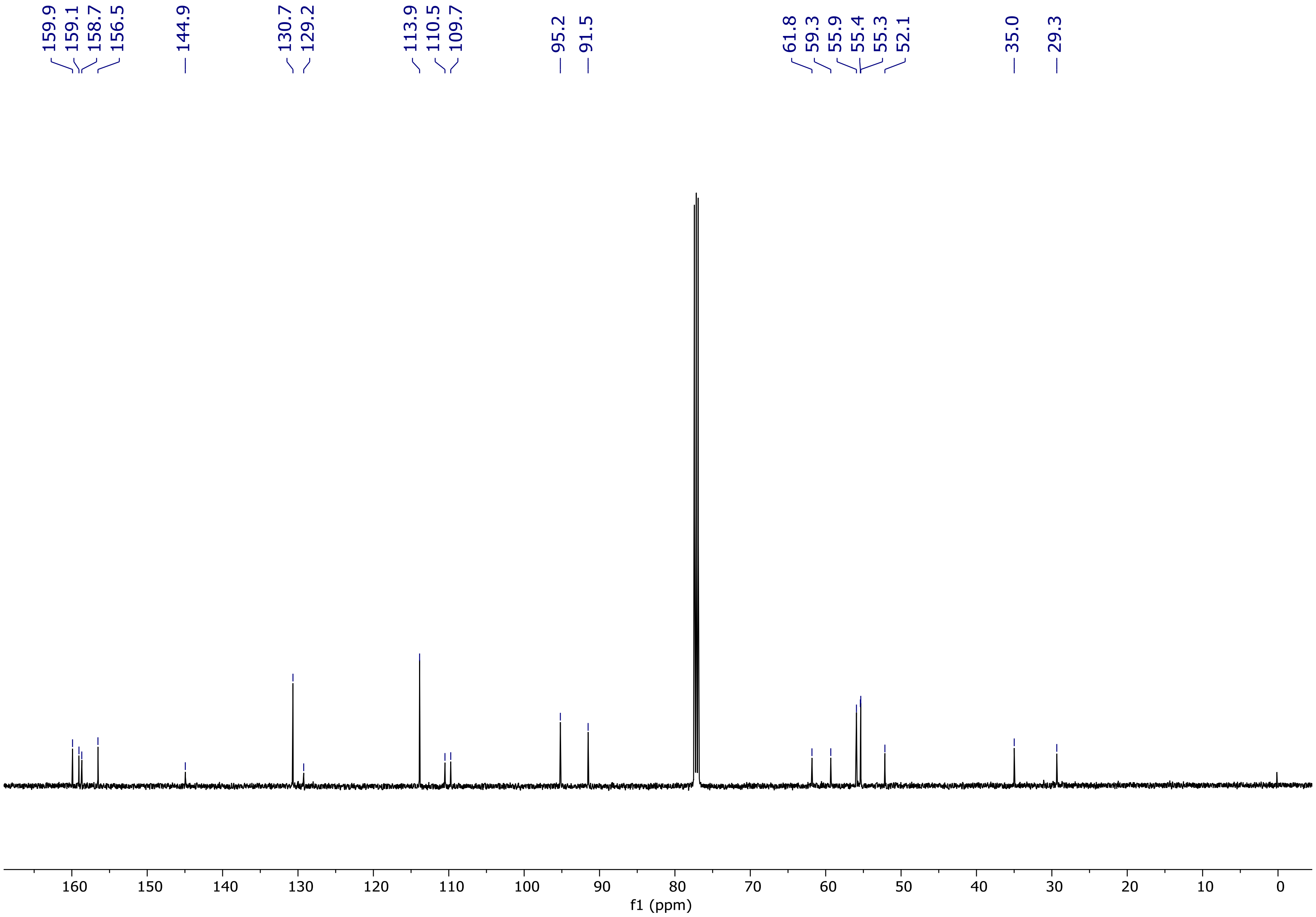

^{1}H NMR spectrum of compound 13l (500 MHz, $CDCl_3$):

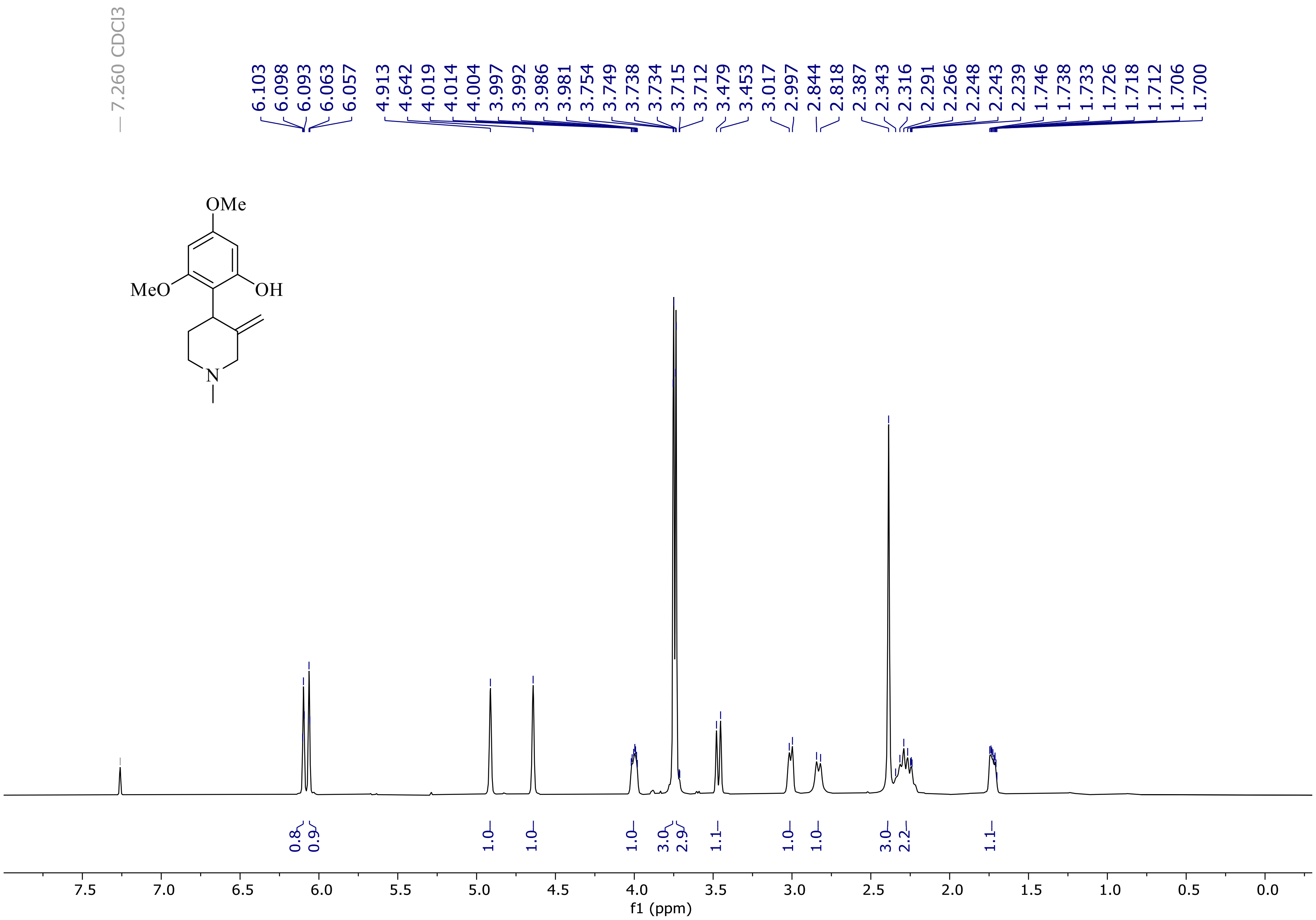

$^{13}C\{^{1}H\}$ NMR spectrum of compound 13l (125 MHz, $CDCl_3$):

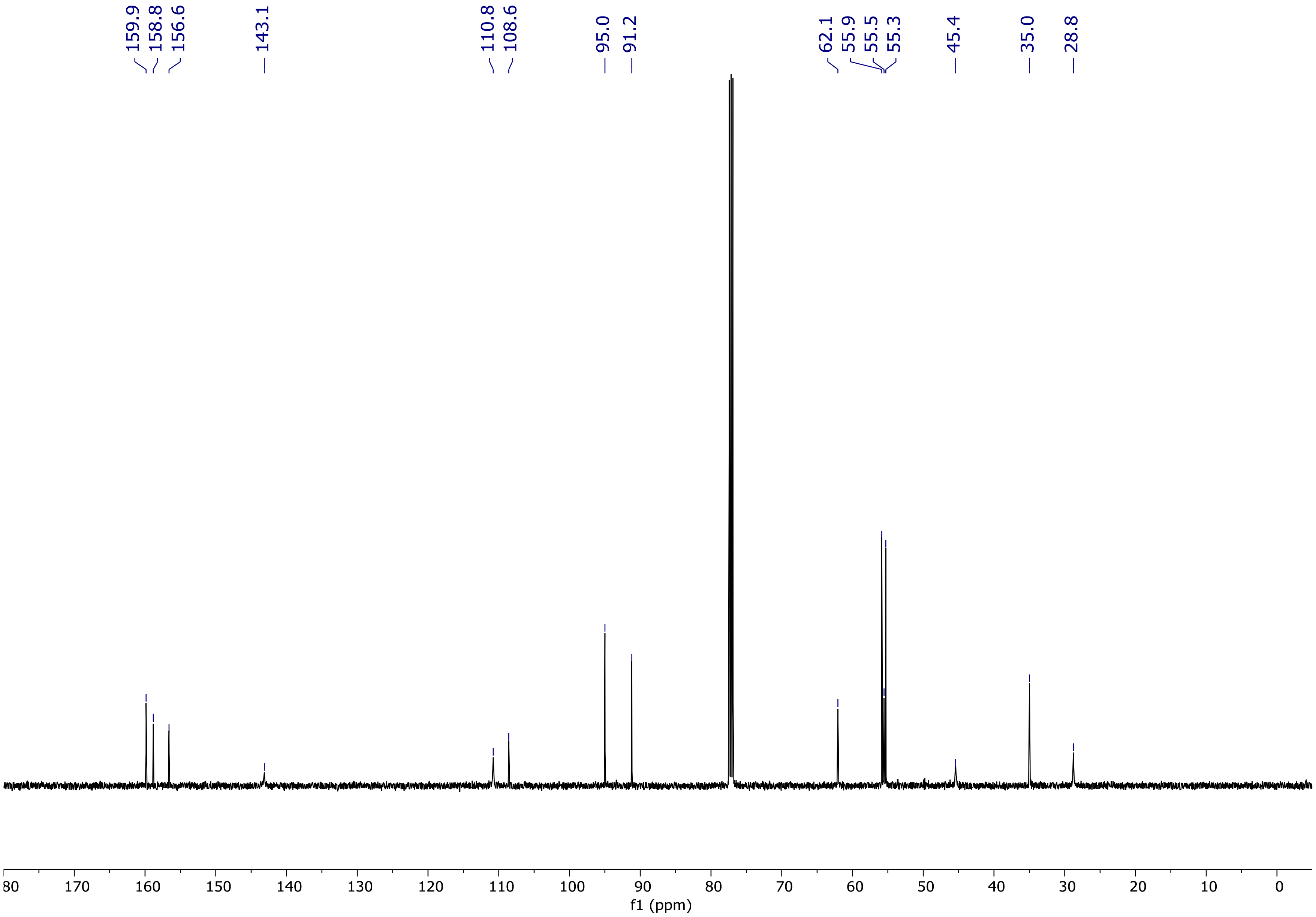

^{1}H NMR spectrum of compound 16 (500 MHz, $CDCl_3$):

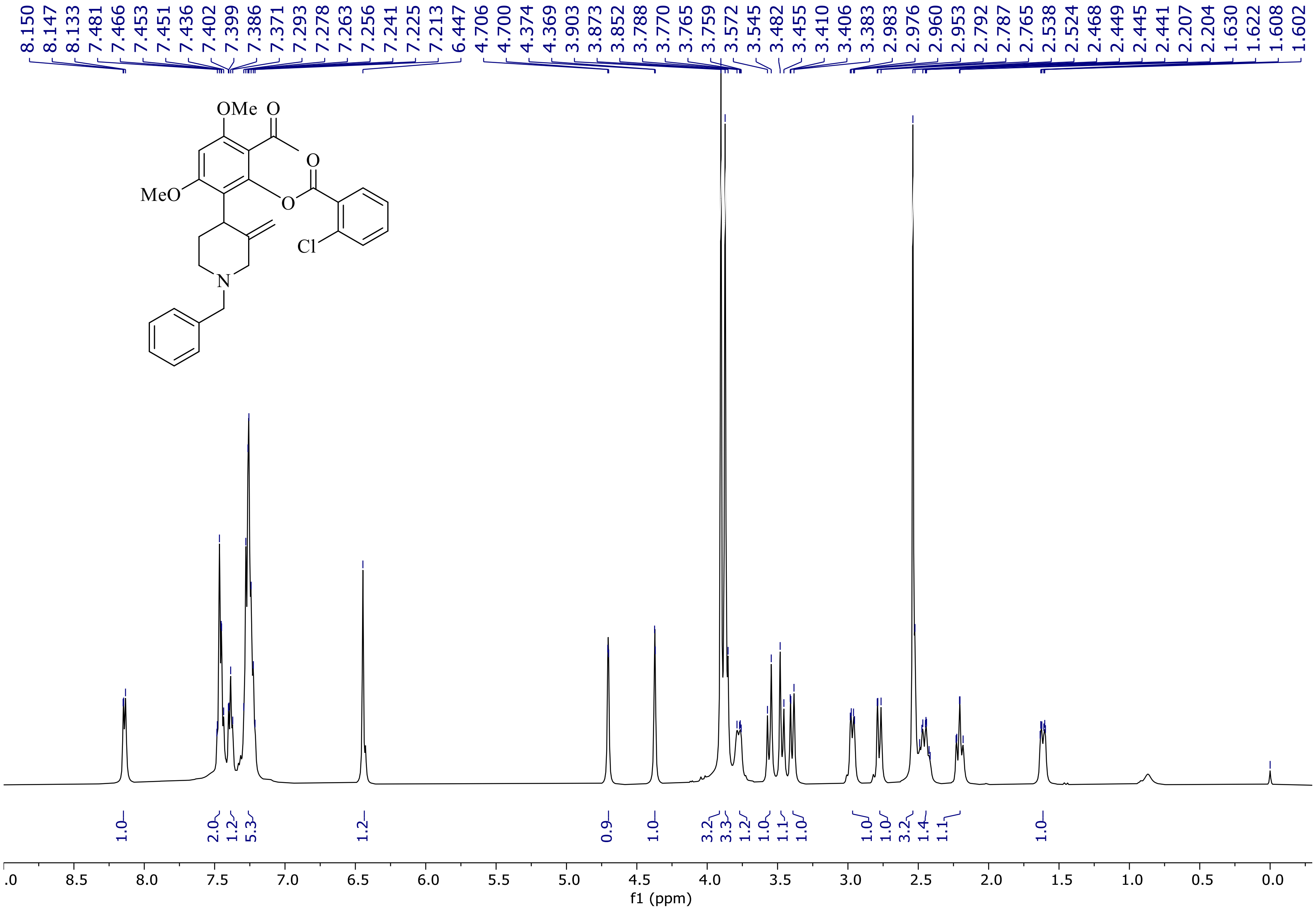

$^{13}C\{^{1}H\}$ NMR spectrum of compound 16 (125 MHz, $CDCl_3$):

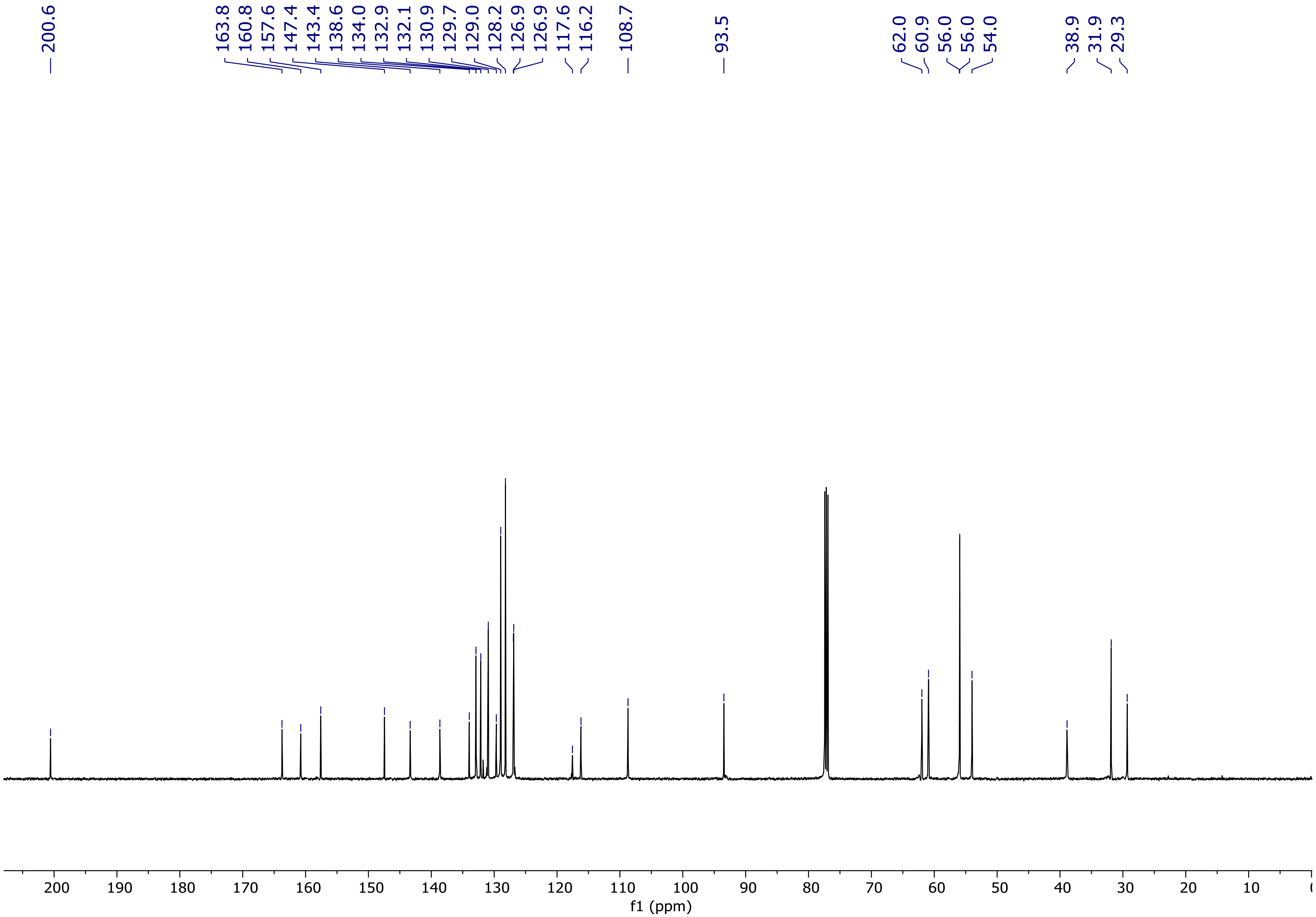

^{1}H NMR spectrum of compound 17 (500 MHz, CDCl₃):

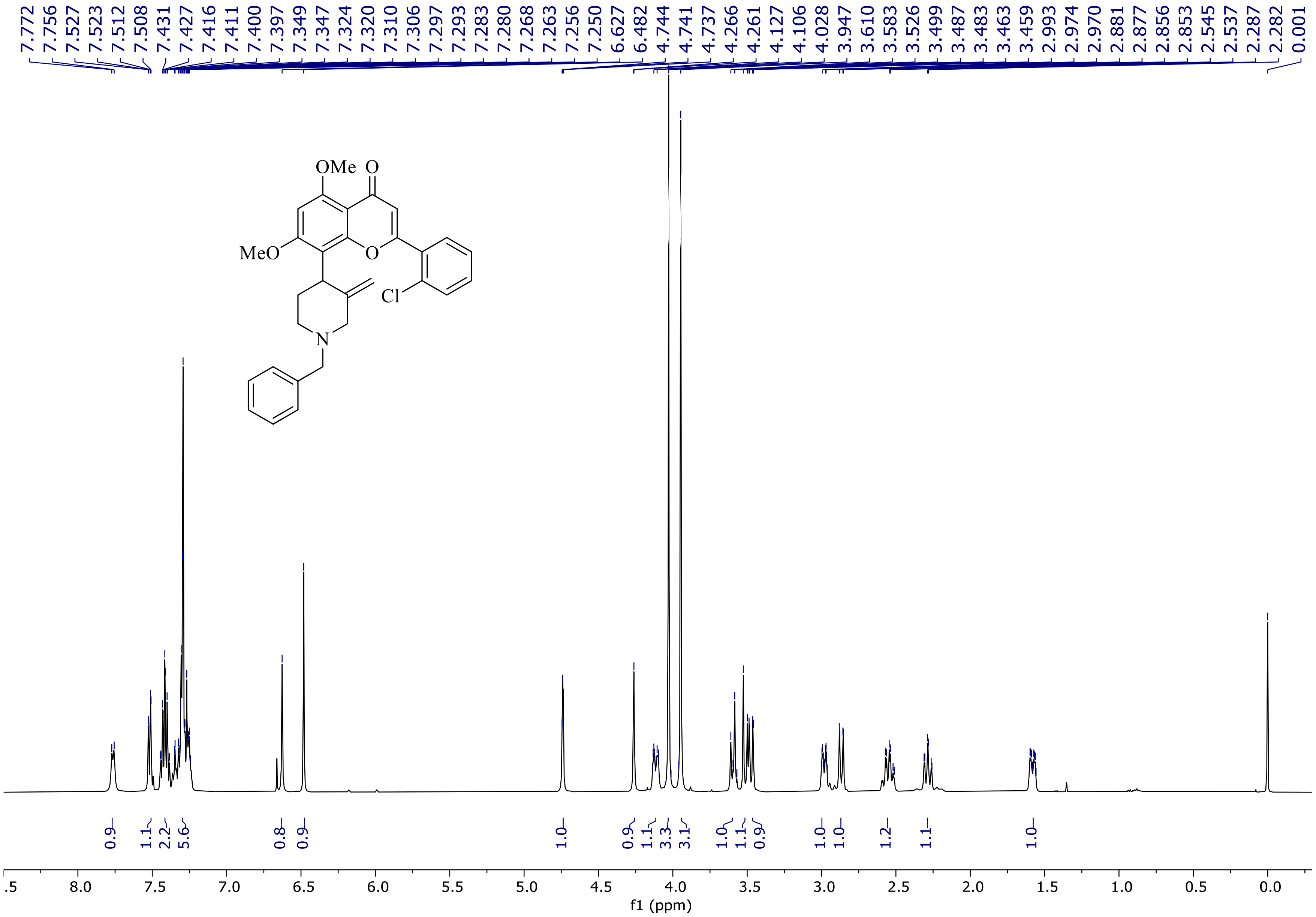

$^{13}C\{^{1}H\}$ NMR spectrum of compound 17 (125 MHz, $CDCl_3$):

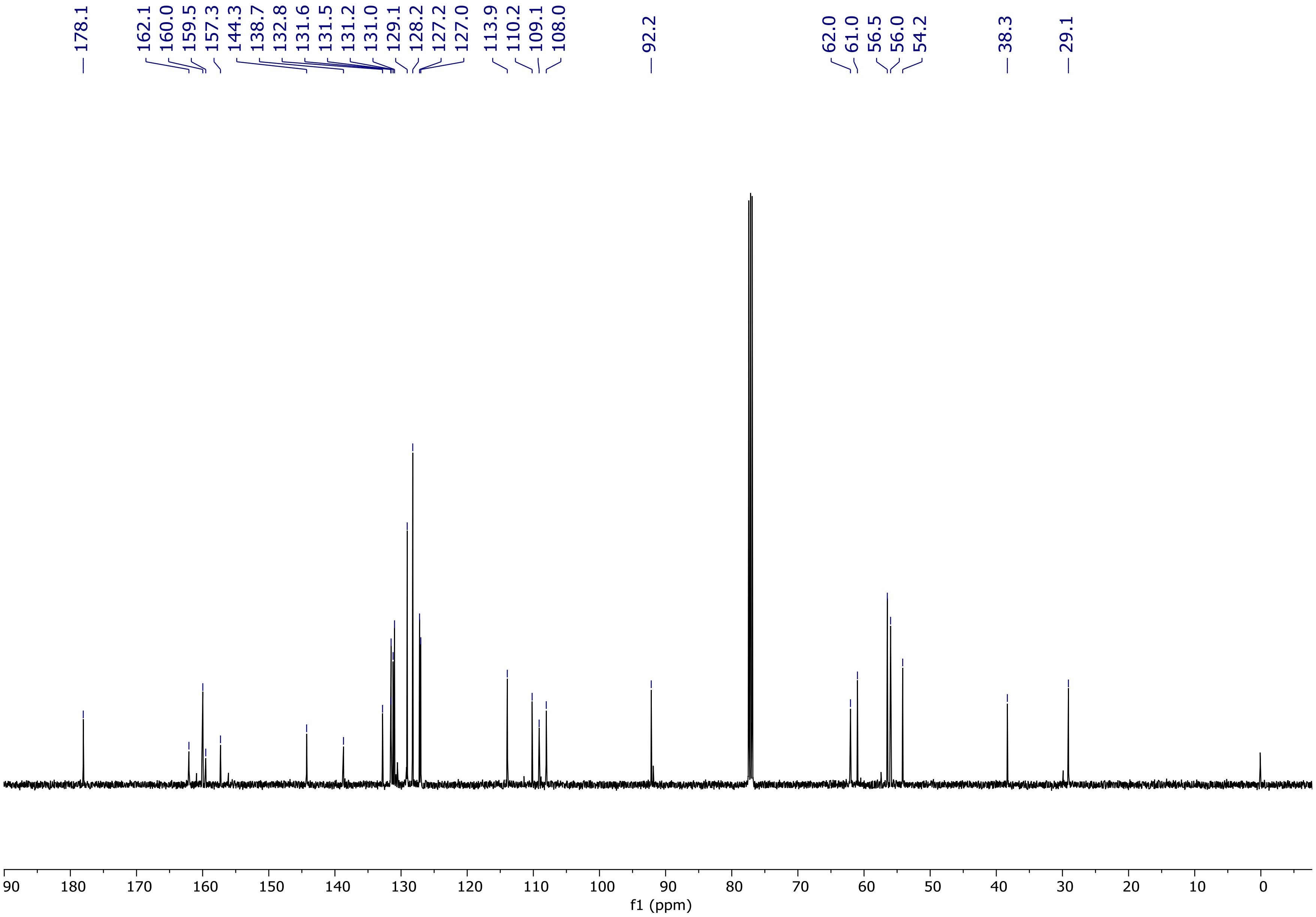

^{1}H NMR spectrum of compound 19 (500 MHz, CDCl$_3$):

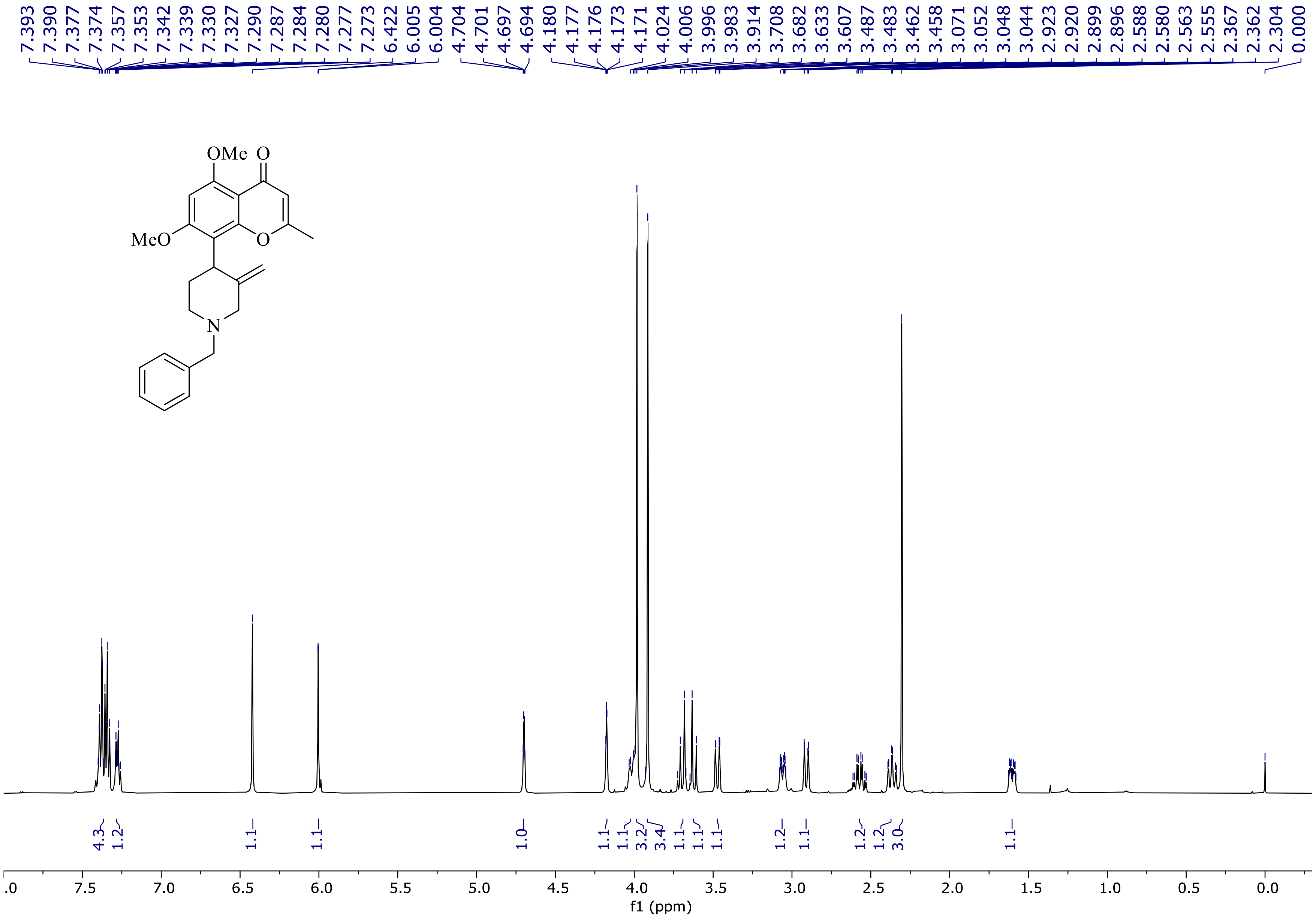

$^{13}C\{^{1}H\}$ NMR spectrum of compound 19 (125 MHz, $CDCl_3$):

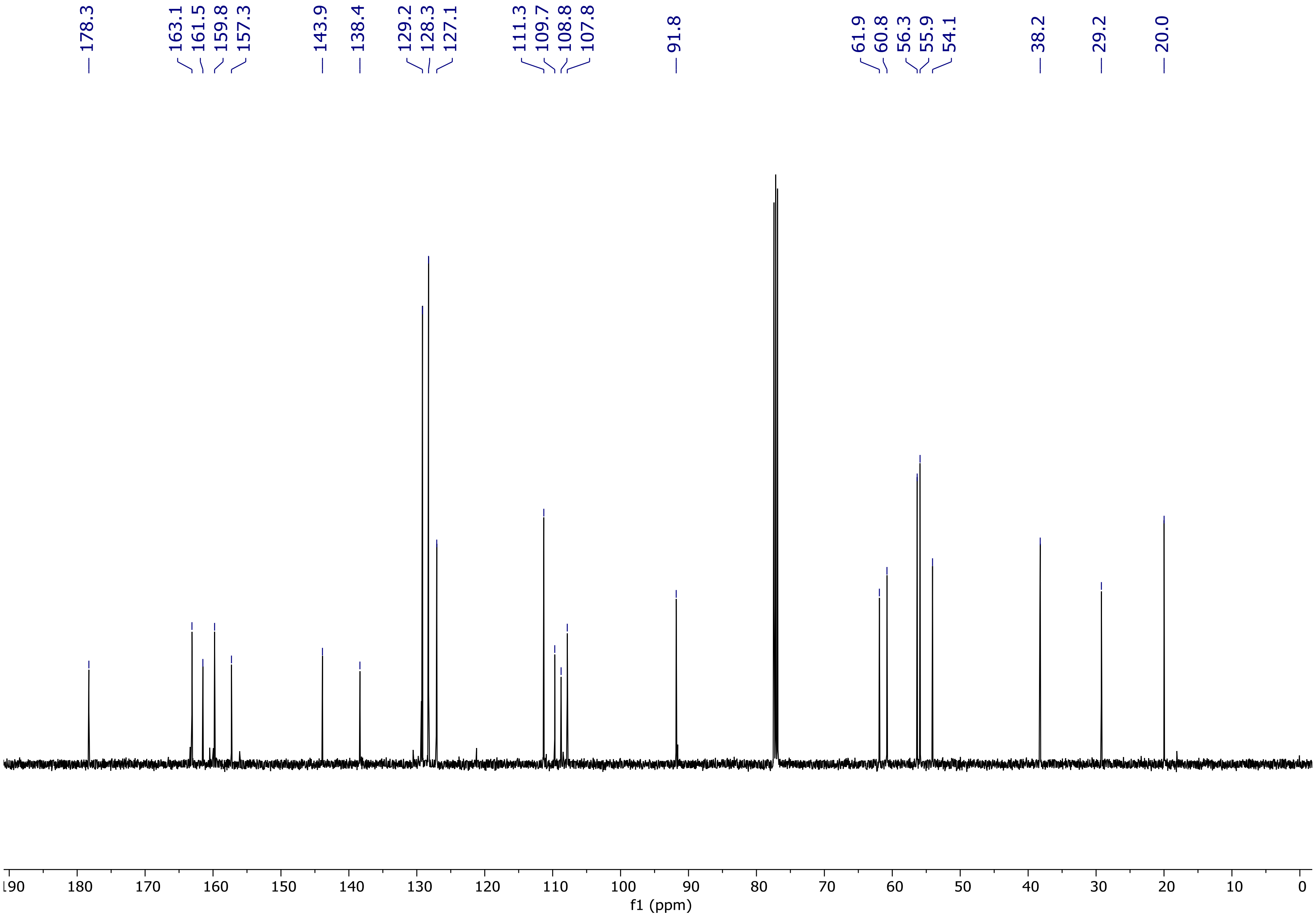

^{1}H NMR spectrum of compound 21 (500 MHz, CD_3CN):

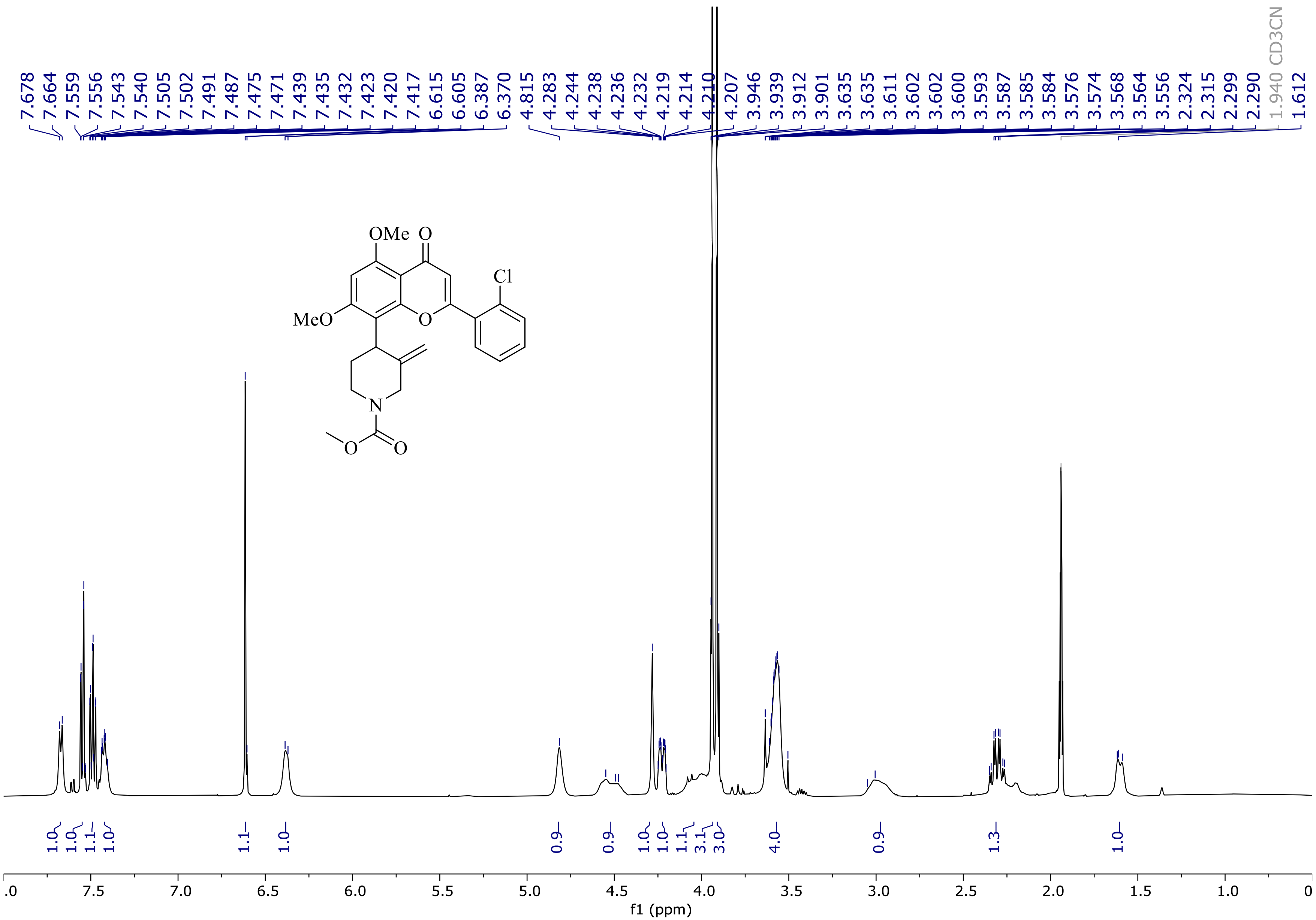

$^{13}C\{^{1}H\}$ NMR spectrum of compound 21 (125 MHz, CD_3CN):

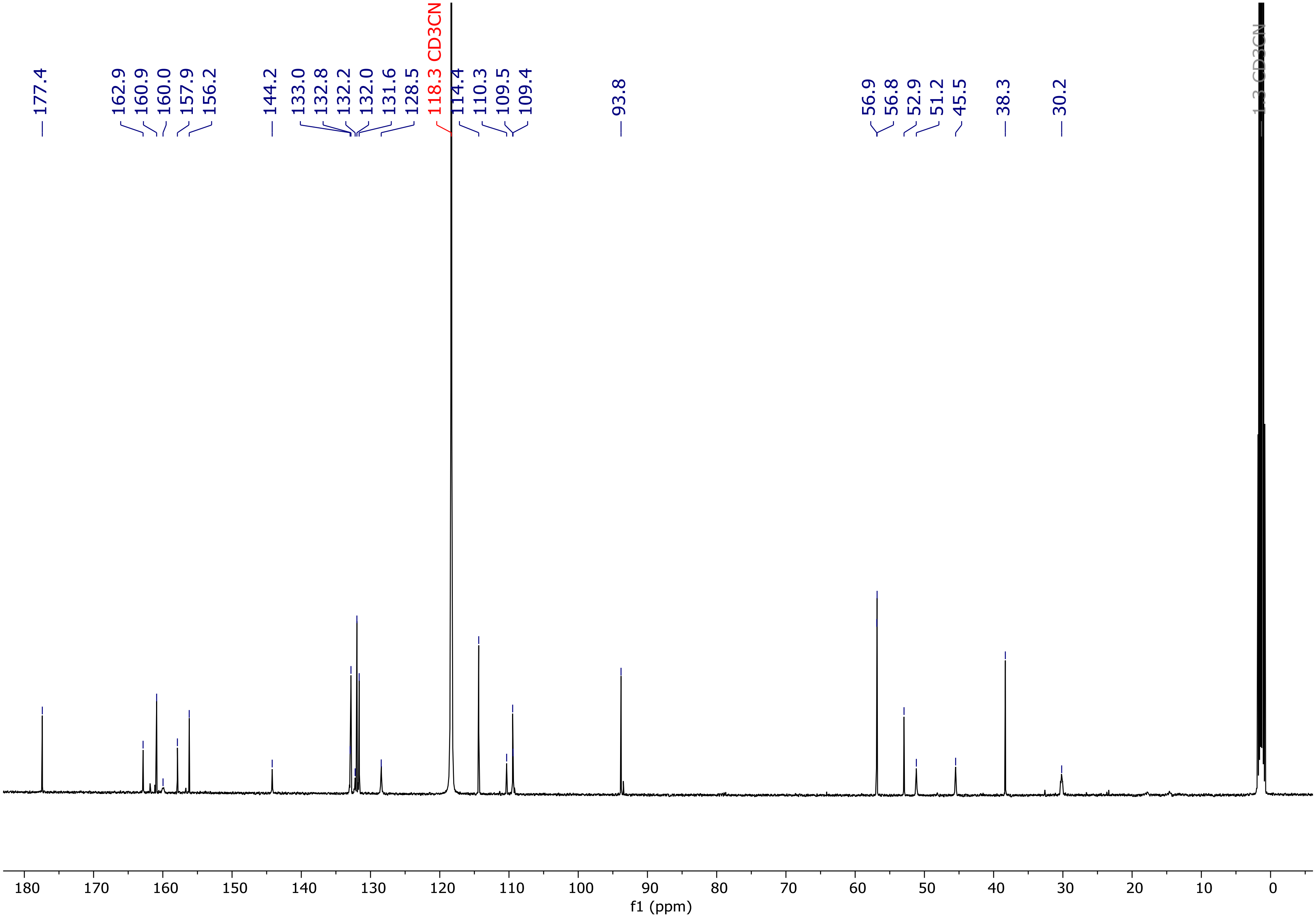

^{1}H NMR spectrum of compound 22 (500 MHz, $CDCl_3$):

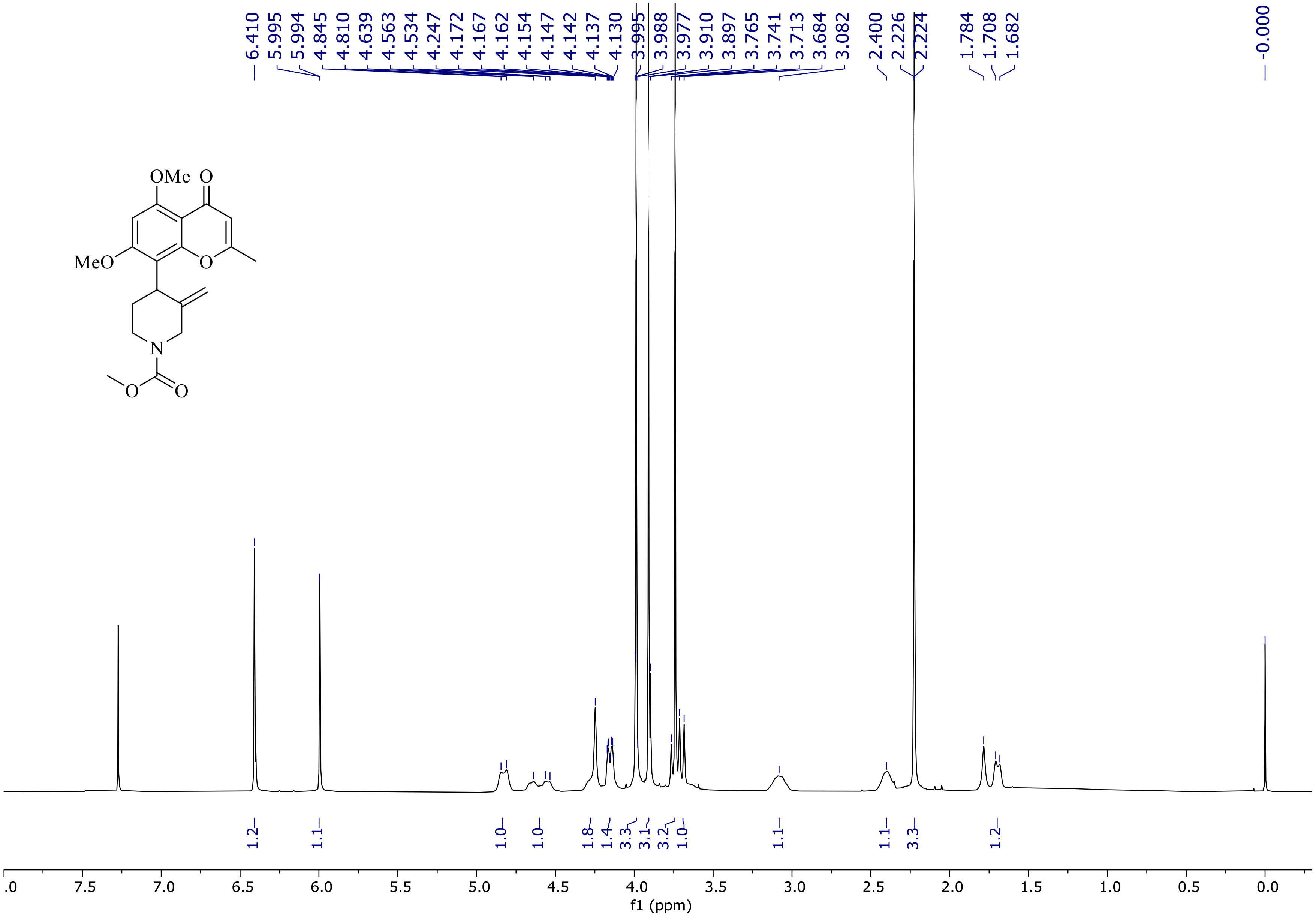

$^{13}C\{^1H\}$ NMR spectrum of compound 22 (125 MHz, $CDCl_3$):

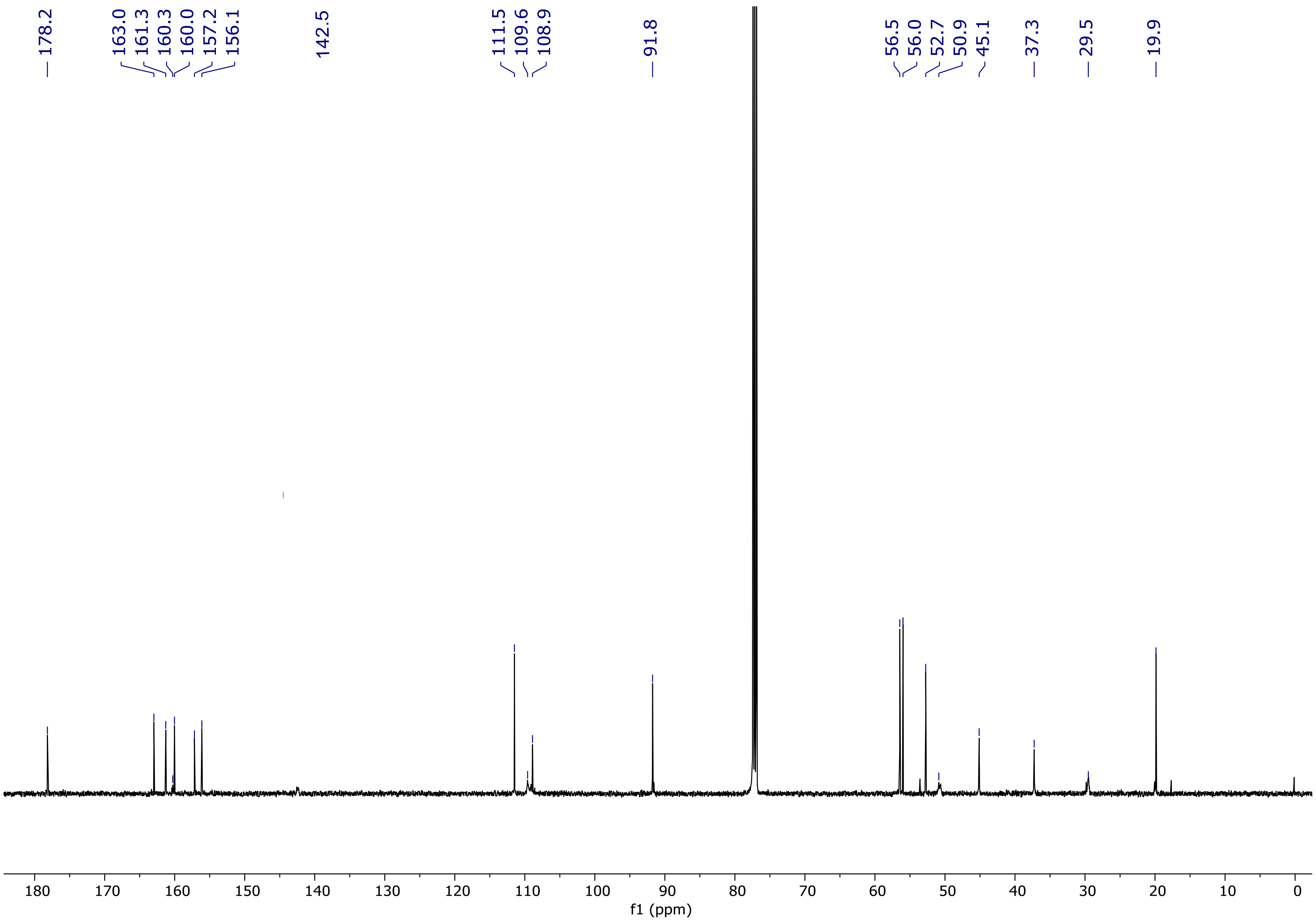

^{1}H NMR spectrum of compound 23 (500 MHz, $CDCl_3$):

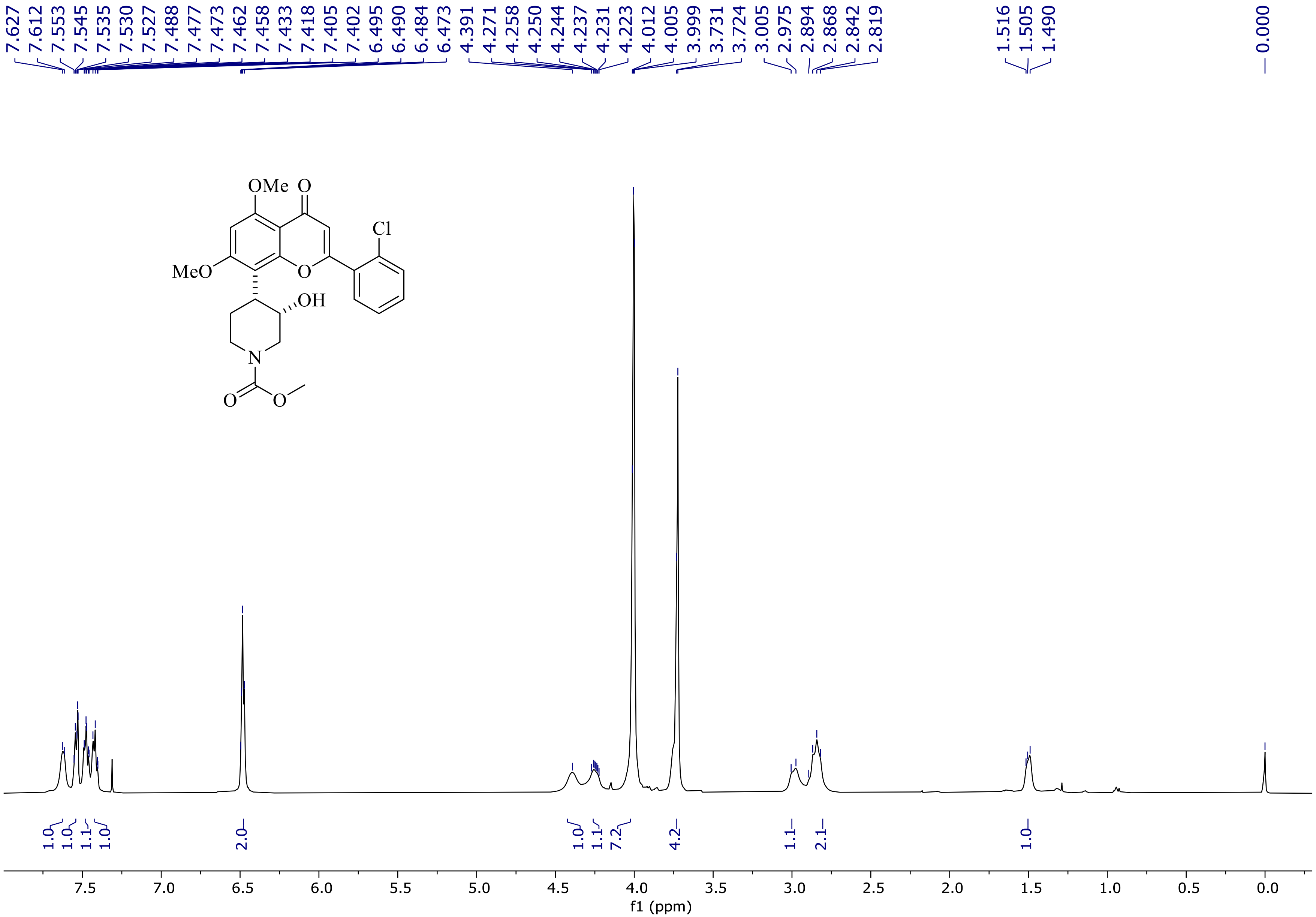

$^{13}C\{^{1}H\}$ NMR spectrum of compound 23 (125 MHz, $CDCl_3$):

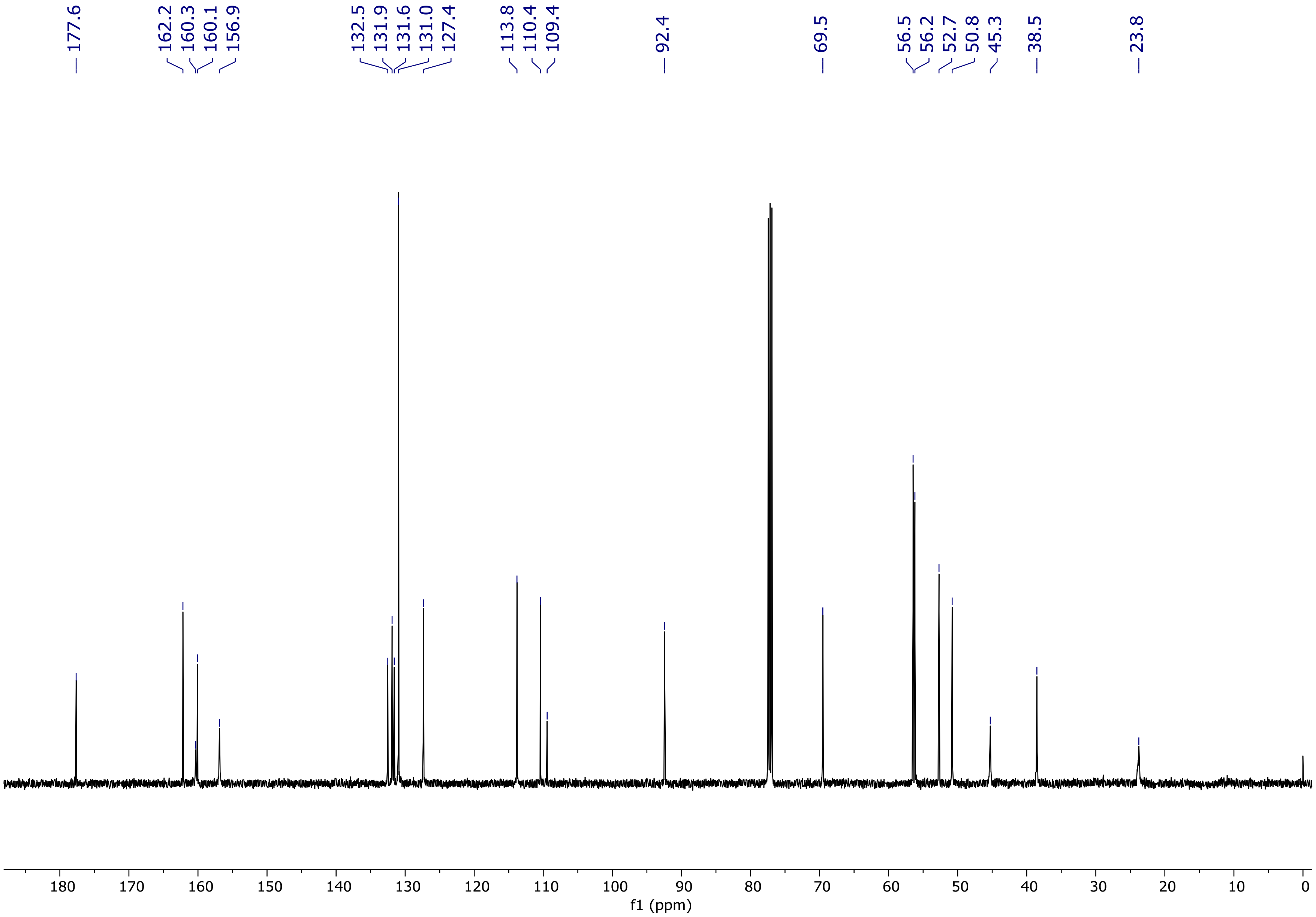

^{1}H NMR spectrum of compound 23 (500 MHz, CD_3CN):

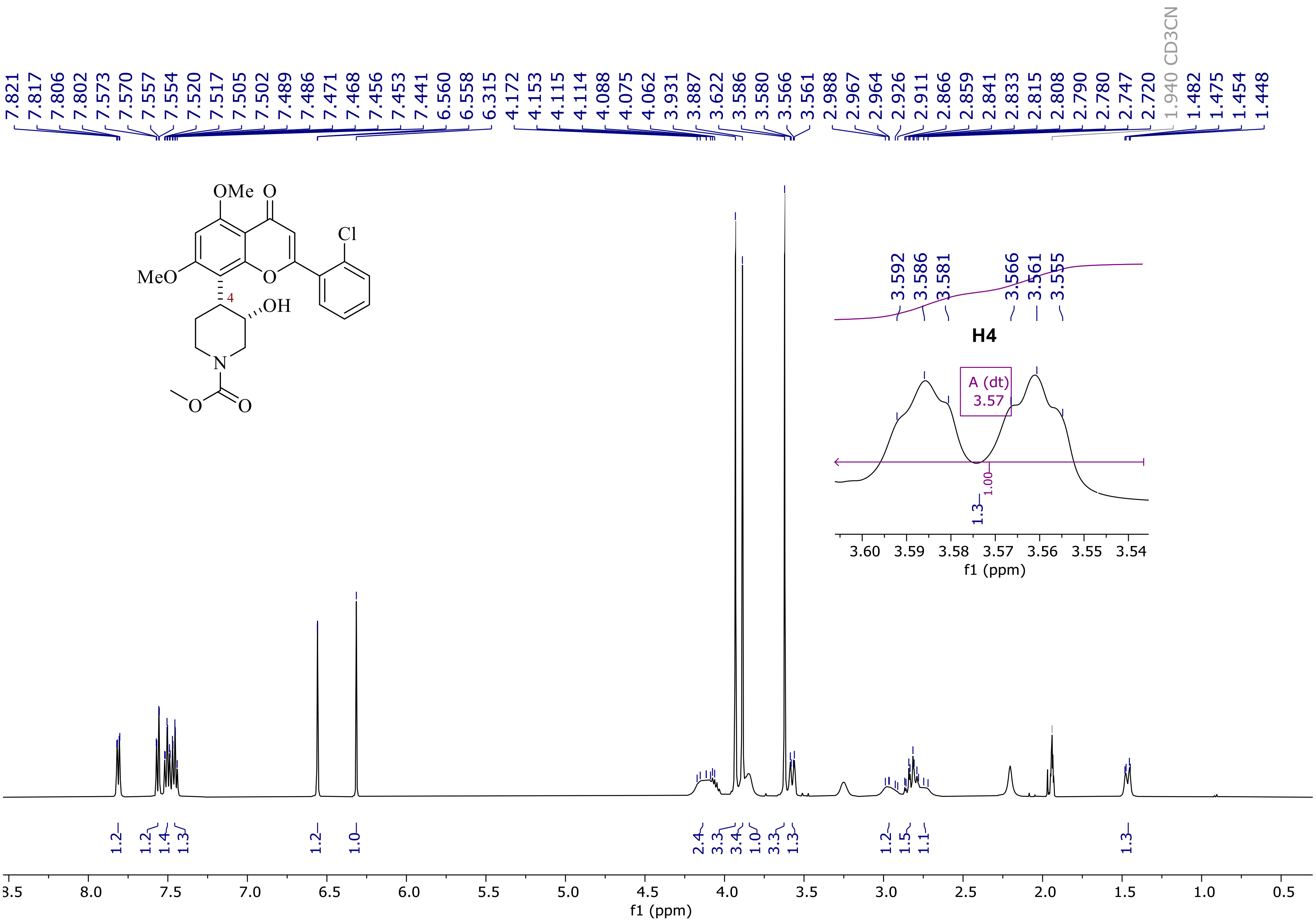

$^{13}C\{^{1}H\}$ NMR spectrum of compound 23 (125 MHz, CD_3CN):

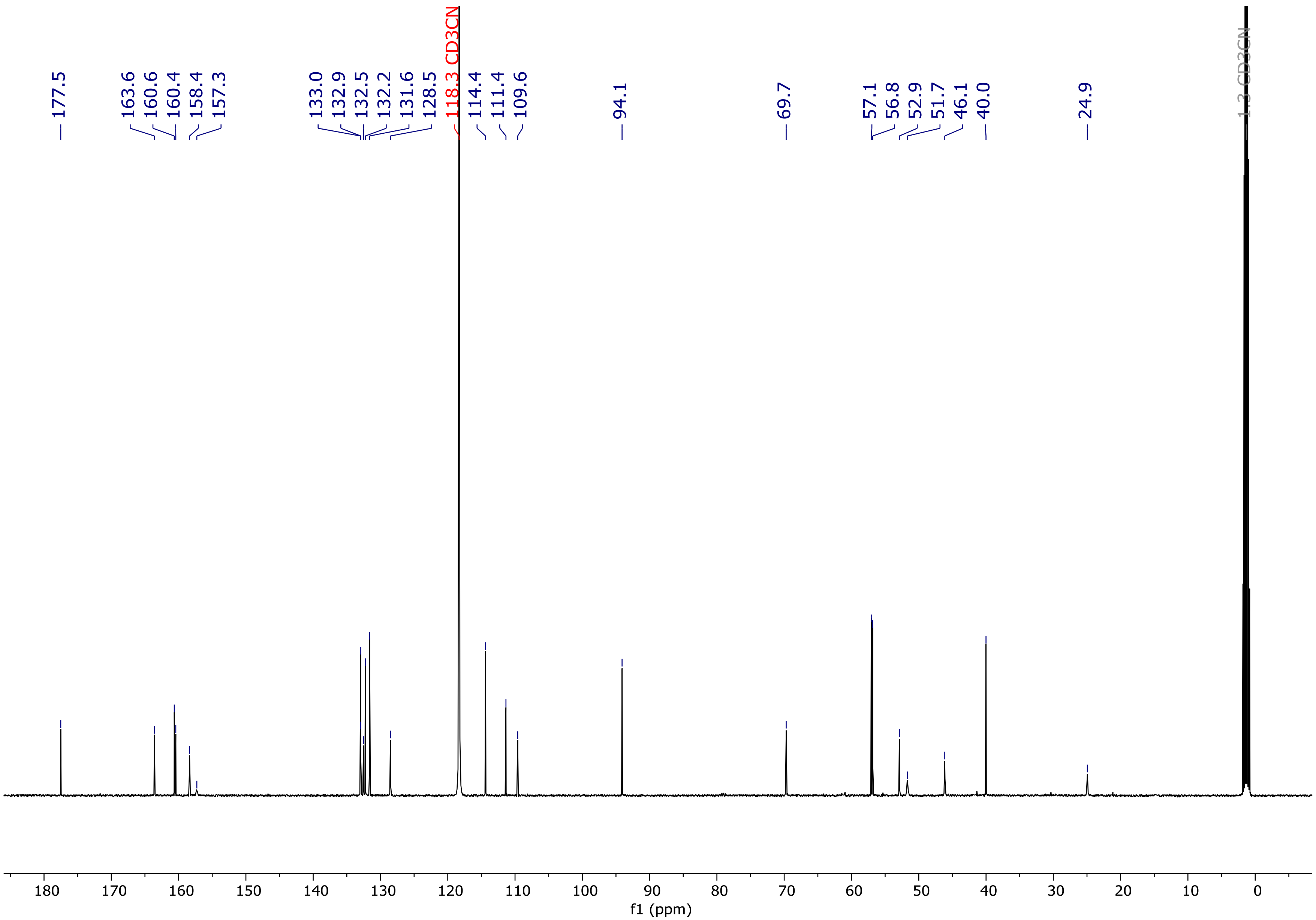

^{1}H NMR spectrum of compound 24 (500 MHz, $CDCl_3$):

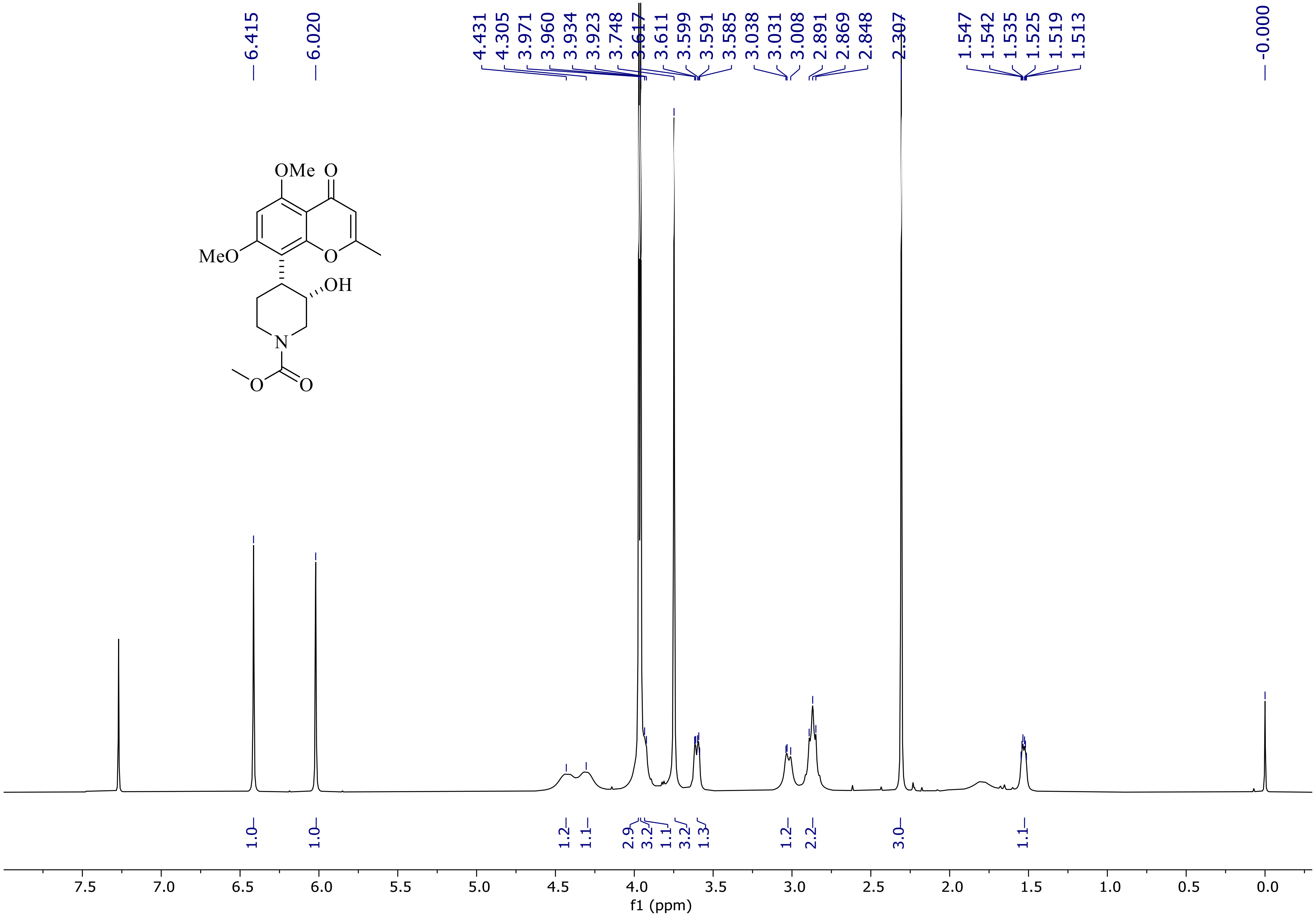

$^{13}C\{^1H\}$ NMR spectrum of compound 24 (125 MHz, $CDCl_3$):

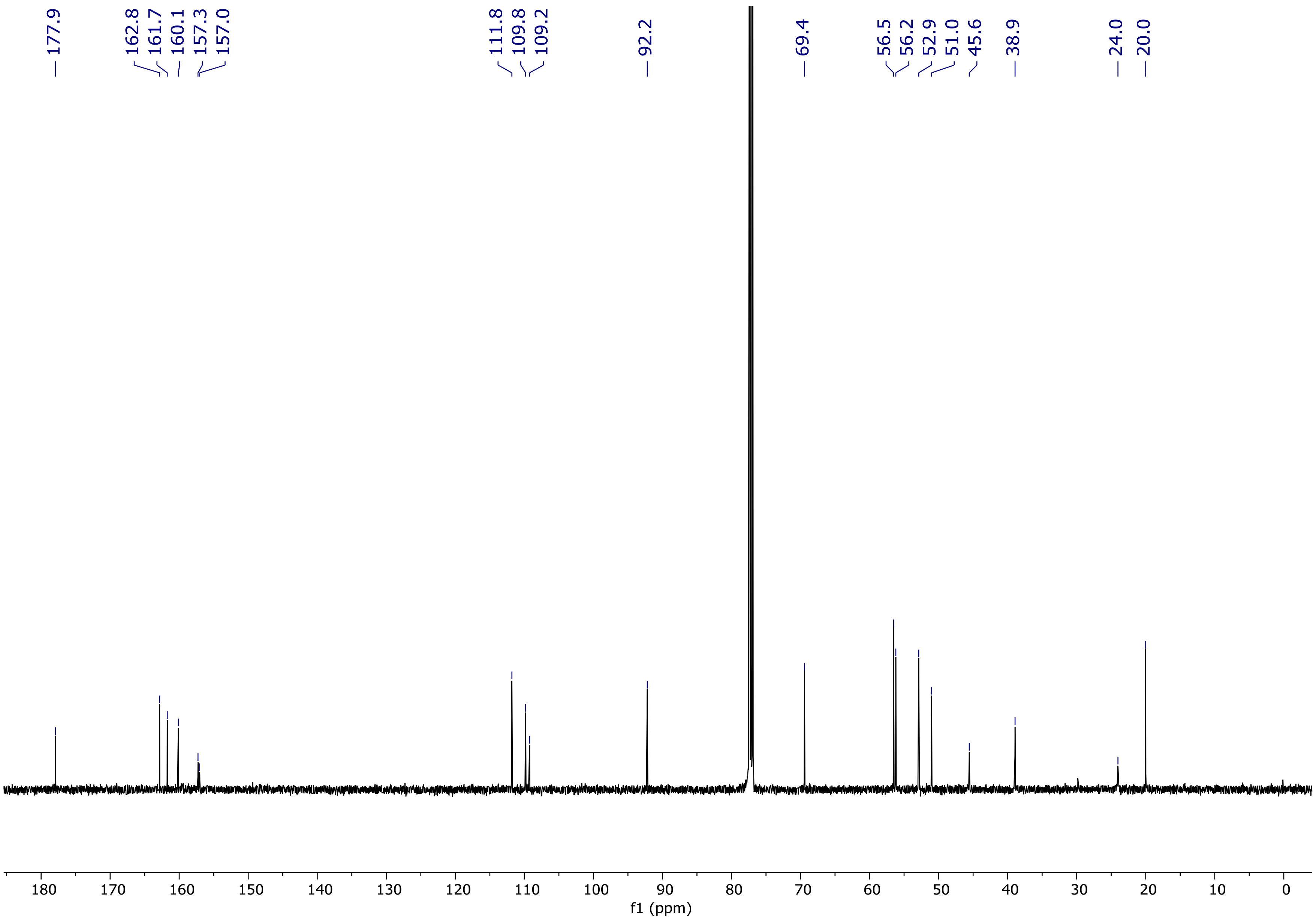

^{1}H NMR spectrum of compound 25 (500 MHz, CD_3CN):

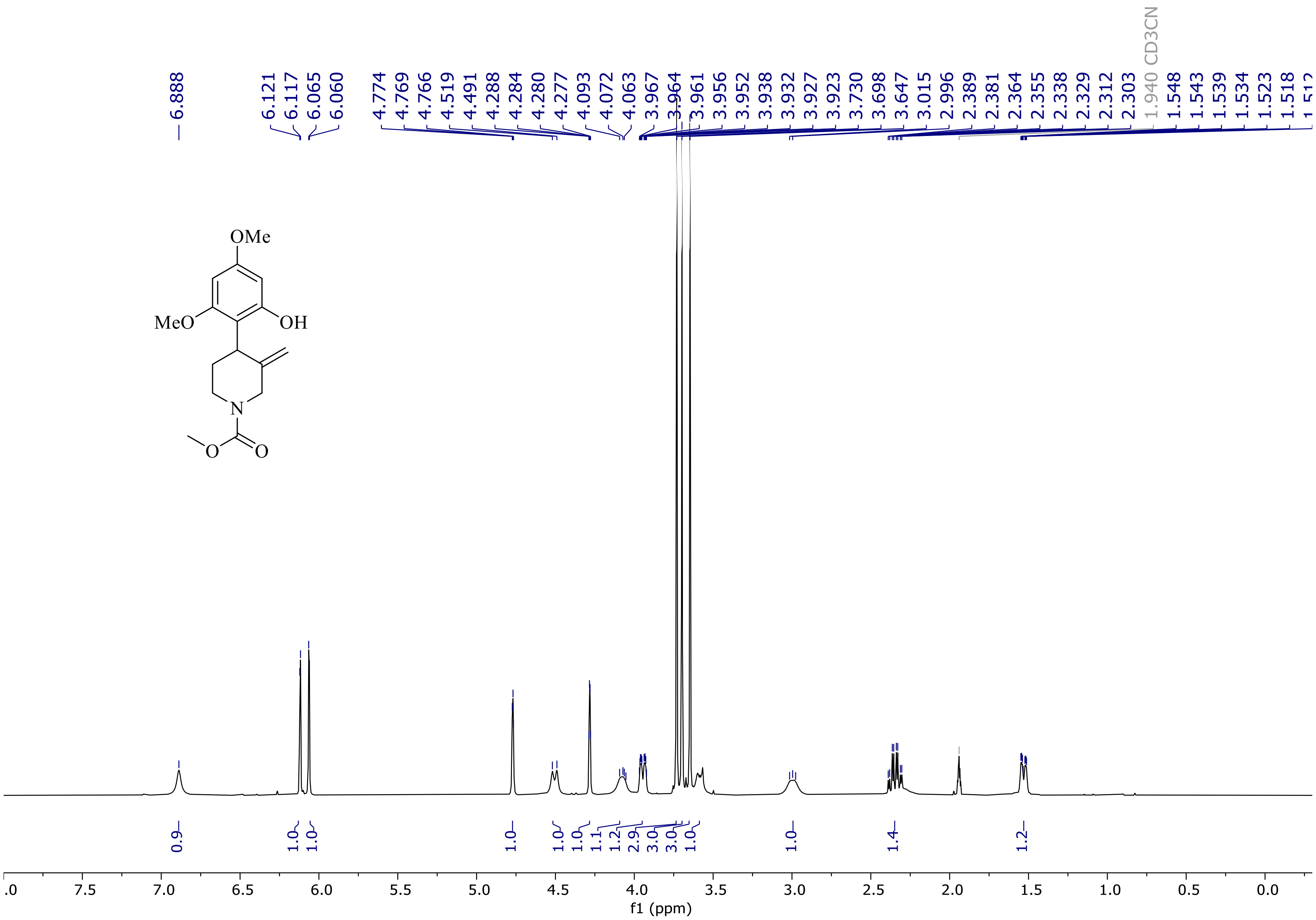

$^{13}C\{^1H\}$ NMR spectrum of compound 25 (125 MHz, CD_3CN):

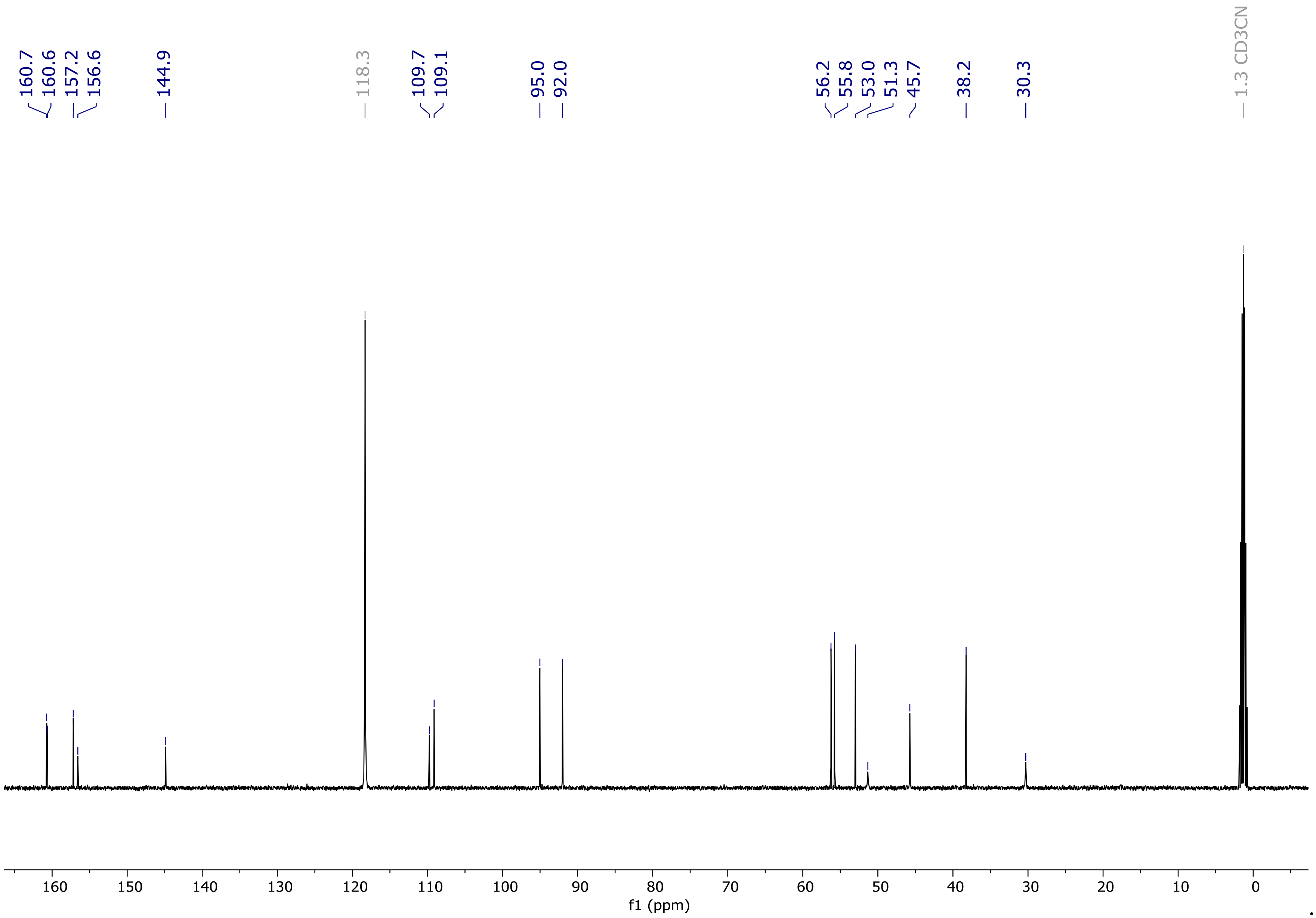

^{1}H NMR spectrum of compound 27 (500 MHz, CDCl₃):

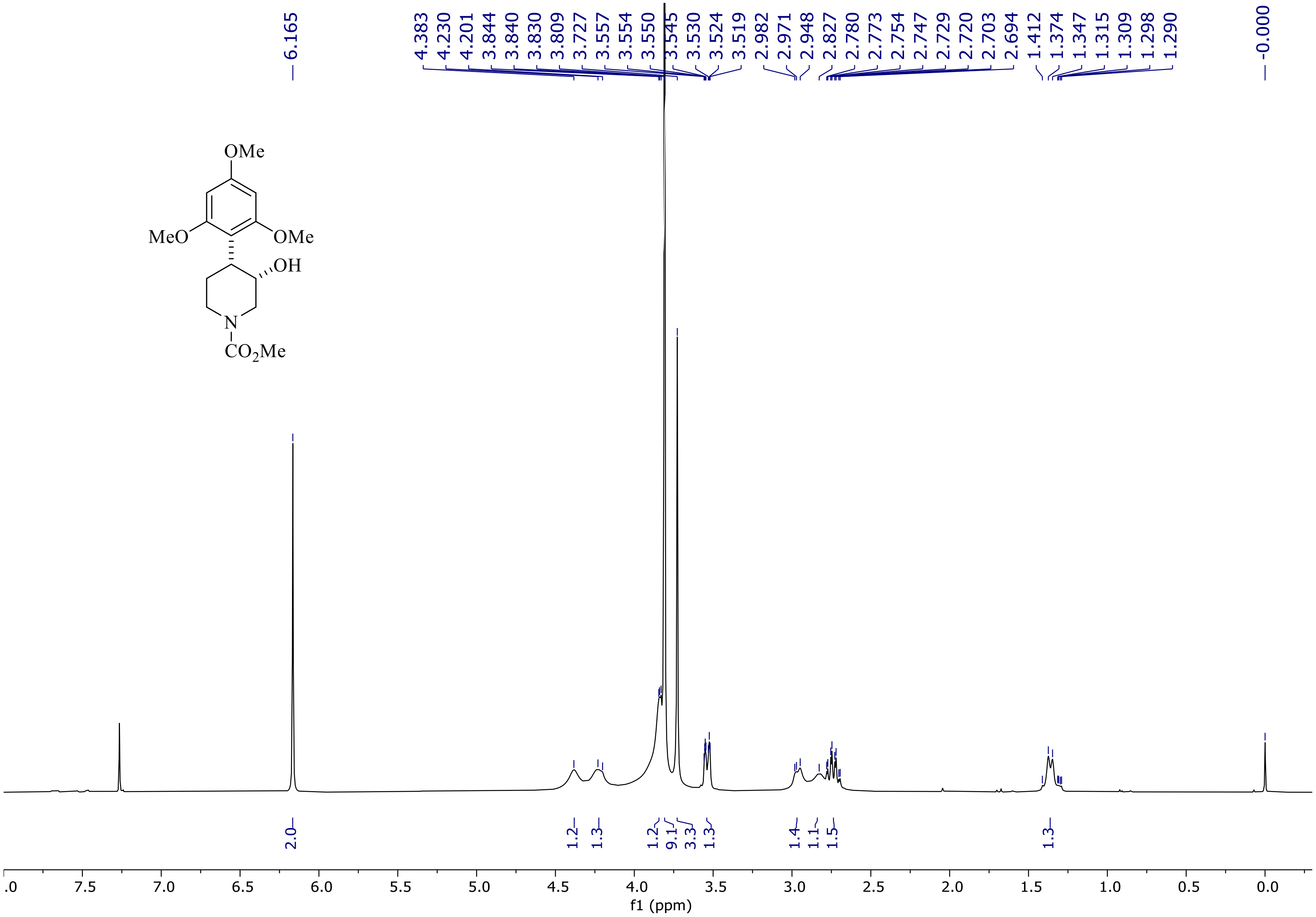

$^{13}C\{^1H\}$ NMR spectrum of compound 27 (125 MHz, $CDCl_3$):

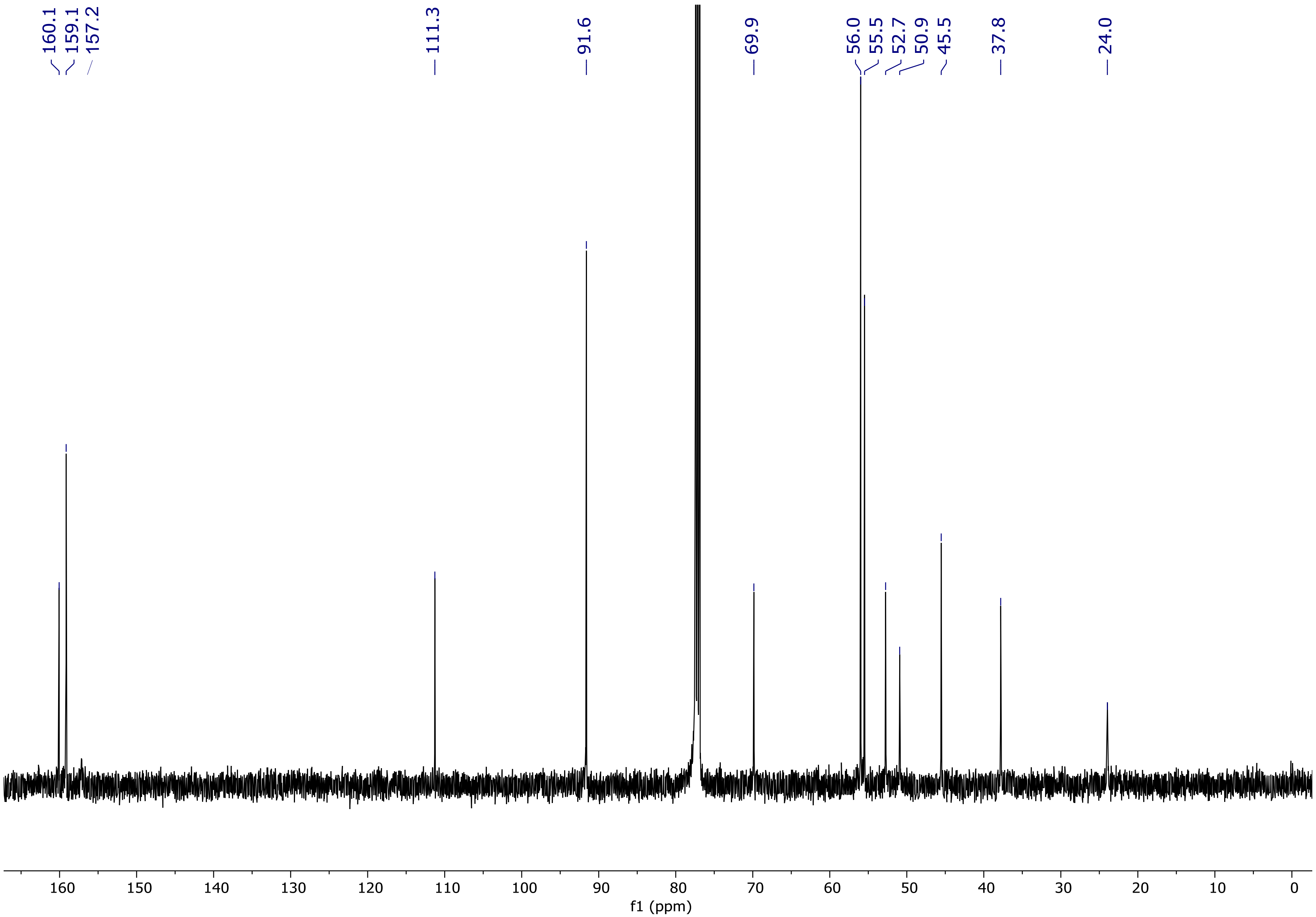

^{1}H NMR spectrum of compound 28 (500 MHz, $CDCl_3$):

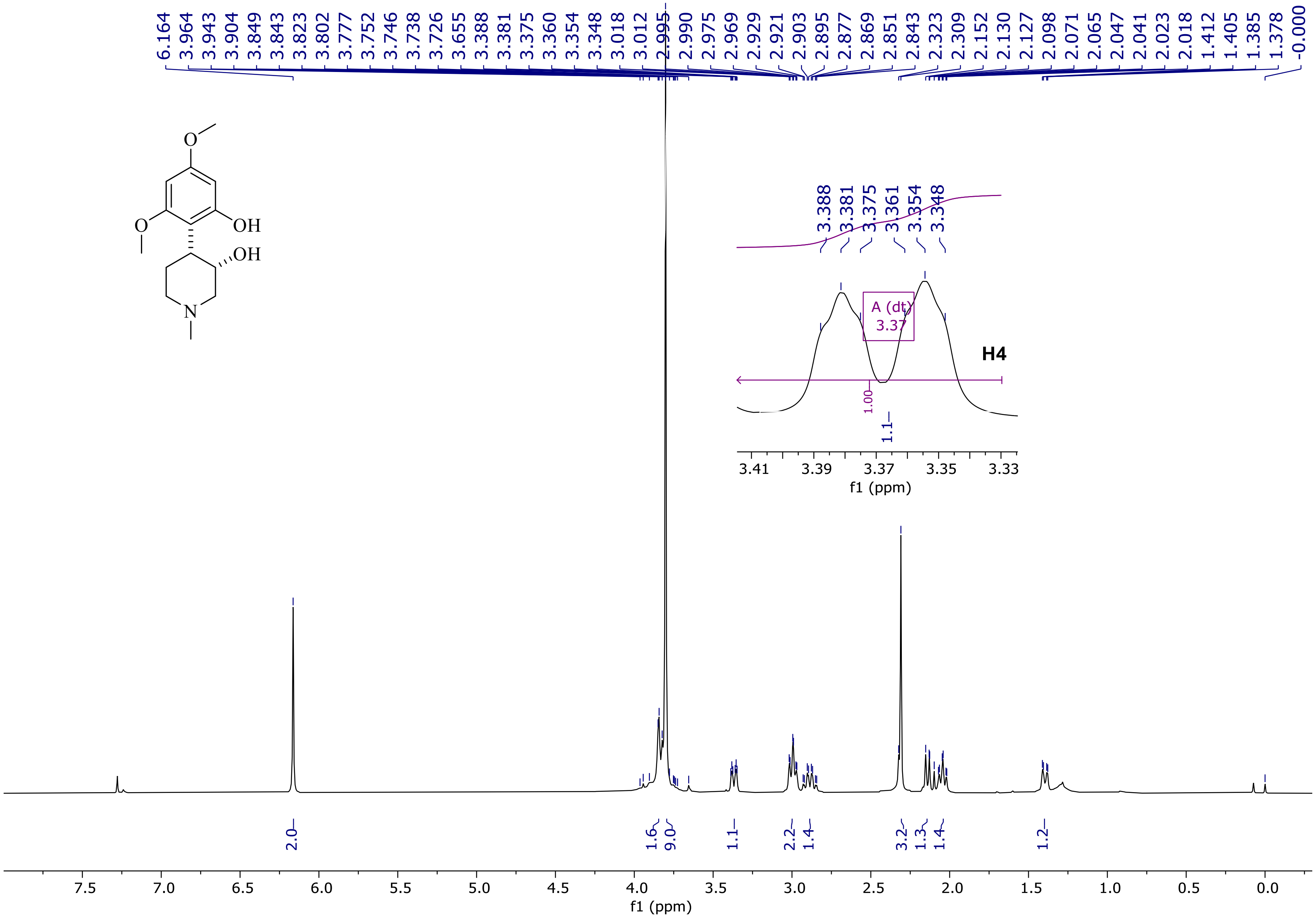

$^{13}C\{^1H\}$ NMR spectrum of compound 28 (125 MHz, $CDCl_3$):

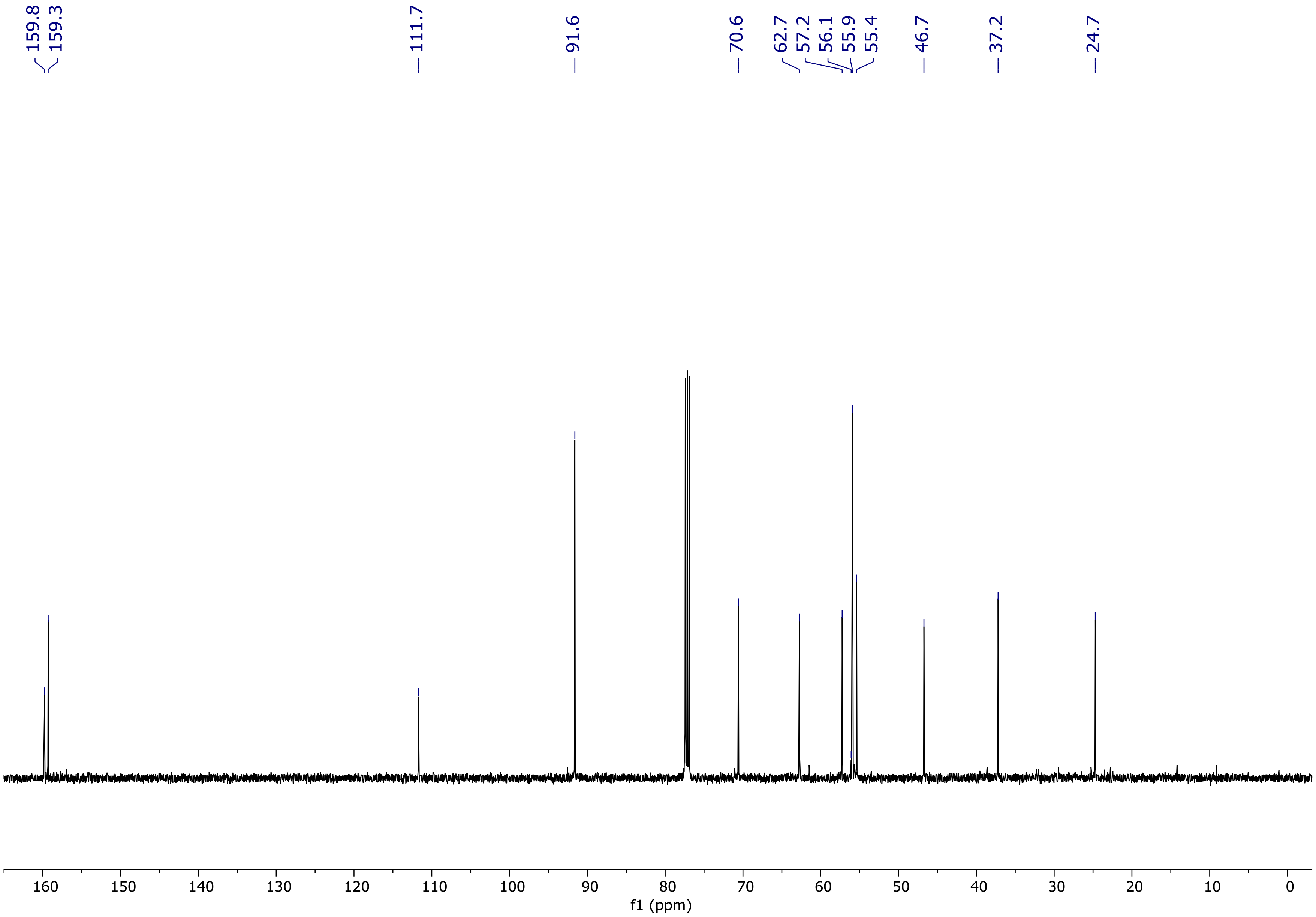

COSY NMR spectrum of compound 28 (500 MHz, $CDCl_3$):

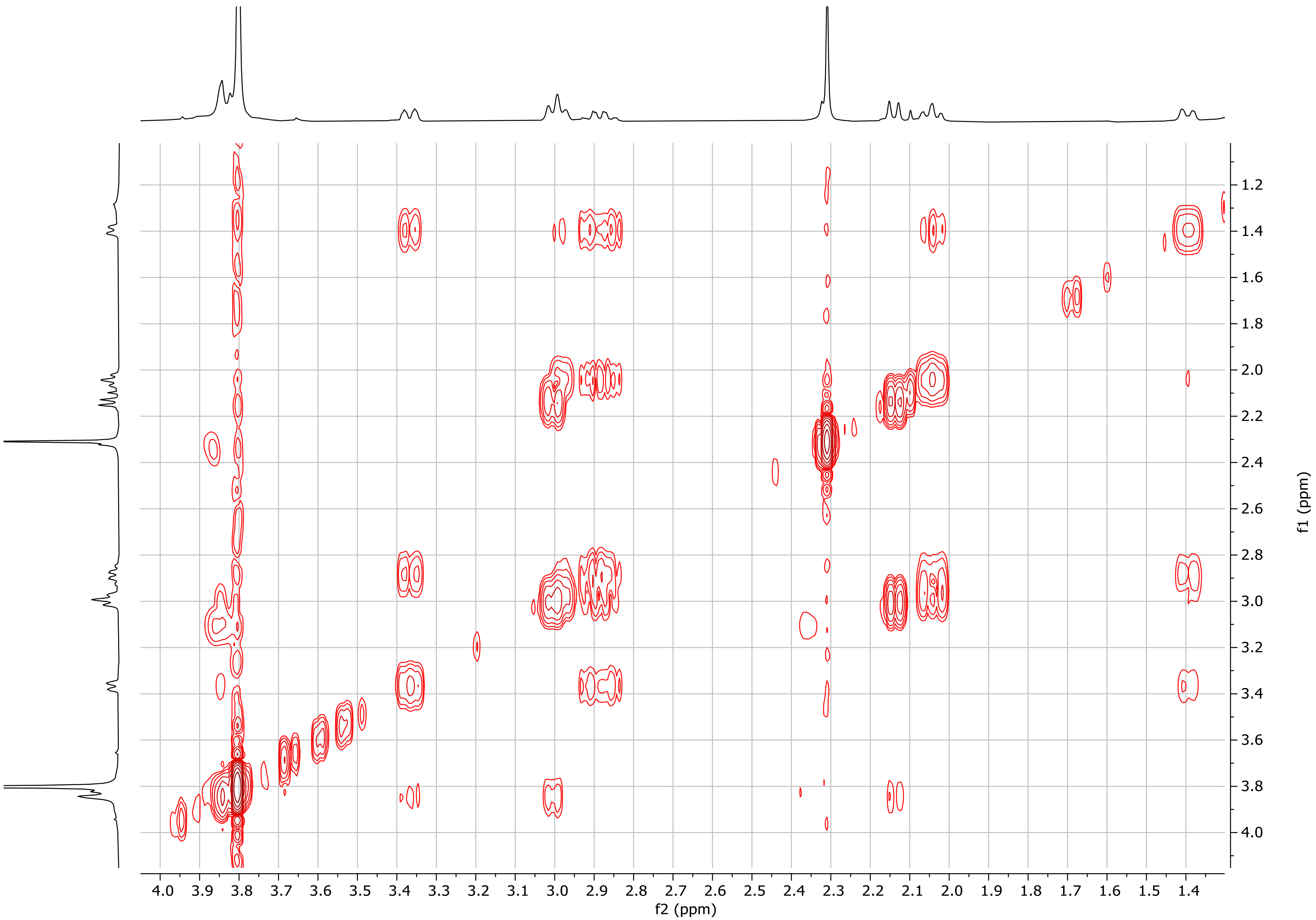

HSQC NMR spectrum of compound 28 (500 MHz, $CDCl_3$):

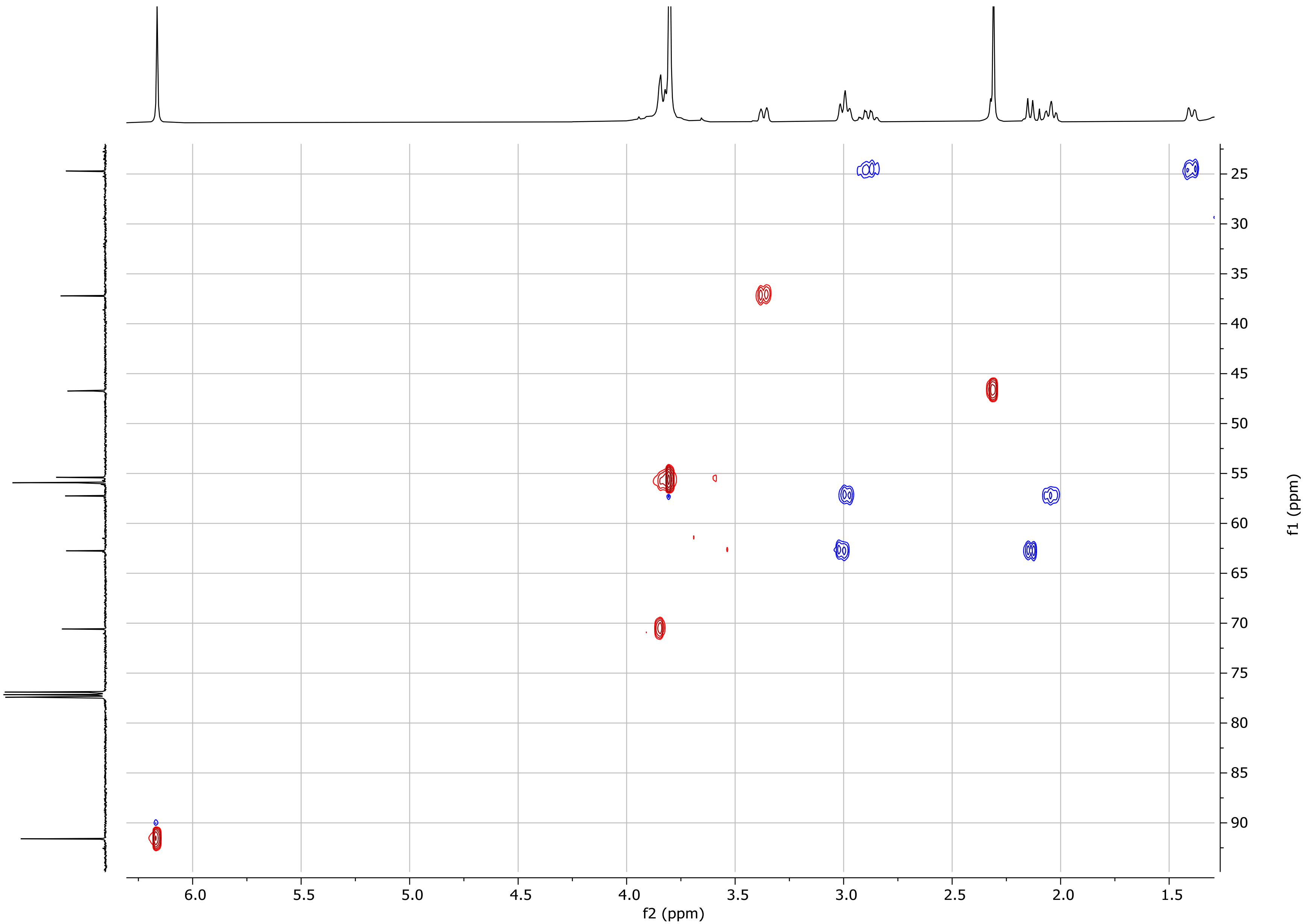

Table 1. NMR data reported by Naik* and our work

Position	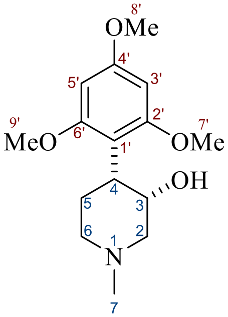 Naik *et. al* (^{1}H NMR 90 MHz, $CDCl_3$)		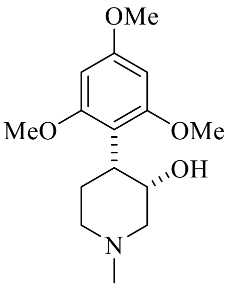 Our research group (^{1}H NMR 500 MHz, $CDCl_3$)		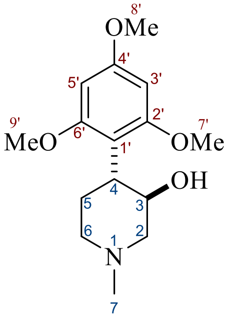 Naik *et. al* (^{1}H NMR 90 MHz, $CDCl_3$)	
	δ ^{13}C	δ ^{1}H	δ ^{13}C	δ ^{1}H	δ ^{13}C	δ ^{1}H
1	-	-	-	-	-	-
2	-	2.1 (m,1H)<br>3.0 (m, 1H)	62.7	2.14 (d, *J* = 12.5 Hz, 1H)<br>2.99 (m, 1H)	-	1.9 (m,1H)<br>3.0 (m, 1H)
**3**	**-**	**3.8 (bs, 1H)**	**70.6**	**3.84 (br, 1H)**	**-**	**4.2 (m, 1H)**
4	-	3.3 (m, 1H)	37.2	3.37 (dt, *J* = 13.5, 3.3 Hz, 1H)	-	3.0 (m, 1H)
5	-	1.4 (m, 1H)<br>3.0 (m, 1H)	24.7	1.40 (dd, *J* = 13.5, 3.5 Hz, 1H)<br>2.89 (qd, *J* = 13.0, 3.9 Hz, 1H)	-	1.55 (m, 2H)
6	-	2.1 (m,1H)<br>3.0 (m, 1H)	57.2	2.04 (td, *J* = 11.9, 2.8 Hz, 1H)<br>2.99 (m, 1H)	-	1.9 (m,1H)<br>3.0 (m, 1H)
7	-	2.25 (s, 3H)	46.7	2.31 (s, 3H)	-	2.30 (s, 3H)
1'	-	-	111.7	-	-	-
2' y 6'	-	-	159.3	-	-	-
3' y 5'	-	6.1 (s, 2H)	91.6	6.16 (s, 2H)	-	6.15 (s, 2H)
4'	-	-	159.8	-	-	-
8'	-	3.8 (bs, 9H)	55.4	3.80 (s, 3H)	-	3.75 (s, 3x 3H)
7' y 9'	-		55.9	3.80 (s, 6H)	-	
OH	-	2.1 (m, 1H)	-	-	-	1.9 (m, 1H)

*Naik et. al only reports ^{1}H NMR spectra without signals assignation.
